# Geometric *E*→*Z* Isomerisation of Alkenyl Silanes by Selective Energy Transfer Catalysis: Stereodivergent Synthesis of Triarylethylenes via a Formal *anti*‐Metallometallation

**DOI:** 10.1002/anie.201910169

**Published:** 2019-10-31

**Authors:** Svenja I. Faßbender, John J. Molloy, Christian Mück‐Lichtenfeld, Ryan Gilmour

**Affiliations:** ^1^ Organisch Chemisches Institut Westfälische Wilhelms-Universität Münster Corrensstraße 40 48149 Münster Germany

**Keywords:** alkenes, catalysis, geometric isomerisation, Hiyama–Denmark coupling, medicinal chemistry

## Abstract

An efficient geometrical *E*→*Z* isomerisation of alkenyl silanes is disclosed via selective energy transfer using an inexpensive organic sensitiser. Characterised by operational simplicity, short reaction times (2 h), and broad substrate tolerance, the reaction displays high selectivity for trisubstituted systems (*Z*/*E* up to 95:5). In contrast to thermal activation, directionality results from deconjugation of the π‐system in the *Z*‐isomer due to A^1,3^‐strain thereby inhibiting re‐activation. The structural importance of the β‐substituent logically prompted an investigation of mixed bis‐nucleophiles (Si, Sn, B). These versatile linchpins also undergo facile isomerisation, thereby enabling a formal *anti*‐metallometallation. Mechanistic interrogation, supported by a theoretical investigation, is disclosed together with application of the products to the stereospecific synthesis of biologically relevant target structures.

## Graphical abstract

**A geometrical** ***E*** **→*Z* isomerisation** of alkenyl silanes proceeding through selective energy transfer is enabled by an organic sensitiser and displays high selectivity for trisubstituted systems. Mixed bis‐nucleophiles (Si, Sn, B) also undergo facile isomerisation, thereby enabling a formal *anti*‐metallometallation.

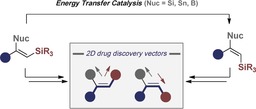

## Introduction

**Scheme 1:**
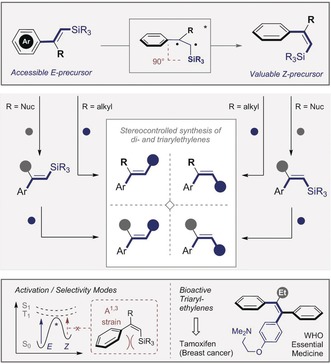
Conceptual framework of the stereodivergent synthesis of di‐ and triarylethylenes by chemoselective, stereospecific cross‐coupling.

**Scheme 2:**
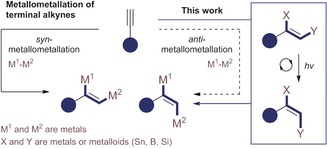
Bypassing the challenge of *anti*‐metallometallation of terminal alkynes.

Stereodivergent routes to highly functionalised alkenes require access to both *E*‐ and *Z*‐geometric isomers of the alkenyl nucleophile to translate the stereochemical information encoded at the substrate level to the product through stereospecific coupling.^1^, ^2^, ^3^ Generating both *E*‐ and *Z*‐substrate isomers is, however, often complicated by mechanistic and/or thermodynamic constraints,^4^ requiring independent synthesis routes to be developed to prepare both substrates. Conceptually, geometric alkene isomerisation^5^ streamlines this problem by allowing pre‐existing synthesis routes to the major (e.g., *E*) isomer to be utilised,^6^ with the addition of a one‐step isomerisation at the end of the sequence (*E*→*Z*). In the case of styrenyl systems that contain an embedded chromophore, energy transfer catalysis offers a practical activation method to achieve efficient geometrical *E→Z* isomerisation.^4^, ^5^, ^7^, ^8^ Predicated on selective energy transfer from an excited‐state photosensitiser to the starting material,^4^, ^5^, ^9^ this platform ensures that only the *E*‐geometric isomer is activated. The resulting intermediate can collapse to either geometric isomer (Scheme 1, inset). As the product cannot be re‐excited, the *Z*‐isomer accumulates thereby mitigating microscopic reversibility.^10^ In an initial assessment of this energy transfer concept to access *Z*‐configured nucleophiles for cross‐coupling and subsequent stereospecific modification, we recently disclosed the geometric isomerisation of styrenyl organoboron systems.8k Despite the high *Z*‐selectivity, efficiency was offset by lengthy reaction times and an expensive Ir^III^ photocatalyst. Moreover, attempts to develop a chemoselective transmetallation of bis‐nucleophiles for stereodivergent synthesis were unsuccessful. The latter challenge is pertinent given the practical importance of *syn*‐metallometallation in alkyne functionalisation, and the preparative advantages of realising a formal *anti*‐metallometallation by geometric isomerisation (Scheme 2).^11^

To validate this platform for the stereodivergent synthesis of triarylethylenes from a common precursor, alkenyl silanes that are synonymous with the venerable Hiyama–Denmark coupling^12^ were investigated (Scheme 1). Elegant studies by Denmark and co‐workers have unequivocally established the stereospecific nature of this palladium‐mediated transformation rendering it ideal for this task.2f, 2h, 13 These studies also highlight the fact that trisubstituted *Z*‐substrates are more laborious to prepare than their *E*‐congeners.2e–2h Although numerous strategies exist to access *Z*‐configured alkenyl silanes,^14^ the emphasis remains largely on 1,2‐disubstituted systems. A geometric isomerisation of multiply substituted *E*‐alkenyl silanes to the corresponding Z‐isomers would thus complement the existing pallet of methods. Herein, an operationally facile *E*→*Z* isomerisation of alkenyl silanes has been developed by selective energy transfer catalysis.^9^ Both geometric isomers can be further functionalised by stereospecific transformations. Furthermore, by introducing a second metal or metalloid (R=Nuc=Sn, B, Si), sequential orthogonal coupling enables the stereocontrolled synthesis of triarylethylenes from a single scaffold: This constitutes a formal *anti*‐metallometallation.

## Results and Discussion

To identify suitable conditions for the title transformation, the photoisomerisation of alkenyl silane ***E*** **‐1** to ***Z*** **‐1** was investigated (Table 1). Initially, direct irradiation of ***E*** **‐1** at 365 nm was attempted in the absence of a catalyst. Whilst this led to partial isomerisation (*Z*/*E* 12:88) after 24 h, the need for a photocatalyst was apparent. To that end, the reaction was explored in the presence of a variety of small‐molecule catalysts. In all cases, 5 mol % catalyst loading was employed, and the *Z*/*E* ratios were determined by ^1^H NMR spectroscopy.

**Table 1:** Optimisation of the photocatalytic isomerisation of ***E*** **‐1**→***Z*** **‐1**.^[a]^ 
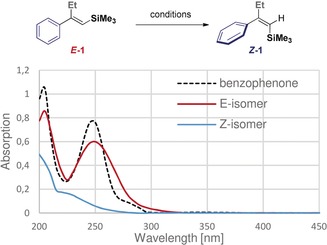

Entry	Catalyst	Solvent	*t* [h]	Irradiation wavelength [nm]	*Z*/*E* ratio^[b]^
1	–	cyclohexane	24	365	12:88
2	(−)‐riboflavin	MeCN	24	450	0:100
3	Ir(ppy)_3_	MeCN	24	450	48:52
4	benzil	MeCN	24	402	1:99
5	thioxanthone	cyclohexane	24	402	89:11^[c]^
6	benzophenone	MeCN	24	365	93:7^[c]^
7	benzophenone	cyclohexane	24	365	95:5
8	benzophenone	cyclohexane	12	365	95:5
9	benzophenone	cyclohexane	6	365	95:5
10	benzophenone	cyclohexane	2	365	95:5

[a] Reactions were performed in degassed solvent on a 0.1 mmol scale at ambient temperature under argon atmosphere, using 5 mol % of the catalyst. [b] *Z*/*E* selectivity determined by ^1^H NMR spectroscopy. [c] ^1^H NMR spectrum of the crude reaction mixture confirmed partial decomposition.

Whereas irradiation at >400 nm with various photocatalysts proved ineffective (entries 2–5), the reaction with benzophenone at 365 nm efficiently catalysed the isomerisation of ***E*** **‐1** to ***Z*** **‐1** (*Z*/*E=*93:7, entry 6; please see the absorption spectra in Table 1, inset).

**Figure 1:**
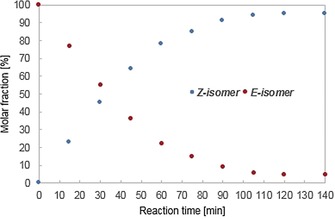
Progress monitoring for the reaction of ***E*** **‐1** to ***Z*** **‐1** by HPLC analysis using a CHIRACEL OJ H column; hexane/^i^PrOH=99:1, 0.5 mL min^−1^, *t*_R_(***E*** **‐1**)=7.2 min, *t*_R_(***Z*** **‐1**)=6.4 min.

Performing the reaction in acetonitrile led to small, but detectable, substrate degradation. However, changing the reaction medium to cyclohexane resolved this issue and further enhanced the selectivity (*Z*/*E*=95:5, entry 7). Furthermore, the reaction time could be reduced to only 2 hours (entries 8–10) with no impact on selectivity or efficiency. Confirmation that the process proceeds in a clean and highly selective manner is evident from reaction progress analysis by HPLC (Figure 1).

Having identified suitable photoisomerisation reaction conditions, the scope and limitations of the transformation were investigated (Table 2). Specifically, a process of substrate editing was initiated to establish the effect of the β‐substituent, the aryl group, and the silyl motif on efficiency. This test set of alkenyl silanes were then sequentially exposed to the standard isomerisation conditions.

**Table 2:** Establishing substrate scope.^[a]^ 
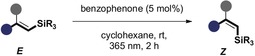

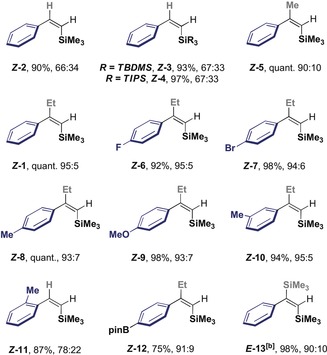

[a] Reactions were performed in degassed cyclohexane on a 0.1 mmol scale at ambient temperature using 5 mol % benzophenone and irradiated for 2 h (365 nm); *Z:E* selectivity was determined by ^1^H NMR spectroscopy; yields refer to isolated, purified mixtures of *E*‐ and *Z*‐isomer. [b] Product isomer labelled as (*E*), based on the higher IUPAC priority of Si than C.

Initially, α,β‐unsubstituted alkenyl silanes (R^1^=H) were investigated. As reaction selectivity is predicated on deconjugation through allylic strain in the product (Scheme 1, lower left),8f these scaffolds lacking a sufficiently bulky R^1^ group were expected to be most challenging. Remarkably, however, moderate levels of selectivity favouring the *Z*‐isomer were observed (*Z*/*E* 66:34, ***Z*** **‐2**). Increasing the steric demand of the silyl group from TMS to TBDMS and TIPS (***Z*** **‐3** and ***Z*** **‐4**, respectively) was well tolerated and led to comparable yields and selectivities. As a control experiment, ***Z*** **‐4** was independently subjected to the standard reaction conditions, which led to the same photostationary composition as that of the *E*‐isomer. Comparison with the β‐Me and β‐Et species, which reacted with high levels of stereoselectivity (***Z*** **‐5** and ***Z*** **‐1,** *Z*/*E* 90:10 and 95:5, respectively), further underscores the importance of the β‐substituent to induce A^1,3^‐allylic strain. This translates directly to high *Z*‐selectivity. Electronic modulation of the aryl ring at the *para* position was well tolerated. Examples that include electron‐deficient halides (***Z*** **‐6** and ***Z*** **‐7**) and electron‐rich motifs (***Z*** **‐8** and ***Z*** **‐9**) consistently furnished excellent *Z*/*E* ratios (up to 95:5).

Introducing a substituent at the *meta* position of the ring had no tangible effect on the stereoselectivity (***Z*** **‐10**, 95:5). To further explore the effect of A^1,3^‐strain, the *ortho*‐methyl derivative ***E*** **‐11** was synthesised and subjected to the reaction. Interestingly, this modification led to a notable increase in selectivity (*Z*/*E* 78:22) when compared to ***Z*** **‐2** (*Z*/*E* 78:22 vs. 66:34). To expand the synthetic versatility of the substrate scope, the *para*‐Bpin compound ***Z*** **‐12** and bis‐silyl species ***E*** **‐13** were accessed to provide a platform for orthogonal and bidirectional coupling. Gratifyingly, smooth and efficient isomerisation was observed in both scenarios with excellent levels of geometric control (*Z*/*E* 91:9 and *E*/*Z* 90:10, respectively; note that ***E*** **‐13** reflects the higher IUPAC priority of Si over C).

**Scheme 3:**
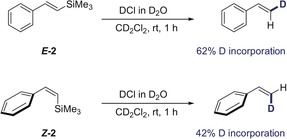
Stereospecific deuteration of *E*‐ and *Z*‐alkenyl silanes. Complete consumption of the starting materials observed.

Prior to extending the study to a broader scope of silane derivatives for subsequent cross‐coupling, deuteration of ***E*** **‐** and ***Z*** **‐2** was performed to ensure that the stereochemical information in the substrate would be efficiently translated to the product (Scheme 3). In line with seminal studies by Fleming and co‐workers,^15^ independently exposing the geometric isomers to DCl in D_2_O led to 62 % and 42 % deuterium incorporation, respectively.

**Scheme 4:**
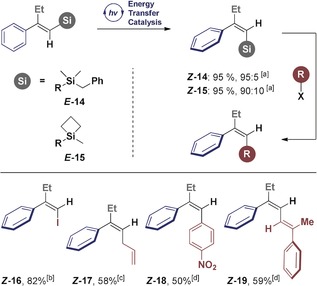
Isomerisation of benzylsilanes and siletanes, and stereospecific derivatisation. [a] Standard conditions for the isomerisation: Silane (1.0 equiv), benzophenone (5 mol %), cyclohexane, 2 h, rt, 365 nm. [b] Reaction conditions: ***Z*** **‐15** (1.0 equiv), *N*‐iodosuccinimide (1.2 equiv), MeCN, 20 min, rt. [c] ***Z*** **‐14** (0.1 equiv), allyl acetate (8.0 equiv), TBAF (1.0 m in THF, 6.2 equiv), Pd_2_(dba)_3_⋅CHCl_3_ (0.1 equiv), THF, rt, 18 h. [d] ***Z*** **‐15** (1.2 equiv), aryl or alkenyl iodide (1.0 equiv), TBAF⋅3 H_2_O (3.0 equiv), Pd(dba)_2_ (7.5 mol %), THF, rt, 18 h.

Motivated by the venerable history of benzylsilanes and siletanes in the evolution of cross‐coupling,13a substrates ***E*** **‐14** and ***E*** **‐15** were investigated. Both substrate classes can be converted into more reactive heterofunctional silanes upon activation with a fluoride source, rendering their inclusion in this study valuable (Scheme 4).^16^ Pleasingly, both ***E*** **‐14** and ***E*** **‐15** were excellent substrates for this transformation (*Z*/*E* 95:5 and 90:10, respectively). To demonstrate the utility of the protocol towards scale‐up, both reactions were performed on a 1 mmol scale with no erosion of stereoselectivity. Representative functionalisations of ***Z*** **‐14** included a Hiyama–Denmark coupling to generate the skipped diene ***Z*** **‐17** in 58 % with no loss of stereochemical integrity. Siletane ***Z*** **‐15** was smoothly processed to the *Z*‐configured stilbene ***Z*** **‐18** and the conjugated diene ***Z*** **‐19** (59 %). Finally, umpolung of the alkenyl nucleophile ***Z*** **‐15** to generate the electrophilic species ***Z*** **‐16** proved facile (82 %).

**Scheme 5:**
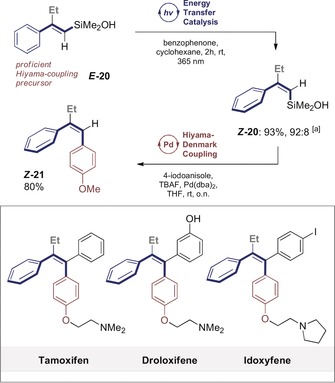
Isomerisation of alkenyl silanol ***E*** **‐20** and stereospecific coupling. [a] *Z*/*E* selectivity determined by ^1^H NMR spectroscopy.

To further extend the scope of the isomerisation, a model silanol (***E*** **‐20**) was investigated because of the efficiency of these systems in stereospecific Hiyama–Denmark coupling processes.2f, 17 Exposure to the standard conditions developed in Table 1 furnished ***Z*** **‐20** in 93 % yield and with a *Z*/*E* ratio of 92:8. As a coupling partner for the Hiyama–Denmark reaction, 4‐iodoanisole was selected because of the structural importance of ether units in bioactive triarylethylenes, such as in Tamoxifen, Droloxifene, Clomifene, and Idoxyfene (Scheme 5).^18^ Gratifyingly, stilbene ***Z*** **‐21** was isolated in 80 % yield as a single geometric isomer.

**Scheme 6:**
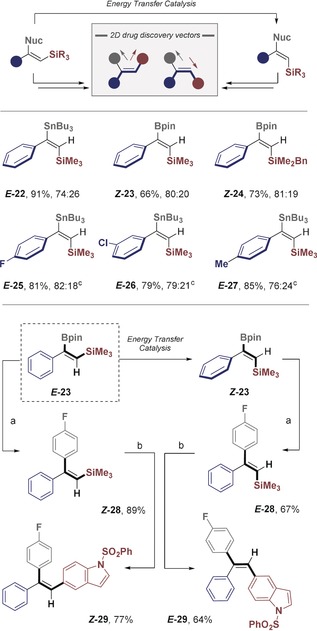
Stereocontrolled construction of triarylethylenes ***Z*** **‐** and ***E*** **‐29** from a single precursor (***E*** **‐23**). Isomerisation reactions were performed on the a 0.1 mmol scale under standard conditions. Reagents and conditions: a) styrenyl BPin (1.0 equiv), aryl bromide (1.05 equiv), Pd(OAc)_2_ (5 mol %), SPhos (10 mol %), K_3_PO_4_ (3.0 equiv), H_2_O (5 equiv), 1,4‐dioxane, 80 °C, 4 h; b) styrenyl silane (1.0 equiv), NIS (1.2 equiv), MeCN, rt, 16 h; then styrenyl iodide (1.0 equiv), arylBPin (1.05 equiv), Pd(OAc)_2_ (5 mol %), SPhos (10 mol %), K_3_PO_4_ (3.0 equiv), H_2_O (5.0 equiv), 1,4‐dioxane, 80 °C, 4 h; c) reactions performed using thioxanthone as the catalyst.

To extend the methodology to include triarylethylene scaffolds, which are privileged in medicinal chemistry, the feasibility of alkenyl bis‐nucleophiles (R=Si, Sn, B) was examined. As delineated in the introduction, alkenyl silanes containing an adjacent nucleophile would provide a formal solution to the *anti*‐metallometallation challenge, and thus streamline routes for the chemoselective, stereocontrolled construction of triarylethylenes (Scheme 6, top).

The generation of bis‐nucleophilic species by *syn*‐addition to phenylacetylene is well‐established with complete regiocontrol.^19^ Despite these advances in regiocontrol, achieving geometric control (formal *anti*‐products) remains conspicuously challenging.^20^ Facilitated by the ease of substrate preparation, various β‐Sn‐ and β‐BPin‐substituted *E*‐alkenyl silanes were prepared and subjected to the isomerisation conditions (Scheme 6, middle).

Despite significant changes to both the electronic and steric profiles of the substrates, efficient isomerisation was observed, thereby providing a solution to this intractable problem. The TMS‐substituted alkene ***Z*** **‐22** bearing a stannyl residue as well as boronic acid pinacol ester ***E*** **‐23** were smoothly converted into the corresponding easily isolable geometric isomers. Variation of the silyl rest was also tolerated as indicated by the benzylsilane (***E*** **‐24**→***Z*** **‐24**, 81:19). Electronic modulation of the aryl ring by *para* and *meta* substitution had little influence on the selectivity of β‐Sn derivatives, and incorporation of a *p*‐chloro group on the phenyl group provides a third vector for subsequent functionalisation (***E*** **‐25**–**27**). Finally, to validate the utility of this transformation, the stereocontrolled synthesis of both geometric isomers of triarylethylene **29** was conducted (Scheme 6, bottom).

Substrate ***E*** **‐23** containing the β‐BPin alkenyl silane motif served as a convenient starting point for this endeavour. Exposure to benzophenone under irradiation rapidly furnished the easily isolable ***Z*** **‐23**. Isomers ***E*** **‐23** and ***Z*** **‐23** were then independently subjected to Suzuki–Miyaura conditions, resulting in β‐arylation with high yields (***Z*** **‐28** and ***E*** **‐28**). Finally, iodination and coupling with a boronic ester provides a stereospecific route to the geometric isomers ***Z*** **‐29** and ***E*** **‐29**.

**Figure 2:**
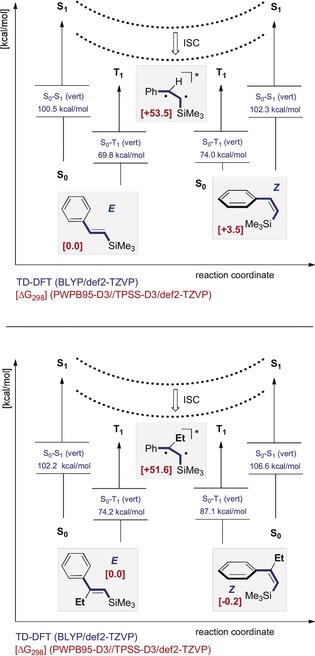
Energy profile for the photoisomerisations of ***E*** **‐2** to ***Z*** **‐2** (R=H, top) and ***E*** **‐1** to ***Z*** **‐1** (R=Et, bottom).

The generality of this isomerisation and the importance of the alkenyl silane core was a powerful impetus to interrogate the underlying origin of selectivity. Whilst allylic strain in the product is clearly essential in preventing re‐conjugation of the styrenyl chromophore, thus inhibiting re‐excitation, the ramifications on electronic structure required clarification. To that end, a computational study of the isomerisation of ***E*** **‐2**→***Z*** **‐2** (R=H) and ***E*** **‐1**→***Z*** **‐1** (R=Et) was conducted at the DFT level of theory (Figure 2, please see the Supporting Information).^21^

In the case of the unsubstituted system (R=H), for the net contra‐thermodynamic isomerisation process (***E*** **‐2**→***Z*** **‐2**, Δ*G*=+3.5 kcal mol^−1^), vertical (S_0_→S_1_) excitations of 100.5 and 102.3 kcal mol^−1^ were determined from the ground‐state *E*‐ and *Z*‐isomers, respectively (TD‐DFT, BLYP/def2‐TZVP). The analogous, spin‐forbidden (S_0_→T_1_) excitations were calculated to be 69.8 and 74.0 kcal mol^−1^ (Figure 2, top). In the case of the substituted systems ***E*** **‐** and ***Z*** **‐1**, the substituent combination rendered the transformation essentially thermoneutral (***E*** **‐1**→***Z*** **‐1**, Δ*G*=−0.2 kcal mol^−1^). However, the difference between the *E* and *Z* isomer is larger for the first singlet (S_0_→S_1_) excitation (102.2 versus 106.6 kcal mol^−1^) and the forbidden (S_0_→T_1_) excitation (74.2 versus 87.1 kcal mol^−1^).

**Figure 3:**
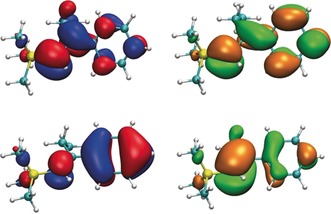
Frontier orbitals of the T_1_ state of ***E*** **‐1** at S_0_ geometry (PWPB95/def2‐TZVP). Top left: α‐HOMO; bottom left: α‐HOMO‐1; top right: β‐LUMO+1; bottom right: β‐LUMO.

In both ground (S_0_) states of ***E*** **‐1** and ***Z*** **‐1**, the HOMO and LUMO are constituted mainly by the π/π* orbitals of the ethene bond, with significant mixing of phenyl π‐orbitals (Figure 3). The (forbidden) first triplet excitation to the T_1_ of ***E*** **‐1** corresponds to a HOMO–LUMO excitation (see the Supporting Information). In this case, the two singly occupied orbitals of T_1_ are both delocalised over the styrenyl system and add up to localisation of spin density in the alkenyl group (Figure 5), promoting rotation around the alkenyl C−C bond to the energy minimum of the triplet state.

**Figure 4:**
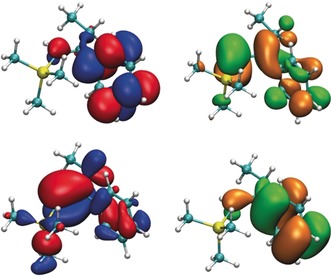
Frontier orbitals of the T_1_ state of ***Z*** **‐1** at S_0_ geometry (PWPB95/def2‐TZVP). Top left: α‐HOMO; bottom left: α‐HOMO‐1; top right: β‐LUMO+1; bottom right: β‐LUMO.

The more distorted styrenyl moiety of ***Z*** **‐1**, however, shows a significantly higher energy for the forbidden first vertical triplet excitation (87 kcal mol^−1^) in the TD‐DFT (B‐LYP) calculation and for the (relaxed) T_1_ wavefunction (100 kcal mol^−1^ with PWPB95‐D3) at the same geometry. The larger torsional angle (61.6°) aggravates the mixing of ethene and phenyl π‐orbitals and leads to two rather localised α‐spin orbitals in the T_1_ state (Figure 4). Consequently, spin density is largely accumulated in the phenyl ring.

**Figure 5:**
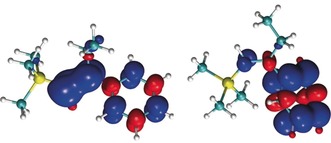
Comparison of the calculated spin densities of the triplet states for ***E*** **‐1** (left) and ***Z*** **‐1** (right) at the S_0_ geometry (R=Et). Spin density isosurface in blue: +0.005, red: −0.005 a.u.

A comparison of the calculated spin densities of ***E*** **‐1** and ***Z*** **‐1** (Figure 5) clearly illustrates this distinction, and thus it is conceivable that in the *E*‐scenario the (S_0_→T_1_) excitation leads to accumulation of spin density in the alkenyl group, facilitating rotation around that C−C bond. Whilst A^1,3^‐strain is ultimately responsible for the conformation, the reactivity differences between these energetically comparable isomers is a consequence of differences in the initial localisation of spin density. This may prove to be a valuable strategy to direct thermoneutral reactions at the structure/conformation level.^22^

## Conclusion

In conclusion, an operationally simple, geometric *E*→*Z* isomerisation of alkenyl silanes has been developed based on selective energy transfer catalysis at 365 nm using inexpensive benzophenone as the catalyst (1 kg=45 €). The transformation is efficient (2 h) and is compatible with a range of common Si groups, including (benzyl)silanes, silanols, and siletanes. Conveniently, the isomerisation can be added to pre‐existing *E*‐selective synthetic sequences, thereby enabling stereodivergence. The concept has been extended to include non‐symmetric bis‐nucleophilic systems (R=Si, Sn and B), thereby providing a general platform for the chemoselective, stereospecific generation of triarylethylenes from common precursors. This strategy of isomerising alkenyl bis‐nucleophiles containing metals and/or metalloids, prior to transmetallation, constitutes a formal solution to the challenge of *anti*‐metallometallation. Given the ubiquity of triarylethylenes in pharmaceutical design, it is envisaged that this “ethylene vector synthon” strategy will facilitate the exploration of new areas of 2D chemical space and find application in the stereocontrolled construction of well‐defined aromatic scaffolds. This is particularly true given the facile manner in which triarylethylenes can be converted into their tetrasubstituted analogues.^23^

A computational investigation to interrogate the origin of selectivity revealed that inducing A^1,3^‐strain causes significant differences in the localisation of spin density in energetically comparable isomers. The vertical (S_0_→T_1_) excitation in the *E*‐scenario leads to an accumulation of spin density in the alkene, thus enabling bond rotation. This finding further underscores the importance of understanding structure–function interplay^24^ in giving directionality to contra‐thermodynamic or thermoneutral processes.

## Conflict of interest

The authors declare no conflict of interest.

## Supporting information

As a service to our authors and readers, this journal provides supporting information supplied by the authors. Such materials are peer reviewed and may be re‐organized for online delivery, but are not copy‐edited or typeset. Technical support issues arising from supporting information (other than missing files) should be addressed to the authors.

Supplementary
