# Supplementary material for: Geometric E→Z Isomerisation of Alkenyl Silanes by Selective Energy Transfer Catalysis: Stereodivergent Synthesis of Triarylethylenes via a Formal anti‐Metallometallation
Source: Angew Chem Int Ed Engl. 2019 Oct 31;58(51):18619–26. doi: 10.1002/anie.201910169 (PMC6916377; doi:10.1002/anie.201910169)
Supplement: Supplementary file 1 — Supplementary [file ANIE-58-18619-s001.pdf]

Angewandte Chemie
Eine Zeitschrift der Gesellschaft Deutscher Chemiker

Supporting Information

Geometric $E \rightarrow Z$ Isomerisation of Alkenyl Silanes by Selective Energy Transfer Catalysis: Stereodivergent Synthesis of Triarylethylenes via a Formal *anti*-Metallometallation

*Svenja I. Faßbender[+], John J. Molloy[+], Christian Mück-Lichtenfeld, and Ryan Gilmour**

anie_201910169_sm_miscellaneous_information.pdf

1. General Information 1
2. Experimental Section 3
3. Reaction Progress Monitoring 35
4. DFT Calculations 37
5. NMR Spectra of Key Compounds 57
6. References 109

1. General Information

All chemicals were purchased as reagent grade and used without further purification. Solvents for purification (extraction and chromatography) were purchased as technical grade and distilled on the rotary evaporator prior to use. For column chromatography SiO_2-(40-63 µm for flash chromatography, VWR Chemicals) was used as stationary phase. Analytical thin layer chromatography (TLC) was performed on aluminum foil pre-coated with SiO_2-60 F_{254} (*Merck*) and visualised with a UV-lamp (254 nm) or CAM stain solution. Concentration *in vacuo* was performed at ~10 mbar and 40 °C, drying at ~10^{-2} mbar and rt. NMR spectra were measured by the NMR service of the Organisch-Chemisches Institut, Westfälische Wilhelms-Universität Münster on a *Bruker AV300, Bruker AV400, Agilent DD2 500* or *Agilent DD2 600* spectrometer at room temperature. The resonance multiplicity is abbreviated as: s (singlet), d (doublet), t (triplet), q (quadruplet), m (multiplet) and b (broad). Assignments of unknown compounds are based on DEPT, COSY (HH and FF), HMBC, HSQC and NOESY spectra. Melting points were measured on a *Büchi B-545* melting-point apparatus in open capillaries and are uncorrected. IR spectra were recorded on a *Perkin-Elmer 100 FT-IR* spectrometer, selected adsorption bands are reported in wavenumbers (cm^{-1}) and intensities are reported as: w (weak), m (medium), s (strong) and br (broad). Mass spectra were measured by the MS service of the Organisch-Chemisches Institut, Westfälische Wilhelms-Universität Münster. Isomerisation reactions were performed utilising a UVA LED (emission spectrum see Figure 1). Further isomerisation reactions were performed with a *Winger WEPRB3-S1 Power LED Star royalblue (450 nm) 3 W* (emission spectrum see Figure 2) and a set up of 4 *Winger WEPUV3-S2 UV Power LED Star (Schwarzlicht) 1.2 W* lamps (emission spectrum see Figure 3). The forward current per chip was set to 700 mA, the resulting forward voltage was 3.4 V while the resulting radiant flux was 3000 mW and 1200 mW for the 450 nm- and the 402 nm-lamp, respectively. The distance between the reaction vessels and the UV-lamp was set at approximately 0.5 cm.

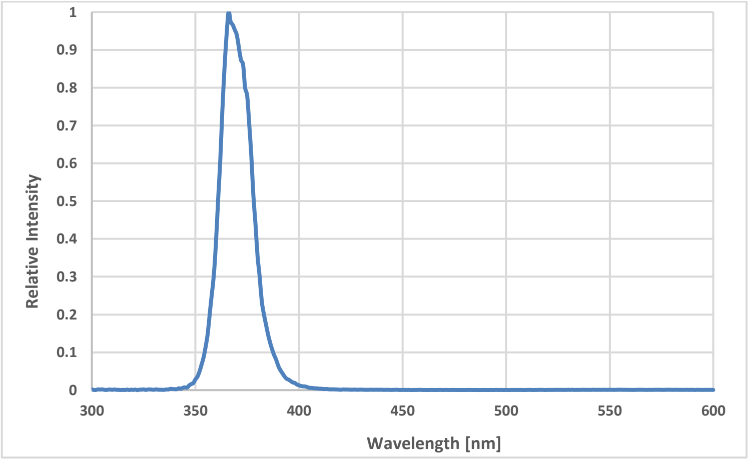

Figure S1: Emission spectrum of the utilised UVA-LED (365 nm).

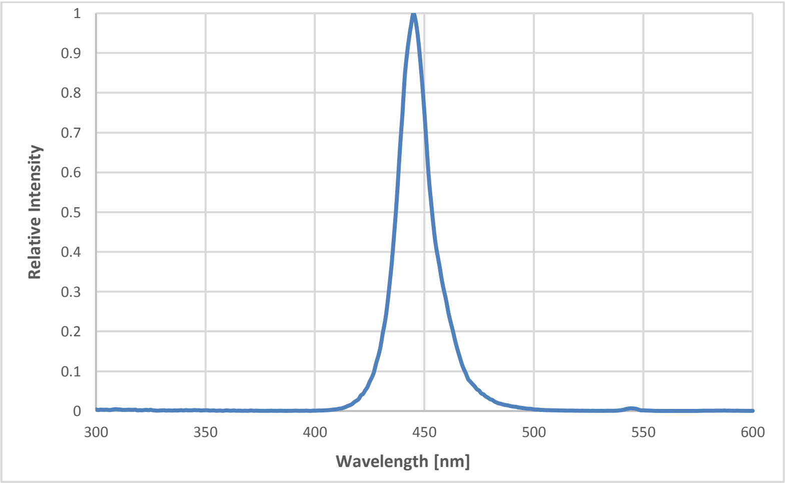

Figure S2: Emission spectrum of the utilised UV-lamp *Winger WEPRB3-S1 Power LED Star royalblue (450 nm) 3W – 35lm by Winger.*

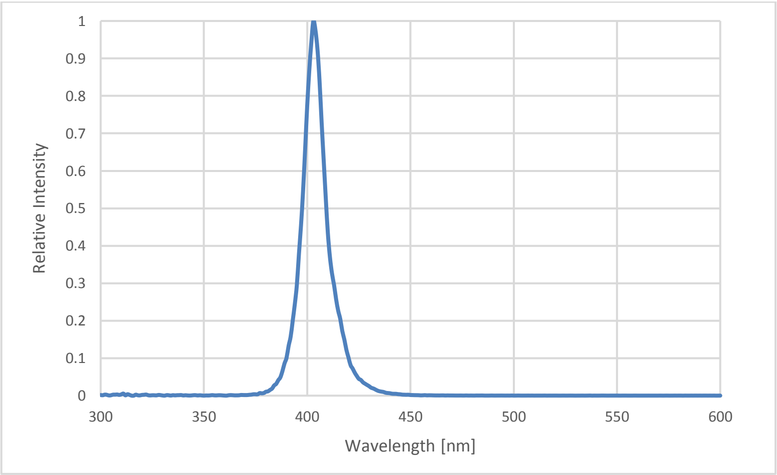

Figure S3: Emission spectrum of the utilised UV-lamp *Winger WEPUV3-S2 UV Power LED Star (Schwarzlicht) 1.2 W.*

2. Experimental Section

2.1. Preparation of starting materials

General Procedure A for the preparation of vinyl bromides from styrenes[1]
Bromine (1.2 eq.) was added dropwise to a solution of the specified styrene (1.0 eq.) in $CHCl_3$ at 0°C and the mixture was stirred for 1 h at room temperature. The reaction was quenched by the addition of $Na_2S_2O_3$-solution (aq., sat.), the organic phase was separated, dried over $MgSO_4$ and concentrated *in vacuo*. The residue was dissolved in ^{t}BuOH, potassium *tert*-butoxide (1.5 eq.) was added and the mixture refluxed for 1h. The reaction was allowed to come to room temperature, water was added, the organic phase was separated and the aqueous phase was extracted by Et_2O (3x). The combined organic phases were dried over $MgSO_4$, the crude product was concentrated *in vacuo* and purified by column chromatograohy (SiO_2, specified combination of solvents).

General Procedure B for the preparation of vinyl silanes from vinyl bromides
An oven-dried Schlenk-flask was charged with the specified vinyl bromine (1.0 eq.) in dry THF under argon atmosphere. *n*-Butyllithium (1.2 eq.) was added slowly at -78 °C and the mixture was stirred for 30 min before the specified chlorosilane (1.3 eq.) was added. The mixture was stirred overnight and allowed to gradually warm to room temperature. The reaction was quenched by the addition of water, the organic phase was separated and the aqueous phase was extracted with Et_2O (3x). The combined organic phases were dried over $MgSO_4$, the crude product was concentrated *in vacuo* and purified by column chromatography (SiO_2, specified combination of solvents).

General Procedure C for the preparation of vinyl silanes by Negishi Coupling[2]
The specified phenylacetylene (1.0 eq.) and $Pd(PPh_3)_4$ (0.02 eq.) were dissolved in dioxane in an oven-dried Schlenk-flask under argon atmosphere. Diethylzinc (1.0 M in hexane, 1.0 eq.) was added slowly at 0°C, followed by iodotrimethylsilane (2.0 eq.), and the mixture was stirred for 1 h at room temperature. The reaction was stopped by the addition of water, the mixture was filtered through a plug of celite, the organic phase

was separated and the aqueous phase was extracted with cyclohexane (3x). The combined organic phases were dried over $MgSO_4$, the crude product was concentrated in *vacuo* and purified by column chromatography (SiO_2, 100% CH) to yield the specified vinyl silanes.

General Procedure D for Si/Sn addition across phenylacetylenes

To an oven-dried Schlenk flask was added $Pd(PPh_3)_4$ (2 mol%). The flask was sealed and purged with argon before the addition of 1,4-dioxane (0.5 M), alkyne (1.0 eq.), and trimethyl(tributylstannyl)silane (1.15 eq.). The reaction was heated to 100 °C with stirring for 1 h. The crude residue was concentrated under reduced pressure and purified by column chromatography (SiO_2, specified combination of solvents).

(*E*)-Trimethyl(2-phenylbut-1-en-1-yl)silane (*E*-1)

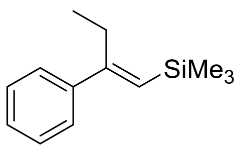

According to General Procedure C, phenylacetylene (0.27 mL, 2.5 mmol, 1.0 eq.) was converted to ***E*-1** yielding a colorless oil (305 mg, 60%); analytical data in agreement with the literature.[3]

^{1}H NMR (300 MHz, $CDCl_3$) δ = 7.41 – 7.31 (m, 2H), 7.31 – 7.14 (m, 3H), 5.68 (s, 1H), 2.59 (q, *J* = 7.5 Hz, 2H), 0.93 (t, *J* = 7.5 Hz, 3H), 0.14 (s, 9H) ppm.

Trimethyl(phenylethynyl)silane (30)[4]

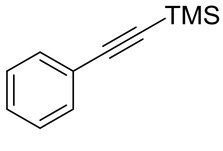

Phenylacetylene (0.55 mL, 5.0 mmol, 1.0 eq.) and dry THF (10 mL) were added to an oven-dried Schlenk flask under argon atmosphere and cooled to -78°C, before *n*-butyllithium (1.6 M in hexane, 3.40 mL, 5.5 mmol, 1.1 eq.) was added dropwise. The reaction mixture was stirred for 30 min at -78°C. Chlorotrimethylsilane (0.76 mL, 6.0 mmol, 1.2 eq.) was added and the mixture stirred for another 30 min at -78°C before being stirred at room temperature for 1.5 h. The reaction was quenched by the addition of NH_4Cl solution (aq., sat.), the organic phase was separated, the aqueous phase was extracted with Et_2O (3x 10 mL), the combined organic phases were washed with brine and dried over $MgSO_4$. Evaporation of the solvent *in vacuo* yielded **30** as a colorless oil (783 mg, 90%); analytical data in agreement with the literature.[4]

^{1}**H NMR** (200 MHz, $CDCl_3$) δ = 7.46 – 7.32 (m, 2H), 7.29 – 7.15 (m, 3H), 0.20 (s, 9H) ppm.

(*E*)-Trimethyl(styryl)silane (*E*-2)[4]

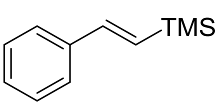

An oven-dried Schlenk-flask was charged with **30** (550 mg, 3.16 mmol, 1.0 eq.) and pentane (10 mL) under argon atmosphere and cooled to 0°C. DIBAL-H (1.0 M in toluene, 3.79 mL, 3.79 mmol, 1.2 eq.) was added slowly, the reaction was stirred at room temperature overnight, quenched with ice-cooled H_2SO_4 (5%, aq.) at 0°C and filtered over a celite plug. The organic phase was separated, the aqueous phase was extracted with Et_2O (3x 10 mL), the combined organic phases were dried over $MgSO_4$ and the crude product concentrated *in vacuo*. Purification by column chromatography (SiO_2, CH) yielded ***E*-2** as a colorless oil (426 mg, 76%); analytical data in agreement with the literature.[4]

^{1}**H NMR** (300 MHz, $CDCl_3$) δ = 7.48 – 7.41 (m, 2H), 7.39 – 7.18 (m, 3H), 6.88 (d, *J* = 19.1 Hz, 1H), 6.49 (dd, *J* = 19.1, 0.7 Hz, 1H), 0.16 (s, 9H) ppm.

***tert*-Butyldimethyl(phenylethynyl)silane (31)**

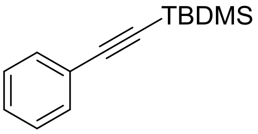

Phenylacetylene (0.55 mL, 5.0 mmol, 1.0 eq.) and dry THF (10 mL) were added, via syringe, to an oven-dried round-bottom flask under an argon atmosphere. The flask was cooled to -78 °C before the dropwise addition of *n*-butyllithium (1.6 M in hexane, 3.40 mL, 5.5 mmol, 1.1 eq.) and the mixture was stirred for 30 min. *tert*-Butyldimethylsilyl chloride (901.2 mg, 6.0 mmol, 1.2 eq.) as a solution in THF (1mL) was added dropwise and the reaction mixture was stirred for 30 min at -78 °C. The reaction mixture was allowed to warm to room temperature and was stirred for 1.5 h. The reaction was quenched by the addition of aq. sat. NH_4Cl solution (30 mL) and organics were separated. The aqueous phase was extracted with Et_2O (3x 10 mL) and the combined organic phases were washed with brine (50 mL), dried over $MgSO_4$, and concentrated under reduced pressure. The crude residue was purified by column chromatography (SiO_2, 100% *n*-pentane) to afford the desired product as a clear oil (768 mg, 71%). Analytical data was in agreement with the literature.[5]

^{1}H NMR (300 MHz, $CDCl_3$) δ = 7.52 – 7.43 (m, 2H), 7.34 – 7.24 (m, 3H), 1.01 (s, 9H), 0.19 (s, 6H) ppm.

(*E*)-*tert*-Butyldimethyl(styryl)silane (*E-3*)

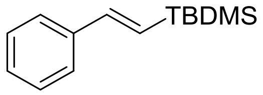

DIBAL-H (1.0 M in toluene, 3.79 mL, 3.79 mmol, 1.2 eq.) was added dropwise to a solution of **31** (550 mg, 3.16 mmol, 1.0 eq.) in pentane (10 mL) at 0°C under an argon atmosphere. The reaction mixture was stirred at room temperature overnight. The reaction was quenched with cold H_2SO_4 (5%, aq.) at 0°C and filtered over celite. The reaction was diluted in Et_2O (25 mL) and organics separated. The aqueous phase was extracted with Et_2O (3x 10 mL). The combined organic phases were washed with brine (50 mL), dried over $MgSO_4$, and concentrated under reduced pressure. The crude residue was purified by column chromatography (SiO_2, 100% *n*-pentane) to afford the desired product as a colorless oil (605 mg, 88%). Analytical data was in agreement with the literature.[6]

^{1}H NMR (400 MHz, $CDCl_3$) δ = 7.41 – 7.33 (m, 2H), 7.26 (td, *J* = 8.0, 7.6, 1.8 Hz, 2H), 7.21 – 7.14 (m, 1H), 6.82 (d, *J* = 19.2 Hz, 1H), 6.41 (d, *J* = 19.2 Hz, 1H), 0.85 (s, 9H), 0.05 (s, 6H) ppm.

Triisopropyl(phenylethynyl)silane (32)

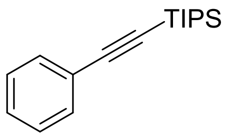

Phenylacetylene (0.55 mL, 5.0 mmol, 1.0 eq.) and dry THF (10 mL) were added, via syringe, to an oven-dried round-bottom flask under an argon atmosphere. The flask was cooled to -78 °C before the dropwise addition of *n*-Butyllithium (1.6 M in hexane, 3.40 mL, 5.5 mmol, 1.1 eq.) and the mixture was stirred for 30 min. Triisopropylsilyl chloride (1.28 mL, 6.0 mmol, 1.2 eq.) was added dropwise and the reaction mixture was stirred for 30 min at -78 °C. The reaction mixture was allowed to warm to room temperature and was stirred for 1.5 h. The reaction was quenched by the addition of aq. sat. NH_4Cl solution (30 mL) and organics were separated. The aqueous phase was extracted with Et_2O (3x 10 mL) and the combined organic phases were washed with brine (50 mL), dried over $MgSO_4$, and concentrated under reduced pressure. The crude residue was purified by column chromatography (SiO_2, 100% *n*-pentane) to afford the desired product as a clear oil (1.16 g, 90%). Analytical data was in agreement with the literature.[6]

^{1}**H NMR** (400 MHz, $CDCl_3$): δ = 7.44 – 7.36 (m, 2H), 7.26 – 7.19 (m, 3H), 1.08 – 1.02 (m, 21H) ppm.

(*Z*)-Triisopropyl(styryl)silane (*Z*-4)

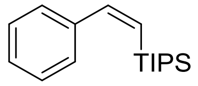

DIBAL-H (1.0 M in toluene, 3.79 mL, 3,79 mmol, 1.2 eq.) was added dropwise to a solution of **32** (817 mg, 3.16 mmol, 1.0 eq.) in pentane (10 mL) at 0°C under an argon atmosphere. The reaction mixture was stirred at room temperature overnight. The reaction was quenched with cold H_2SO_4 (5%, aq.) at 0°C and filtered over celite. The reaction was diluted in Et_2O (25 mL) and organics separated. The aqueous phase was extracted with Et_2O (3x 10 mL). The combined organic phases were washed with brine (50 mL), dried over $MgSO_4$, and concentrated under reduced pressure. The crude residue was purified by column chromatography (SiO_2, 100% *n*-pentane) to afford the desired product as a colorless oil (200 mg, 24%). Analytical data was in agreement with the literature.[6]

^{1}**H NMR** (300 MHz, $CDCl_3$): δ = 7.50 (d, *J* = 15.6 Hz, 1H), 7.32 – 7.20 (m, 5H), 5.72 (d, *J* = 15.7 Hz, 1H), 1.17 – 0.85 (m, 21H) ppm.

(*E*)-(1-Bromoprop-1-en-2-yl)benzene (33)

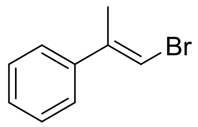

According to General Procedure A, *α*-methylstyrene (0.90 mL, 7.0 mmol, 1.0 eq.) was converted to **33**. Purification by column chromatography (SiO_2, 100% CH) yielded a colorless oil (680 mg, 49%); analytical data in agreement with the literature.[1]

^{1}**H NMR** (400 MHz, $CDCl_3$) δ = 7.40 – 7.27 (m, 5H), 6.46 (q, *J* = 1.3 Hz, 1H), 2.24 (d, *J* = 1.3 Hz, 3H) ppm.

(*E*)-Trimethyl(2-phenylprop-1-en-1-yl)silane (*E*-5)

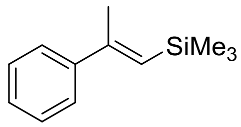

According to General Procedure B, **33** (190.3 mg, 1.0 mmol, 1.0 eq) and trimethylchlorosilane were converted to ***E*-5** to yield a colorless oil (72 mg, 38%) after purified by column chromatography (SiO_2, 100% CH); analytical data in agreement with the literature.[3]

^{1}**H NMR** (400 MHz, CD_2Cl_2) δ = 7.50 – 7.41 (m, 2H), 7.38 – 7.19 (m, 3H), 5.93 (q, *J* = 0.9 Hz, 1H), 2.21 (d, *J* = 0.8 Hz, 3H), 0.20 (s, 9H) ppm.

(*E*)-(2-(4-Fluorophenyl)but-1-en-1-yl)trimethylsilane (*E*-6)

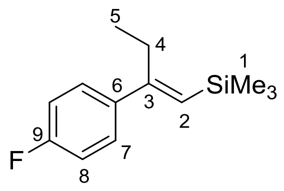

According to General Procedure C, 1-ethynyl-4-fluorobenzene (0.23 mL, 2.0 mmol, 1.0 eq.) was converted to ***E*-6** yielding a colorless oil (371 mg, 83%).

R$_f$ = 0.65 (CH); **HR-APCI-MS**: m/z: 329.02861 ($[M+Ag]^+$, calcd. for $C_{13}H_{19}FSiAg^+$: 329.02855); ^{1}**H NMR** (500 MHz, CD_2Cl_2) δ = 7.42 – 7.34 (m, 2H; H7), 7.04 – 6.96 (m, 2H; H8), 5.70 (s, 1H; H2), 2.63 (q, *J* = 7.5 Hz, 2H; H4), 0.96 (t, *J* = 7.5 Hz, 3H; H5), 0.19 (s, 9H; H1) ppm; ^{13}**C NMR** (126 MHz, CD_2Cl_2) δ = 162.7 (d, *J* = 245.2 Hz, C9), 158.5 (C3), 140.1 (d, J_{CF} = 3.2 Hz, C6), 128.4 (d, J_{CF} = 7.9 Hz, 2C, C7), 127.8 (d, J_{CF} = 1.2 Hz, C2), 115.3 (d, J_{CF} = 21.2 Hz, 2C, C8), 28.5 (C4), 14.39 (C5), 0.4 (3C, C1) ppm; ^{19}**F NMR** (470 MHz, CD_2Cl_2) δ = -116.59 ppm; **IR (ATR)**: ῦ = 2959(w), 1601(m), 1506(m), 1465(w), 1247(m), 1232(m), 1158 (m), 922(w), 858(s), 831(s), 767(m), 749(m), 731(m), 712(m), 689(m) cm^{-1}.

(*E*)-(2-(4-Bromophenyl)but-1-en-1-yl)trimethylsilane (*E*-7)

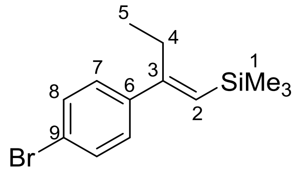

According to General Procedure C, 4-bromophenylacetylene (362.1 mg, 2.0 mmol, 1.0 eq.) was converted to ***E*-7** yielding a colorless oil (248 mg, 44%).

R$_f$ = 0.75 (CH); **GC-EI-MS**: m/z: 284.04120 ($[M]^+$, calcd. for $C_{13}H_{19}BrSi^+$: 282.04143); ^{1}**H NMR** (500 MHz, CD_2Cl_2) δ = 7.47 – 7.41 (m, 2H; H8), 7.32 – 7.25 (m, 2H; H7), 5.75 (s, 1H; H2), 2.62 (q, *J* = 7.5 Hz, 2H; H4), 0.96 (t, *J* = 7.5 Hz, 3H; H5), 0.19 (s, 9H; H1) ppm; ^{13}**C NMR** (126 MHz, CD_2Cl_2) δ = 158.3 (C3), 143.0 (C6), 131.7 (2C, C8), 128.7 (C2), 128.6 (2C, C7), 121.5 (C9), 28.3 (C2), 14.4 (C5), 0.4 (3C, C1) ppm; **IR (ATR)**: ῦ = 2956(w), 2824(w), 1593(w), 1557(w), 1486(m), 1464(w), 1393(w), 1374 (w), 1259(w), 1246(m), 1072(m), 1007(m), 944(w), 922(m), 856(s), 812(s), 776(m), 718(w), 689(m) cm^{-1}.

(*E*)-Trimethyl(2-(p-tolyl)but-1-en-1-yl)silane (*E*-8)

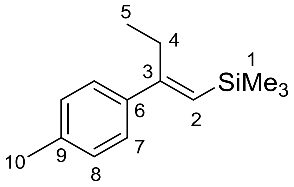

According to General Procedure C, 4-ethynyltoluene (0.63 mL, 5.0 mmol, 1.0 eq.) was converted to ***E*-8** yielding a colorless oil (434 mg, 40%).

R R_f = 0.65 (CH); **HR-APCI-MS**: m/z: 325.05361 ([M+Ag]$^+$, calcd. for $C_{14}H_{22}SiAg^+$: 325.05362); **^{1}H NMR** (500 MHz, CD_2Cl_2) δ = 7.33 – 7.26 (m, 2H; H7), 7.13 (dq, *J* = 7.9, 0.6 Hz, 2H; H8), 5.73 (d, *J* = 0.7 Hz, 1H; H2), 2.64 (qd, *J* = 7.5, 0.6 Hz, 2H; H4), 2.34 (s, 3H; H10), 0.97 (td, *J* = 7.5, 0.7 Hz, 3H; H5), 0.20 (d, *J* = 0.7 Hz, 9H; H1) ppm; **^{13}C NMR** (126 MHz, CD_2Cl_2) δ = 159.5 (C3), 140.9 (C6), 137.6 (C9), 129.4 (2C, C8), 126.6 (C2), 126.5 (2C, C7), 28.3 (C4), 21.3 (C10), 14.6 (C5), 0.5 (3C, C1) ppm; **IR (ATR):** ῦ = 2956(w), 1596(w), 1509(w), 1247(m), 922(m), 855(s), 807(s), 766(m), 748(m), 727(m), 688(m) cm^{-1}.

(*E*)-(2-(4-Methoxyphenyl)but-1-en-1-yl)trimethylsilane (*E*-9)

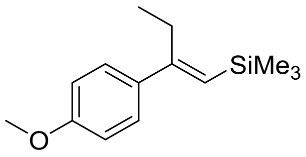

According to General Procedure C, 4-ethynylanisole (0.26 mL, 2.0 mmol, 1.0 eq.) was converted to ***E*-9** yielding a colorless oil (205 mg, 44%); analytical data in agreement with the literature.[2]

^{1}H NMR (400 MHz, $CDCl_3$) δ = 7.40 – 7.32 (m, 2H), 6.90 – 6.80 (m, 2H), 5.68 (s, 1H), 3.81 (s, 3H), 2.61 (q, *J* = 7.5 Hz, 2H), 0.99 (t, *J* = 7.5 Hz, 3H), 0.18 (s, 9H) ppm.

(*E*)-Trimethyl(2-(m-tolyl)but-1-en-1-yl)silane (*E*-10)

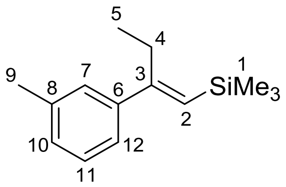

According to General Procedure C, 3-ethynyltoluene (0.25 mL, 1.9 mmol, 1.0 eq.) was converted to ***E*-10** yielding a colorless oil (367 mg, 88%).

R_f = 0.67 (CH); **HR-APCI-MS**: m/z: 325.05350 ([M+Ag]$^+$, calcd. for $C_{14}H_{22}SiAg^+$: 325.05362); **^{1}H NMR** (500 MHz, CD_2Cl_2) δ = 7.25 – 7.15 (m, 3H; C7, C10, C11), 7.11 – 7.03 (m, 1H; H12), 5.72 (s, 1H; H2), 2.64 (q, *J* = 7.5 Hz, 2H; H4), 2.35 (s, 3H; H9), 0.97 (t, *J* = 7.5 Hz, 3H; H5), 0.20 (s, 9H; H1) ppm; **^{13}C NMR** (126 MHz, CD_2Cl_2) δ =159.9 (C3), 144.0 (C6), 138.3 (C8), 128.6 (C10/C11), 128.5 (C12), 127.6 (C7), 127.4 (C2), 123.8 (C10/C11), 28.4 (C4), 21.8 (C9), 14.5 (C5), 0.5 (C1)

ppm; **IR (ATR):** ῦ = 2956(w), 1594(w), 1577(w), 1464(w), 1246(m), 867(s), 834(s), 782(s), 765(m), 723(m); 688(m) cm^{-1}.

Trimethyl(*o*-tolylethynyl)silane (*E*-11)

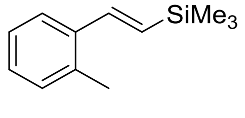

An oven-dried Schlenk flask was charged with 2-ethynyltoluene (0.63 mL, 5.0 mmol, 1.0 eq.) in dry THF (10 mL) under argon atmosphere. *n*-Butyllithium (2.5 M in hexane, 2.2 mL, 5.5 mmol, 1.1 eq.) was added slowly at -78°C and the reaction mixture was stirred for 30 min at room termperature. Chlorotrimethylsilane (0.76 mL, 6.0 mmol, 1.2 eq.) was added and the mixture stirred for another 30 min at -78°C before being stirred at room temperature for 1.5 h. The reaction was quenched by the addition of NH_4Cl solution (aq., sat.), the organic phase was separated, the aqueous phase was extracted with Et_2O (3x 10 mL), the combined organic phases were washed with brine, dried over $MgSO_4$ and concentrated *in vacuo*. The residue was dissolved in pentane (15 mL) and cooled to 0°C, before DIBAL-H (1.0 M in toluene, 6.0 mL, 6.0 mmol, 1.2 eq.) was added slowly. The reaction was stirred at room temperature overnight, quenched with ice-cooled H_2SO_4 (5%, aq.) at 0°C and filtered over a celite plug. The organic phase was separated, the aqueous phase was extracted with Et_2O (3x 10 mL), the combined organic phases were dried over $MgSO_4$ and the crude product concentrated *in vacuo*. Purification by column chromatography (SiO_2, 100% CH) yielded ***E*-11** as a colorless oil (556 mg, 59%); analytical data in agreement with the literature.[7]

^{1}H NMR (400 MHz, $CDCl_3$) δ = 7.58 – 7.47 (m, 1H), 7.21 – 7.07 (m, 4H), 6.39 (d, *J* = 19.0 Hz, 1H), 2.38 (s, 3H), 0.17 (s, 9H) ppm.

(*E*)-(4-(1-(Trimethylsilyl)but-1-en-2-yl)phenyl)boronic acid, pinacol ester (*E*-12)

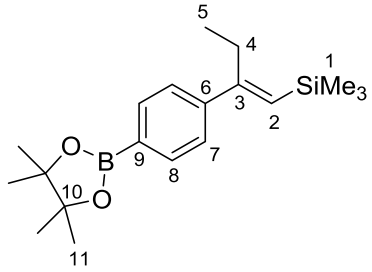

According to General Procedure C, 4-ethynylphenylboronic acid, pinacol ester (231 mg, 0.7 mmol, 1.0 eq.) was converted to ***E*-12** yielding a white solid (172 mg, 74%) after purification by column chromatography (EtOAc/CH 1:30).

R_f = 0.81 (EtOAc/CH 1:3); **M.p.:** 61.3-64.5 °C; **HR-ESI-MS:** m/z: 353.2079 ($[M+Na]^+$, calcd. for $C_{19}H_{31}O_2BSiNa^+$: 353.2082); **^{1}H NMR** (600 MHz, CD_2Cl_2) δ = 7.72 – 7.68 (m,

2H; H8), 7.40 (d, *J* = 8.3 Hz, 1H; H7), 5.80 (s, 1H; H2), 2.65 (q, *J* = 7.5 Hz, 2H; H4), 1.33 (s, 12H; H11), 0.95 (t, *J* = 7.5 Hz, 3H; H5), 0.20 (s, 9H; H1); **^{13}C NMR** (151 MHz, CD_2Cl_2) δ = 159.5 (C3), 146.6 (C6), 135.1 (2C, C8), 128.6 (C2), 126.1 (2C, C7), 84.3 (C10), 28.3 (C4), 25.3 (4C, C11), 14.4 (C5), 0.4 (3C,C1) ppm (C9 missing due to quadrupolar relaxation); **^{11}B NMR** (192 MHz, CD_2Cl_2) δ = 30.88 ppm; **IR (ATR):** $\tilde{\nu}$ = 2960(w), 1607(m), 1593(w), 1462(w), 1397(m), 1359(s), 1323(m), 1260(m), 248(m), 1142(s), 1097(m), 1083(m), 1015(m), 963(m), 926(m), 857(s), 837(s), 751(m), 693(m), 673(m), 659(s) cm^{-1}.

(*Z*)-(1-Phenylethene-1,2-diyl)bis(trimethylsilane) (*Z*-13)

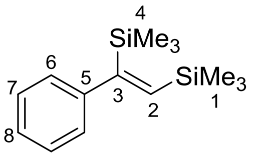

***Z*-22** (562 mg, 1.21 mmol, 1.0 eq.) and dry THF (10 mL) were added to an oven-dried Schlenk flask under argon and cooled to -78°C. *n*-Butyllithium (2.5 M in hexane, 0.50 mL, 1.33 mmol, 1.1 eq.) was added slowly and the mixture was stirred for 30 min before trimethylchlorosilane (0.18 mL, 1.45 mmol, 1.2 eq.) was added. The reaction was stirred overnight while being allowed to gradually warm to rt. The reaction was quenched by the addition of water, the organic phase was separated, the aqueous phase was extracted with Et_2O (3x), dried over $MgSO_4$ and concentrated i*n vacuo.* Purification by column chromatography (SiO_2, 100% CH) yielded ***Z*-13** as a colorless oil (54 mg, 18%).

R_f = 0.77 (CH); **GC-EI-MS**: m/z: 248.14119 ($[M]^+$, calcd. for $C_{14}H_{24}Si_2^+$: 248.14111); **^{1}H NMR** (500 MHz, CD_2Cl_2) δ = 7.28 – 7.22 (m, 2H; H7), 7.18 – 7.12 (m, 1H; H8), 7.05 – 6.99 (m, 2H; H6), 6.42 (s, 1H; H2), 0.22 (s, 9H; H1), 0.16 (s, 9H; H4) ppm; **^{13}C NMR** (126 MHz, CD_2Cl_2) δ = 164.8 (C3), 151.7 (C5), 149.5 (C2), 128.3 (2C, C7), 126.8 (2C, C6), 126.0 (C8), 1.4 (3C, C4), 1.2 (3C, C1) ppm; **IR (ATR):** $\tilde{\nu}$ = 2954(w), 2896(w), 1596(w), 1485(w), 1440(w), 1405(w), 1247(m), 1202(w), 1071(w), 1030(w), 921(w), 863(s), 829(s), 782(m), 755(m), 719(w), 697(s) cm^{-1}.

But-1-en-2-ylbenzene (34)

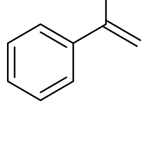

An oven-dried Schlenk-flask was charged with methyltriphenylphosphonium bromide (17.86 g, 50 mmol, 1.0 eq.) in dry THF (100 mL) under argon atmosphere. *n*-Butyllithium (1.6 M in hexanes,

31.25 mL, 50 mmol, 1.0 eq.) was added at 0°C and the mixture was stirred for 1 h at 0°C before propiophenone (6.65 mL, 50 mmol, 1.0 eq.) was added and then stirred for 16 h while being allowed to warm up to room temperature. The reaction was quenched by the addition of water (50 mL), the organic phase was separated and the aqueous phase was extracted with Et_2O (3x 50 mL). The combined organic phases were dried over $MgSO_4$, concentrated *in vacuo* and the crude product was purified by column chromatography (SiO_2, CH) to yield **34** as a colorless oil (5.03 g, 76%); analytical data in agreement with the literature.[1]

^{1}H NMR (300 MHz, $CDCl_3$) δ = 7.46 – 7.38 (m, 2H), 7.38 – 7.24 (m, 3H), 5.31 – 5.25 (m, 1H), 5.07 (dd, *J* = 2.8, 1.3 Hz, 1H), 2.78 – 2.21 (m, 2H), 1.11 (t, *J* = 7.4 Hz, 3H) ppm.

(*E*)-(1-Bromobut-1-en-2-yl)benzene (35)

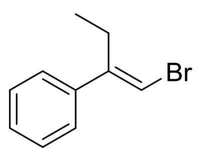

According to General Procedure A, **34** (4.32 g, 32 mmol, 1.0 eq.) was converted to **35** yielding a colorless oil (3.10 g, 46%); analytical data in agreement with the literature.[1]

^{1}H NMR (300 MHz, $CDCl_3$) δ = 7.40 – 7.28 (m, 5H), 6.32 (s, 1H), 2.70 (q, *J* = 7.5 Hz, 2H), 1.03 (t, *J* = 7.5 Hz, 3H) ppm.

(*E*)-Benzyldimethyl(2-phenylbut-1-en-1-yl)silane (*E*-14)

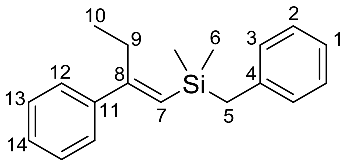

According to General Procedure B, **35** (920 mg, 4.36 mmol, 1.0 eq.) and benzyldimethylchlorosilane (1.03 mL, 5.67 mmol, 1.3 eq.) were converted to ***E*-14** yielding a colorless oil (454 mg, 37%).

R_f = 0.36 (CH); **GC-EI-MS**: m/z: 189.10931 ([M-Bn]$^+$, calcd. for $C_{12}H_{17}Si^+$: 189.10940); **^{1}H NMR** (500 MHz,CD_2Cl_2) δ = 7.41 – 7.36 (m, 2H; H12), 7.34 – 7.30 (m, 2H; H13), 7.28 – 7.24 (m, 1H; H14), 7.24 – 7.19 (m, 2H; H2), 7.10 – 7.05 (m, 3H; H1; H3), 5.68 (s, 1H; H7), 2.59 (q, *J* = 7.5 Hz, 2H; H9), 2.26 (s, 2H; H5), 0.94 (t, *J* = 7.5 Hz, 3H; H10), 0.18 (d, *J* = 0.6 Hz, 6H; H6) ppm; **^{13}C NMR** (126 MHz, CD_2Cl_2) δ = 160.7 (C8), 144.0 (C11), 140.9 (C4), 128.9 (2C, C3), 128.7 (2C, C13), 128.6 (2C, C2), 127.8 (C14), 126.8 (2C, C12), 125.7 (C7), 124.5 (C1), 28.7 (C9), 27.2 (C5), 14.4 (C10), -1.4 (2C, C6) ppm;

IR (ATR): ῦ = 3058(w), 3023(w), 2962(w), 1594(w), 1570(w), 1492(m), 1451(w), 1247(m), 1206(w), 1153(w), 1056(w), 928(w), 904(w), 826(s), 790(m), 759(s), 694(s) cm^{-1}.

(*E*)-1-Methyl-1-(2-phenylbut-1-en-1-yl)siletane (*E*-15)[8]

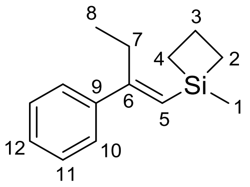

A Schlenk tube was charged with magnesium turnings (285 mg, 10.0 mmol, 2.5 eq.) and flame-dried before dry THF (10 mL) was added under argon atmosphere. The tube was placed into the sonication bath for 15 min. After addition of a grain of iodine, 1 mL of a solution of **35** (990 mg, 4.69 mmol, 1.0 eq.) in THF (5 mL) was added, the mixture was put into the sonication bath for another 10 min and then refluxed until the Grignard-reaction was initiated. The rest of the solution was added slowly over 15 min, followed by 1-chloro-1-methylsilacyclobutane (0.75 mL, 6.10 mmol, 1.3 eq.), and the mixture was refluxed for 2 h. The reaction was allowed to come to room temperature before 1 M HCl (10 mL) was added at 0°C. The organic phase was separated and the aqueous phase was extracted with cyclohexane (3x10 mL). The combined organic phases were washed with $Na_2S_2O_3$-solution (aq., sat.), dried over $MgSO_4$ and concentrated *in vacuo.* Purification by column chromatography (SiO_2, 100% CH) yielded a colorless oil (576 mg, 57%).

R_f = 0.66 (CH); **HR-APCI-MS**: m/z: 215.12459 ($[M-H]^-$, calcd. for $C_{14}H_{19}Si^-$: 215.12505); **^{1}H NMR** (500 MHz, CD_2Cl_2) δ = 7.45 – 7.42 (m, 2H; H10), 7.36 – 7.31 (m, 2H; H11), 7.30 – 7.25 (m, 1H; H12), 5.91 (s, 1H; H5), 2.69 (q, *J* = 7.5 Hz, 2H; H7), 2.23 – 2.05 (m, 2H; H3), 1.27 – 1.18 (m, 2H; H2/H4), 1.16 – 1.07 (m, 2H; H2/H4), 1.01 (t, *J* = 7.5 Hz, 3H; H8), 0.45 (s, 3H; H1) ppm; **^{13}C NMR** (126 MHz, CD_2Cl_2) δ = 160.4 (C6), 143.4 (C9), 128.8 (2C, C11), 128.0 (C12), 126.8 (2C, C10), 126.3 (C5), 28.7 (C7), 18.9 (C3), 15.9 (2C, H2, C4), 14.5 (C8), 0.0 (C1) ppm; **IR (ATR):** ῦ = 3058(w), 2964(w), 2929(w), 2872(w), 1592(w), 1570(w), 1493(w), 1463(w), 1442(w), 1395(w), 1374(w), 1247(w), 1119(m), 1075(w), 1031(w), 1015(w), 927(m), 900(m), 865(s), 843(m), 765(s), 748(s), 715(m), 692(s) cm^{-1}.

(*E*)-Dimethyl(2-phenylbut-1-en-1-yl)silanol (*E*-20)

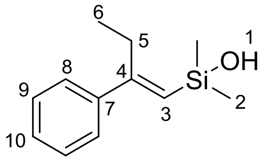

A Schlenk tube was charged with magneisum turnings (304 mg, 12.5 mmol, 2.5 eq.) and flame-dried before dry THF (15 mL) was added under argon atmosphere. The tube was placed into the sonication bath for 15 min. After addition of a grain of iodine, 1 mL of a solution of vinyl bromide **35** (1055 mg, 5 mmol, 1.0 eq.) in dry THF (5 mL) was added dropwise, the mixture was put into the sonication bath for another 10 min and then refluxed until the Grignard-reaction was initiated. The rest of the vinyl bromide solution was added slowly over 15 min, followed by chlorodimethylsilane (0.72 mL, 6.50 mmol, 1.3 eq.), and the mixture was refluxed for overnight. The reaction was allowed to come to room temperature before 1 M HCl (10 mL) was added at 0°C. The organic phase was separated and the aqueous phase was extracted with cyclohexane (3x10 mL). The combined organic phases were washed with $Na_2S_2O_3$-solution (aq., sat.), dried over $MgSO_4$ and concentrated *in vacuo.* The crude vinylsilane was purified by column chromatography (SiO_2, 100% CH) and dissolved in MeCN (4 mL), before $[Ir(1,5\text{-cod})Cl]_2$ (12 mg, 0.018 mmol, 0.01 eq.) and H_2O (64 µL, 3.6 mmol, 2.0 eq.) were added. The mixture was stirred at rt for 1 h, the solvent was evaporated under reduced pressure and the crude vinyl silanol was purified by column chromatography (EtOAc/ CH 1:20) to yield a colorless oil (420 mg, 41%).

R_f = 0.18 (EtOAc/ CH 1:8); **GC-EI-MS**: m/z: 191.08849 ($[M\text{-}CH_3]^+$, calcd. for $C_{11}H_{15}OSi^+$: 191.08867); **^{1}H NMR** (600 MHz, CD_2Cl_2) δ = 7.43 – 7.39 (m, 2H, H8), 7.32 (td, *J* = 7.0, 1.2 Hz, 2H, H9), 7.28 – 7.25 (m, 1H, H10), 5.72 (s, 1H, H3), 2.73 (q, *J* = 7.5 Hz, 2H, H5), 0.99 (t, *J* = 7.5 Hz, 3H, H6), 0.30 (s, 6H, H2) ppm; **^{13}C NMR** (151 MHz, CD_2Cl_2) δ = 161.1 (C4), 143.6 (C7), 128.7 (2C, C9), 128.0 (C10), 126.8 (2C, C8), 126.5 (C3), 28.5 (C5), 14.5 (C6), 1.9 (2C, C2) ppm; **IR (ATR):** $\tilde{\upsilon}$ = 3267(b), 2962(w), 2926(w), 2851(w), 1595(w), 1571(w), 1493(w), 1450(w), 1250(m), 1075(w), 1015(w), 929(w), 834(s), 802(m), 764(s), 693(s) cm^{-1}.

(*Z*)-Trimethyl(2-phenyl-2-(tributylstannyl)vinyl)silane (*Z*-22)[9]

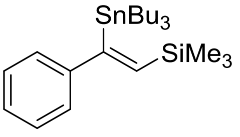

Prepared according to General Procedure D, phenylacetylene (0.55 mL, 5.0 mmol, 1.0 eq.) was converted to **Z-22** in 1 h yielding a colourless oil (2209 mg, 95%) after purification by column chromatography (100% *n*-pentane). Analytical data in agreement with the literature.[10]

^{1}H NMR (300 MHz, $CDCl_3$) δ = 7.43 – 7.36 (m, 2H), 7.27 (td, *J* = 7.2, 1.4 Hz, 1H), 7.12 (dt, *J* = 7.8, 1.5 Hz, 2H), 6.70 (s, 1H), 1.64 – 1.48 (m, 6H), 1.47 – 1.33 (m, 6H), 1.10 – 1.01 (m, 6H), 0.99 (t, *J* = 7.3 Hz, 9H), 0.32 (s, 9H).

(*E*)-(1-Phenyl-2-(trimethylsilyl)vinyl)boronic acid, pinacol ester (*E*-23)[11]

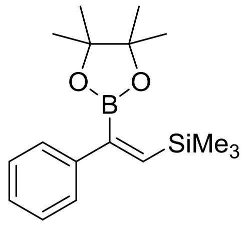

Dichlorophenylborane (0.64 mL, 5.0 mmol, 1.1 eq.) and trimethylsilylacetylene (0.65 mL, 4.55 mmol, 1.0 eq.) were added to an oven-dried Schlenk flask under argon-atmosphere. The mixture was dissolved in CH_2Cl_2 (7 mL) and refluxed for 1 h. A precooled solution of pinacol (590 mg, 5.0 mmol, 1.1 eq.) in trimethylamine (1mL) was carefully added to the chilled mixture dropwise. After stirring at room temperature for 30 min the solvent was evaporated *in vacuo*, the residue was diluted with cyclohexane (20 mL), filtered and concentrated under reduced pressure. Purification by column chromatography (SiO_2, CH/CH_2Cl_2 20:1) yielded ***E*-23** as a yellow oil (135 mg, 9%); analytical data in agreement with the literature.[11]

^{1}H NMR (400 MHz, $CDCl_3$) = δ 7.42 – 7.35 (m, 2H), 7.29 (ddd, *J* = 7.9, 7.0, 1.4 Hz, 2H), 7.24 – 7.15 (m, 1H), 6.72 (s, 1H), 1.33 (s, 12H), 0.21 (s, 9H) ppm.

Benzyldimethylethynylsilane (36)[6]

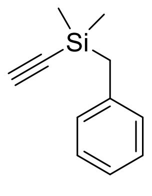

To an oven-dried two-neck flask equipped with a reflux condenser was added benzyldimethylchlorosilane (2 mL, 10.8 mmol, 1 eq.) and dry THF (5 mL) under a dry argon atmosphere. Ethynylmagnesium bromide (21.5 mL of 0.5 M solution in THF, 10.8 mmol, 1.0 eq.) was added via syringe and the solution was heated to reflux with stirring for 3 h. After the reaction was complete, solvent was removed under reduced pressure and the crude residue was purified by short path distillation to afford the desired product as a colorless oil (1.3 g, 69%). Analytical data was in agreement with the literature.[6]

^{1}H NMR (300 MHz, $CDCl_3$) δ = 7.29 – 7.21 (m, 2H), 7.16 – 7.07 (m, 3H), 2.44 (s, 1H), 2.24 (s, 2H), 0.17 (s, 6H) ppm.

(*E*)-(2-(Benzyldimethylsilyl)-1-phenylvinyl)boronic acid, pinacol ester (*E*-24)

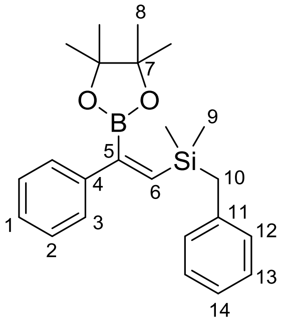

To an oven-dried Schlenk flask was added **36** (523 mg, 3 mmol, 1.0 eq.). The flask was sealed and purged with argon before the addition of DCM (10 mL, 0.3 M), and dichlorophenyl borane (440 µL, 3.3 mmol, 1.1 eq.) via syringe. The flask was heated to 60 °C with stirring for 1 h. The reaction was allowed to cool to room temperature and was added slowly to a precooled solution of pinacol (390 mg, 3.3 mmol, 1.1 eq.) in trimethylamine (1 mL). After stirring for 5 min solvent was removed from the reaction mixture under reduced pressure, and the resulting solution passed through a pad of silica eluting with DCM (5 mL). The filtrate was concentrated under reduced pressure and the crude residue was purified by column chromatography (SiO_2, 0 – 5 % EtOAc/CH) to afford the desired product as a yellow solid (295 mg, 26%).

R_f = 0.63 (EtOAc/CH, 1:9); **M.p.** 76.4 – 78.3 ˚C; **HR-ESI-MS:** m/z: 401.2090 ($[M+Na]^+$, calcd. for $C_{23}H_{31}O_2BSiNa^+$: 401.2083); **^{1}H NMR** (600 MHz, $CDCl_3$) δ = 7.42 – 7.37 (m, 2H, H3), 7.35 – 7.29 (m, 2H; H2), 7.28 – 7.20 (m, 3H; H1/13), 7.12 – 7.06 (m, 3H; H12/H14), 6.71 (s, 1H; H6), 2.39 (s, 2H; H10), 1.38 (s, 12H; H8), 0.21 (s, 6H; H9) ppm; **^{13}C NMR** (151 MHz, $CDCl_3$) δ = 149.1 (C6), 145.8 (C4), 140.7 (C11), 128.5 (2C, C12), 128.2 (2C, C13), 128.1 (2C, C2), 127.2 (2C, C3), 127.0 (C1), 124.0 (C14), 84.1 (2C, C7), 26.8 (C10), 25.3 (4C, C8), -1.6 (2C, C9) ppm; **^{11}B NMR** (192 MHz, $CDCl_3$) δ = 30.12 ppm; **IR (ATR):** $\tilde{\nu}$ = 2976(w), 1596(w), 1544(w), 1490(w), 1450(w), 1373(m), 1323(m), 1237(m), 1201(s), 1138(s), 814(s), 761(s) cm^{-1}.

(*Z*)-(2-(4-Fluorophenyl)-2-(tributylstannyl)vinyl)trimethylsilane (*Z*-25)

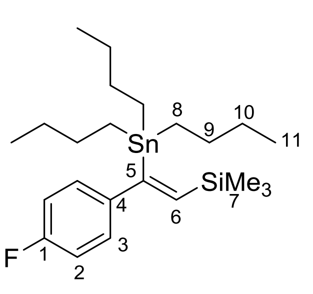

Prepared according to General Procedure D, 1-ethynyl-4-fluorobenzene (120 mg, 1 mmol, 1 eq.) was converted to **Z-25** in 1 h yielding a colourless oil (460 mg, 95%) after purification by column chromatography (100% *n*-pentane).

R_f = 0.86 (*n*-pentane); **GC-EI-MS:** m/z: 469.17375 ($[M-CH_3]^+$, calcd. for $C_{22}H_{38}FSiSn^+$: 469.17487); **^{1}H NMR** (500 MHz, $CDCl_3$): δ = 6.95 (d, *J* = 0.9 Hz, 2H; H2), 6.94 – 6.93 (m, 2H; H3), 6.54 (s, 1H; H6), 1.48 – 1.34 (m, 6H; H8), 1.32 – 1.22 (m, 6H; H9), 0.94 – 0.89 (m, 6H; H10), 0.86 (t, *J* = 7.3 Hz, 9H; H11), 0.18 (s,

9H; H7).; **^{13}C NMR:** (126 MHz, $CDCl_3$) δ = 165.1 (C5), 161.3 (d, J_{CF} = 243.7 Hz; C1), 149.1 (d, J_{CF} = 1.1 Hz; C6), 148.07 (d, J_{CF} = 3.2 Hz; C4), 127.5 (d, J_{CF} = 7.7 Hz; 2C, C3), 114.7 (d, J_{CF} = 21.2 Hz; 2C, C2), 29.2 (3C, C8), 27.5 (3C, C9), 13.8 (3C, C11), 12.1 (3C, C10), 0.3 (3C, C7).; **^{19}F NMR** (470 MHz, $CDCl_3$) δ = -118.20 ppm; **IR (ATR):** ῦ = 2955(m), 2921(m), 2871(w), 2854(w), 1598(w), 1499(s), 1247(m), 1220(m), 1154(w), 878(s), 855(s), 810(s) cm^{-1}.

(*Z*)-(2-(3-Chlorophenyl)-2-(tributylstannyl)vinyl)trimethylsilane (*Z*-26)

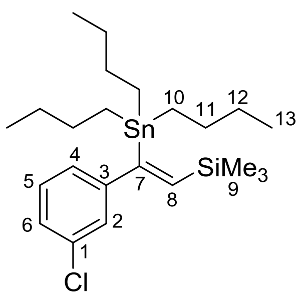

Prepared according to General Procedure D, 1-chloro-3-ethynylbenzene (123 µL, 1 mmol, 1 eq.) was converted to ***Z*-26** in 1 h yielding a colorless oil (482 mg, 96%) after purification by column chromatography (SiO_2, 100% *n*-pentane). Containing <5% des-silyl product.

R_f = 0.86 (*n*-pentane); **GC-EI-MS:** m/z: 485.14444 ($[M-CH_3]^+$, calcd. for $C_{22}H_{38}ClSiSn^+$: 485.14429); **^{1}H NMR** (500 MHz, $CDCl_3$) δ = 7.18 (t, *J* = 7.8 Hz, 1H; H5), 7.11 (ddd, *J* = 8.0, 2.1, 1.2 Hz, 1H; H6), 6.97 (ddd, *J* = 2.1, 1.7, 0.4 Hz, 1H; H2), 6.84 (ddd, *J* = 7.6, 1.7, 1.1 Hz, 1H; H4), 1.46 – 1.37 (m, 6H; H10), 1.32 – 1.22 (m, 6H; H11), 0.94 – 0.89 (m, 6H; H12), 0.86 (t, *J* = 7.3 Hz, 9H; H13), 0.18 (s, 9H; H9) ppm; **^{13}C NMR** (126 MHz, $CDCl_3$) δ = 164.8 (C7), 153.9 (C3), 149.6 (C8), 133.8 (C1), 129.2 (C5), 126.1 C2), 125.5 (C6), 124.3 (C4), 29.2 (3C, C10), 27.5 (3C, C11), 13.7 (3C, C13), 12.2 (3C, C12), 0.2 (3C, C9) ppm; **IR (ATR):** ῦ = 2955(m), 2920(m), 2871(w), 2854(w), 1588(w), 1560(w), 1464(m), (1376(w), 1246(m), 1075(w), 855(s), 832(s), 766(s), 686(s) cm^{-1}.

(*Z*)-Trimethyl(2-(p-tolyl)-2-(tributylstannyl)vinyl)silane (*Z*-27)

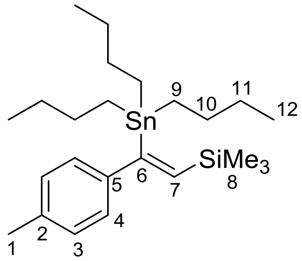

Prepared according to General Procedure D, 1-ethynyl-4-methylbenzene (127 µL, 1 mmol, 1 eq.) was converted to ***Z*-27** in 1 h yielding a colorless oil (437 mg, 91%) after purification by column chromatography (SiO_2, 100% *n*-pentane).

R_f = 0.86 (*n*-pentane); **HR-ESI-MS:** m/z: 503.2117 ($[M+Na]^+$, calcd. for $C_{24}H_{44}SiSnNa^+$: 503.2130); **^{1}H NMR** (400 MHz, $CDCl_3$) δ = 7.12 – 7.06 (m, 2H; H3), 6.96 – 6.90 (m, 2H; H4), 6.58 (s, 1H; H7), 2.35 (s, 3H; H1), 1.54 – 1.40 (m,

6H; H9), 1.36 – 1.25 (m, 6H; H10), 1.01 – 0.92 (m, 6H; H11), 0.89 (t, *J* = 7.3 Hz, 9H; H12), 0.21 (s, 9H; H8) ppm; **^{13}C NMR** (101 MHz, $CDCl_3$) δ = 166.0 (C6), 149.2 (C2), 148.1 (C7), 135.1 (C5), 128.7 (2C, C3), 126.0 (2C, C4), 29.2 (3C, C9), 27.5 (3C, C10), 21.2 (C1), 13.8 (3C, C12), 12.2 (3C, C11), 0.4 (3C, C8) ppm; **IR (ATR):** $\tilde{\upsilon}$ = 2954(m), 2919(m), 2871(w), 1503(m), 1463(w), 1376(w), 1246(s), 1071(w), 877(s), 852(s), 832(s) cm^{-1}.

2.2. Photosensitised isomerisation of vinyl silanes

General Procedure E for the isomerisation of vinyl silanes

A round-bottom flask was charged with the specific vinyl silane (0.1 mmol, 1.0 eq.) and benzophenone (0.005 mmol, 0.05 eq.) in degassed cyclohexane (1.5 mL). The reaction vessel was sealed with a septum, equipped with an argon balloon and placed above the UV-lamp with a distance of approximately 0.5 cm. The mixture was stirred for 2 h at room temperature under UV light irradiation (UVA LED, 365 nm). The crude reaction mixture was filtered through a plug of SiO_2, diluted with cyclohexane (5 mL) and concentrated *in vacuo*. The *E*-/*Z*-isomer ratio was determined by the integration of the ^{1}H NMR spectra.

(*Z*)-Trimethyl(2-phenylbut-1-en-1-yl)silane (*Z*-1)

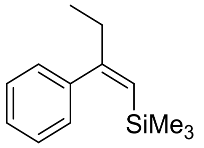

According to the General Procedure E, ***E*-1** (20.4 mg, 0.1 mmol, 1.0 eq.) was converted to ***Z*-1** yielding a colorless oil (quant., *Z*/*E* 95:5); analytical data in agreement with the literature.[12]

^{1}H NMR (400 MHz, $CDCl_3$) δ = 7.33 – 7.21 (m, 3H), 7.17 – 7.09 (m, 2H), 5.55 (s, 1H), 2.40 (q, *J* = 7.4 Hz, 2H), 1.01 (t, *J* = 7.4 Hz, 3H), -0.19 (s, 9H).

(*Z*)-Trimethyl(styryl)silane (*Z*-2)

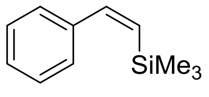

According to General Procedure E, ***E*-2** (17.6 mg, 0.1 mmol, 1.0 eq.) was converted to ***Z*-2** yielding a colorless oil (15.8 mg, 90%, *Z*/*E* 66:34); analytical data in agreement with the literature.[13]

^{1}H NMR (300 MHz, $CDCl_3$) δ = 7.89 – 7.27 (m, 6H), 5.84 (d, *J* = 15.1 Hz, 1H), 0.06 (s, 9H) ppm.

(*Z*)-*tert*-Butyldimethyl(styryl)silane (*Z*-3)

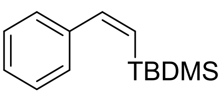

According to General Procedure E, ***E*-3** (21.8 mg, 0.1 mmol, 1 eq.) was converted to ***Z*-3** yielding a colorless oil (20.2 mg, 93%; *Z/E* 67:33). Analytical data was in agreement with the literature.[6]

^{1}H NMR (400 MHz, $CDCl_3$) δ = 7.48 (d, *J* = 15.4 Hz, 1H), 7.34 – 7.22 (m, 5H), 5.87 (d, *J* = 15.4 Hz, 1H), 0.91 (s, 9H), -0.07 (s, 6H) ppm.

(*Z*)-Triisopropyl(styryl)silane (*Z*-4)

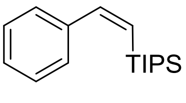

Prepared according to General Procedure E, ***Z*-4** (26.1 mg, 0.1 mmol, 1 eq.) was subjected to standard conditions to yield a mixture of isomers as a colorless oil (25.4 mg, 97%; *Z/E* 67:33). Analytical data was in agreement with the literature.[6]

^{1}H NMR (300 MHz, $CDCl_3$) δ = 7.50 (d, *J* = 15.6 Hz, 1H), 7.32 – 7.20 (m, 5H), 5.72 (d, *J* = 15.7 Hz, 1H), 1.17 – 0.85 (m, 21H) ppm.

(*Z*)-Trimethyl(2-phenylprop-1-en-1-yl)silane (*Z*-5)

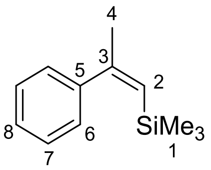

According to the General Procedure E, ***E*-5** (19.0 mg, 0.1 mmol, 1.0 eq.) was converted to ***Z*-5** yielding a colorless oil (quant., *Z*/*E* 90:10).

R_f = 0.73 (CH); **GC-EI-MS**: m/z: 190.11719 ($[M]^+$, calcd. for $C_{12}H_{18}Si_2^+$: 190.11723); **^{1}H NMR** (600 MHz, CD_2Cl_2) δ = 7.31 – 7.28 (m, 2H; H7), 7.28 – 7.23 (m, 1H; H8), 7.21 – 7.17 (m, 2H; H6), 5.60 (q, *J* = 1.4 Hz, 1H; H2), 2.17 (d, *J* = 1.4 Hz, 3H; H4), -0.18 (s, 9H; H1) ppm; **^{13}C NMR** (151 MHz, CD_2Cl_2) δ = 156.0 (C3), 145.4 (C5), 128.6 (C2), 128.4 (2C, C7), 128.0 (2C, C6), 127.5 (C8), 30.1 (C4), 0.3 (3C, C1) ppm; **IR (ATR):** $\tilde{\upsilon}$ = 2954(w), 2905(w), 1610(w), 1595(w), 1491(w), 1436(w), 1259(w), 246(m), 1062(w), 1027(w), 859(m), 833 (s), 783(w), 762(m), 753(m), 698(s) cm^{-1}.

(*Z*)-(2-(4-Fluorophenyl)but-1-en-1-yl)trimethylsilane (*Z*-6)

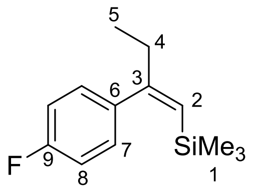

According to General Procedure E, ***E*-6** (22.2 mg, 0.1 mmol, 1.0 eq.) was converted to ***Z*-6** yielding a colorless oil (20.4 mg, 92%, *Z*/*E* 95:5).

Rf = 0.73 (CH); **HR-APCI-MS**: m/z: 329.02861 ([M+Ag]$^+$, calcd. for $C_{13}H_{19}FSiAg^+$: 329.02855); **^{1}H NMR** (600 MHz, CD_2Cl_2) δ = 7.17 – 7.07 (m, 2H; H7), 7.03 – 6.95 (m, 2H; H8), 5.58 (t, *J* = 1.5 Hz, 1H; H2), 2.38 (qd, *J* = 7.4, 1.5 Hz, 2H; H4), 0.99 (t, *J* = 7.4 Hz, 3H; H5), -0.18 (s, 9H; H1) ppm; **^{13}C NMR** (151 MHz, CD_2Cl_2) δ 162.5 (d, J_{CF} = 244.1 Hz, C9), 160.7 (C3), 141.0 (d, J_{CF} = 3.3 Hz, C6), 130.2 (d, J_{CF} = 8.0 Hz, 2C, C7), 115.0 (d, J_{CF} = 21.2 Hz, 2C, C8), 35.9 (C4), 12.9 (C5), 0.3 (3C, C1) ppm; **^{19}F NMR** (564 MHz, CD_2Cl_2) δ = -116.96 ppm; **IR (ATR):** ṽ = 2956(w), 2926(w), 2870(w), 1605(w), 1492(w), 1450(w), 1335(w), 1252(m), 1063(s), 839(m), 761(s), 698(s) cm^{-1}.

(*Z*)-(2-(4-Bromophenyl)but-1-en-1-yl)trimethylsilane (*Z*-7)

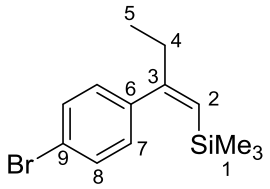

According to General Procedure E, ***E*-7** (28.2 mg, 0.1 mmol, 1.0 eq.) was converted to ***Z*-7** yielding a colorless oil (27.6 mg, 98%, *Z*/*E* 94:6).

R_f = 0.77 (CH); **GC-EI-MS**: m/z: 282.0433 ([M]$^+$, calcd. for $C_{13}H_{19}BrSi^+$: 282.0439); **^{1}H NMR** (600 MHz, CD_2Cl_2) δ = 7.45 – 7.42 (m, 2H; H8), 7.06 – 7.02 (m, 2H; H7), 5.59 (t, *J* = 1.5 Hz, 1H; H2), 2.38 (qd, *J* = 7.4, 1.4 Hz, 2H; H4), 0.99 (t, *J* = 7.4 Hz, 3H; H5), -0.18 (s, 9H; H1) ppm; **^{13}C NMR** (151 MHz, CD_2Cl_2) δ = 160.4 (C3), 144.0 (C6), 131.4 (2C, C8), 130.4 (2C, C7), 126.6 (C2), 121.2 (C9), 35.7 (C4), 12.9 (C5), 0.3 (3C, C1) ppm; **IR (ATR):** ṽ = 2960(w), 2933(w), 2897(w), 1607(w), 1483(m), 1459(w), 1246(m), 1099(w), 1070(m), 1010(m), 946(w), 927(w), 850(s), 826(s), 763(m), 746(m), 724(w), 700(m), 690(m) cm^{-1}.

(*Z*)-Trimethyl(2-(p-tolyl)but-1-en-1-yl)silane (*Z*-8)

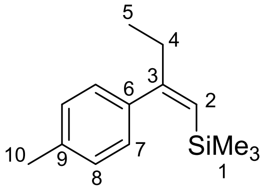

According to General Procedure E, ***E*-8** (21.8 mg, 0.1 mmol, 1.0 eq.) was converted to ***Z*-8** yielding a colorless oil (quant., *Z*/*E* 93:7).

Rf = 0.77 (CH); **HR-APCI-MS**: m/z: 325.05364 ($[M+Ag]^+$, calcd. for $C_{14}H_{22}SiAg^+$: 325.05362); **^{1}H NMR** (500 MHz, CD_2Cl_2) δ = 7.14 – 7.08 (m, 2H; H8), 7.05 – 6.96 (m, 2H; H7), 5.53 (t, *J* = 1.5 Hz, 1H; H2), 2.39 (qd, *J* = 7.4, 1.5 Hz, 2H; H4), 2.34 (d, *J* = 0.6 Hz, 3H; H10), 0.99 (t, *J* = 7.4 Hz, 3H; H5), -0.19 (s, 9H; H1) ppm; **^{13}C NMR** (126 MHz, CD_2Cl_2) δ = 161.9 (C3), 142.0 (C6), 137.0 (C9), 128.9 (2C, C7), 128.4 (2C, C8), 125.3 (C2), 35.8 (C4), 21.4 (C10), 13.1 (C5), 0.4 (C1) ppm; **IR (ATR):** $\tilde{\upsilon}$ = 2962(w), 1601(w), 1509(w), 1455(w), 1245(m), 1110(w), 1020(w), 946(w), 928(w), 864(s), 847(s), 831(s), 769(m), 745(m), 727(m), 688(m) cm^{-1}.

(*Z*)-(2-(4-Methoxyphenyl)but-1-en-1-yl)trimethylsilane (*Z*-9)

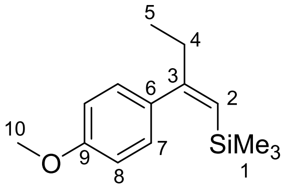

According to General Procedure E, ***E*-9** (23.4 mg, 0.1 mmol, 1.0 eq.) was converted to ***Z*-9** yielding a colorless oil (22.9 mg, 98%; *Z*/*E* 93:7).

Rf = 0.80 (EtOAc/CH = 1:7); **GC-EI-MS**: m/z: 234.14351 ($[M]^+$, calcd. for $C_{14}H_{22}OSi^+$: 234.14344); **^{1}H NMR** (600 MHz, CD_2Cl_2) δ = 7.09 – 7.04 (m, 2H; H7), 6.87 – 6.80 (m, 2H; H8), 5.53 (t, *J* = 1.4 Hz, 1H; H2), 3.80 (s, 3H; H10), 2.39 (qd, *J* = 7.4, 1.4 Hz, 2H; H4), 0.99 (t, *J* = 7.4 Hz, 3H; H5), -0.17 (s, 9H; H1) ppm; **^{13}C NMR** (151 MHz, CD_2Cl_2) δ = 161.5 (C3), 159.3 (C9), 137.4 (C6), 129.6 (2C, C7), 125.4 (C2), 113.6 (2C, C8), 55.7 (C10), 35.9 (C4), 13.1 (C5), 0.4 (C1)ppm; **IR (ATR):** $\tilde{\upsilon}$ = 2954(w), 2835(w), 1609(m), 1507(m), 1461(w), 1286(w), 1241(s), 1172(m), 1107(w), 1034(m), 941(w), 924(w), 864(s), 828(s), 805(s), 745(m), 688(m) cm^{-1}.

(*Z*)-Trimethyl(2-(m-tolyl)but-1-en-1-yl)silane (*Z*-10)

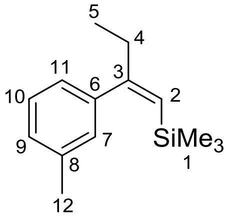

According to General Procedure E, ***E*-10** (21.8 mg, 0.1 mmol, 1.0 eq.) was converted to ***Z*-10** yielding a colorless oil (20.5 mg, 94%), *Z*/*E* 95:5).

R_f = 0.79 (CH); **GC-EI-MS**: m/z: 218.14848 ([M]$^+$, calcd. for $C_{14}H_{22}Si^+$: 218.14853); **^{1}H NMR** (600 MHz, CD_2Cl_2) δ = 7.18 (t, *J* = 7.5 Hz, 1H; H10), 7.07 (dddd, *J* = 7.6, 1.9, 1.2, 0.7 Hz, 1H; H9), 6.97 (tt, *J* = 1.8, 0.7 Hz, 1H; H7), 6.94 (dddd, *J* = 7.5, 1.8, 1.2, 0.6 Hz, 1H; H11), 5.54 (t, *J* = 1.5 Hz, 1H; H2), 2.40 (qd, *J* = 7.4, 1.5 Hz, 2H; H4), 2.34 (s, 3H; H12), 1.00 (t, *J* = 7.4 Hz, 3H; H5), -0.19 (s, 9H; H1) ppm; **^{13}C NMR** (151 MHz, CD_2Cl_2) δ = 162.1 (C3), 144.9 (C6), 137.8 (C8), 129.4 (C7), 128.1 (C10), 128.0 (C9), 125.5 (C11), 125.4 (C2), 35.7 (C4), 21.7 (C12), 13.0 (C5), 0.4 (3C, C1) ppm; **IR (ATR):** $\tilde{\upsilon}$ = 2955(w), 1596(w), 1580(w), 1459(w), 1259(w), 1245(m), 847(s), 832(s), 786(m), 768(m), 745(m), 708(m), 688(m) cm^{-1}.

(*Z*)-Trimethyl(2-methylstyryl)silane (*Z*-11)

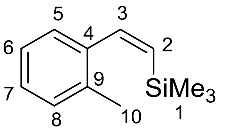

According to General Procedure E, ***E*-11** (19.0 mg, 0.1 mmol, 1.0 eq.) was converted to ***Z*-11** yielding a colorless oil (16.5 mg, 87%, *Z*/*E* 78:22).

R_f = 0.80 (CH); **GC-EI-MS**: m/z: 190.11718 ([M]$^+$, calcd. for $C_{12}H_{18}Si^+$: 190.11723) ; **^{1}H NMR** (CD_2Cl_2) δ = 7.42 (d, *J* = 14.9 Hz, 1H; H3), 7.23 – 7.04 (m, 5H; H5; H6; H7; H8), 5.90 (d, *J* = 14.9 Hz, 1H; H2), 2.25 (s, 3H; H10), -0.07 (s, 9H; H1) ppm; **^{13}C NMR** (126 MHz, CD_2Cl_2) δ = 146.8 (C3), 140.7 (C4), 136.3 (C9), 133.5 (C2), 129.9 (C8), 129.2 (C5/C6/C7), 128.0 (C5/C6/C7), 125.8 (C5/C6/C7), 20.1 (C10), 0.2 (3C, C1) ppm; **IR (ATR):** $\tilde{\upsilon}$ = 2956(w), 1594(w), 1570(w), 1483(w), 1456(w), 1245(m), 1155(w), 1104(w), 1044(w), 988(w), 942(w), 859(m), 836(s), 797(m), 766(m), 756(m), 743(m), 728(m), 689(m), 666(m) cm^{-1}.

(*Z*)-(4-(1-(Trimethylsilyl)but-1-en-2-yl)phenyl)boronic acid, pinacol ester (*Z*-12)

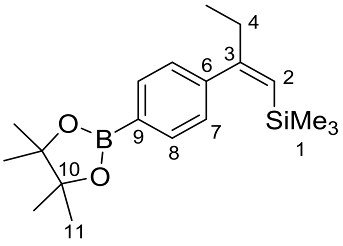

According to General Procedure E, ***E*-12** (33.0 mg, 0.1 mmol, 1.0 eq.) was converted to ***Z*-12** yielding a colorless oil (24.8 mg, 75%, *Z*/*E* 91:9).

R_f = 0.78 (CH); **HR-ESI-MS**: m/z: 353.2073 ([M+Na]$^+$, calcd. for $C_{19}H_{31}BO_2SiNa^+$: 353.2079); **^{1}H NMR** (500 MHz, CD_2Cl_2) δ = 7.70 – 7.67 (m, 2H; H8), 7.17 – 7.13 (m, 2H; H7), 5.58 (t, *J* = 1.5 Hz, 1H; H2), 2.40 (qd, *J* = 7.4, 1.5 Hz, 2H; H4), 1.34 (s, 12H; H11), 0.99 (t, *J* = 7.4 Hz, 3H; H5), -0.20 (s, 9H; H1) ppm; **^{13}C NMR** (126 MHz, CD_2Cl_2) δ = 161.7 (C3), 148.0 (C6), 134.7 (2C, C8),

128.0 (2C, C7), 125.9 (C2), 84.3 (2C, C10), 35.7 (C4), 25.3 (4C, C11), 13.0 (C5), 0.4 (3C, C1) ppm (C9 missing due to quadrupolar relaxation); **IR (ATR):** ῦ = 2976(w), 1601(m), 1510(w), 1456(w), 1396(m), 1355(s), 1318(m), 1260(m), 1246(m), 1143(s), 1088(m), 1020(m), 962(m), 930(w), 850(s), 831(s), 749(m), 696(m), 659(s) cm^{-1}.

(*E*)-(1-Phenylethene-1,2-diyl)bis(trimethylsilane) (*E*-13)

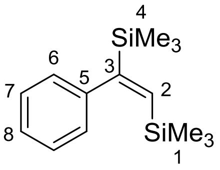

According to General Proceudure E, ***Z*-13** (24.8 mg, 0.1 mmol, 1.0 eq.) was converted to ***E*-13** yielding a colorless oil (24.3 mg, 98%, *E*/*Z* 90:10).

Rf = 0.77 (CH); **GC-EI-MS**: m/z: 248.14107 ($[M]^+$, calcd. for $C_{14}H_{24}Si_2^+$: 248.14111); **^{1}H NMR** (500 MHz, CD_2Cl_2) δ = 7.30 – 7.23 (m, 2H; H7), 7.21 – 7.14 (m, 1H; H8), 6.97 – 6.85 (m, 2H; H6), 6.35 (s, 1H; H2), 0.05 (s, 9H; H4), -0.20 (s, 9H; H1) ppm; **^{13}C NMR** (126 MHz, CD_2Cl_2) δ = 167.1 (C3), 146.1 (C5), 144.4 (C2), 128.1 (2C, C7), 127.9 (2C, C6), 126.1 (C8), 0.2 (3C, C1), -1.5 (3C, C4) ppm; **IR (ATR):** ῦ = 3075(w), 2954(w), 2897(w), 1597(w), 1486(w), 1440(w), 1404(w), 1306(w), 1245(s), 1171(w), 1070(w), 1029(w), 933(m), 829(s), 772(m), 754(m), 700(s), 688(m) cm^{-1}.

(*Z*)-Benzyldimethyl(2-phenylbut-1-en-1-yl)silane (*Z*-14)

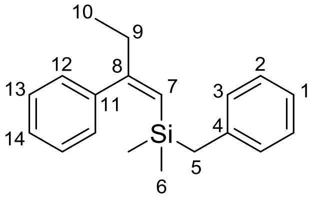

According to General Procedure E, ***E*-14** (28.0 mg, 0.1 mmol, 1.0 eq.) was converted to ***Z*-14** yielding a colorless oil (26.6 mg, 95%; *Z*/*E* 95:5).

Rf = 0.52 (CH); **GC-EI-MS**: m/z: 265.14089 ($[M-CH_3]^+$, calcd. for $C_{18}H_{21}Si^+$: 265.14070); **^{1}H NMR** (500 MHz, CD_2Cl_2) δ = 7.32 – 7.23 (m, 3H; H13; H14), 7.18 (dd, *J* = 8.3, 7.0 Hz, 2H; H2), 7.11 – 7.00 (m, 3H; H1; H12), 6.98 – 6.92 (m, 2H; H3), 5.55 (t, *J* = 1.5 Hz, 1H; H7), 2.42 (qd, *J* = 7.4, 1.5 Hz, 2H; H9), 1.97 (s, 2H; H5), 1.01 (t, *J* = 7.4 Hz, 3H; H10), -0.28 (s, 6H; H6) ppm; **^{13}C NMR** (126 MHz, CD_2Cl_2) = δ 162.9 (C8), 144.9 (C11), 141.1 (C4), 128.8 (2C, C3), 128.5 (2C, C2), 128.5 (2C, C12), 128.3 (2C, C13), 127.4 (C14), 124.4 (C1), 123.7 (C7), 36.0 (C9), 27.3 (C5), 13.0 (C10), -1.76 (C6) ppm; **IR (ATR):** ῦ = 3059(w), 3024(w), 2963(w), 1594(w), 1492(m), 1451(w), 1441(w), 1295(w), 1246(m), 1206(w), 1152(w), 1077(w), 1056(w), 1027(w), 1001(w), 935(w), 904(w), 846(s), 826(s), 809(m), 758(m), 696(s) cm^{-1}.

(Z)-1-Methyl-1-(2-phenylbut-1-en-1-yl)siletane (*Z*-15)

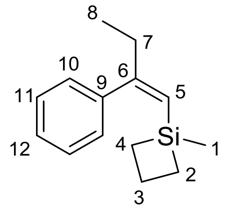

According to General Procedure E, ***E*-15** (21.6 mg, 0.1 mmol, 1.0 eq.) was converted to ***Z*-15** yielding a colorless oil (20.5 mg, 95%; *Z*/*E* 95:5).

Rf = 0.73 (CH); **GC-EI-MS**: m/z: 201.10951 ($[M-CH_3]^+$, calcd. for $C_{13}H_{17}Si^+$: 201.10940); **^{1}H NMR** (500 MHz, CD_2Cl_2) δ = 7.32 – 7.23 (m, 3H; H11; H12), 7.21 – 7.16 (m, 2H; H10), 5.75 (t, *J* = 1.4 Hz, 1H; H5), 2.49 (qd, *J* = 7.4, 1.4 Hz, 2H; H7), 1.98 – 1.77 (m, 2H; H3), 1.05 (t, *J* = 7.4 Hz, 3H; H8), 0.82 – 0.75 (m, 4H; H2; H4), 0.02 (s, 3H; H1) ppm; **^{13}C NMR** (126 MHz, CD_2Cl_2) δ = 162.8 (C6), 144.5 (C9), 128.4 (2C, C11), 128.2 (2C, C10), 127.7 (C12), 124.9 (C5), 34.8 (C7), 18.4 (C3), 16.0 (2C, C2, C4), 13.1 (C8), -0.7 (C1) ppm; **IR (ATR):** ῦ = 2963(w), 2930(w), 1593(w), 1572(w), 1490(w), 1441(w), 1394(w), 1247(w), 1185(w), 1118(m), 1079(w), 1026(w), 933(w), 903(m), 866(m), 840(m), 765(m), 697(s) cm^{-1}.

(*Z*)-Dimethyl(2-phenylbut-1-en-1-yl)silanol (*Z*-20)

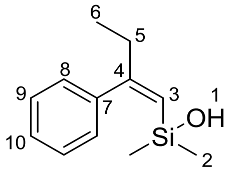

Acoording to General Procedure E, ***E*-20** (20.6 mg, 0.1 mmol, 1.0 eq.) was converted to ***Z*-20** yielding a colorless oil (19.2 mg, 93 %).

R$_f$ = 0.33 (EtOAc/ CH 1:8); **HR-ESI-MS:** m/z: 229.1027 ($[M+Na]^+$, calcd. for $C_{12}H_1NaOSi^+$: 229.1019); **^{1}H NMR** (500 MHz, CD_2Cl_2) δ = 7.35 – 7.27 (m, 3H, H-9, H-10), 7.23 – 7.17 (m, 2H, H-8), 5.58 (t, *J* = 1.5 Hz, 1H, H-C3), 2.43 (qd, *J* = 7.4, 1.5 Hz, 2H, H-C5), 1.02 (td, *J* = 7.4, 0.5 Hz, 3H), -0.08 (s, 4H) ppm; **^{13}C NMR** (126 MHz, CD_2Cl_2) δ = 162.8 (C4), 144.8 (C7), 128.6 (2C, C9), 128.3 (2C, C8), 127.7 (C10), 125.0 (C3), 35.4 (C5), 12.8 (C6), 1.6 (2C, C2) ppm; **IR (ATR):** ῦ = 3284 (b), 2963(w), 1594(w), 1490(w), 1441(w), 1250(m), 1077(w), 1026(w), 935(w), 836(s), 766(s), 699(s) cm^{-1}.

(*E*)-Trimethyl(2-phenyl-2-(tributylstannyl)vinyl)silane (*E*-22)

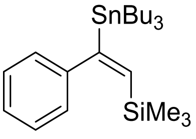

According to General Procedure E, ***Z*-22** (46.6 mg, 0.1 mmol, 1.0 eq.) was converted to ***E*-22** yielding a colorless oil (42.4 mg, 91 %, *E*/*Z* 74:26); analytical data in agreement with the literature.[14]

^{1}**H NMR** (300 MHz, $CDCl_3$) δ = 7.24 (ddt, *J* = 9.3, 7.9, 1.5 Hz, 2H), 7.16 – 7.07 (m, 1H), 6.97 – 6.87 (m, 2H), 6.15 (s, 1H), 1.53 – 1.36 (m, 6H), 1.34 – 1.18 (m, 6H), 0.93 – 0.80 (m, 15H), -0.15 (s, 9H) ppm.

(*E*)-(1-Phenyl-2-(trimethylsilyl)vinyl)boronic acid, pinacol ester (*Z*-23)

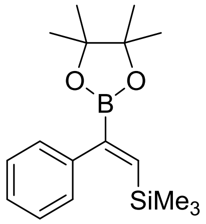

According to General Procedure E, ***E*-23** (30.2 mg, 0.1 mmol, 1.0 eq.) was converted to ***Z*-23** giving a light yellow oil (19.9 mg, 66%, *Z*/*E* 80:20); analytical data in agreement with the literature.[15]

^{1}**H NMR** (400 MHz, $CDCl_3$) δ = 7.42 – 7.02 (m, 5H), 6.84 (s, 1H), 1.27 (s, 12H), -0.12 (s, 9H) ppm.

(*Z*)-(2-(Benzyldimethylsilyl)-1-phenylvinyl)boronic acid, pinacol ester (*Z*-24)

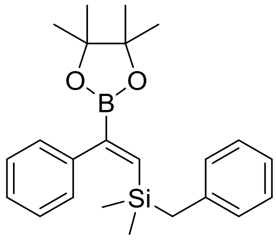

According to General Procedure E, ***E*-24** (37.8 mg, 0.1 mmol, 1.0 eq.) was converted to ***Z*-24** yielding a colorless oil (27.6 mg, 73%; *Z/E* 81:19). Product was unstable on SiO_2 and could only be isolated quickly containing a benzophenone impurity.

R$_f$ = 0.60 **GC-EI-MS**: m/z: 363.19476 ($[M-CH_3]^+$, calcd. for $C_{22}H_{28}BOSi^+$: 363.19461); ^{1}**H NMR** (600 MHz, $CDCl_3$) δ = 7.29 – 7.24 (m, 2H), 7.24 – 7.20 (m, 1H), 7.19 – 7.14 (m, 2H), 7.13 – 7.09 (m, 2H), 7.06 – 7.02 (m, 1H), 6.92 – 6.89 (m, 2H), 6.83 (s, 1H), 1.98 (s, 2H), 1.28 (s, 12H), -0.20 (s, 6H). ppm; ^{13}**C NMR** (151 MHz, $CDCl_3$) δ = 148.7, 143.7, 140.2, 128.4, 128.2, 128.2, 127.8, 126.7, 124.1, 84.0, 26.5, 24.9, -2.0. ppm; ^{11}**B NMR** (192 MHz, $CDCl_3$): δ = 29.77 ppm; **IR (ATR):** ῦ = 2978(m), 1604(w), 1438(w), 1389(m), 1356(s), 1322(s), 1269(s), 1211(w), 1141(s), 1089(s), 1027(s), 858(m), 697(s), 654(s) cm^{-1}.

(*E*)-(2-(4-Fluorophenyl)-2-(tributylstannyl)vinyl)trimethylsilane (*E*-25)

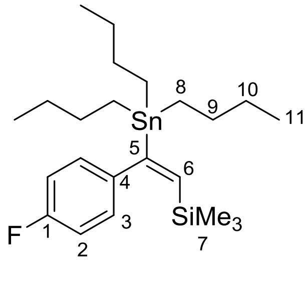

Prepared according to General Procedure E, ***Z*-25** (48.3 mg, 0.1 mmol, 1.0 eq.) was converted to ***E*-25**, employing thioxanthone (1 mg, 0.005 mmol, 5 mol%) as a photocatalyst, yielding a colorless oil (39.1 mg, 81%; *E/Z* 82:18).

***R**_f* = 0.93 (*n*-pentane); **GC-EI-MS:** m/z: 427.12707 ($[M-C_4H_9]^+$, calcd. for $C_{19}H_{32}FSiSn^+$: 427.12764); **^{1}H NMR** (600 MHz, $CDCl_3$) δ = 6.96 – 6.90 (m, 2H; H2), 6.89 – 6.83 (m, 2H; H3), 6.15 (s, 1H; H6), 1.47 – 1.39 (m, 6H; H8), 1.33 – 1.21 (m, 6H; H9), 0.86 (app. t, *J* = 7.4 Hz, 15H; H10, 11), -0.16 (s, 9H; H7) ppm; **^{13}C NMR** (151 MHz, $CDCl_3$) δ = 167.8 (C5), 161.1 (d, J_{CF} = 243.2 Hz; C1), 147.1 (d, J_{CF} = 1.4 Hz; C6), 144.6 (d, J_{CF} = 3.2 Hz; C4), 127.5 (d, J_{CF} = 7.8 Hz; 2C, C3), 114.7 (d, J_{CF} = 21.2 Hz; 2C, C2), 29.1 (3C, C8), 27.4 (3C, C9), 13.8 (3C, C11), 10.2 (3C, C10), 0.5 (3C, C7) ppm; **^{19}F NMR** (564 MHz, $CDCl_3$) δ = -118.57 ppm; **IR (ATR):** ῦ = 2956(m), 2924(m), 2872(w), 2854(w), 1602(m), 1498(m), 1464(w), 1413(w), 1376(w), 1245(m), 1219(m), 1153(m), 850(s), 837(s), 751(m), 679(m) cm^{-1}.

(*E*)-(2-(3-Chlorophenyl)-2-(tributylstannyl)vinyl)trimethylsilane (*E*-26)

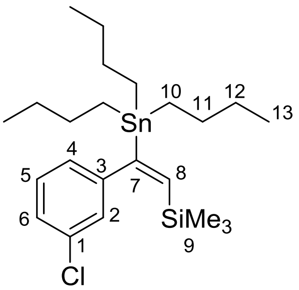

According to General Procedure E, ***Z*-26** (50.0 mg, 0.1 mmol, 1.0 eq.) was converted to ***E*-26**, employing thioxanthone (1 mg, 0.005 mmol, 5 mol%) as a photocatalyst, yielding a colorless oil (39.5 mg, 79%; *E/Z* 79:21).

***R**_f* = 0.93 (*n*-pentane); **GC-EI-MS:** m/z: 443.09767 ($[M-C_4H_9]^+$, calcd. for $C_{19}H_{32}ClSiSn^+$: 443.09742); **^{1}H NMR** (600 MHz, $CDCl_3$) δ = 7.16 (t, *J* = 7.8 Hz, 1H; H5), 7.10 (ddd, *J* = 7.9, 2.1, 1.1 Hz, 1H; H6), 6.91 (t, *J* = 1.9 Hz, 1H; H2), 6.79 (ddd, *J* = 7.6, 1.6, 1.1 Hz, 1H; H4), 6.16 (s, 1H; H8), 1.50 – 1.36 (m, 6H; H10), 1.32 – 1.23 (m, 6H; H11), 0.95 – 0.81 (m, 15H; H12, 13), -0.14 (s, 9H; H9) ppm; **^{13}C NMR** (151 MHz, $CDCl_3$) δ = 167.2 (C7), 150.5 (C3), 147.2 (C8), 133.7 (C1), 129.2 (C5), 126.1 (C2), 125.2 (C6), 124.3 (C4), 29.1 (3C, C10), 27.4 (3C, C11), 13.8 (3C, C13), 10.3 (3C, C12), 0.5 (3C, C9) ppm; **IR (ATR):** ῦ = 2955(m), 2924(m), 2871(w), 2853(w), 1590(w), 1557(w), 1464(m), 1376(w), 1246(s), 1074(m), 875(s), 855(s), 790(s), 676(s) cm^{-1}.

(*E*)-Trimethyl(2-(p-tolyl)-2-(tributylstannyl)vinyl)silane (*E*-27)

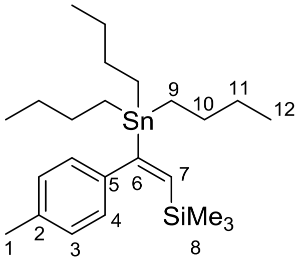

According to General Procedure E, ***Z*-27** (47.9 mg, 0.1 mmol, 1.0 eq.) was converted to ***E*-27**, employing thioxanthone (1 mg, 0.005 mmol, 5 mol%) as a photocatalyst, yielding a colorless oil (40.7 mg, 85%; *E/Z* 76:24).

R_f = 0.93 (*n*-pentane);**GC-EI-MS:** m/z: 423.15245 ($[M-C_4H_9]^+$, calcd. for $C_{20}H_{35}SiSn^+$: 423.15273); **^{1}H NMR** (600 MHz, $CDCl_3$) δ = 7.05 – 7.01 (m, 2H; H3), 6.82 – 6.78 (m, 2H; H4), 6.11 (s, 1H; H7), 2.31 (s, 3H; H1), 1.48 – 1.40 (m, 6H; H9), 1.33 – 1.22 (m, 6H; H10), 0.90 – 0.81 (m, 15H; H11,12), -0.15 (s, 9H; H8) ppm; **^{13}C NMR** (151 MHz, $CDCl_3$) δ = 168.9 (C6), 146.00 (C7), 145.6 (C2), 134.6 (C5), 128.5 (2C, C3), 126.1 (2C, C4), 29.1 (3C, C9), 27.4 (3C, C10), 21.2 (C1), 13.8 (3C, C12), 10.2 (3C, C11), 0.6 (3C, C8) ppm; **IR (ATR):** $\tilde{\upsilon}$ = 2955(m), 2924(m), 2871(w), 2854(w), 1608(w), 1546(w), 1500(w), 1463(w), (1405(w), 1245(m), 1178(w), 1073(w), 1020(w), 855(s), 747(s), 681(s) cm^{-1}.

2.3. Subsequent transformations of vinyl silanes

General Procedure F for Hiyama-Denmark cross coupling reactions of *Z*-15[16]

A flame-dried Schlenk-tube was charged with ***Z*-15** (0.12 mmol, 1.2 eq.) and dry THF (0.8 mL) under argon atmosphere. Tetrabutylammoniumfluoride trihydrate (3.0 eq.) was added portionwise and the mixture was stirred for 10 min at room temperature. The specific halide (1.0 eq.) and $Pd(dba)_2$ (0.05-0.75 eq.) were added and the mixture stirred at room temperature for 24 h. The crude reaction mixture was filtered through a plug of silica, diluted with EtOAc, concentrated *in vacuo* and purified by column chromatography (SiO_2, specified combination of solvents).

***trans*-D-Styrene (37)**

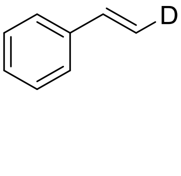

***E*-2** (17.6 mg, 0.1 mmol, 1.0 eq.) was weighed out into an oven-dried round-bottom flask. The flask was sealed and purged with argon before the addition of CD_2Cl_2 (1 mL) and DCl in D_2O (37% w/v, 32 µL, 0.3

mmol, 3 eq.). The reaction was stirred at room temperature for 1 h before analysis by ^{1}H NMR indicating deuterium incorporation in the designated position (*trans*) 62%.

cis-D-Styrene (38)

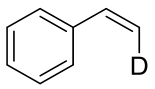

***Z*-2** (17.6 mg, 0.1 mmol, 1.0 eq.) was weighed out into an oven-dried round-bottom flask. The flask was sealed and purged with argon before the addition of CD_2Cl_2 (1 mL) and DCl in D_2O (37% w/v, 32 µL, 0.3 mmol, 3 eq.). The reaction was stirred at room temperature for 1 h before analysis by ^{1}H NMR indicating deuterium incorporation in the designated position (*cis*) 42%.

(*E*)-(1-Iodobut-1-en-2-yl)benzene (*Z*-16)

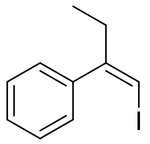

N-Iodosuccinimide (27.0 mg, 0.12 mmol, 1.2 eq.) was added to a solution of ***Z*-15** (21.6 mg, 0.1 mmol, 1.0 eq.) in MeCN (3 mL) and the mixture was stirred for 20 min at room temperature. After addition of water (3 mL) and CH_2Cl_2 (3mL) the organic phase was separated, the aqueous phase was extracted with CH_2Cl_2 (3x 5 mL). The combined organic phases were washed with $Na_2S_2O_3$-solution (aq., sat.), dried over $MgSO_4$ and concentrated *in vacuo*. Purification by column chromatography (SiO_2, 100% CH) yielded a colorless oil (21.2 mg, 82%); analytical data in agreement with the literature.[3]

^{1}H NMR (400 MHz, $CDCl_3$) δ = 7.43 – 7.28 (m, 3H), 7.22 – 7.13 (m, 2H), 6.27 (t, *J* = 1.7 Hz, 1H), 2.52 (dq, *J* = 7.3, 1.2 Hz, 2H), 1.01 (t, *J* = 7.4 Hz, 3H) ppm.

(*Z*)-Hepta-3,6-dien-3-ylbenzene (*Z*-17)[17]

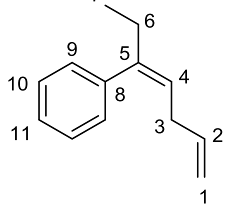

An oven-dried Schlenk-flask was charged with ***Z*-14** (28.0 mg, 0.1 mmol, 1.0 eq.) and dry THF (1mL) under argon atmosphere. TBAF (1.0 M in THF, 0.62 mL, 0.62 mmol, 6.2 eq.) was added, followed by allyl acetate (86.3 µL, 0.8 mmol, 8.0 eq.) and $Pd_2dba_3 \cdot CHCl_3$ (10.4 mg, 0.01 mmol, 0.1 eq). The mixture was stirred for 16 h at room temperature, filtered through a short plug of silica and concentrated *in vacuo*. The crude product was purified by column chromatography (SiO_2, 100% CH) to yield a colorless oil (10.0 mg, 58%).

R_f = 0.74 (CH); **GC-EI-MS**: m/z: 172.12468 ($[M]^+$, calcd. for $C_{13}H_{16}^{+}$: 172.12465); **^{1}H NMR** (600 MHz, CD_2Cl_2) δ = 7.39 – 7.31 (m, 2H; H10), 7.27 – 7.22 (m, 1H; H11), 7.20 – 7.11 (m, 2H; H9), 5.84 (ddt, *J* = 17.1, 10.1, 6.1 Hz, 1H; H2), 5.50 (tt, *J* = 7.5, 1.4 Hz, 1H; H4), 5.07 – 4.94 (m, 2H; H1), 2.69 (ddt, *J* = 7.7, 6.3, 1.3 Hz, 2H; H3), 2.40 (qq, *J* = 7.4, 1.1 Hz, 2H; H6), 1.00 (t, *J* = 7.4 Hz, 3H; H7) ppm; **^{13}C NMR** (151 MHz, CD_2Cl_2) δ = 143.9 (C5), 141.1 (C8), 137.7 (C4), 128.2 (2C, C9), 127.9 (2C, C10), 126.4 (C11), 122.6 (C2), 114.0 (C1), 33.1 (C3), 32.0 (C6), 12.8 (C7) ppm; **IR (ATR):** $\tilde{\upsilon}$ = 3079(w), 2966(w), 2930(w), 1947(w), 1636(w), 1493(w), 1460(w), 1441(w), 1368 (w), 991 (w), 909 (m), 766(m), 699(s) cm^{-1}.

(*Z*)-1-Nitro-4-(2-phenylbut-1-en-1-yl)benzene (*Z*-18)

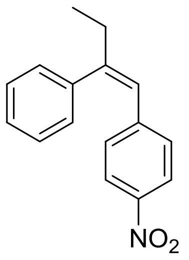

According to General Procedure F, ***Z*-15** (26.0 mg, 0.12 mmol, 1.2 eq.) and 1-iodo-4-nitrobenzene (24.9 mg, 0.1 mmol, 1.0 eq.) were converted to ***Z*-18** using 7.5 mol% of $Pd(dba)_2$. Purification by column chromatopgraphy (SiO_2, EtOAc/CH 1:40) yielded a yellow solid (12.7 mg, 50%); analytical data in agreement with the literature.[18]

^{1}H NMR (400 MHz, $CDCl_3$) δ = 8.00 – 7.83 (m, 2H), 7.31 (m, 3H), 7.11 (m, 2H), 7.06 – 6.99 (m, 2H), 6.47 (s, 1H), 2.56 (qd, *J* = 7.4, 1.5 Hz, 2H), 1.09 (t, *J* = 7.5 Hz) ppm.

(*E*)-(1-Iodoprop-1-en-2-yl)benzene (39)[19]

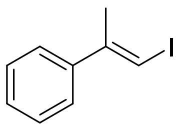

An oven-dried Schlenk-flask was charged with Cp_2ZrCl_2 (1462 mg, 5.0 mmol, 1.0 eq.) and dry CH_2Cl_2 under argon atmosphere. Trimethylaluminum (2.0 M in toluene, 5.0 mL, 10.0 mmol, 2.0 eq.) was added at room temperature to give a yellow solution, followed by phenylacetylene (0.55 mL, 5.0 mmol, 1.0 eq.) and the mixture was stirred overnight. A solution of iodine (1904 mg, 7.5 mmol, 1.2 eq.) in dry THF (5mL) was added at 0°C slowly over 10 min before the mixture was stirred at room temperature for 2 h until the brown solution turned yellow. The reaction was quenched carefully with H_2O/Et_2O (1:1, 20 mL) at 0°C, the organic phase was separated, washed with $Na_2S_2O_3$-solution (aq., sat., 2x 20 mL), dried over MgSO4 and concentrated in vacuo. Purification by column chromatography (SiO_2, 100% CH) yielded a colorless oil (349 mg, 29%); analytical data in agreement with the literature.[20]

^{1}H NMR (400 MHz, $CDCl_3$) δ = 7.39 – 7.28 (m, 5H), 6.52 (q, *J* = 1.1 Hz, 1H), 2.29 (d, *J* = 1.1 Hz, 3H) ppm.

((1*E*,3*Z*)-2-Methylhexa-1,3-diene-1,4-diyl)dibenzene (*Z*-19)

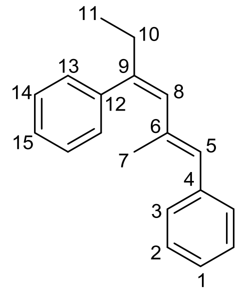

According to General Procedure F, ***Z*-15** (26.0 mg, 0.12 mmol, 1.2 eq.) and **39** (24.4 mg, 0.1 mmol, 1.0 eq.) were converted to ***Z*-19** using 5 mol% of $Pd(dba)_2$. Purification by column chromatography (SiO_2, 100% CH) yielded a colorless oil (14.7 mg, 59%).

R_f = 0.37 (CH); **GC-EI-MS**: m/z: 248.15589 ($[M]^+$, calcd. for $C_{19}H_{20}^+$: 248.15595); **^{1}H NMR** (600 MHz, $CDCl_3$) δ = 7.38 – 7.33 (m, 2H, C13), 7.32 – 7.29 (m, 2H, C3), 7.29 – 7.27 (m, 1H, C15), 7.27 – 7.23 (m, 4H, C2, C14), 7.17 (ddt, *J* = 8.0, 6.6, 1.3 Hz, 1H, C1), 6.51 (dt, *J* = 11.3, 1.3 Hz, 1H; H8), 6.46 (dq, *J* = 11.3, 1.3 Hz, 1H; H5), 2.54 (qd, *J* = 7.4, 1.5 Hz, 2H; H10), 2.23 (d, *J* = 1.3 Hz, 3H; H7), 1.06 (t, *J* = 7.4 Hz, 3H; H11) ppm; **^{13}C NMR** (151 MHz, $CDCl_3$) δ = 146.2 (C9), 143.7 (C4), 141.1 (C12), 135.0 (C6), 128.9 (2C, C2/C14), 128.3 (2C, C2/C14), 128.2 (2C, C13), 127.0 (C15), 126.8 (C1), 125.7 (2C, C3), 124.6(C5), 122.4 (C8), 32.6 (C10), 16.1 (C7), 13.5 (C11) ppm; **IR (ATR):** ῦ = 3055(w), 3027(w), 2963(w), 2927(w), 2871(w), 1594(w), 1492(m), 1441(w), 1378(w), 1257(w), 1077(w), 1025(w), 892(w), 876(m), 842(w), 756(s), 693(s) cm^{-1}.

(*Z*)-1-Methoxy-4-(2-phenylbut-1-en-1-yl)benzene (*Z*-21)

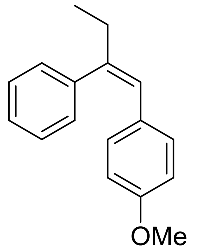

A flame-dried Schlenk-tube was charged with ***Z*-21** (20.6 mg, 0.1 mmol, 1.0 eq.) and dry THF (0.5 mL). TBAF (1.0 M in THF, 0.2 mL, 0.2 mmol, 2.0 eq.) was added slowly at room temperature and the mixture was stirred for 15 min. 4-Iodoanisole (23.4 mg, 0.1 mmol, 1.0 eq.) and $Pd(dba)_2$ (2.8 mg, 0.005 mmol, 0.05 eq.) were sequentially added before the reaction was stirred at room temperature overnight. After filtration through a pad of silica and dilution with EtOAc, the crude reaction mixture was concentrated *in vacuo.* Purification by column chromatography (CH) yielded a colorless oil (19.1 mg, 80%); analytical data in agreement with the literature.[18]

^{1}**H NMR** (300 MHz, $CDCl_3$) δ = 7.38 – 7.25 (m, 3H, H), 7.24 – 7.14 (m, 2H), 6.91 – 6.84 (m, 2H), 6.72 – 6.59 (m, 2H), 6.40 (s, 1H), 3.75 (s, 3H), 2.52 (qd, *J* = 7.4, 1.4 Hz, 2H), 1.09 (t, *J* = 7.4 Hz, 3H) ppm.

(*Z*)-(2-(4-Fluorophenyl)-2-phenylvinyl)trimethylsilane (*Z*-28)[1]

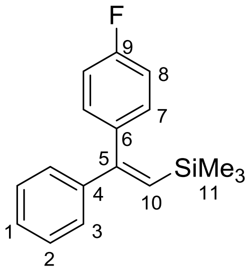

To an oven-dried Schlenk tube was added $Pd(OAc)_2$ (3 mg, 0.01 mmol, 0.05 eq.), SPhos (10 mg, 0.02 mmol, 0.1 eq.), 1-bromo-4-fluorobenzene (46.4 mg, 0.26 mmol, 1.05 eq.), **Z-23** (75.5 mg, 0.25 mmol, 1.0 eq.), and K_3PO_4 (160 mg, 0.75 mmol, 3.0 eq.). The tube was sealed and purged with argon before the addition of 1,4-dioxane (1.0 mL, 0.25 M) and water (22.5 µL, 1.25 mmol, 5.0 eq.). The reaction mixture was stirred at 80 °C for 4 h. After the reaction was complete, the tube was gradually cooled to rt and the reaction mixture was diluted with EtOAc (5 mL) and filtered through a layer of Celite eluting the product with EtOAc (2x 10 mL). The filtrate was washed with water (25 mL), brine (25 mL), dried over MgSO4, and concentrated under reduced pressure. The crude residue was purified by column chromatography (SiO_2, 100 % CH) to yield ***Z*-28** as a clear oil (60.2 mg, 89%).

$\boldsymbol{R_f}$ = 0.78 (*n*-pentane); **HR-ESI-MS**: m/z: 377.02906 ([M+Ag]$^+$, calcd. for $C_{17}H_{19}FSiAg^+$: 377.02855); ^{1}**H NMR** (600 MHz, $CDCl_3$) δ = 7.28 – 7.23 (m, 5H; H1,2,3), 7.20 – 7.14 (m, 2H; H7), 7.07 – 7.01 (m, 2H; H8), 6.29 (s, 1H; H10), -0.10 (s, 9H; H11) ppm; ^{13}**C NMR** (151 MHz, $CDCl_3$) δ = 162.46 (d, J_{CF} = 246.0 Hz; C9), 156.1 (C5), 143.3 (C4), 138.73 (d, J_{CF} = 3.5 Hz; C6), 131.42 (d, J_{CF} = 7.9 Hz; 2C, C7), 130.45 (d, J_{CF} = 0.8 Hz; C10), 128.2 (2C, C2), 127.9 (C1), 127.3 (2C, C3), 115.0 (d, J_{CF} = 21.2 Hz; 2C, C8), 0.1 (3C, C11) ppm; ^{19}**F NMR** (564 MHz, $CDCl_3$) δ = -115.00 ppm. **IR (ATR):** $\tilde{\upsilon}$ = 2954(w), 1601(w), 1567(w), 1505(s), 1491(w), 1444(w), 1404(w), 1335(w), 1247(s), 1222(s), 1157 (s), 1092(w), 1031(w), 1015(w), 907(w), 859(s), 831(s), 812(s), 762(s), 692(s) cm^{-1}.

(*E*)-(2-(4-Fluorophenyl)-2-phenylvinyl)trimethylsilane (*E*-28)[1]

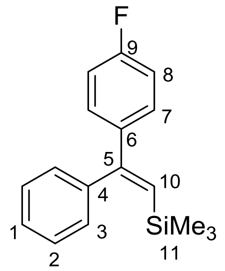

To an oven-dried Schlenk tube was added $Pd(OAc)_2$ (3 mg, 0.01 mmol, 0.05 eq.), SPhos (10 mg, 0.02 mmol, 0.1 eq.), 1-bromo-4-fluorobenzene (46.4 mg, 0.26 mmol, 1.05 eq.), ***E*-23** (75.5 mg, 0.25 mmol, 1.0 eq.), and K_3PO_4 (160 mg, 0.75 mmol, 3.0 eq.). The tube was sealed and purged with argon before the addition of 1,4-dioxane (1.0 mL, 0.25 M) and water (22.5 µL, 1.25 mmol, 5.0 eq.). The reaction mixture was stirred at 80 °C for 4 h. After the reaction was complete, the tube was gradually cooled to room temperature and the reaction mixture was diluted with EtOAc (5 mL) and filtered through a layer of Celite eluting the product with EtOAc (2x 10 mL). The filtrate was washed with water (25 mL), brine (25 mL), dried over $MgSO_4$, and concentrated under reduced pressure. The crude residue was purified by column chromatography (SiO_2, 100 % CH) to yield ***E*-28** as a clear oil, containing 8% of ***Z-28*** (45.2 mg, 67%).

R_f = 0.78 (*n*-pentane); **HR-ESI-MS**: m/z: 377.02921 ($[M+Ag]^+$, calcd. for $C_{17}H_{19}FSiAg^+$: 377.02855); **^{1}H NMR** (600 MHz, $CDCl_3$) δ = 7.36 – 7.33 (m, 3H; H1,2), 7.27 – 7.23 (m, 2H; H3), 7.20 – 7.17 (m, 2H; H7), 6.97 – 6.92 (m, 2H; H8), 6.22 (s, 1H; H10), -0.11 (s, 9H; H11) ppm; **^{13}C NMR** (151 MHz, $CDCl_3$) δ = 162.6 (d, J_{CF} = 247.1 Hz; C9), 156.1 (C5), 142.6 (C4), 139.6 (d, J_{CF} = 3.2 Hz; C6), 129.8 (2C, 3C), 129.7 (d, J_{CF} = 1.7 Hz; C10), 129.0 (d, J_{CF} = 8.1 Hz; 2C, C7), 128.1 (2C, C2), 127.6 (C1), 114.9 (d, J_{CF} = 21.4 Hz; 2C, C8), 0.1 (3C, C11). ppm; **^{19}F NMR** (564 MHz, $CDCl_3$) δ = -115.05 ppm; **IR (ATR):** ῦ = 2953(w), 1602(w), 1506(s), 1491(w), 1443(w), 1406(w), 1334(w), 1247(m), 1234(m), 1158(m), 1014(w), 904(w), 860(m), 831(s), 702(s) cm^{-1}.

(*Z*)-5-(2-(4-Fluorophenyl)-2-phenylvinyl)-1-(phenylsulfonyl)-1*H*-indole (*Z*-29)

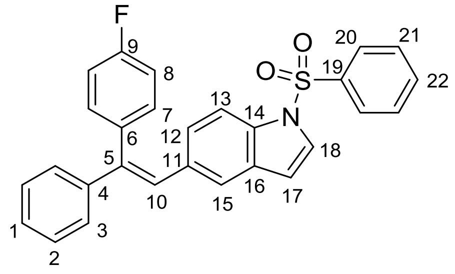

To a solution of ***Z*-28** (67.7 mg, 0.25 mmol, 1.0 eq.) in MeCN (7.5 mL) was added *N*-iodosuccinimide (70.3 mg, 0.31 mmol, 1.2 eq.). The reaction was stirred at room temperature for 16 h. The reaction mixture was diluted with DCM (20 mL) and quenched with aq. sat. sodium thiosulphate (50 mL). Organics were separated, washed with brine (50 mL), dried over $MgSO_4$, and concentrated under reduced pressure. The residue was transferred to an oven dried Schlenk flask

before the addition of $Pd(OAc)_2$ (3 mg, 0.01 mmol, 0.05 eq.), SPhos (10 mg, 0.02 mmol, 0.1 eq.), (1-(phenylsulfonyl)-1*H*-indol-5-yl)boronic acid, pinacol ester (100.6 mg, 0.26 mmol, 1.05 eq.), and K_3PO_4 (160 mg, 0.75 mmol, 3.0 eq.). The tube was sealed and purged with argon before the addition of 1,4-dioxane (1.0 mL, 0.25 M) and water (22.5 µL, 1.25 mmol, 5.0 eq.). The reaction mixture was stirred at 80 °C for 4 h. After the reaction was complete, the tube was gradually cooled to room temperature and the reaction mixture was diluted with EtOAc (5 mL) and filtered through a layer of Celite eluting the product with EtOAc (2x 10 mL). The filtrate was washed with water (25 mL), brine (25 mL), dried over $MgSO_4$, and concentrated under reduced pressure. The crude residue was purified by column chromatography (SiO_2, 0–15%) to yield ***Z*-29** as a white solid (87.3 mg, 77%).

R_f = 0.29 (EtOAc/CH, 1:9); **M.p.** 143.7 – 145.6 ˚C; **HR-ESI-MS**: m/z: 476.1102 ($[M+Na]^+$, calcd. for $C_{28}H_{20}NO_2FSNa^+$: 476.1091); **^{1}H NMR** (600 MHz, $CDCl_3$) δ = 7.90 – 7.85 (m, 2H; H20), 7.78 (dt, *J* = 8.6, 0.8 Hz, 1H; H13), 7.56 – 7.53 (m, 1H; H22), 7.52 (d, *J* = 3.7 Hz, 1H; H18), 7.46 – 7.42 (m, 2H; H21), 7.36 – 7.28 (m, 5H; H1,2,3), 7.23 (dt, *J* = 1.6, 0.7 Hz, 1H; H15), 7.19 – 7.14 (m, 2H; H7), 7.04 – 6.99 (m, 4H; H8,10,12), 6.52 (dd, *J* = 3.7, 0.8 Hz, 1H; H17) ppm; **^{13}C NMR** (151 MHz, $CDCl_3$) δ = 162.3 (d, J_{CF} = 246.8 Hz; C9), 143.4 (C4), 141.2 (C11), 138.3 (C19), 136.3 (d, J_{CF} = 3.5 Hz; C6), 133.9 (C22), 133.7 (C14), 132.9 (C5), 132.3 (d, J_{CF} = 7.9 Hz; 2C, C7), 130.8 (C16), 129.4 (2C, C21), 128.5 (C10), 128.4 (2C, C2), 127.7 (C1), 127.7 (2C, C3), 126.9 (2C, C20), 126.7 (C18), 126.6 (C12), 122.5 (C15), 115.8 (d, J_{CF} = 21.2 Hz; 2C, C8), 113.1 (C13), 109.5 (C17) ppm; **^{19}F NMR** (564 MHz, $CDCl_3$) δ = -115.00 ppm; **IR (ATR):** $\tilde{\upsilon}$ = 3028(w), 2235(w), 2165(w), 2051(w), 2018(w), 1599(w), 1506(m), 1449(m), 1371(s), 1273(m), 1222(s), 1190(s), 1154(m), 1132(m), 1116(s), 1092(m), 988(w), 881(w), 841(w), 811(m), 760(s), 683(s) cm^{-1}.

(*E*)-5-(2-(4-Fluorophenyl)-2-phenylvinyl)-1-(phenylsulfonyl)-1*H*-indole (*E*-29)

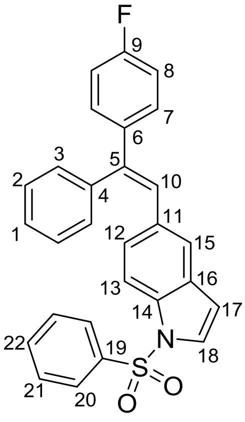

To a solution of ***E*-28** (67.7 mg, 0.25 mmol, 1.0 eq.) in MeCN (7.5 mL) was added *N*-iodosuccinimide (70.3 mg, 0.31 mmol, 1.2 eq.). The reaction was stirred at room temperature for 16 h. The reaction mixture was diluted with DCM (20 mL) and quenched with aq. sat. sodium thiosulphate (50 mL). Organics were separated, washed with brine (50 mL), dried over $MgSO_4$, and concentrated under reduced pressure. The residue was transferred to an oven dried Schlenk flask before the addition of $Pd(OAc)_2$ (3 mg, 0.01 mmol, 0.05 eq.), SPhos (10 mg, 0.02 mmol, 0.1 eq.), (1-(phenylsulfonyl)-1*H*-indol-5-yl)boronic acid, pinacol ester (100.6 mg, 0.26 mmol, 1.05 eq.), and K_3PO_4 (160 mg, 0.75 mmol, 3.0 eq.). The tube was sealed and purged with argon before the addition of 1,4-dioxane (1.0 mL, 0.25 M) and water (22.5 µL, 1.25 mmol, 5.0 eq.). The reaction mixture was stirred at 80 °C for 4 h. After the reaction was complete, the tube was gradually cooled to room temperature and the reaction mixture was diluted with EtOAc (5 mL) and filtered through a layer of Celite eluting the product with EtOAc (2x 10 mL). The filtrate was washed with water (25 mL), brine (25 mL), dried over $MgSO_4$, and concentrated under reduced pressure. The crude residue was purified by column chromatography (SiO_2, 0 – 15 %) to yield ***E*-27** as a white solid, containing 8% ***Z*-29** (72.9 mg, 64%).

R_f = 0.29 (EtOAc/CH, 1:9); **M.p.** 145.2 – 147.6 ˚C; **HR-ESI-MS**: m/z: 476.1081 ([M+Na]$^+$, calcd. for $C_{28}H_{20}NO_2FSNa^+$: 476.1091); **^{1}H NMR** (600 MHz, $CDCl_3$) δ = 7.86 – 7.82 (m, 2H; H20), 7.72 (dd, *J* = 8.7, 0.8 Hz, 1H; H13), 7.54 (dd, *J* = 7.9, 7.1 Hz, 1H; H22), 7.48 (d, *J* = 3.7 Hz, 1H; H12), 7.45 – 7.41 (m, 2H; H2), 7.34 – 7.29 (m, 3H; H1,3), 7.29 – 7.25 (m, 2H; H7), 7.18 (dd, *J* = 1.6, 0.8 Hz, 1H; H15), 7.18 – 7.15 (m, 2H; H21), 7.01 – 6.96 (m, 3H; H8,18), 6.94 (s, 1H; H10), 6.48 (dd, *J* = 3.7, 0.8 Hz, 1H; H17). ppm; **^{13}C NMR** (151 MHz, $CDCl_3$) δ = 162.50 (d, J_{CF} = 247.1 Hz; C9), 141.2 (C4), 140.3 (C11), 139.7 (d, J_{CF} = 3.2 Hz; C6), 138.4 (C19), 133.9 (C22), 133.7 (C14), 132.9 (C5), 130.8 (C16), 130.5 (2C, C21), 129.4 (2C, C2), 129.3 (d, J_{CF} = 7.9 Hz; 2C, C7), 128.9 (2C, C3), 127.9 (C10), 127.7 (C1), 126.9 (2C, C20), 126.7 (C12), 126.6 (C18), 122.5 (C15), 115.2 (d, J_{CF} = 21.4 Hz; 2C, C8), 113.1 (C13), 109.5 (C17) ppm; **^{19}F NMR** (564 MHz, $CDCl_3$) δ = -115.00 ppm; **IR (ATR):** $\tilde{\upsilon}$ = 3134(w), 3100(w), 2242(w), 2108(w), 2032(w), 1598(w), 1505(s), 1450(s), 1373(s), 1275(m), 1220(s), 1191(s), 1174(s), 1134(s), 1119(s), 989(s), 895(m), 800(s), 767(s), 700(s), 684(s) cm^{-1}.

3. Reaction Progress Monitoring

The isomerisstic catation of ***E*-1** was performed on a 0.2 mmol scale according to General Proedure D. The conversion was monitored by HPLC analysis (CHIRACEL OJ-H column, *n*-hexane/*i*-PrOH 99:1, 0.5 mL/min) of 20 µL samples taken from the same reaction solution after the specified time with the *Z*-isomer ***Z*-1** eluting at 6.44 min and the *E*-isomer ***E*-1** at 7.20 min.

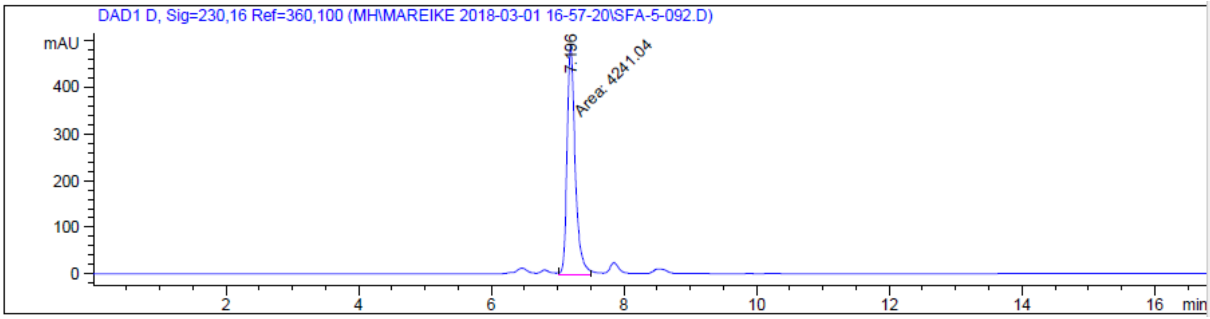

Signal 4: DAD1 D, Sig=230,16 Ref=360,100

Peak #	RetTime [min]	Type	Width [min]	Area [mAU*s]	Height [mAU]	Area %
1	7.196	MM	0.1431	4241.04346	494.05954	100.0000

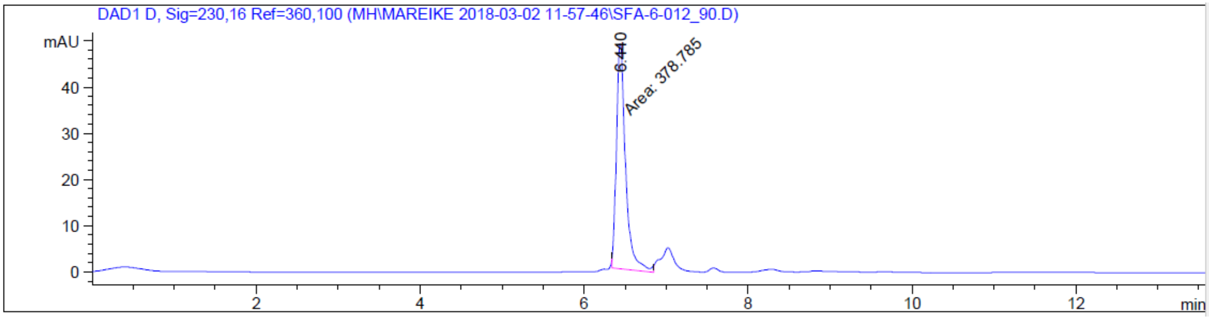

Signal 4: DAD1 D, Sig=230,16 Ref=360,100

Peak #	RetTime [min]	Type	Width [min]	Area [mAU*s]	Height [mAU]	Area %
1	6.440	MM	0.1294	378.78513	48.77850	100.0000

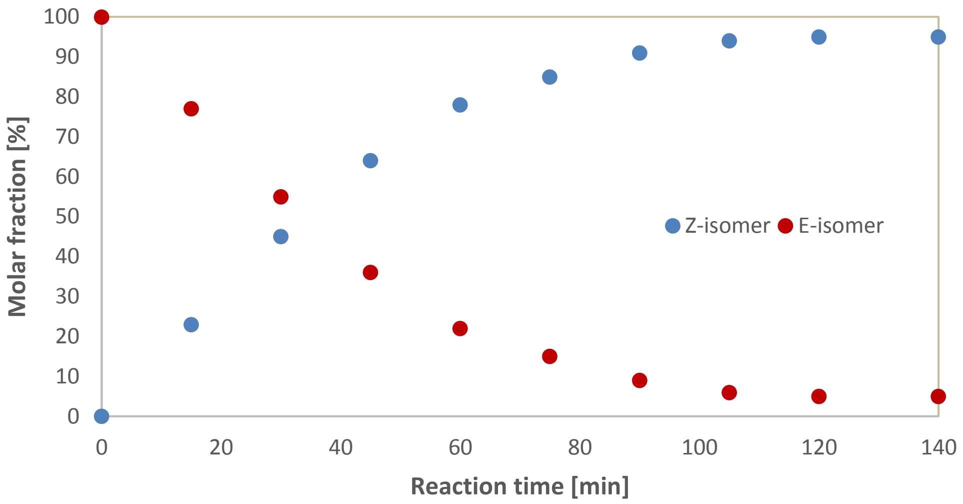

Figure S4: Reaction progress monitoring of the isomerisation by HPLC analysis.

4. DFT Calculations

Method

All structures were optimised without geometry constraints using the TPSS functional[21] and an atom-pairwise dispersion correction (D3)[22]. A flexible triple zeta basis set (def2-TZVP)[23] was used in all calculations. For the calculation of the free enthalpy contributions (G^{RRHO}(298K)), a rotor approximation was applied for vibrational modes with wave numbers below 100 cm^{-1}.[24] The nature of all optimised stationary points was proven by the presence of either 0 (minimum) or 1 (transition structure) imaginary vibrational frequency.

Electronic energies were recalculated with the double hybrid functional PWPB95(-D3)[25] using the structures optimised with TPSS-D3. In PWPB95, a component of the correlation energy is computed by perturbation theory, it performs more accurately in the determination of energies, even for open shell molecules such as radicals or triplet states. The final value for the free enthalpy ΔG(298) was obtained using the PWPB95-D3 electronic energies and G^{RRHO}(298K), obtained with TPSS-D3.

Using the TPSS-D3 S_0 geometries, singlet and triplet excitation energies were calculated with TD-DFT using the B-LYP functional[26]. Energies and spin densities of the T_1 states were calculated with PWPB95-D3/def2-TZVP.

All geometry optimisations and vibrational frequency calculations were performed with the TURBOMOLE 7.3 program.[27] TDDFT and PWPB95-D3 single point calculations were performed with the ORCA (4.0.2) program.[28]

Results

Table S1. DFT-calculated electronic energies after geometry optimisation with TPSS-D3. Single point electronic energies obtained with PWPB95-D3. Free energy corrections G^{RRHO}_{298} are based on harmonic vibrational frequencies obtained with TPSS-D3. The def2-TZVP basis set was used in all calculations.

	E (TPSS-D3) [E_h]	E(PWPB95-D3) [E_h]	G^{RRHO}_{298} [kcal/mol]	E^{T1}(PWPB95-D3)[a] [E_h]
**(*E*)-2**	-718.629088	-718.143126	119.601	-718.019152
**(*Z*)-2**	-718.624427	-718.138517	120.173	-718.005154
**^{3}2**	-718.551052	-718.054075	117.348	--
**(*E*)-1**	-797.304040	-796.737466	153.003	-796.603552
**(*Z*)-1**	-797.303360	-796.737946	153.106	-796.578309
**^{3}1**	-797.229897	-796.651667	150.685	--

[a] Single point energy of the T_1 state using the S_0 geometry

Table S2. TDDFT-calculated vertical excitation energies (BLYP/def2-TZVP) after geometry optimisation with TPSS-D3. Excitation energies in Hartree and kcal/mol (in brackets).

	E (S_0->S_1) [E_h] ([kcal/mol])	E (S_0->T_1) [E_h] ([kcal/mol])
**(*E*)-2**	0.160228 (100.5)	0.111289 (69.8)
**(*Z*)-2**	0.163078 (102.3)	0.117939 (74.0)
**(*E*)-1**	0.162859 (102.2)	0.118287 (74.2)
**(*Z*)-1**	0.169869 (106.6)	0.138840 (87.1)

Figure S5 Structures of (*E*)/(*Z*) isomers and triplet ground state of **2**. In square brackets: relative free energies (ΔG(298)) in kcal/mol. In curly brackets: vertical (single point) energy of the triplet state.

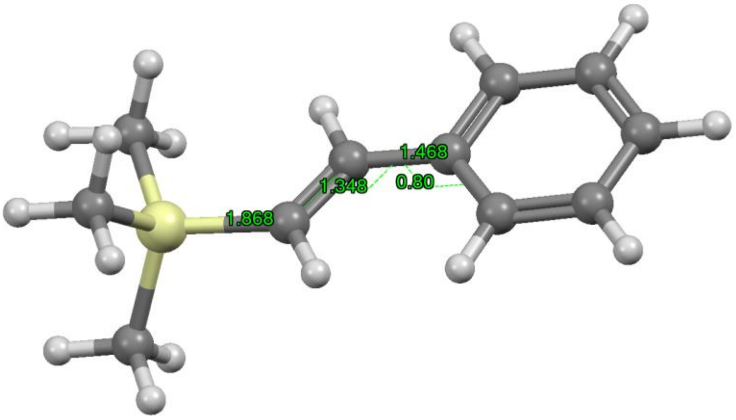

(*E*)-**2** [0.0] {+77.8}

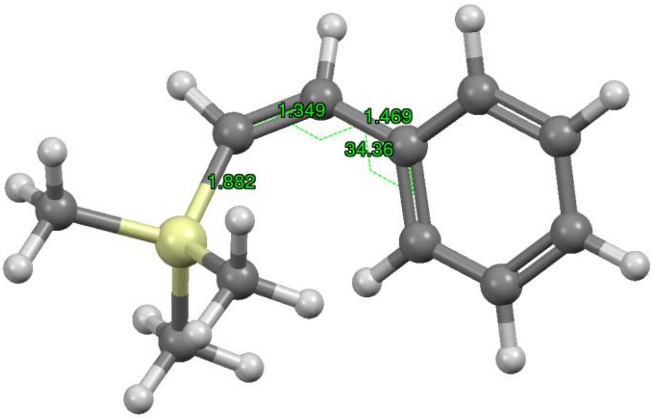

(*Z*)-**2** [+3.5] {+83.7}

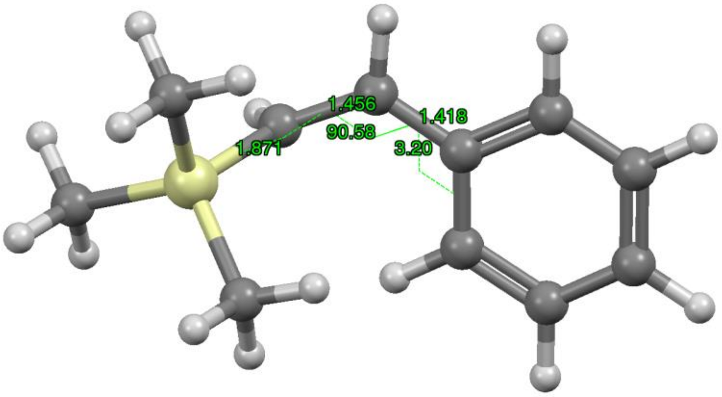

^{3}**2** [+53.5]

Figure S6 Structures of (*E*)/(*Z*) isomers and triplet ground state of **1**. In square brackets: relative free energies (ΔG(298)) in kcal/mol. In curly brackets: vertical (single point) energy of the triplet state.

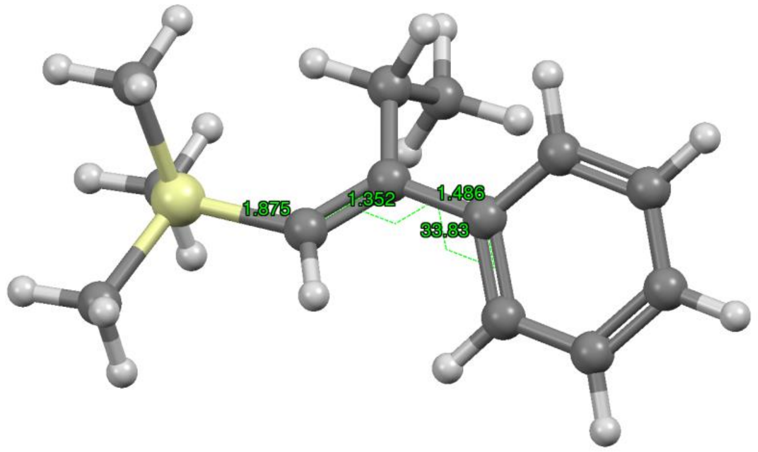

(*E*)-**1** [0.0] {+84.0}

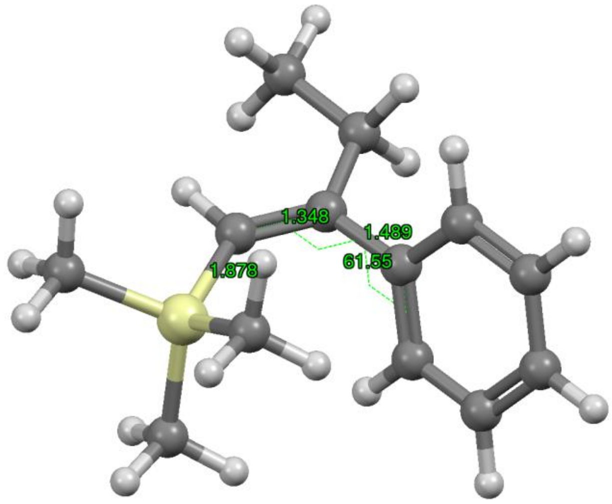

(*Z*)-**1** [-0.2] {+100.2}

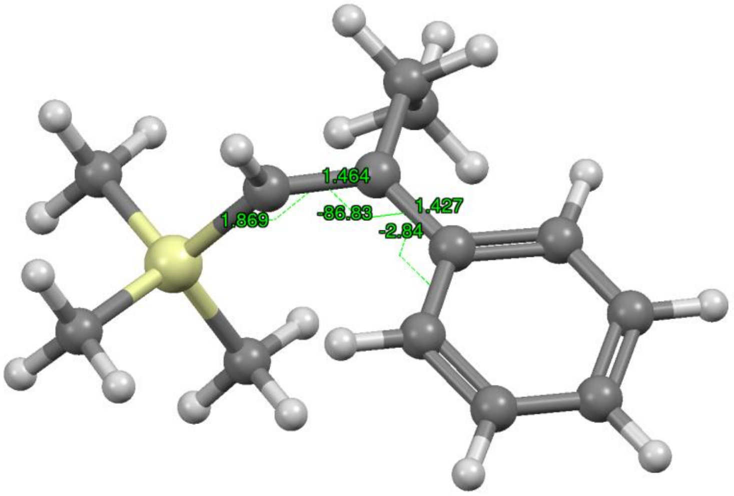

^{3}1 [+51.6]

Orbital analysis of the triplet excitation of (*E*)- and (*Z*)-1

In both ground (S_0) states of (*E*)-**1** and (*Z*)-**1**, HOMO and LUMO (MOs 55 and 56) are constituted mainly by the π/π* orbitals of the ethene bond, with significant admixture of phenyl π orbitals.

The (forbidden) first triplet excitation to T_1 of (*E*)-**1** corresponds to a HOMO-LUMO excitation. The two singly occupied orbitals of T_1 (MOs 55a and 56a) are both delocalised over the styryl system and lead in total to a localisation of spin density in the vinyl group, allowing the triplet state to attain the energy minimum by rotation around the vinyl C-C bond.

The more distorted styryl moeity of (*Z*)-**1**, however, shows a significantly higher energy of the forbidden first vertical triplet excitation (87 kcal/mol) in the TDDFT (B-LYP) calculation and of the energy of the (relaxed) T_1 wave function (100 kcal/mol with PWPB95-D3) at the same geometry. The larger torsional angle (61.6°) aggravates the mixing of ethene and phenyl π orbitals and leads to two rather localised α spin orbitals in the T_1 state (MOs 55a and 56a in Figure S6). Spin density is accumulated mainly in the phenyl ring, suggesting a lower tendency to rotate the vinyl C-C bond. Of course, thermal motion would allow the molecule to leave the S_0 minimum geometry and change the spin density distribution in the further course.

Figure S7 Molecular structure (TPSS-D3/def2-TZVP) and molecular orbitals (0.03 a.u.) of S_0 state of ***(E)*-1** (B-LYP/def2-TZVP)

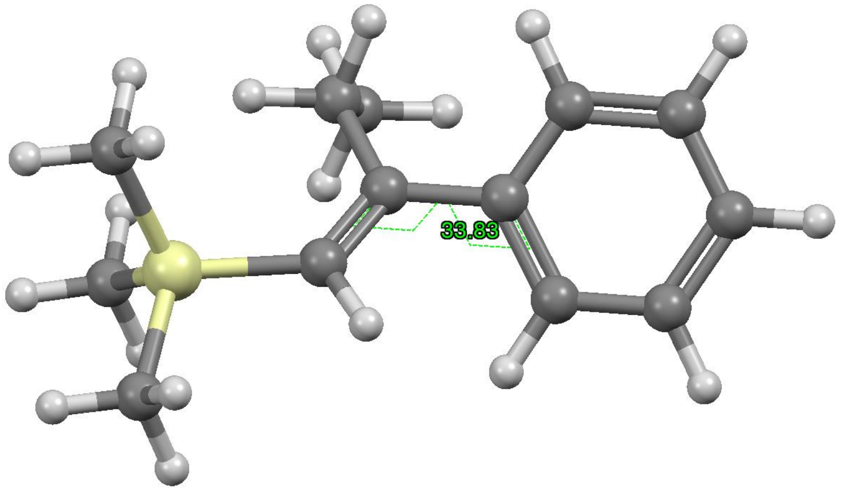

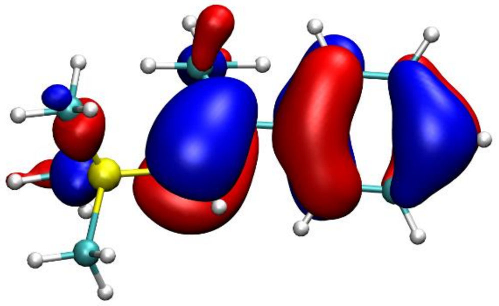

MO 55 (HOMO, occ = 2.0)

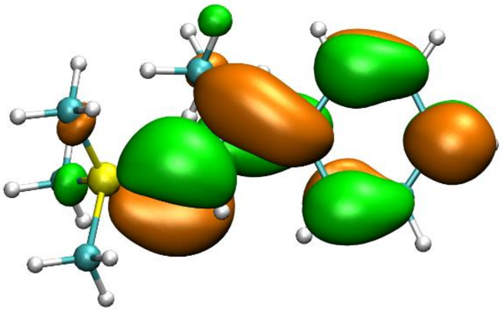

MO 56 (LUMO, occ = 0.0)

First singlet excitation (BLYP/def2-TZVP)

STATE 1: E= 0.162859 au 4.432 eV 35743.4 cm**-1

54a -> 56a : 0.400913 (c= 0.63317672)

55a -> 56a : 0.015279 (c= -0.12361016)

55a -> 57a : 0.578598 (c= -0.76065651)

First triplet excitation (BLYP/def2-TZVP)

STATE 1: E= 0.118287 au 3.219 eV 25961.0 cm**-1

55a -> 56a : 0.966618 (c= 0.98316730)

Figure S8 Molecular structure (TPSS-D3/def2-TZVP) and molecular orbitals (0.03 a.u.) of the S_0 state of **(Z)-1** (B-LYP/def2-TZVP)

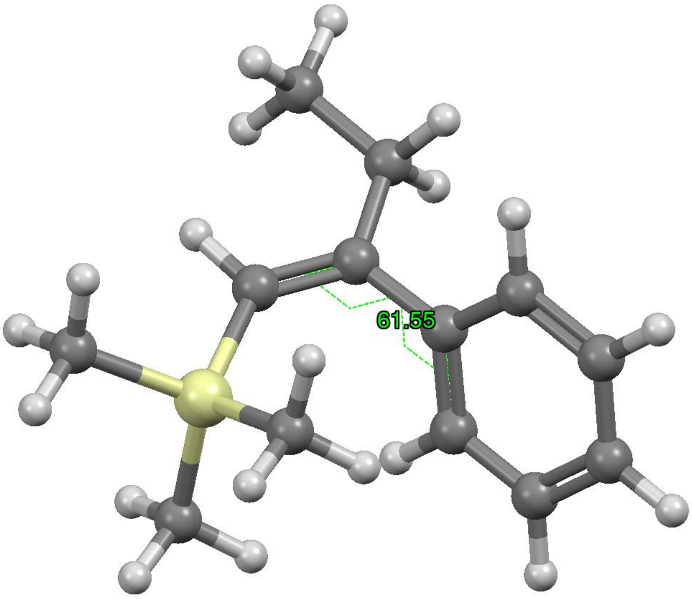

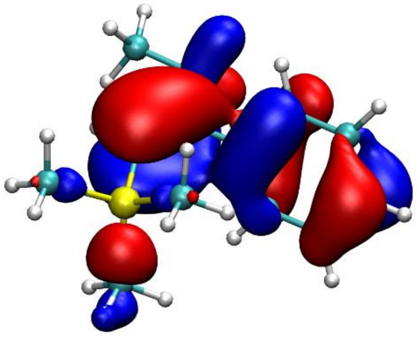

MO 55 (HOMO, occ = 2.0)

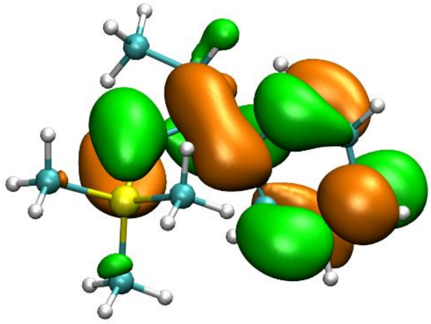

MO 56 (LUMO, occ = 0.0)

First singlet excitation (BLYP/def2-TZVP)

STATE 1: E= 0.169869 au 4.622 eV 37281.9 cm**-1

53a -> 56a : 0.037489 (c= 0.19362120)

53a -> 57a : 0.034189 (c= -0.18490384)

54a -> 56a : 0.078127 (c= -0.27951254)

55a -> 56a : 0.097241 (c= 0.31183559)

55a -> 57a : 0.729890 (c= -0.85433627)

First triplet excitation (BLYP/def2-TZVP)

STATE 1: E= 0.138840 au 3.778 eV 30471.8 cm**-1

53a -> 58a : 0.030353 (c= 0.17421990)

54a -> 57a : 0.025957 (c= -0.16111220)

55a -> 56a : 0.891880 (c= 0.94439419)

55a -> 58a : 0.022254 (c= 0.14917839)

Figure S9 Frontier orbitals of T_1 state of **(E)-1** at S_0 geometry (PWPB95/def2-TZVP)

α Orbitals (occ = 1.0)

β Orbitals (occ = 0.0)

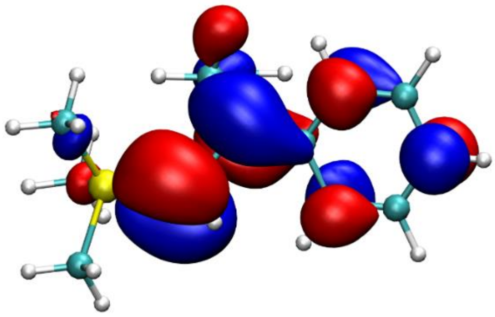

56a (α-HOMO)

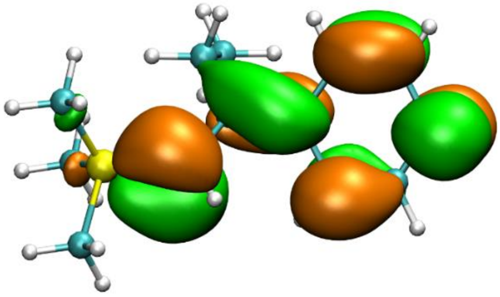

56b (β-LUMO+1)

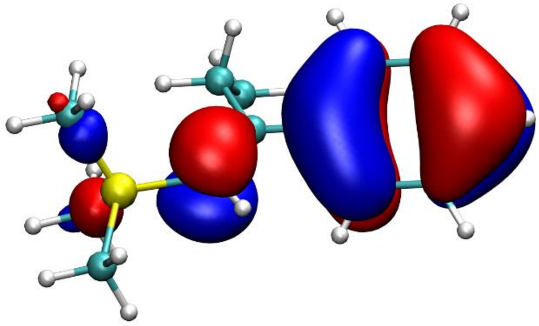

55a (α-HOMO-1)

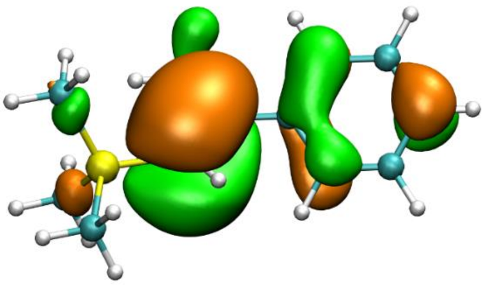

55b (β-LUMO)

Spin Density (0.005 a.u.)

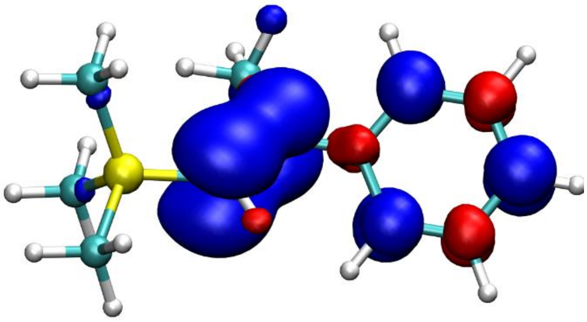

Figure S10 Frontier orbitals of T_1 state of ***(Z)*-1** at S_0 geometry (PWPB95/def2-TZVP)

α Orbitals (occ = 1.0)

β Orbitals (occ = 0.0)

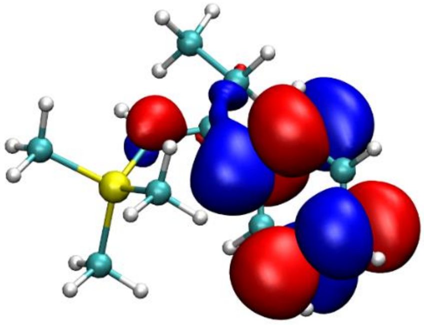

56a (α-HOMO)

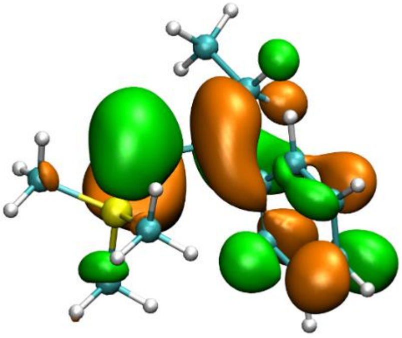

56b (β-LUMO+1)

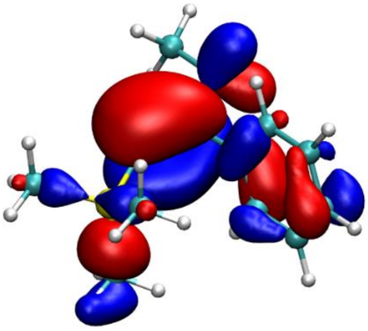

55a (α-HOMO-1)

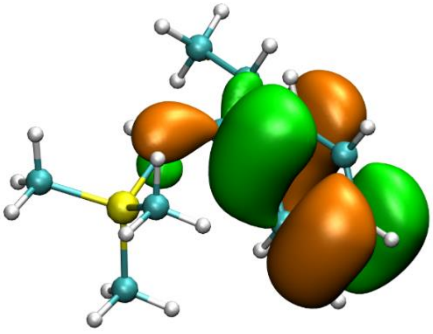

55a (β-LUMO)

Spin Density (0.005 a.u.)

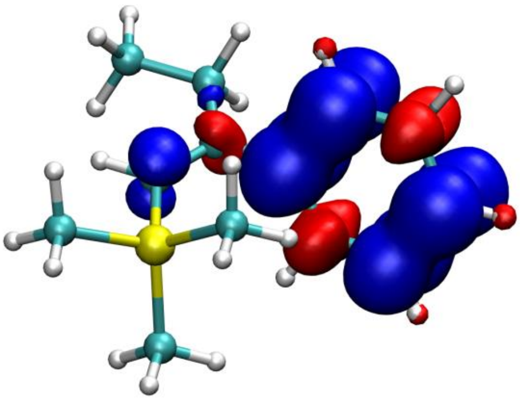

Figure S11 Spin Density of T_1 state of **(E)-2** and **(Z)-2** at S_0 geometry (PWPB95/def2-TZVP)

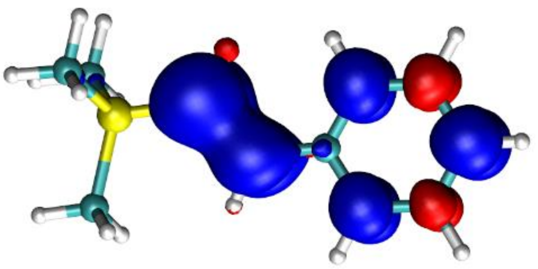

(E)-2

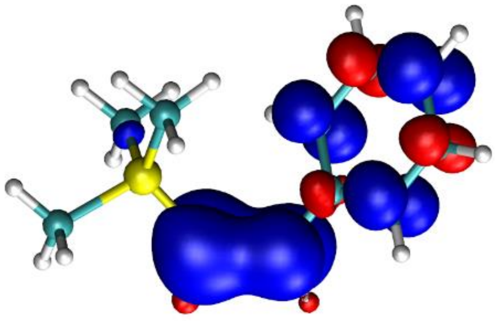

(Z)-2

Cartesian coordinates of optimised structures (TPSS-D3/def2-TZVP) discussed in this work

***E*-2**

E(TPSS-D3/def2-TZVP) = -718.6290880830 (conv)

Lowest Freq. = 19.27 cm^-1

28

E-1 (001/c1/tpss-d3.def2-TZVP)

C	-2.6750754	1.6029786	0.3908153
C	-1.3816445	1.8937613	0.1474433
H	-2.7932183	-3.0017988	-0.3932751
C	-3.3340588	-2.1002277	-0.1186000
C	-4.7018558	-2.1671320	0.1646680
H	-5.2249076	-3.1175739	0.1104617
C	-5.3880727	-1.0056053	0.5175147
H	-6.4507607	-1.0460132	0.7400263
C	-4.7101904	0.2096150	0.5855230
H	-5.2465798	1.1150094	0.8611341
C	-3.3360014	0.2949484	0.3032773
C	-2.6600088	-0.8865748	-0.0505110
H	-1.5972355	-0.8520558	-0.2724906
H	-3.3442919	2.4119367	0.6909974
Si	-0.6315846	3.5989134	0.2874623
H	-0.7252334	1.0719778	-0.1538765
C	-1.9466765	4.8317093	0.8348102
H	-2.7718459	4.8897533	0.1153548
H	-1.5152964	5.8361161	0.9235721
H	-2.3680931	4.5662581	1.8114213

C	0.7723181	3.5531834	1.5474240
H	1.2643025	4.5303430	1.6274377
H	1.5360772	2.8196885	1.2620697
H	0.4030113	3.2787797	2.5422019
C	0.0682195	4.1080213	-1.3893003
H	0.5509698	5.0915185	-1.3334589
H	-0.7207966	4.1620903	-2.1481142
H	0.8186998	3.3895295	-1.7403385

Z-2

E(TPSS-D3/def2-TZVP) = -718.6244265015 (conv)

Lowest Freq. = 29.78 cm^-1

28

Z-1 (002/c1/tpss-d3.def2-TZVP)

C	-2.5892245	1.4641779	0.5310942
C	-1.2515435	1.5582474	0.3852477
H	-3.3749904	-3.0606103	1.3316626
C	-3.7499754	-2.1200034	0.9375128
C	-5.0338421	-2.0522478	0.3932881
H	-5.6578841	-2.9403908	0.3526441
C	-5.5187771	-0.8309376	-0.0786428
H	-6.5216658	-0.7668621	-0.4916686
C	-4.7216195	0.3088390	-0.0151503
H	-5.1035650	1.2590645	-0.3810533
C	-3.4141189	0.2492165	0.4973844
C	-2.9517482	-0.9807147	0.9914162
H	-1.9685824	-1.0284522	1.4481819
H	-3.1613428	2.3841876	0.6741325

Si	0.0671415	0.3160752	-0.1248248
H	-0.8618555	2.5770023	0.4752069
C	-0.5526524	-0.9312655	-1.3936381
H	-1.2905455	-1.6285691	-0.9878277
H	0.2931120	-1.5171346	-1.7753498
H	-1.0140254	-0.4163733	-2.2441365
C	1.4206851	1.3577524	-0.9289493
H	2.2565132	0.7309655	-1.2622780
H	1.8216186	2.0999247	-0.2277309
H	1.0399953	1.8985090	-1.8036158
C	0.8338432	-0.5677674	1.3595379
H	1.6992566	-1.1611107	1.0381749
H	0.1355616	-1.2481079	1.8589740
H	1.1836715	0.1544853	2.1063277

^{3}2

E(TPSS-D3/def2-TZVP) = -718.5510519549 (conv)

Lowest Freq. = 15.72 cm^-1

28

3-1 (002_T/c1/tpss-d3.def2-TZVP)

C	-2.6755300	0.8525453	-0.4608232
C	-1.3584538	1.3217068	-0.0527979
H	-3.6666929	-1.6501593	3.2989299
C	-3.9677154	-1.3171051	2.3090720
C	-5.1862401	-1.7507996	1.7701087
H	-5.8289903	-2.4181920	2.3363750
C	-5.5696368	-1.3147029	0.4938237
H	-6.5141406	-1.6462926	0.0706126

C -4.7525600 -0.4646597 -0.2327048

H -5.0532672 -0.1302874 -1.2229667

C -3.5082484 -0.0139466 0.2924286

C -3.1422210 -0.4662425 1.5916329

H -2.1989153 -0.1303272 2.0134197

H -3.0426596 1.1559008 -1.4457430

Si 0.2069618 0.3966992 -0.4943563

H -1.2944436 2.2732401 0.4888201

C 0.2671111 0.1651667 -2.3666171

H -0.6139811 -0.3847196 -2.7177152

H 1.1567778 -0.4044193 -2.6622041

H 0.2935066 1.1277577 -2.8898654

C 1.6923231 1.3924375 0.0970951

H 2.6310556 0.8774918 -0.1400223

H 1.6618419 1.5435713 1.1827153

H 1.7249911 2.3799605 -0.3779366

C 0.1896624 -1.3044506 0.3209220

H 1.0505601 -1.8985700 -0.0106545

H -0.7214965 -1.8548796 0.0613121

H 0.2338411 -1.2288236 1.4130589

***E*-1**

E(TPSS-D3/def2-TZVP) = -797.3040398818 (conv)

Lowest Freq. = 34.79 cm^-1

34

E-2 (003/c1/tpss-d3.def2-TZVP)

C -2.8583034 1.4703922 0.7394465

C -1.5096706 1.4584872 0.8343181

H	-3.5250244	-3.1239520	1.1600681
C	-3.8823072	-2.1862163	0.7431267
C	-5.0453142	-2.1624627	-0.0290721
H	-5.5928197	-3.0800576	-0.2244273
C	-5.5038211	-0.9472321	-0.5372088
H	-6.4082663	-0.9150294	-1.1386476
C	-4.8067561	0.2311668	-0.2772774
H	-5.1763908	1.1655151	-0.6890880
C	-3.6238500	0.2230804	0.4812731
C	-3.1840317	-1.0089982	0.9955539
H	-2.2955185	-1.0314761	1.6199837
Si	-0.3420334	2.8819141	1.1907284
H	-1.0290625	0.4898313	0.6695662
C	-0.7416826	3.7264933	2.8327089
H	-1.7207691	4.2176110	2.8222753
H	0.0104687	4.4919450	3.0616128
H	-0.7427459	3.0015614	3.6549246
C	1.3878576	2.1392934	1.3001617
H	2.1406361	2.9106890	1.5016013
H	1.6655908	1.6402290	0.3638609
H	1.4504942	1.3959083	2.1041329
C	-0.3673309	4.1617581	-0.1980887
H	0.3657705	4.9553001	-0.0065271
H	-1.3496317	4.6350714	-0.3058110
H	-0.1170323	3.6974665	-1.1589615
C	-3.6635326	2.7399687	0.9134880
C	-4.5541190	2.7076713	2.1669203
H	-3.9424674	2.5897758	3.0672278

H	-5.2577605	1.8705640	2.1252688
H	-5.1285939	3.6354277	2.2588851
H	-4.2927998	2.9055474	0.0290154
H	-2.9831021	3.5950662	0.9660310

***Z*-1**

E(TPSS-D3/def2-TZVP) = -797.3033600240 (conv)

Lowest Freq. = 35.29 cm^-1

34

Z-2 (004/c1/tpss-d3.def2-TZVP)

C	-2.9339836	1.6580284	0.5512678
C	-1.6096872	1.8116938	0.3547749
H	-3.4560999	-2.7196503	2.0500495
C	-3.7650059	-1.9563470	1.3410537
C	-4.7274179	-2.2472629	0.3743268
H	-5.1677319	-3.2388580	0.3217912
C	-5.1262477	-1.2542109	-0.5225588
H	-5.8739483	-1.4742178	-1.2796669
C	-4.5635941	0.0186526	-0.4555052
H	-4.8678440	0.7812772	-1.1676910
C	-3.5748441	0.3155253	0.4938646
C	-3.1951292	-0.6849627	1.3993832
H	-2.4495102	-0.4519637	2.1536397
Si	-0.3322984	0.5553535	-0.2089287
H	-1.2278671	2.8320994	0.4508731
C	-1.0402615	-0.7282879	-1.3931062
H	-1.7353875	-1.4153272	-0.9016425
H	-0.2244059	-1.3226458	-1.8241197

H	-1.5752179	-0.2457838	-2.2190643
C	0.9828291	1.5587195	-1.1211059
H	1.7921232	0.9139172	-1.4843706
H	1.4310885	2.3167901	-0.4669894
H	0.5562632	2.0785704	-1.9873756
C	0.5030466	-0.3094156	1.2484327
H	1.3600063	-0.9011187	0.9023523
H	-0.1826234	-0.9892821	1.7652590
H	0.8733592	0.4165865	1.9818183
C	-3.8878711	2.7963820	0.8599329
C	-3.2767257	4.1953352	0.8923390
H	-2.8220901	4.4528789	-0.0702673
H	-2.5020827	4.2734668	1.6627293
H	-4.0473124	4.9406629	1.1141832
H	-4.3738933	2.5768312	1.8220992
H	-4.7027149	2.7630034	0.1216119

^{3}1

E(TPSS-D3/def2-TZVP) = -797.2298968254 (conv)

Lowest Freq. = 29.04 cm^-1

34

3-2 (003_T/c1/tpss-d3.def2-TZVP)

C	-3.0176217	1.6882671	-0.0798996
C	-1.5767765	1.9401514	-0.1500088
H	-2.4125420	-2.6371572	1.4208300
C	-3.1158141	-1.8470857	1.1703638
C	-4.4922617	-2.0690133	1.3077738
H	-4.8625558	-3.0249442	1.6657837

C	-5.3850382	-1.0422598	0.9766260
H	-6.4551416	-1.2034611	1.0786903
C	-4.9210493	0.1821555	0.5197536
H	-5.6373729	0.9593021	0.2723464
C	-3.5271232	0.4356232	0.3754258
C	-2.6415430	-0.6267336	0.7178727
H	-1.5719621	-0.4635701	0.6118413
Si	-0.5282604	2.5767831	1.2602553
H	-1.0881590	1.7535693	-1.1156792
C	-1.3727553	2.1741542	2.8927166
H	-2.3570023	2.6498822	2.9621463
H	-0.7699507	2.5244219	3.7393777
H	-1.5219850	1.0946821	3.0060043
C	1.1596657	1.7390280	1.1641469
H	1.8283003	2.1137039	1.9488638
H	1.6419262	1.9288968	0.1977875
H	1.0739264	0.6533747	1.2877842
C	-0.2841218	4.4457356	1.1087489
H	0.3486512	4.8196772	1.9237206
H	-1.2385602	4.9822048	1.1529866
H	0.2010794	4.7053485	0.1607223
C	-3.9381772	2.8321620	-0.4451178
C	-4.3951976	3.6580713	0.7755536
H	-3.5374379	4.1146260	1.2786657
H	-4.9141476	3.0247890	1.5015416
H	-5.0740456	4.4606914	0.4663595
H	-4.8212461	2.4445373	-0.9690429
H	-3.4196207	3.4946966	-1.1478706

5. NMR Spectra of Key Compounds

^{1}H NMR (200 MHz, $CDCl_3$)

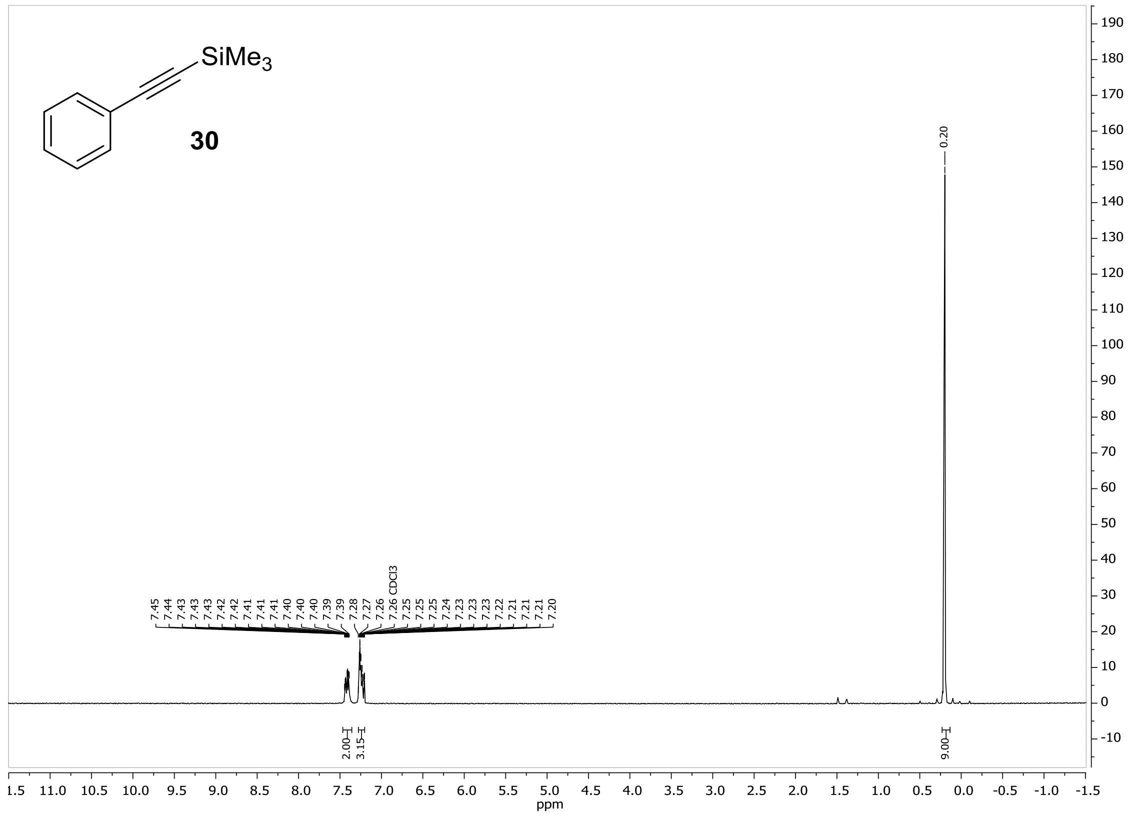

^{1}H NMR (300 MHz, $CDCl_3$)

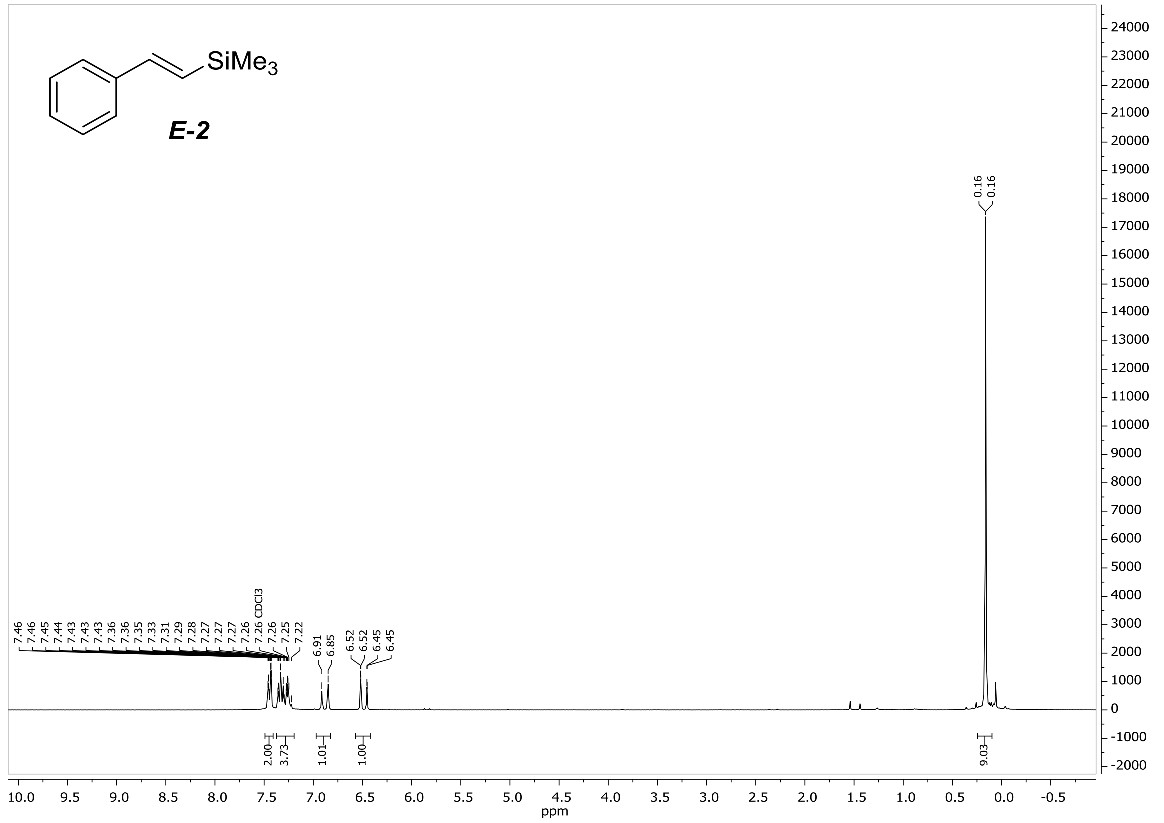

^1H NMR (300 MHz, $CDCl_3$)

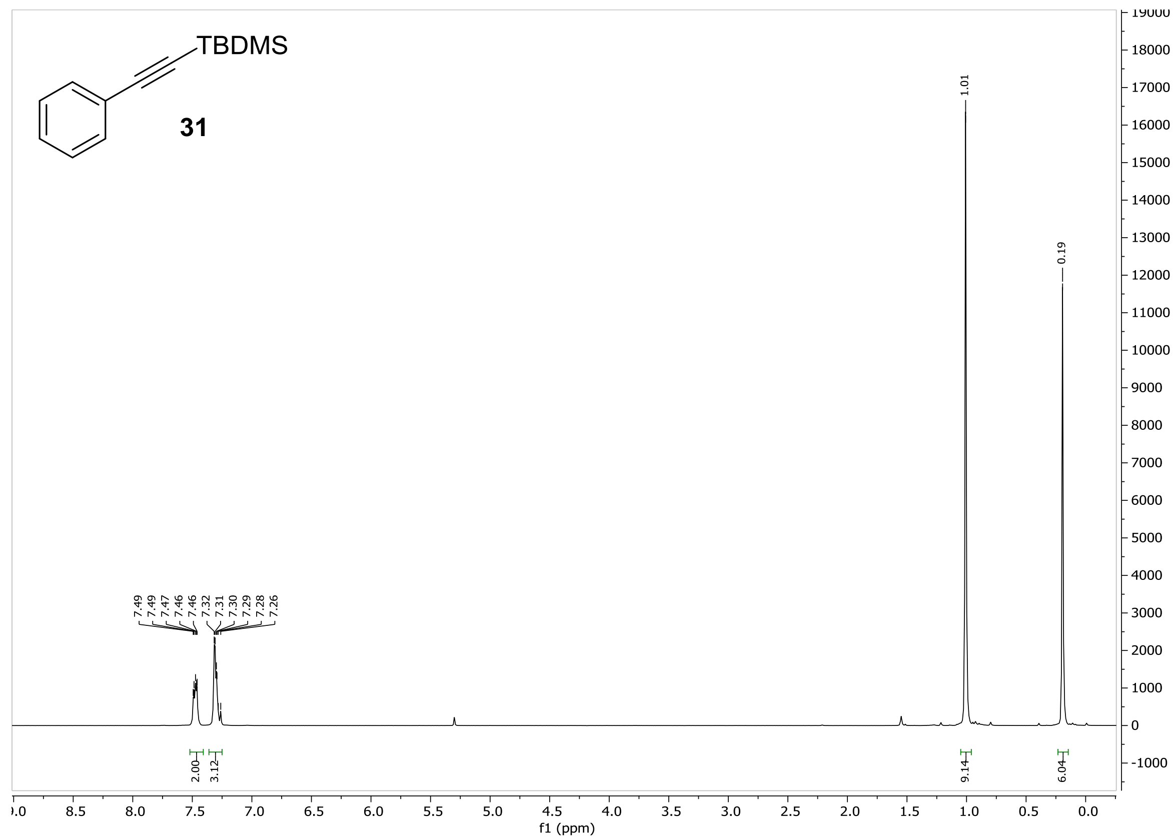

^1H NMR (400 MHz, $CDCl_3$)

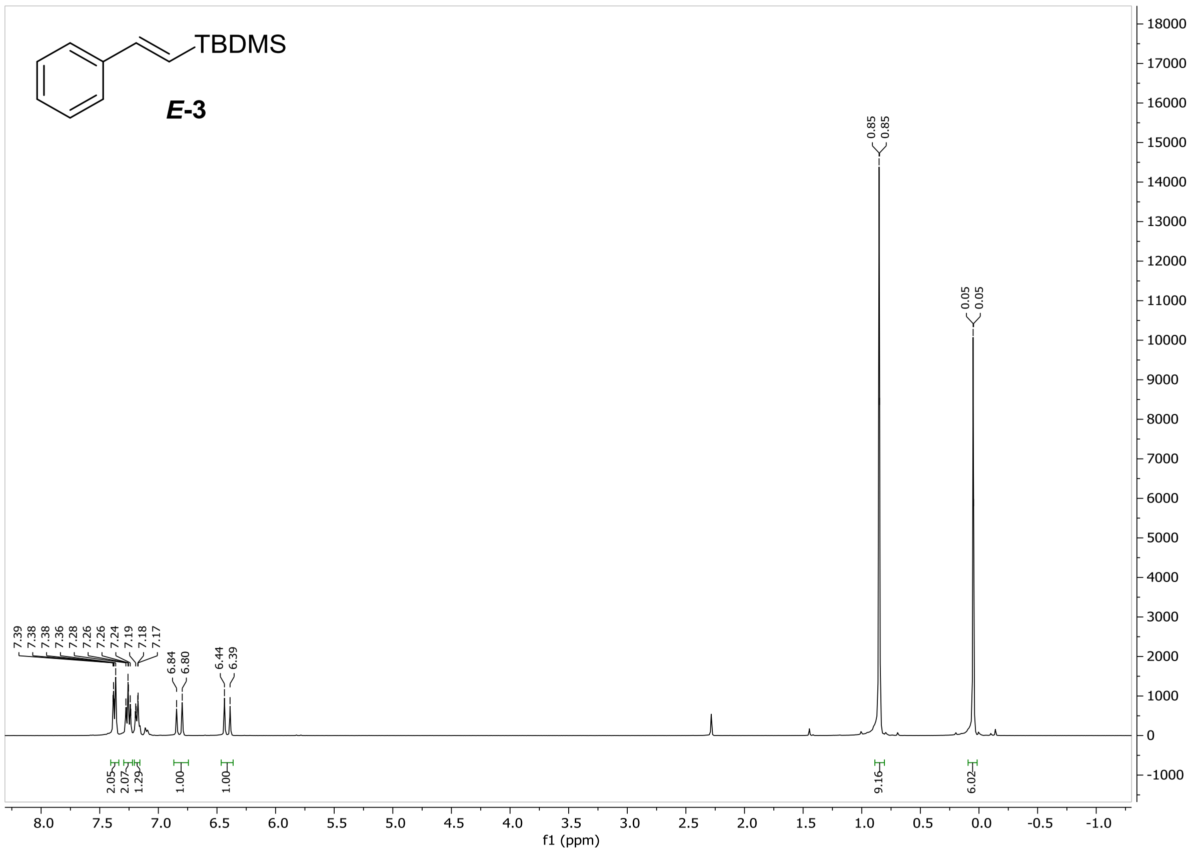

^{1}H NMR (400 MHz, $CDCl_3$)

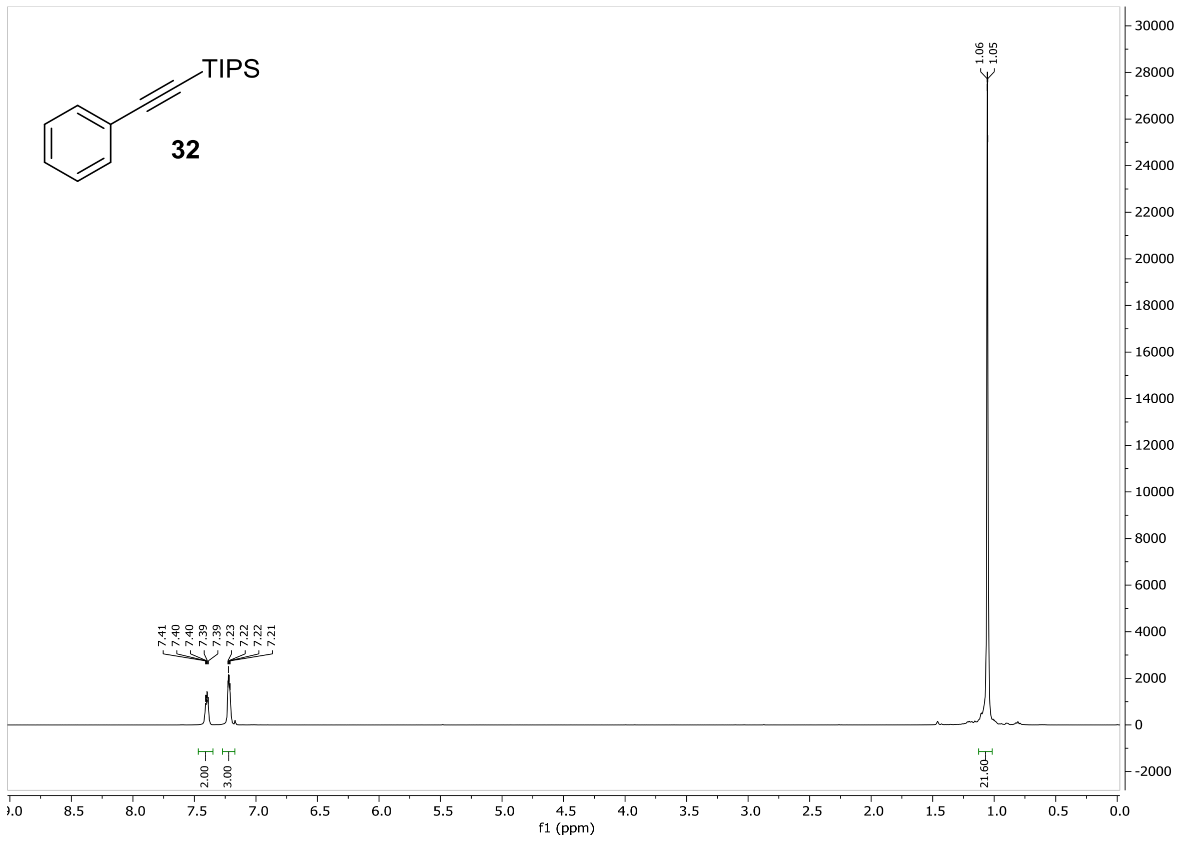

^{1}H NMR (300 MHz, $CDCl_3$

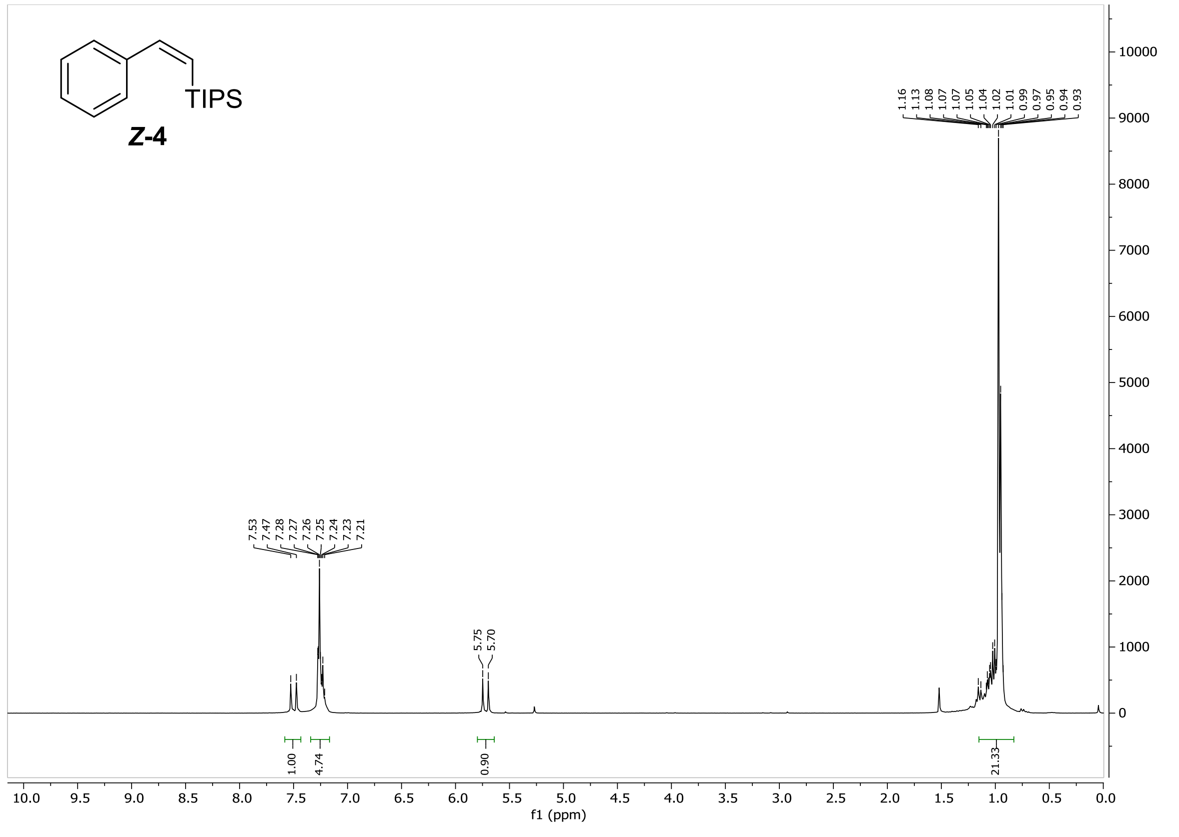

^{1}H NMR (400 MHz, CD_2Cl_2)

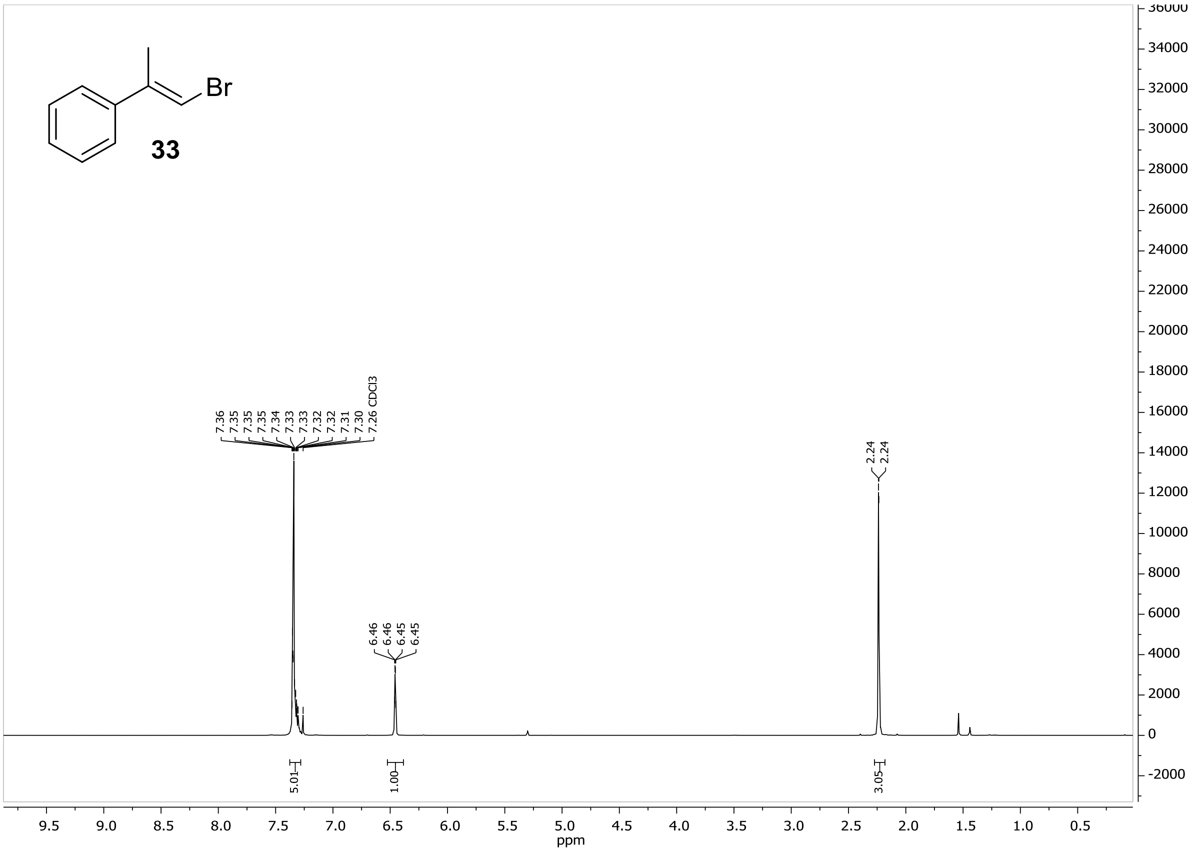

^{1}H NMR (400 MHz, CD_2Cl_2)

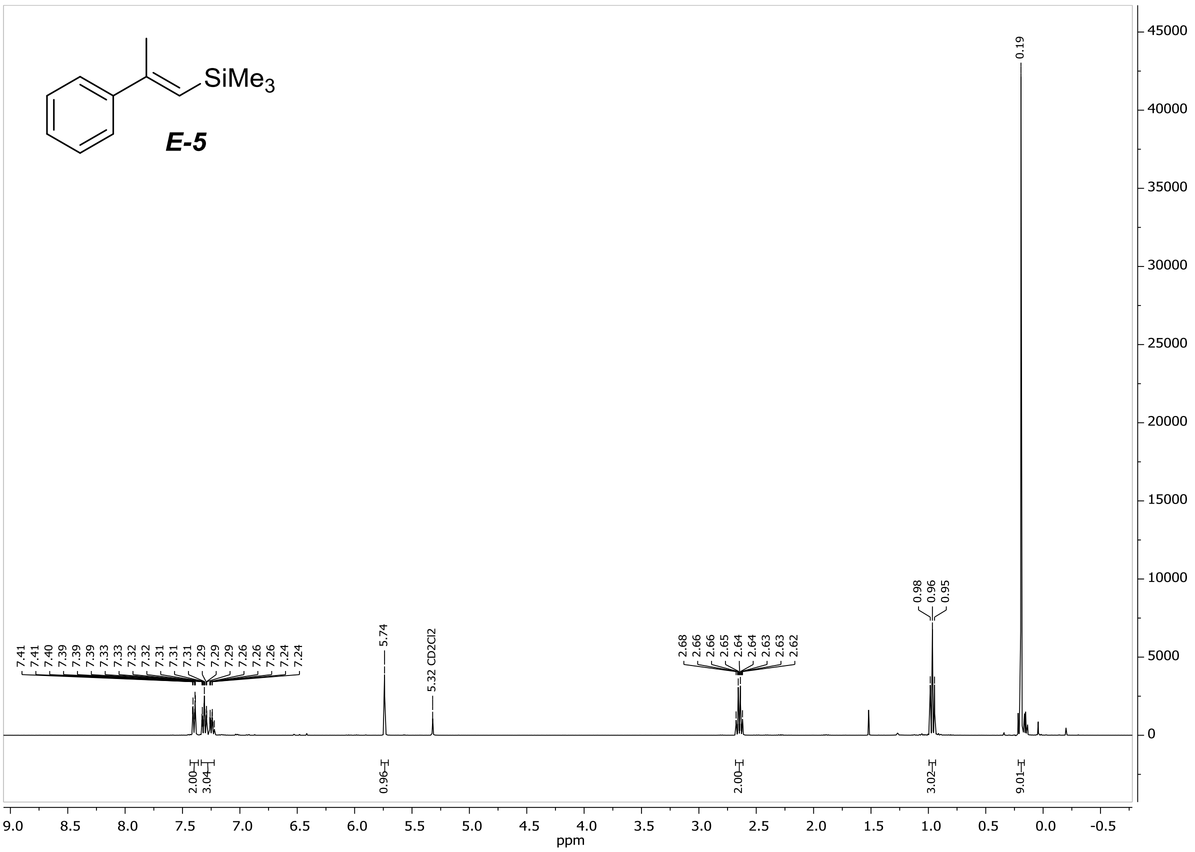

^1H NMR (400 MHz, CD_2Cl_2)

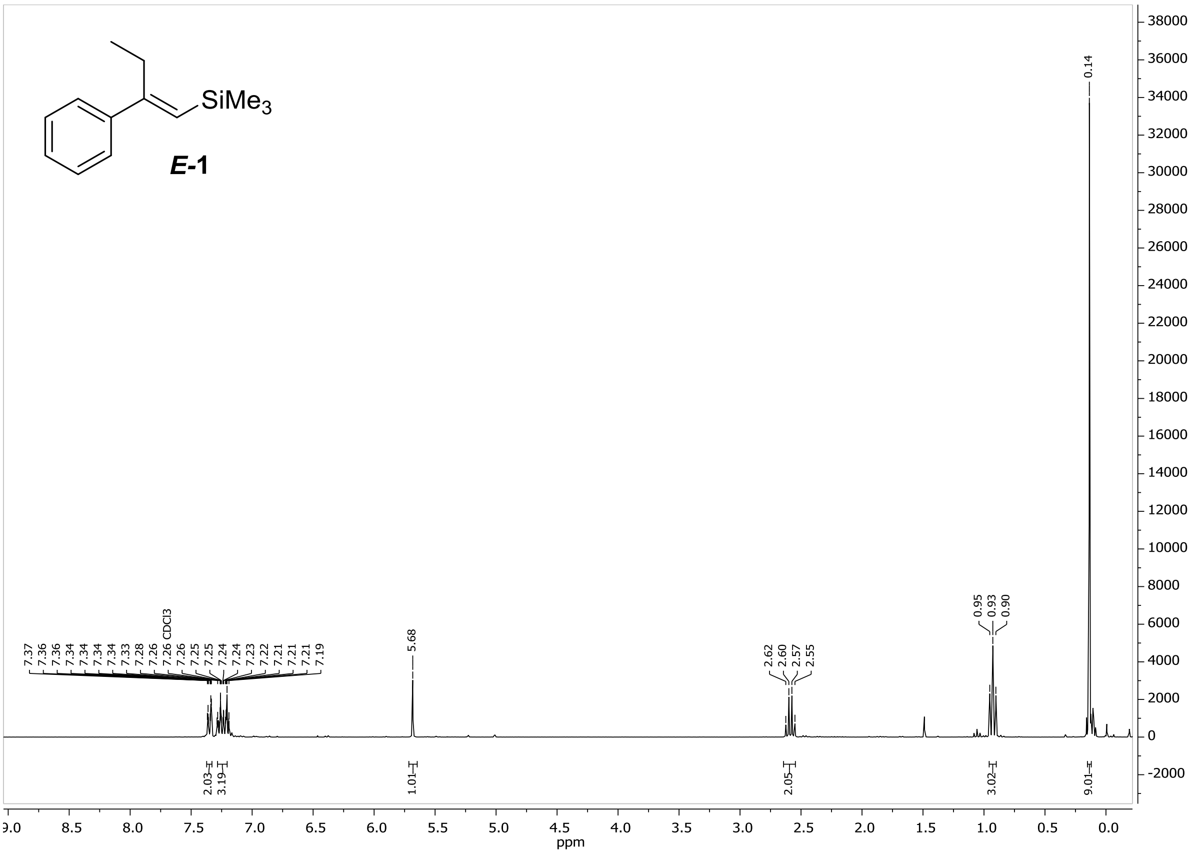

^1H NMR (500 MHz, CD_2Cl_2)

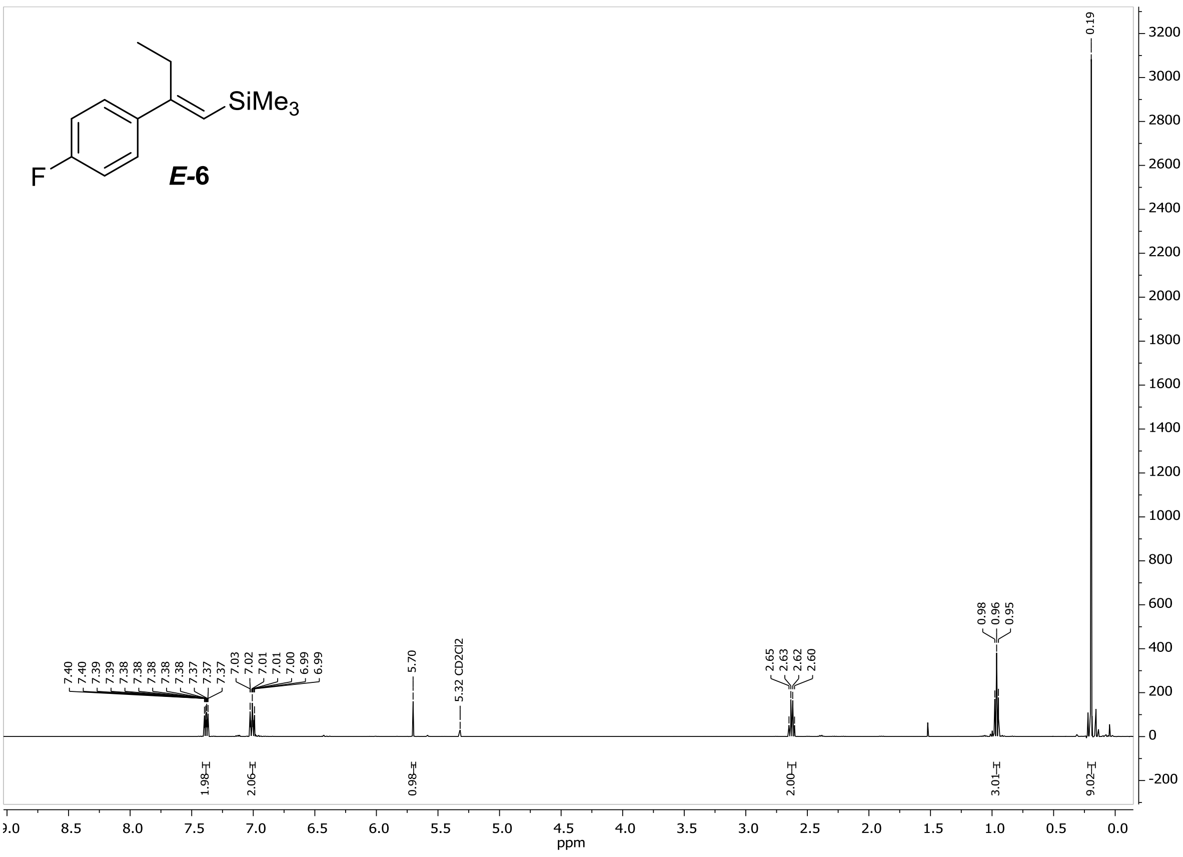

^{13}C NMR (126MHz, CD_2Cl_2)

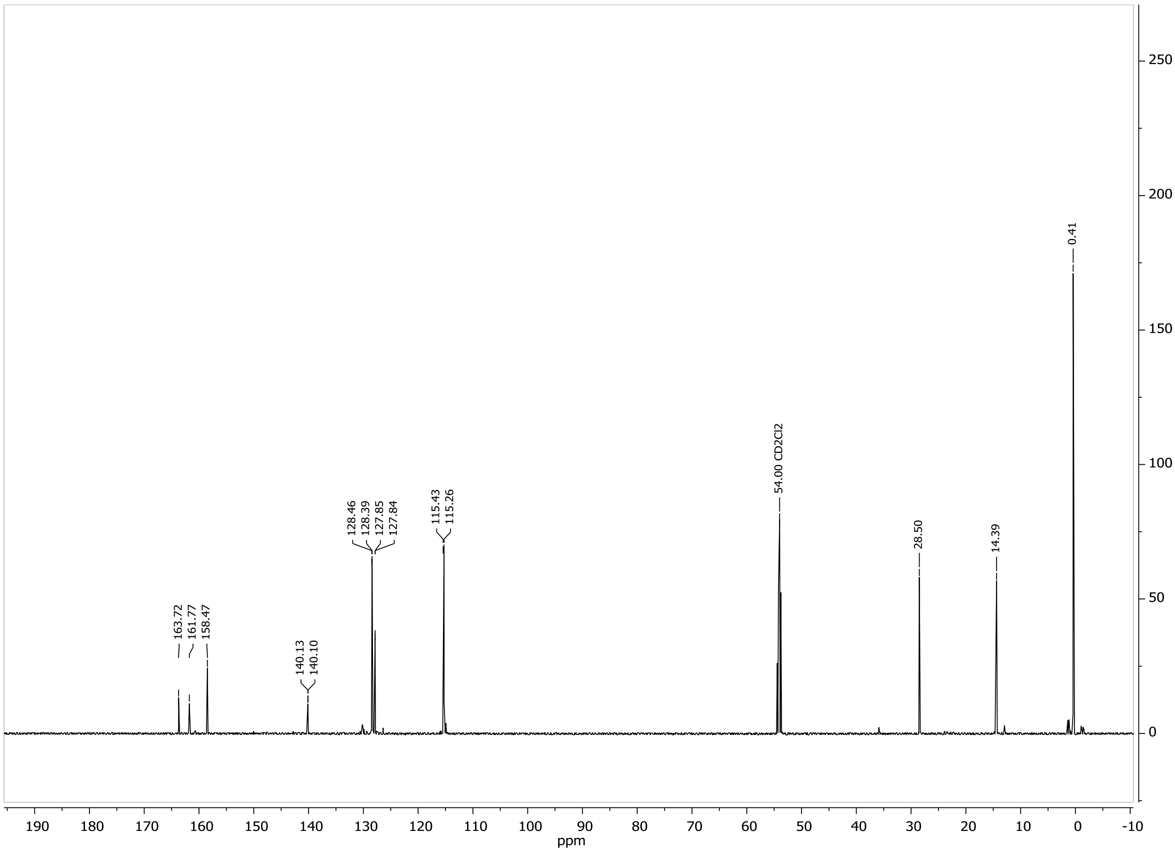

^{19}F NMR (470 MHz, CD_2Cl_2)

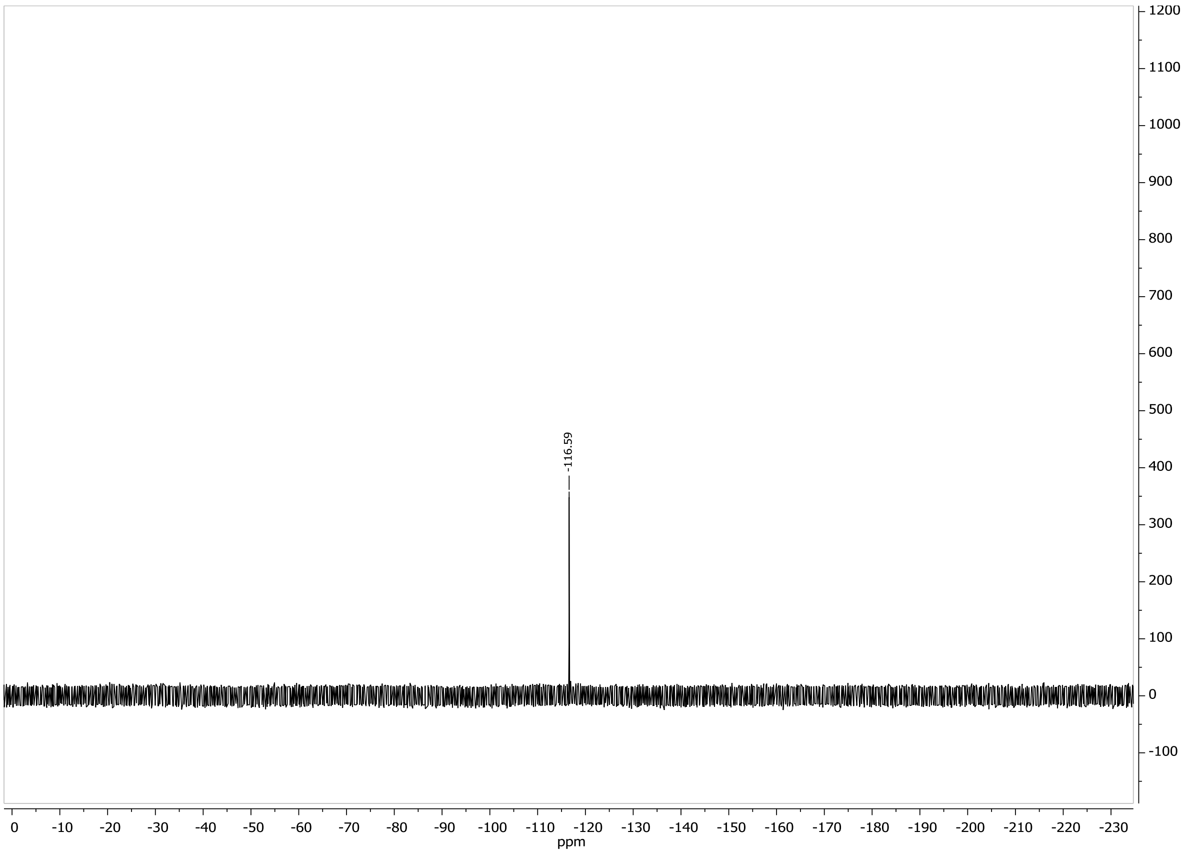

^{1}H NMR (300 MHz, $CDCl_3$

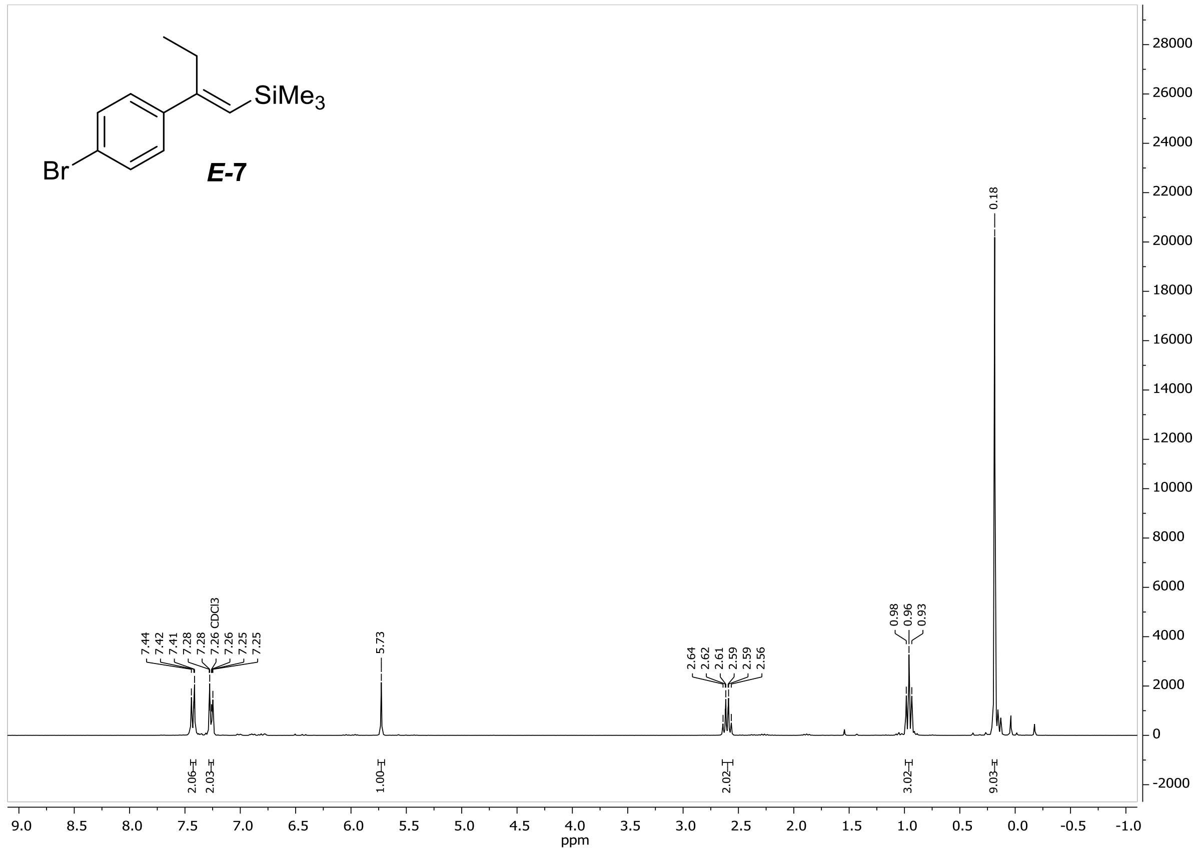

^{13}C NMR (126 MHz, CD_2Cl_2)

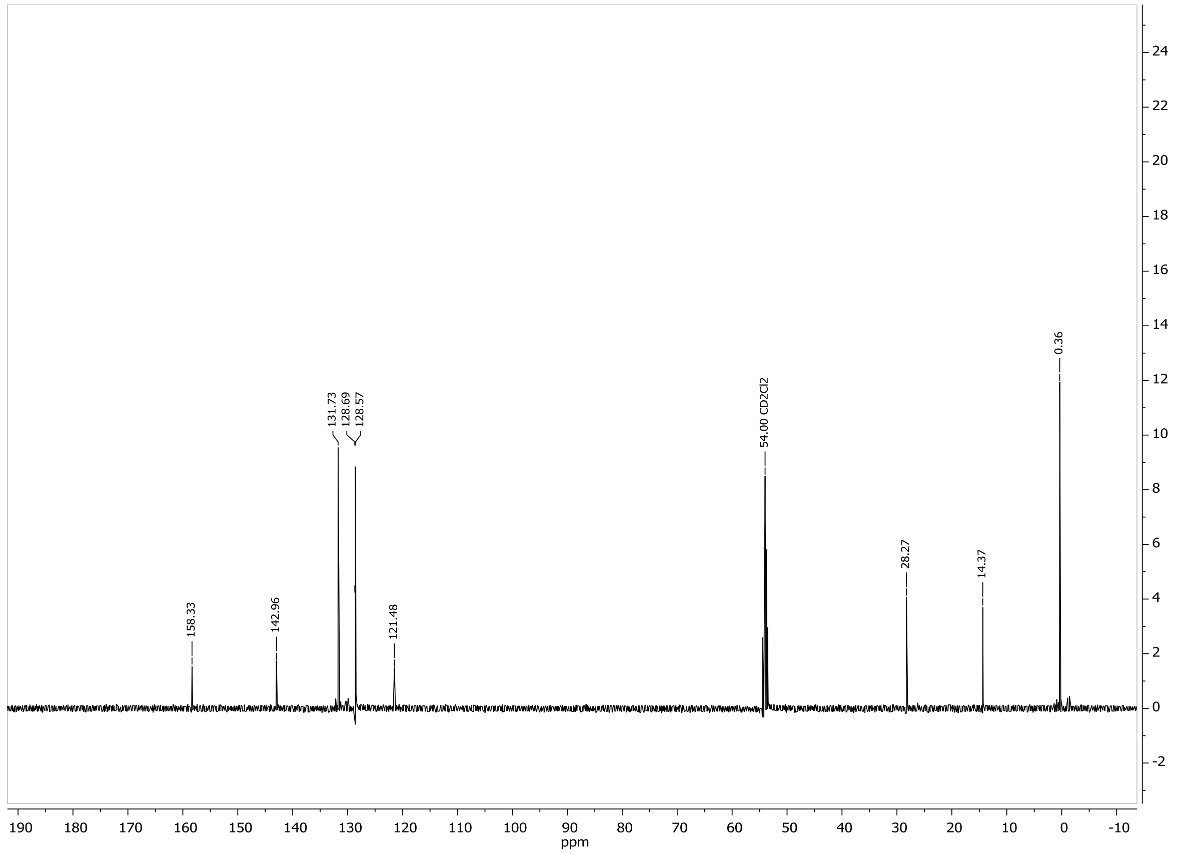

^1H NMR (500 MHz, CD_2Cl_2)

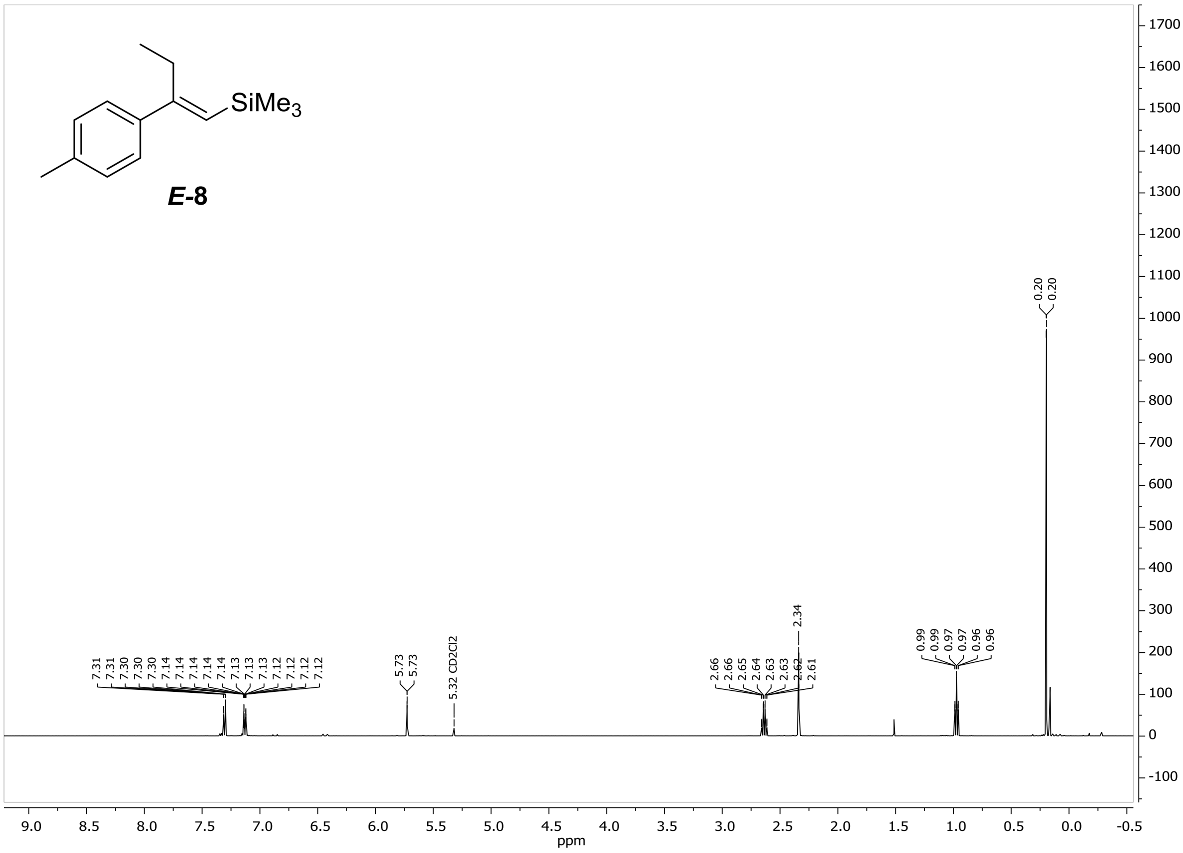

^{13}C NMR (126 MHz, CD_2Cl_2)

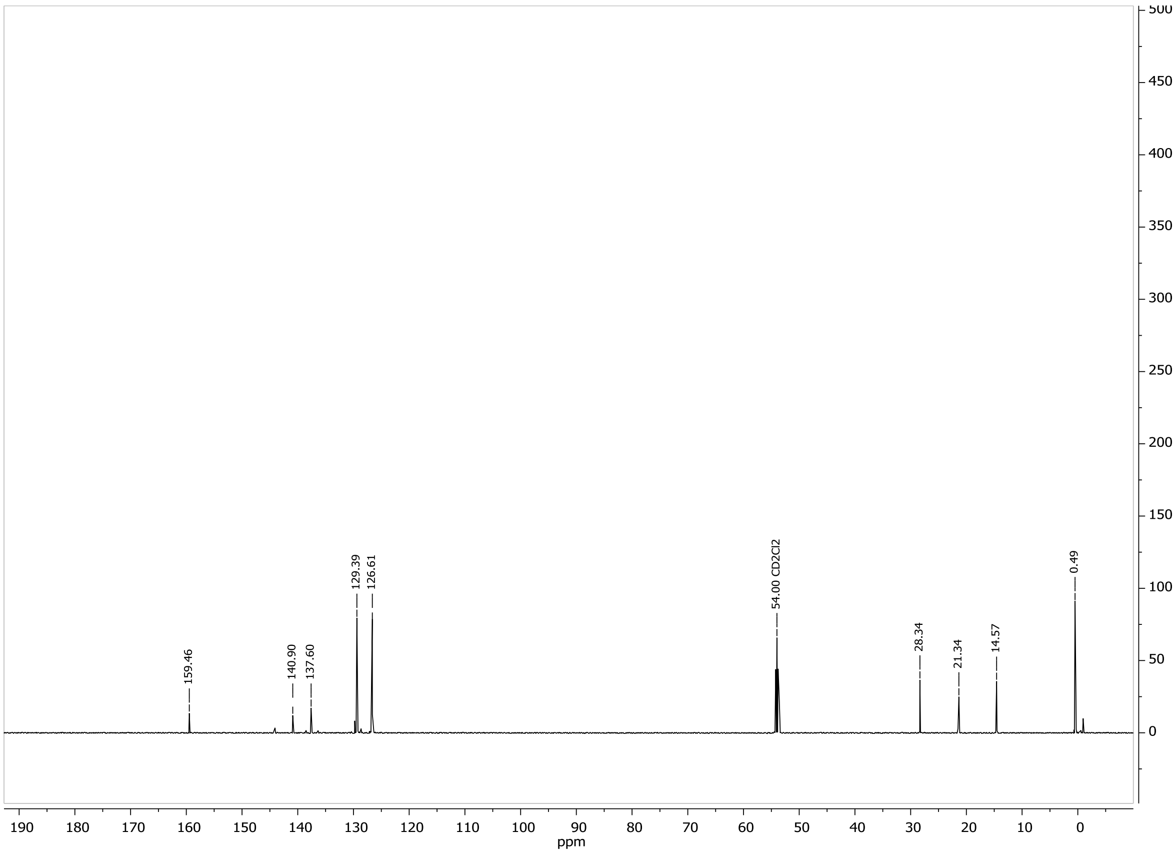

^{1}H NMR (400 MHz, $CDCl_3$)

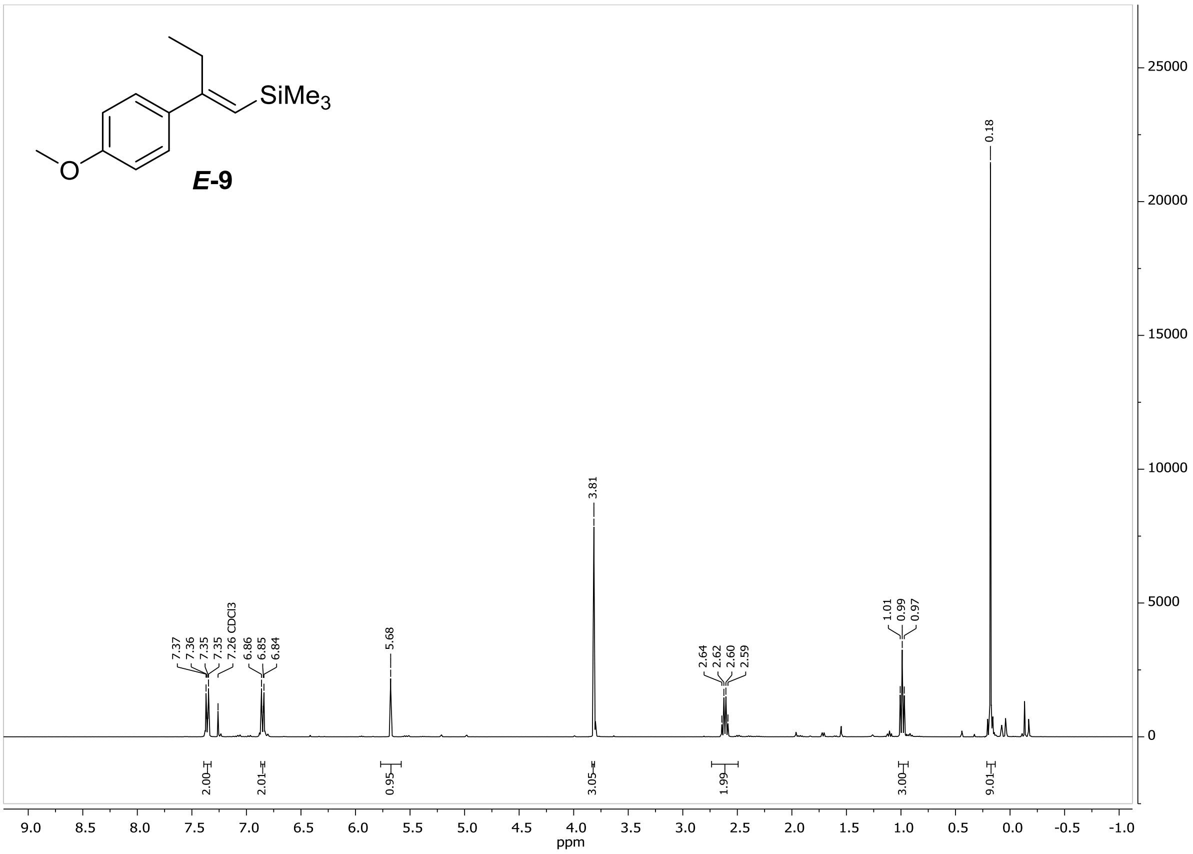

^{1}H NMR (500 MHz, CD_2Cl_2)

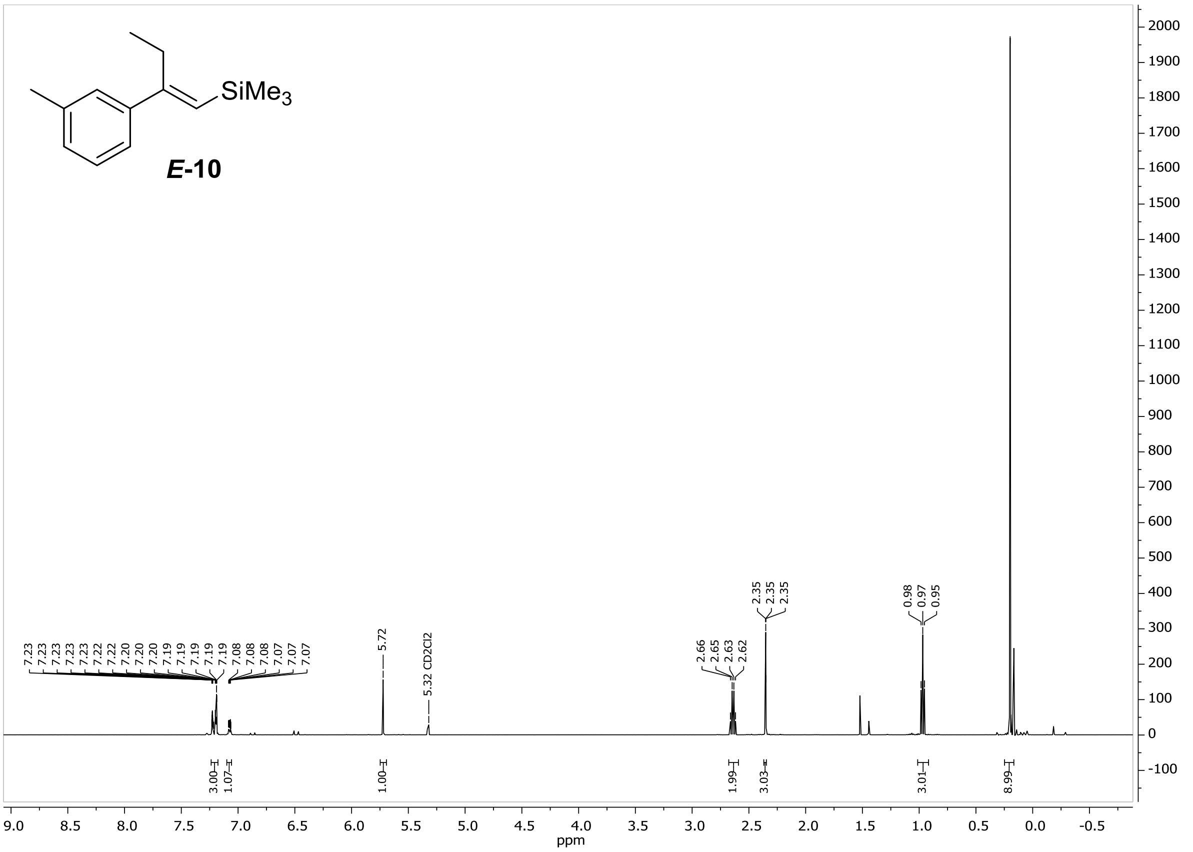

^{13}C NMR (126 MHz, CD_2Cl_2)

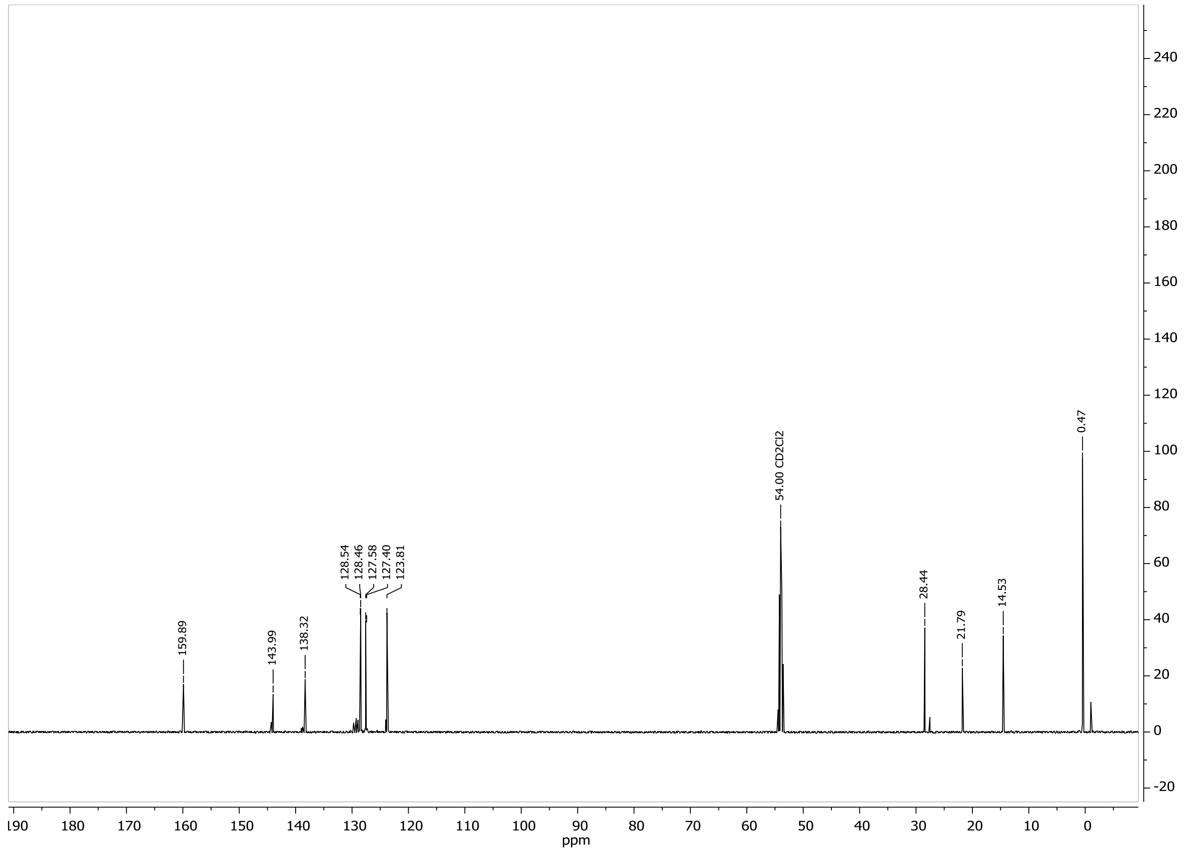

^{1}H NMR (400 MHz, $CDCl_3$)

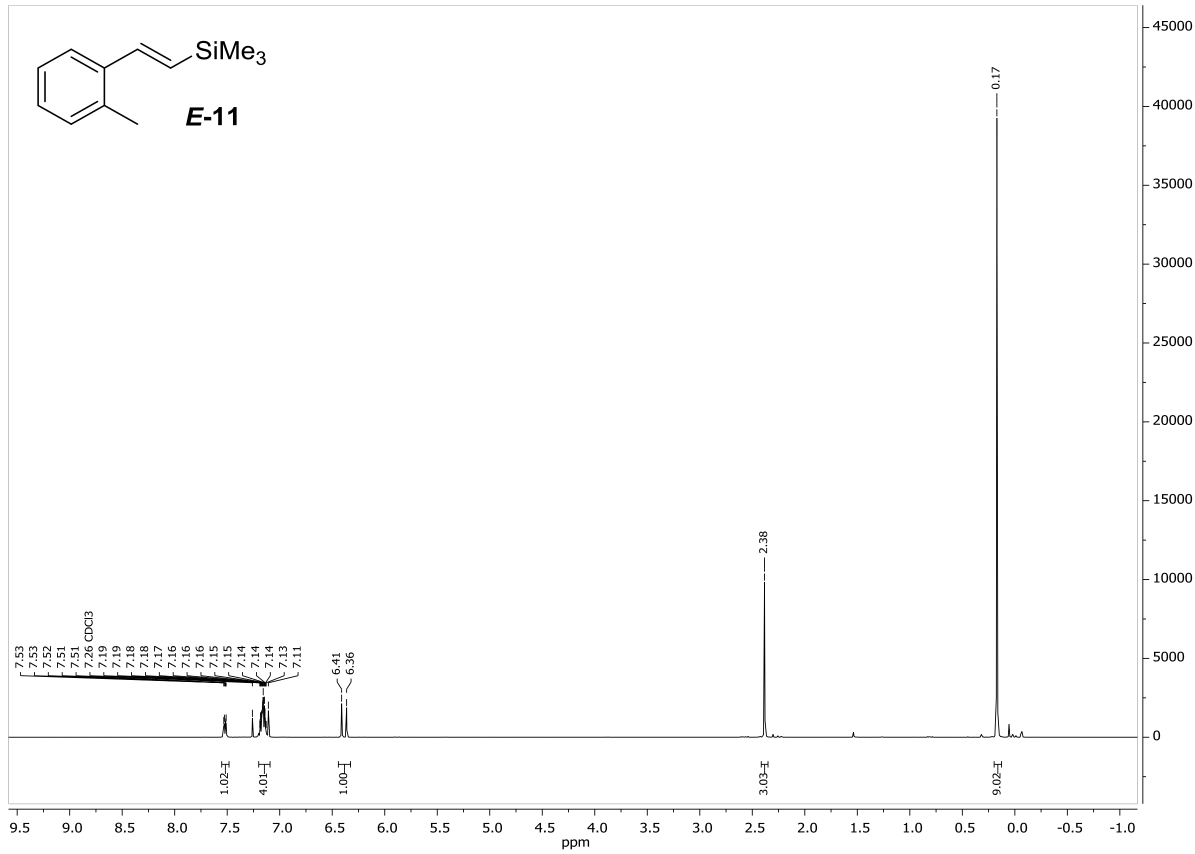

^{1}H NMR (600 MHz, CD_2Cl_2)

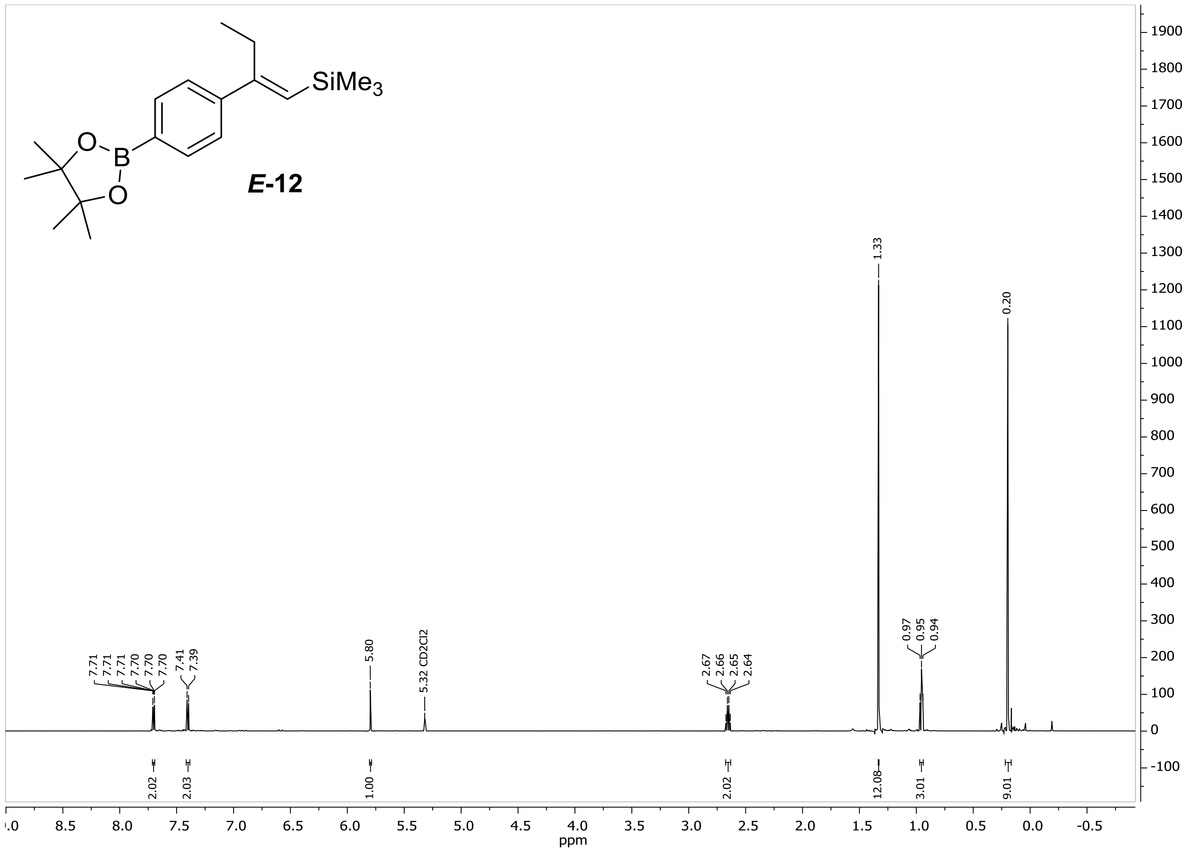

^{13}C NMR (151 MHz, CD_2Cl_2)

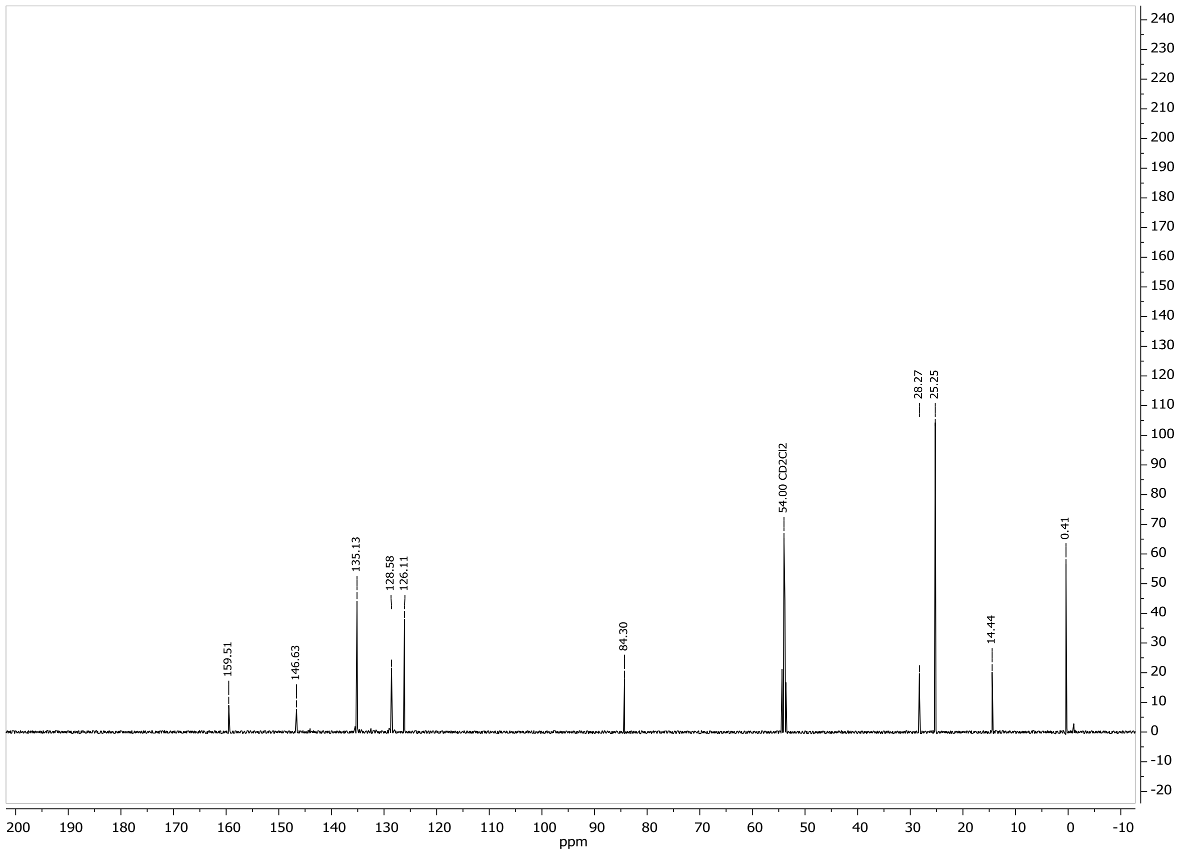

^{1}H NMR (500 MHz, CD_2Cl_2)

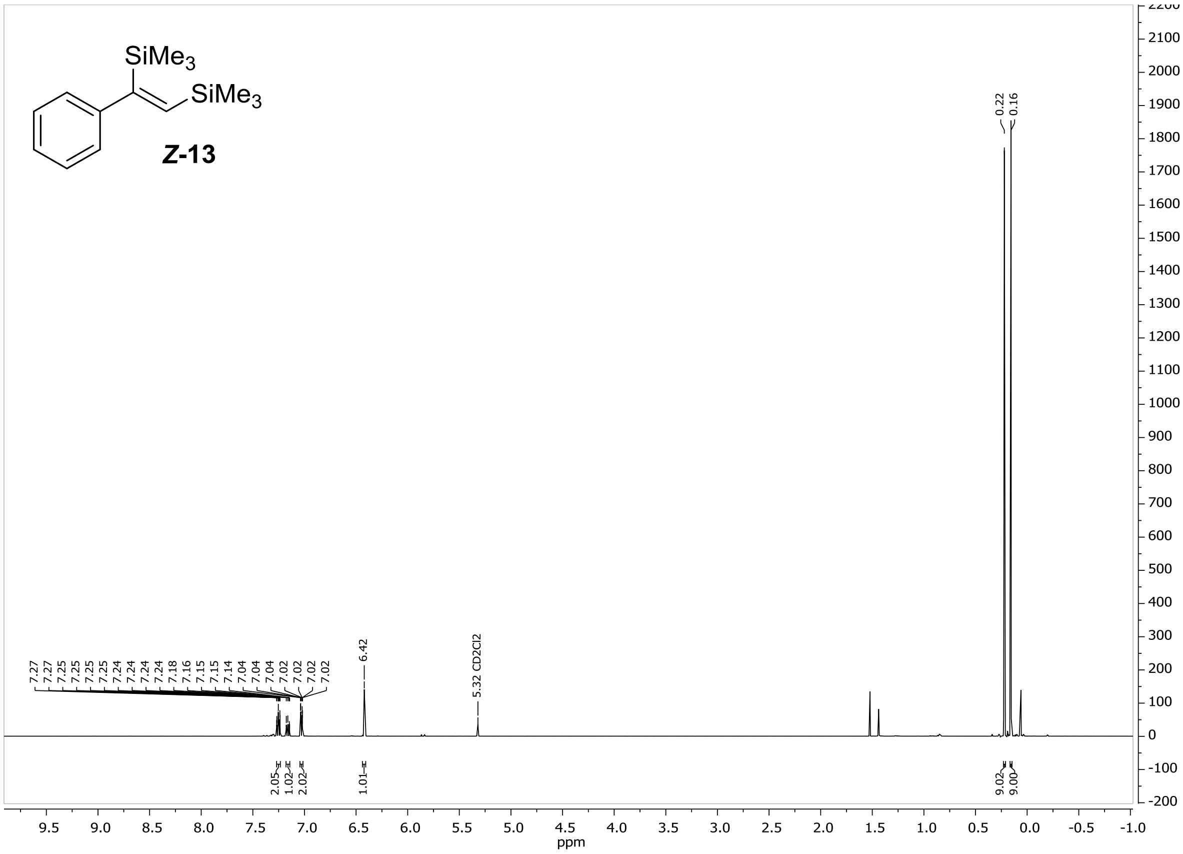

^{13}C NMR (126 MHz, CD_2Cl_2)

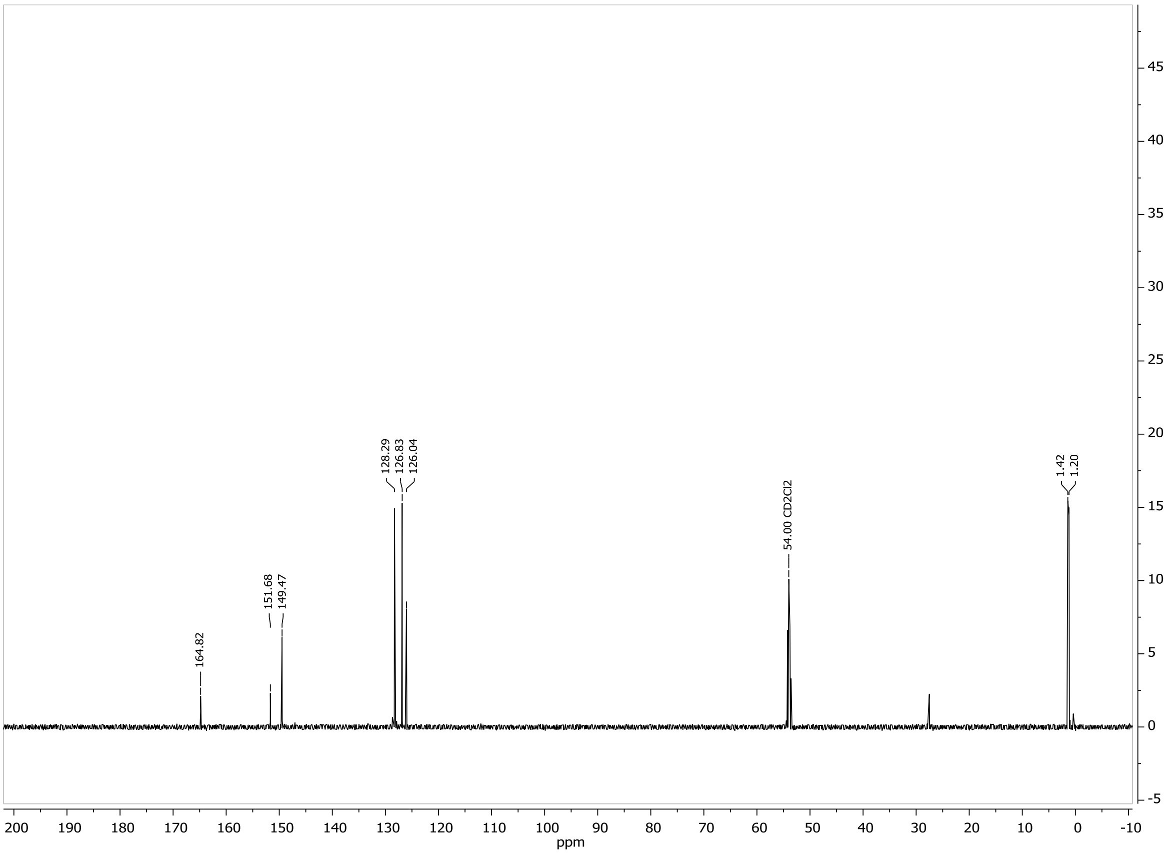

^{1}H NMR (300 MHz, CD_2Cl_2)

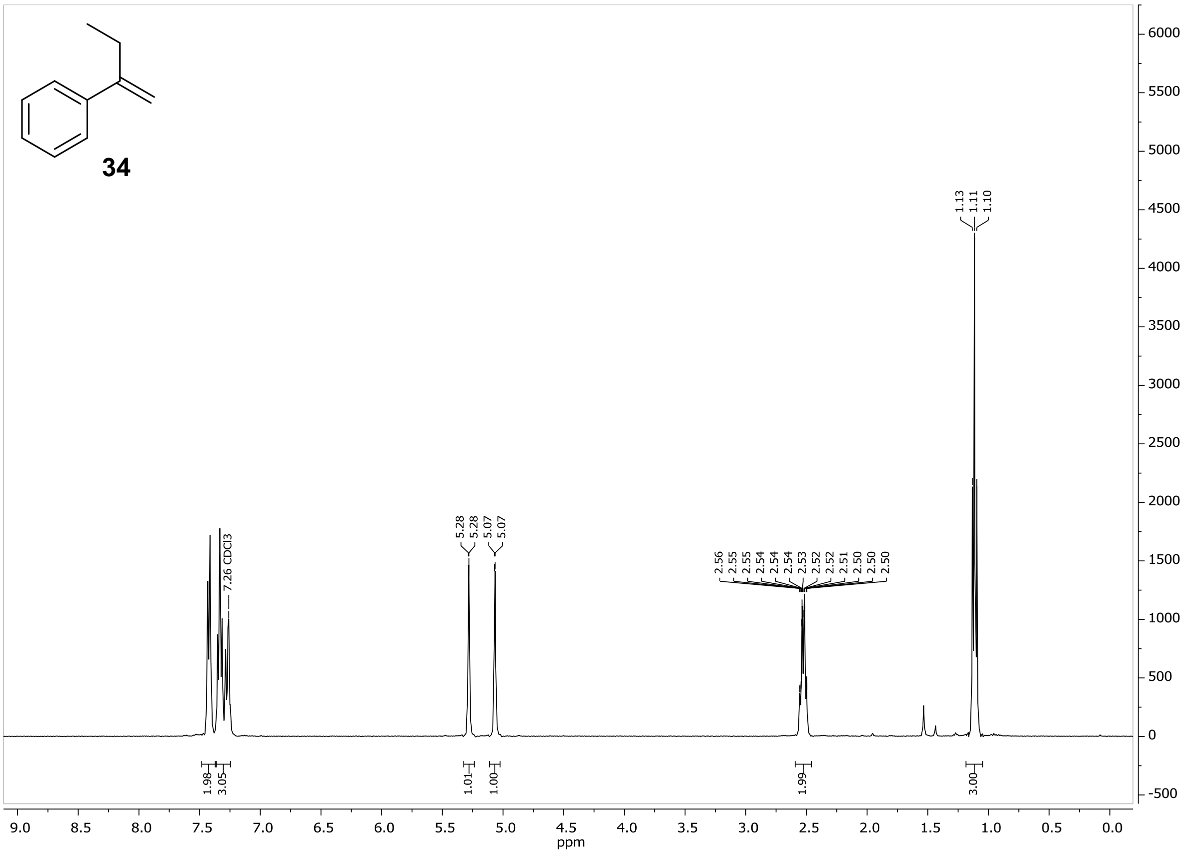

1H NMR (300 MHz, $CDCl_3$)

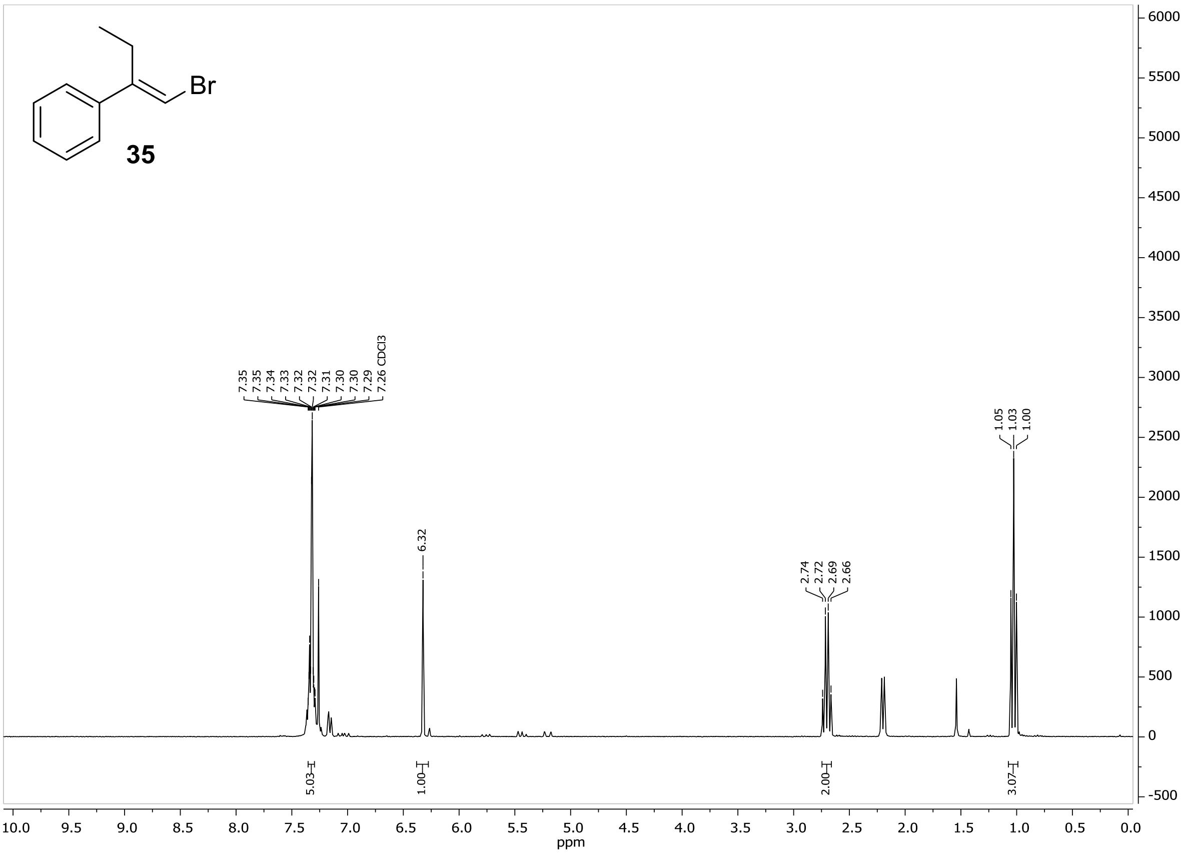

^{1}H NMR (500 MHz,CD_2Cl_2)

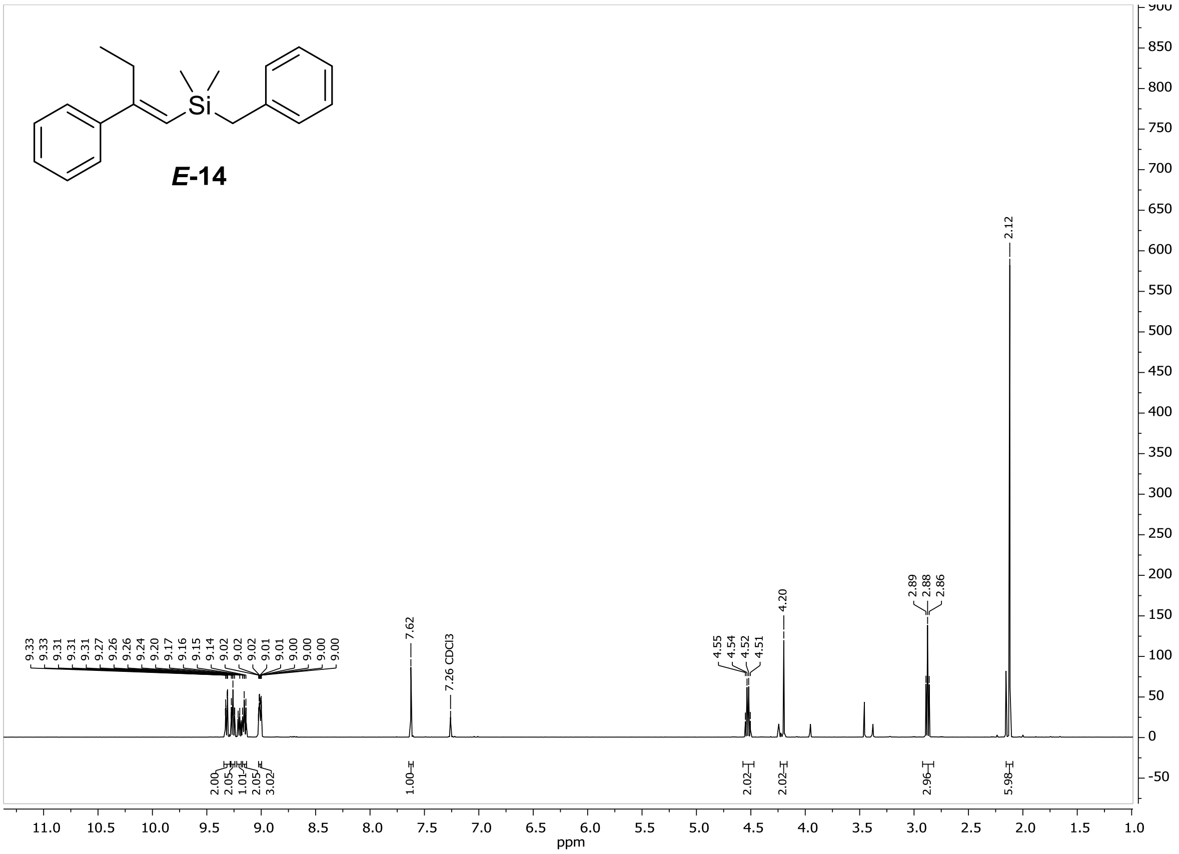

^{13}C NMR (126 MHz, CD_2Cl_2)

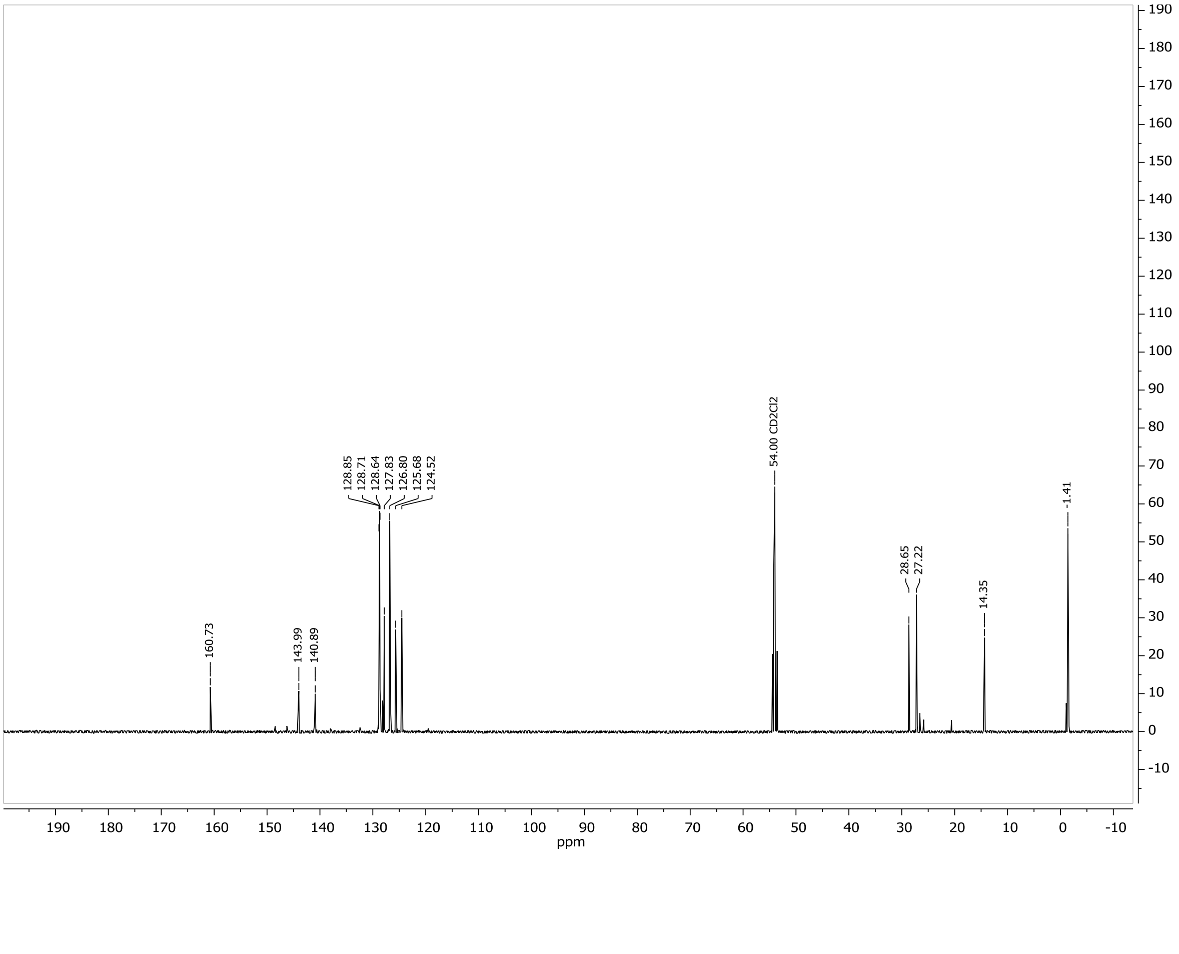

^{1}H NMR (500 MHz, CD_2Cl_2)

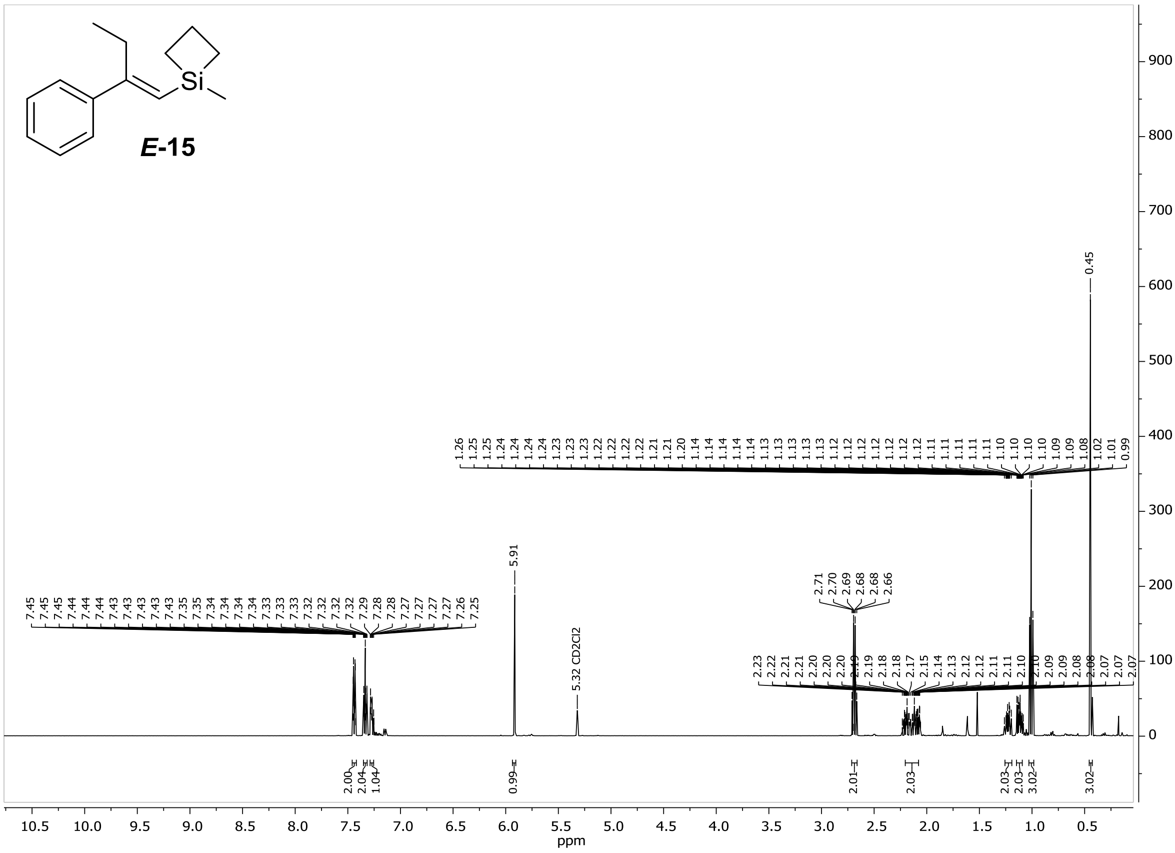

^{13}C NMR (126 MHz, CD_2Cl_2)

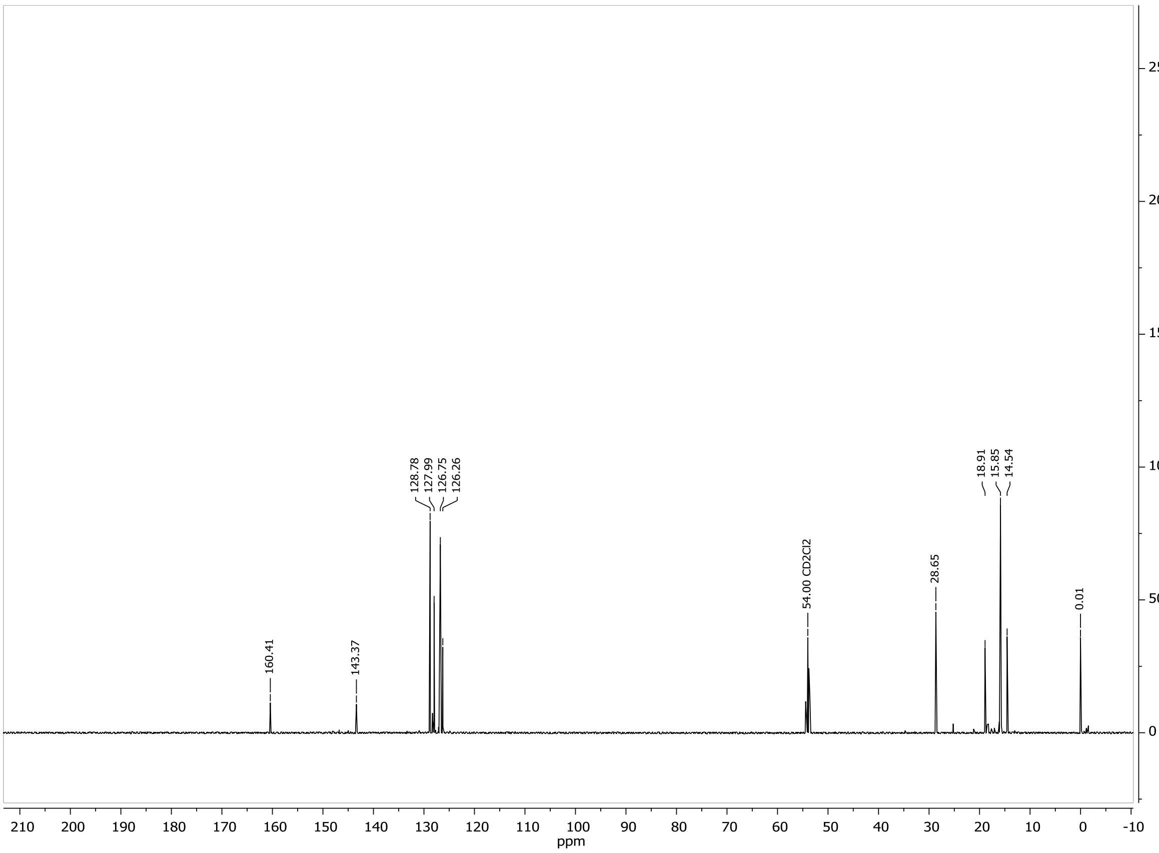

^{1}H NMR (600 MHz, CD_2Cl_2)

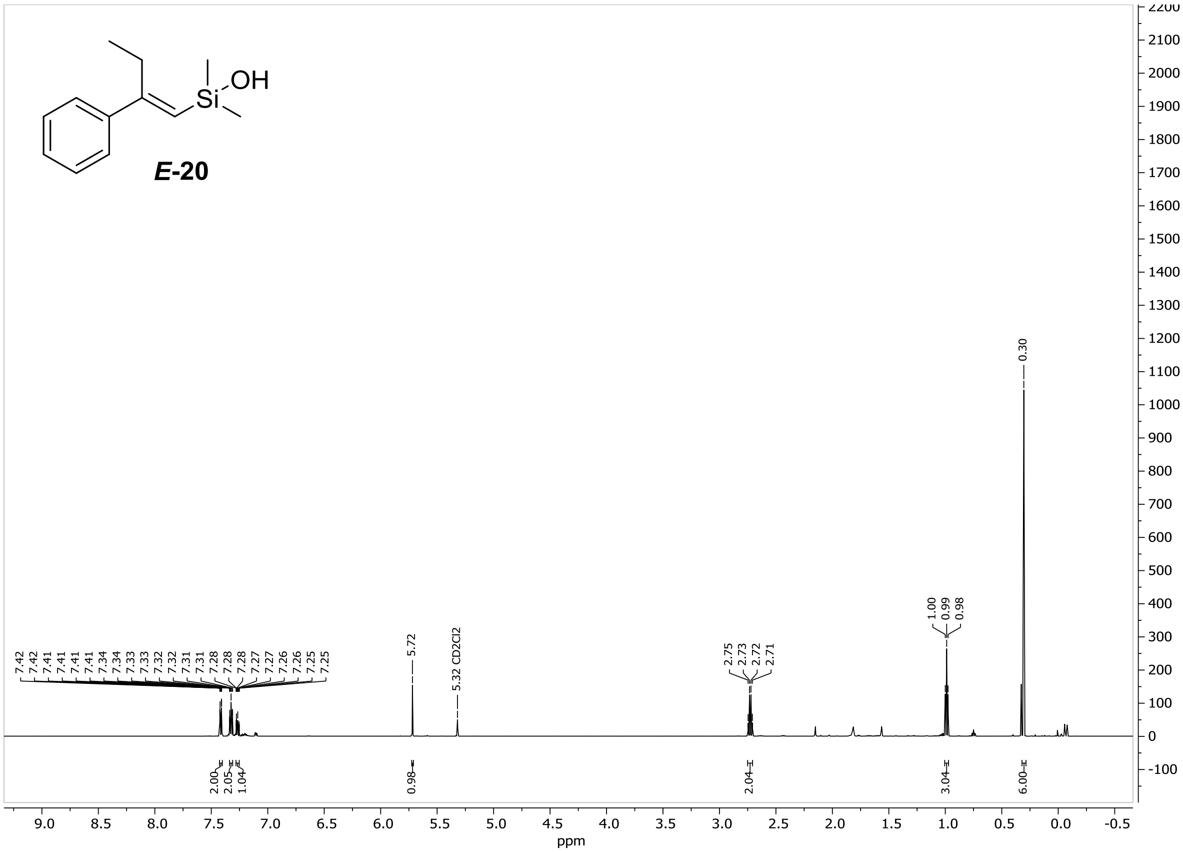

^{13}C NMR (151 MHz, CD_2Cl_2)

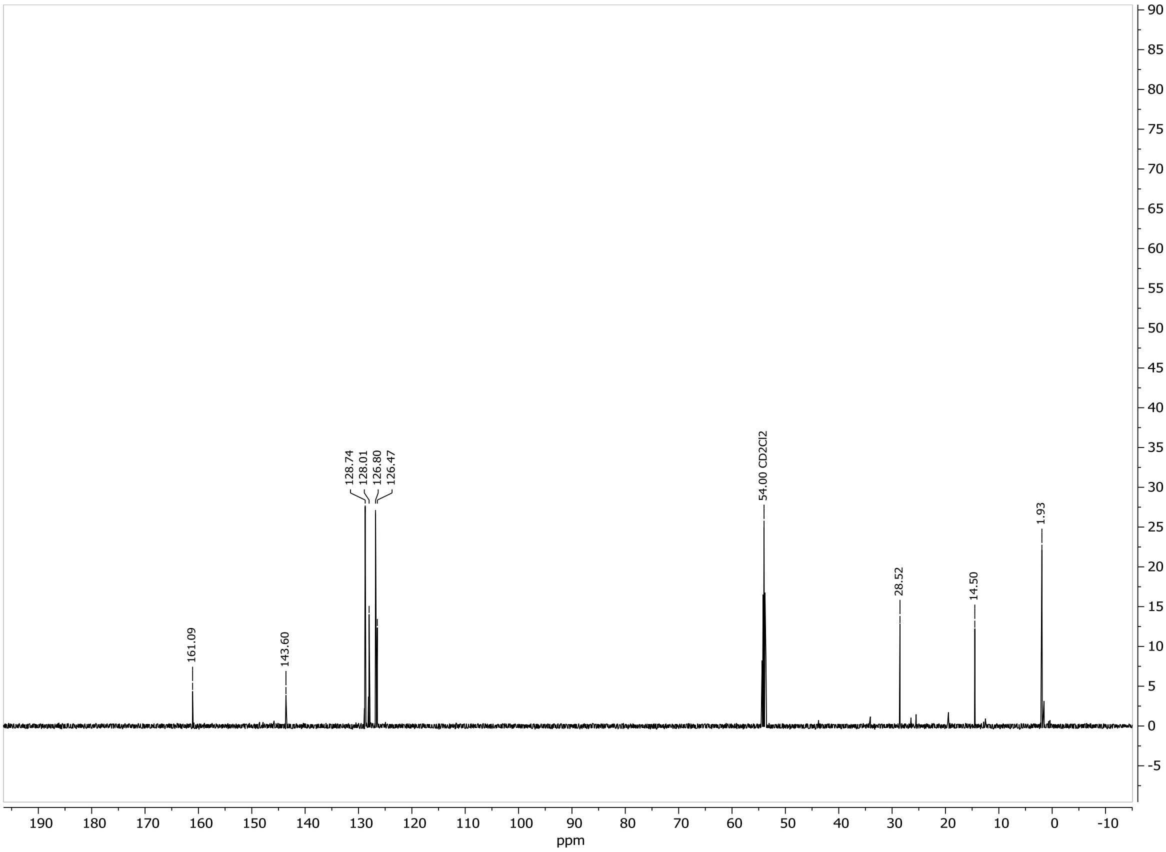

^{1}H NMR (300 MHz, $CDCl_3$)

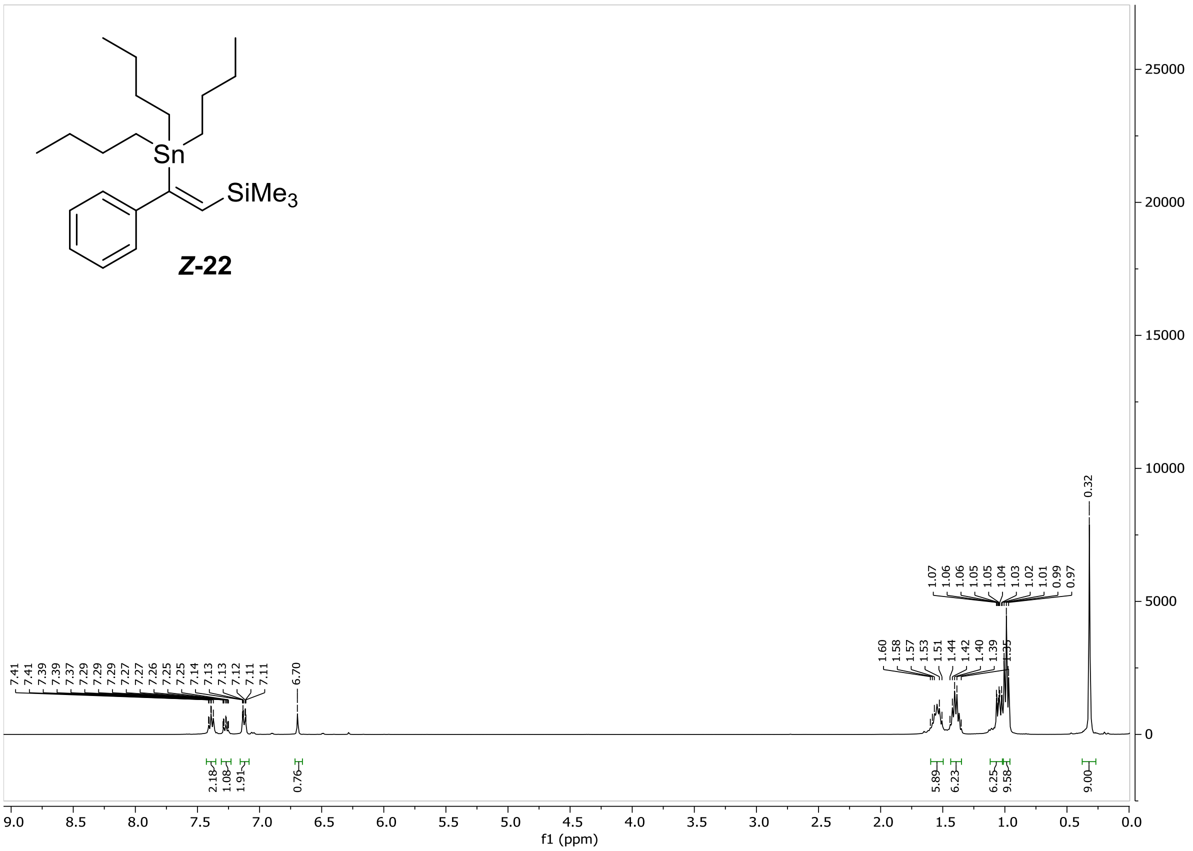

^{1}H NMR (400 MHz, $CDCl_3$)

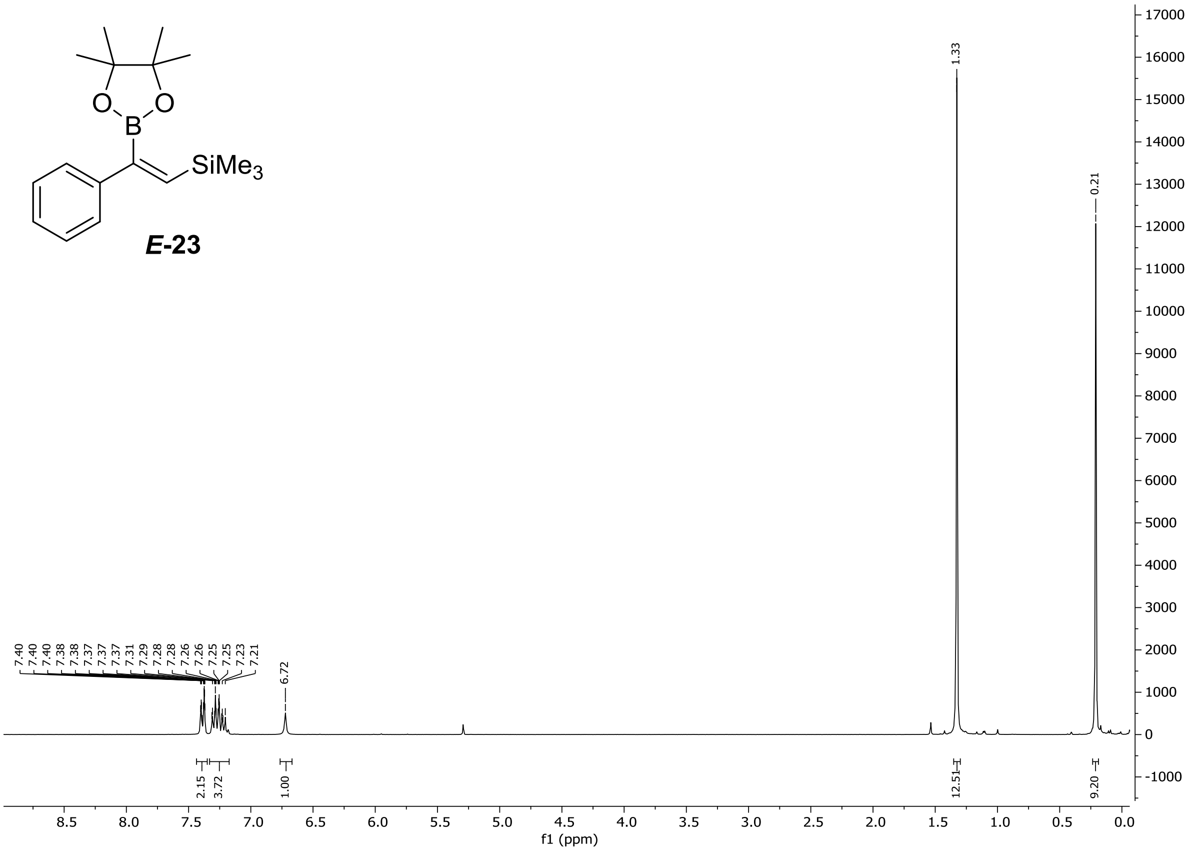

^{1}H NMR (300 MHz, $CDCl_3$)

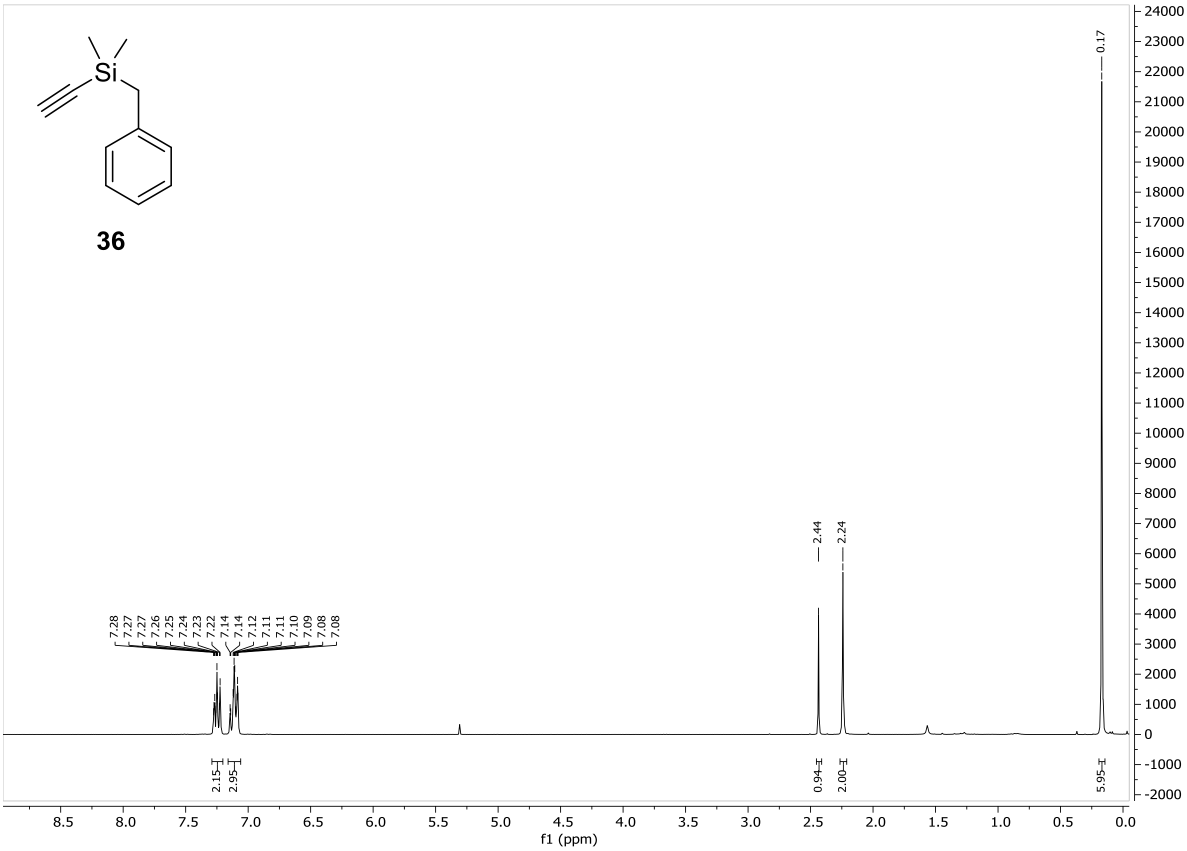

^{1}H NMR (600 MHz, $CDCl_3$)

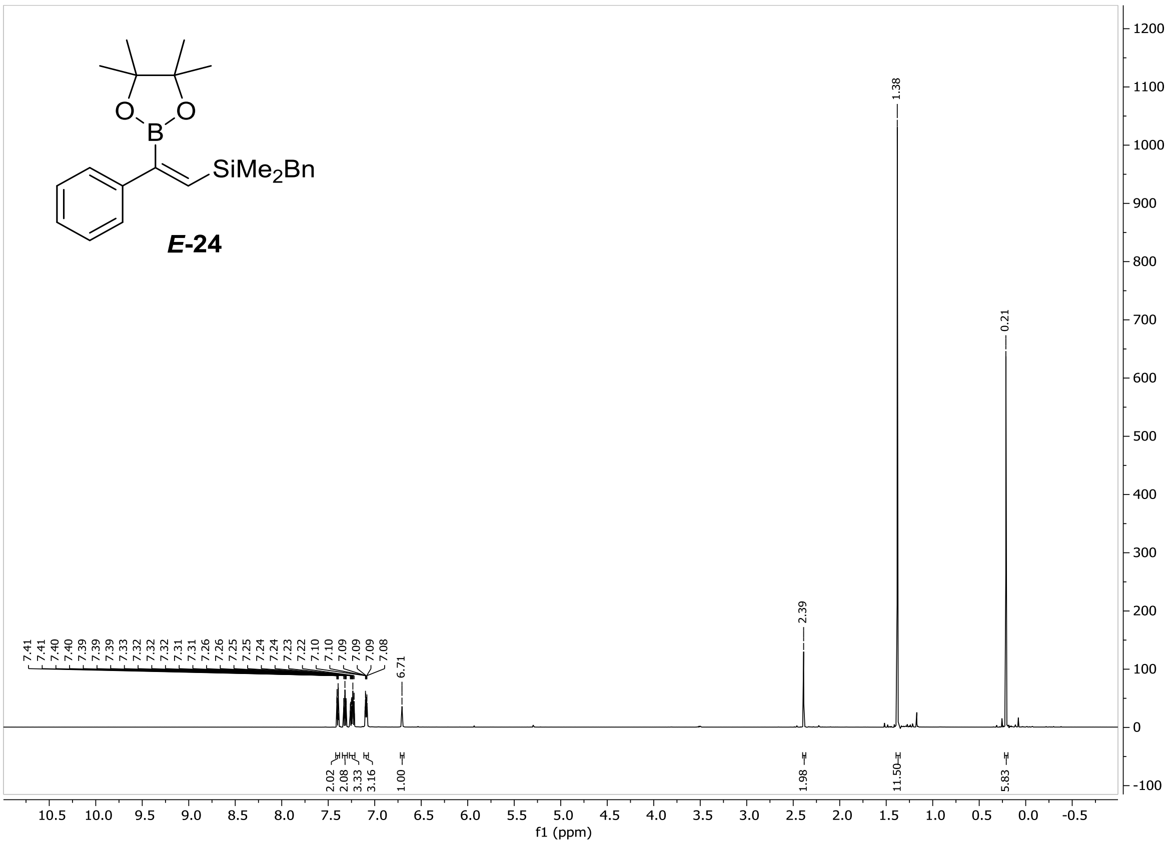

^{13}C NMR (151 MHz, $CDCl_3$)

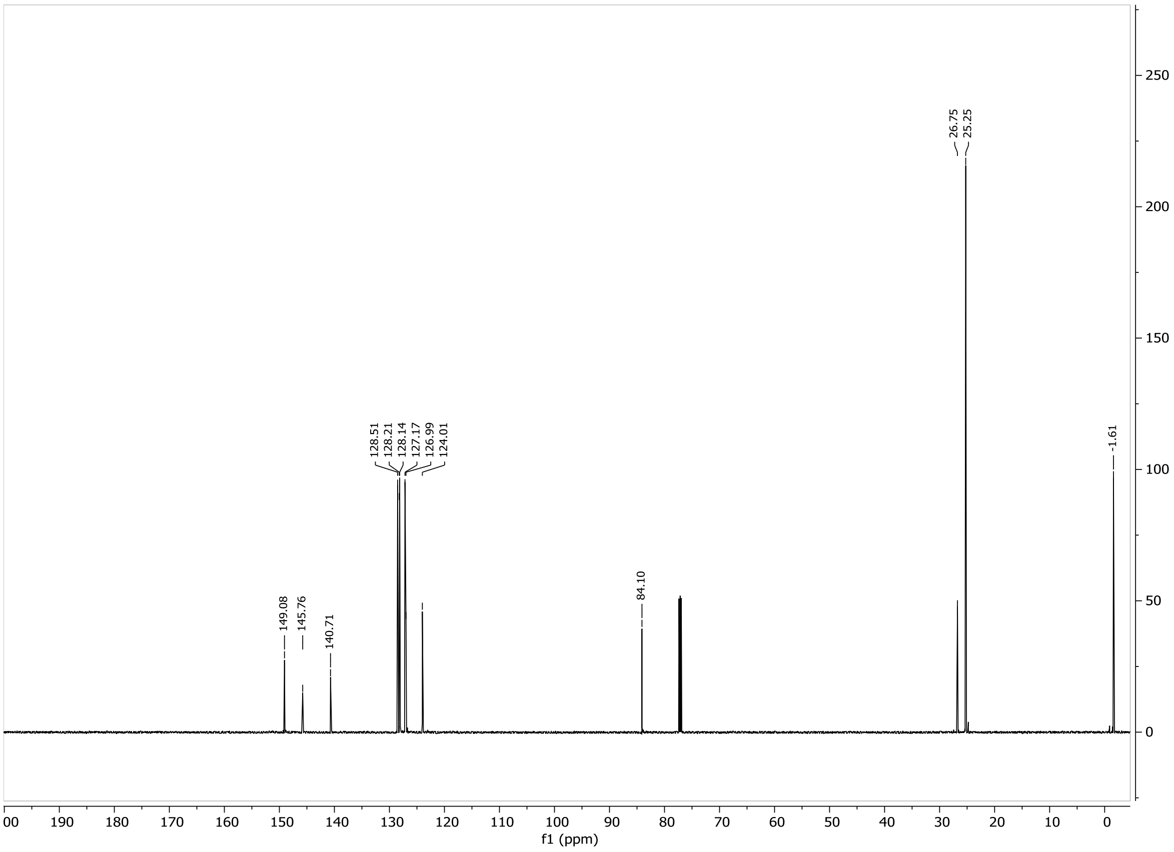

^{1}H NMR (600 MHz, $CDCl_3$)

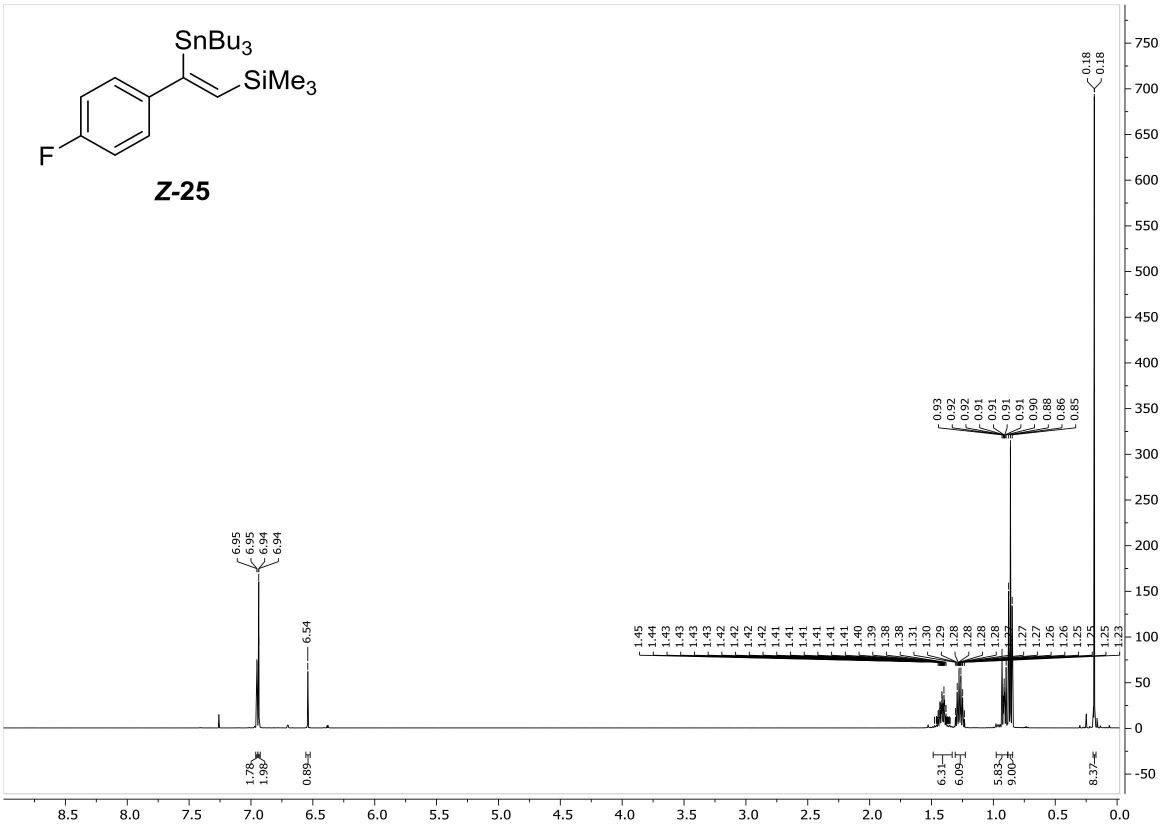

^{13}C NMR (151 MHz, $CDCl_3$)

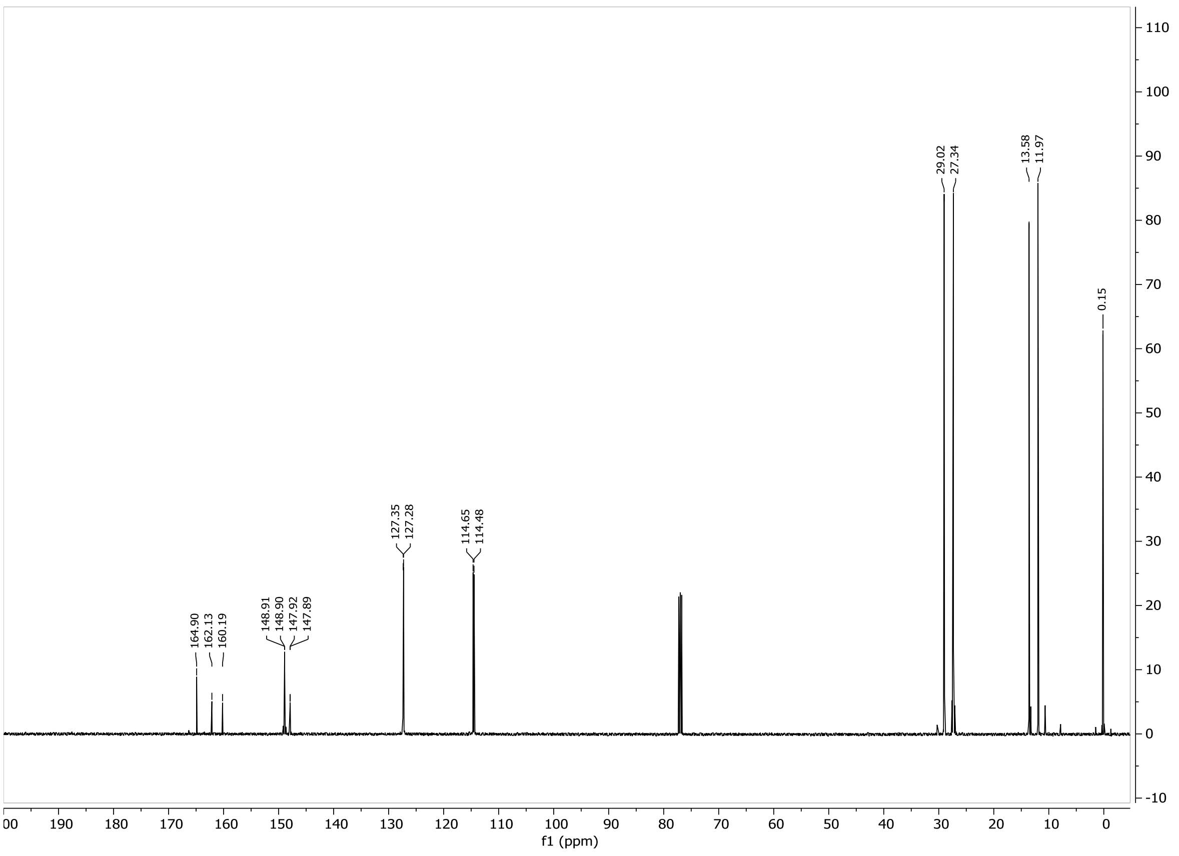

^{19}F NMR (470 MHz, $CDCl_3$)

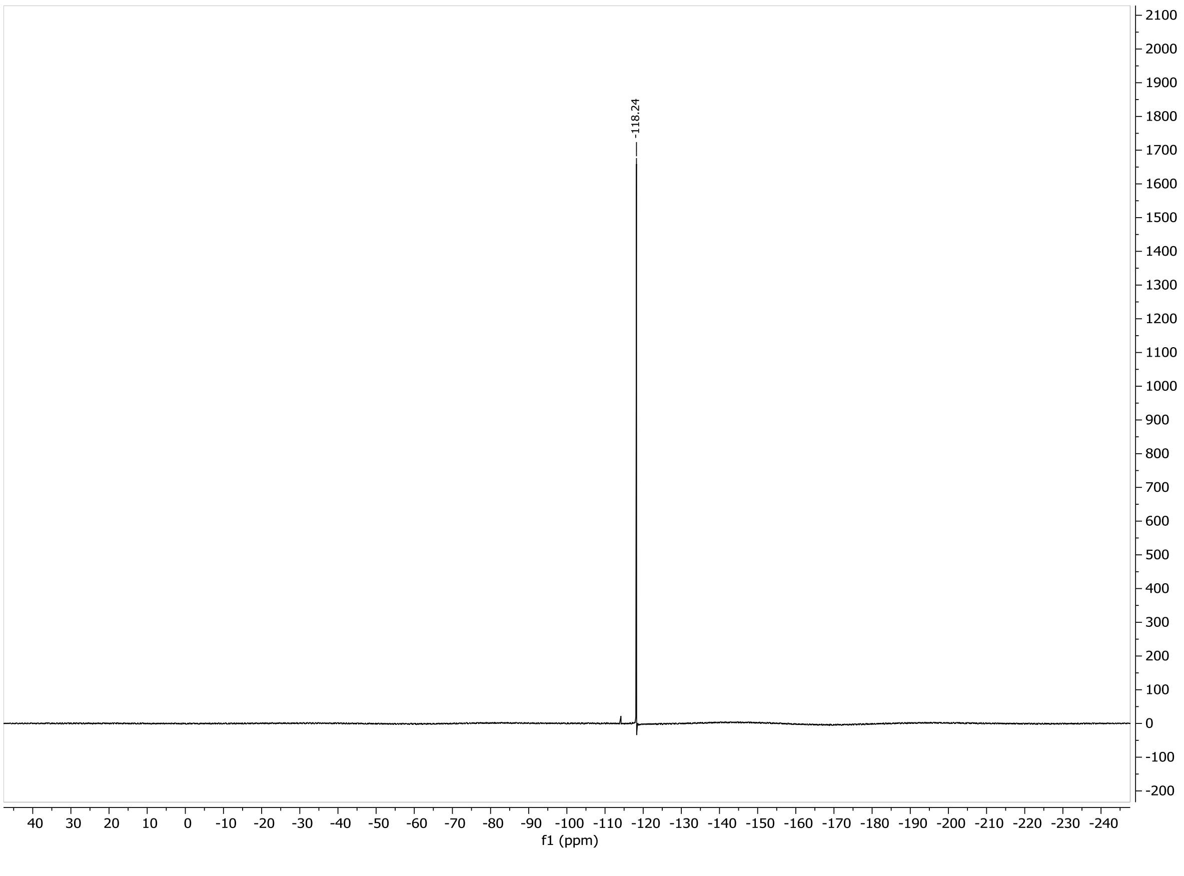

^1H NMR (500 MHz, $CDCl_3$)

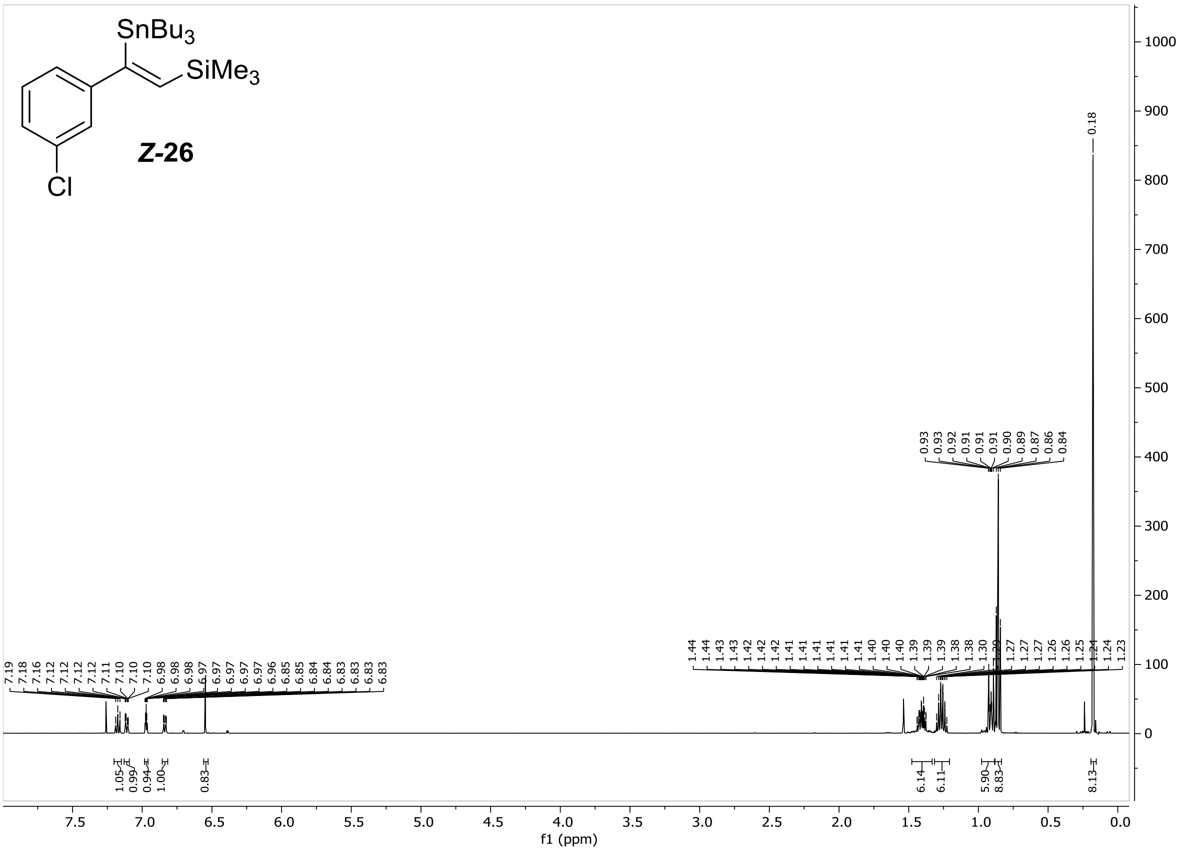

^{13}C NMR (126 MHz, $CDCl_3$)

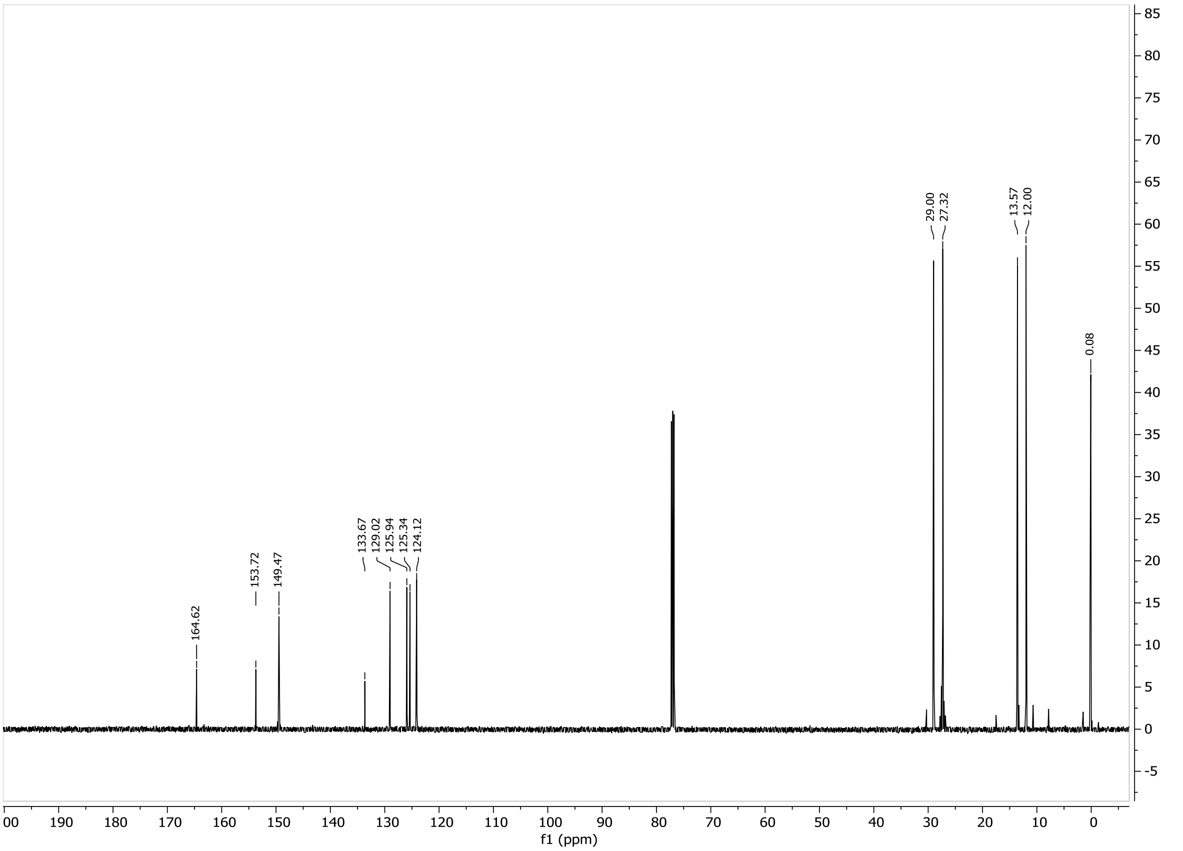

^{1}H NMR (400 MHz, $CDCl_3$)

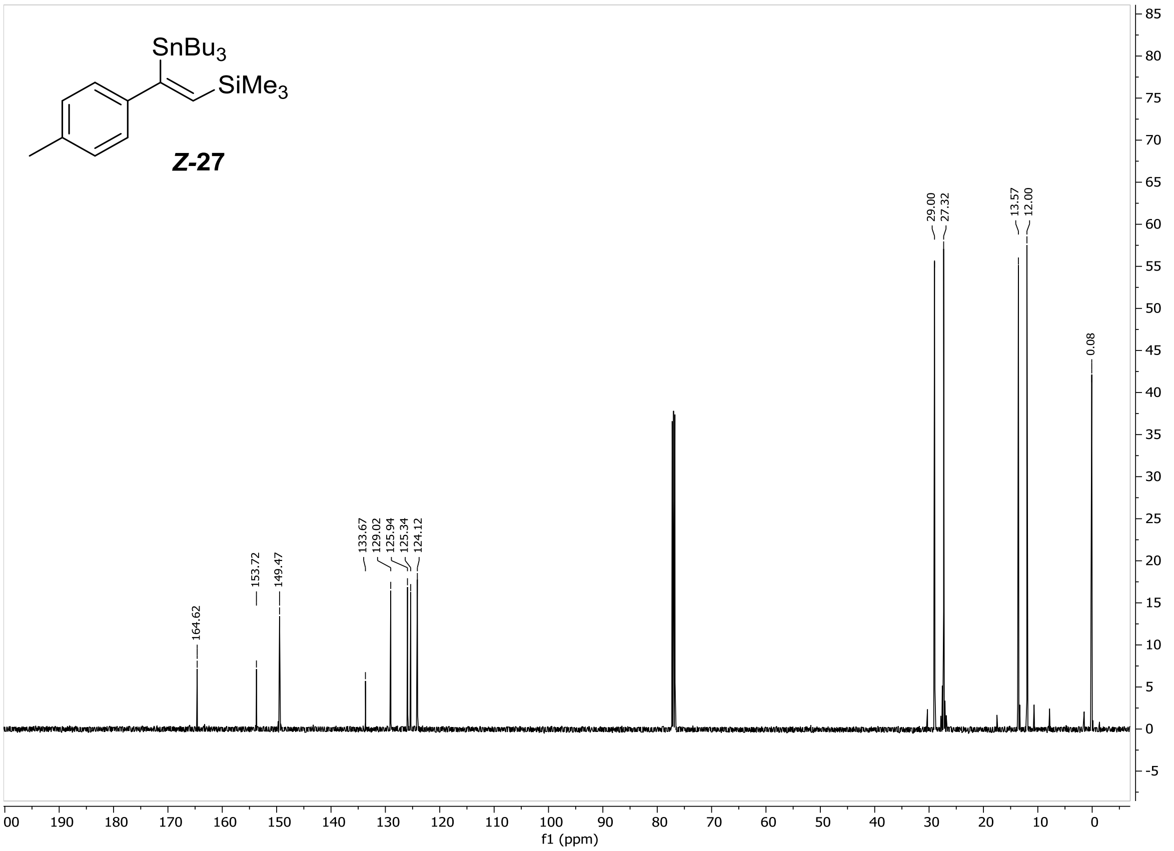

^{13}C NMR (101 MHz, $CDCl_3$)

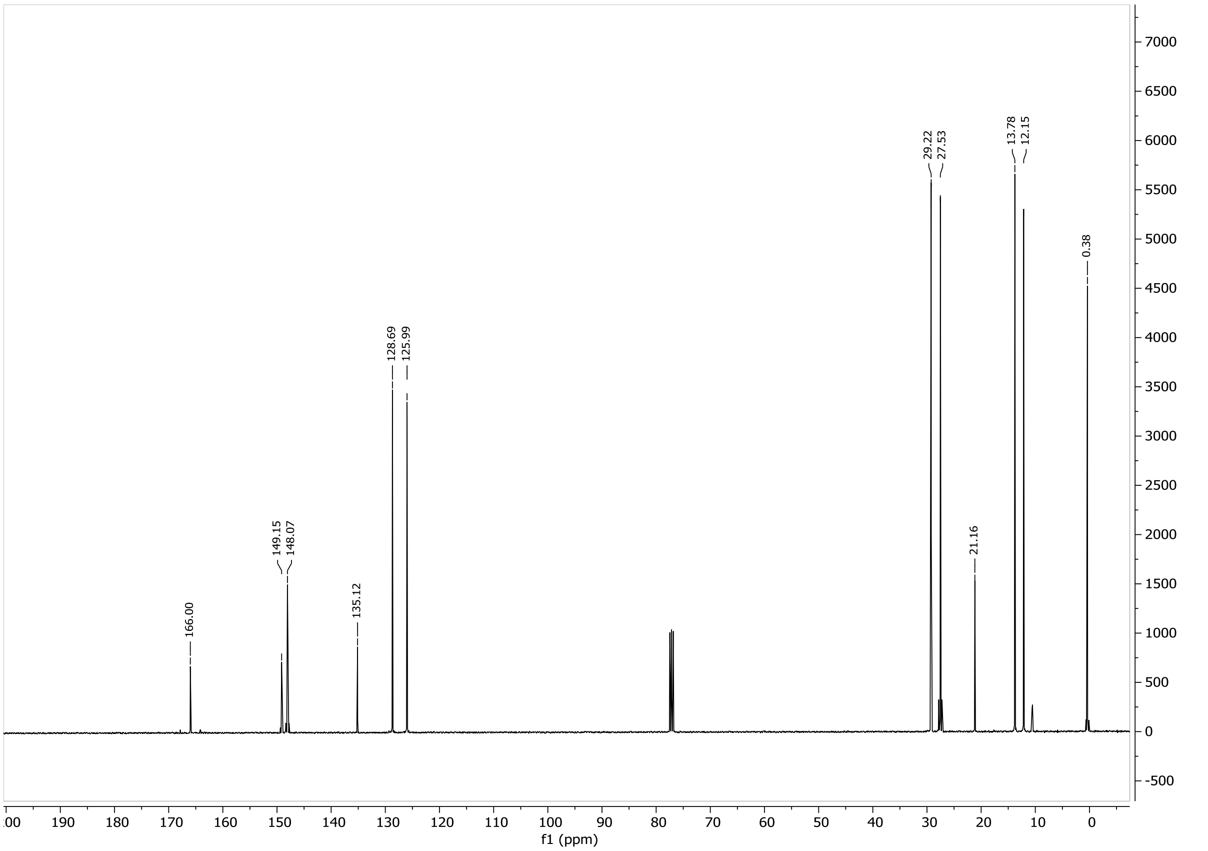

^{1}H NMR (300 MHz, $CDCl_3$)

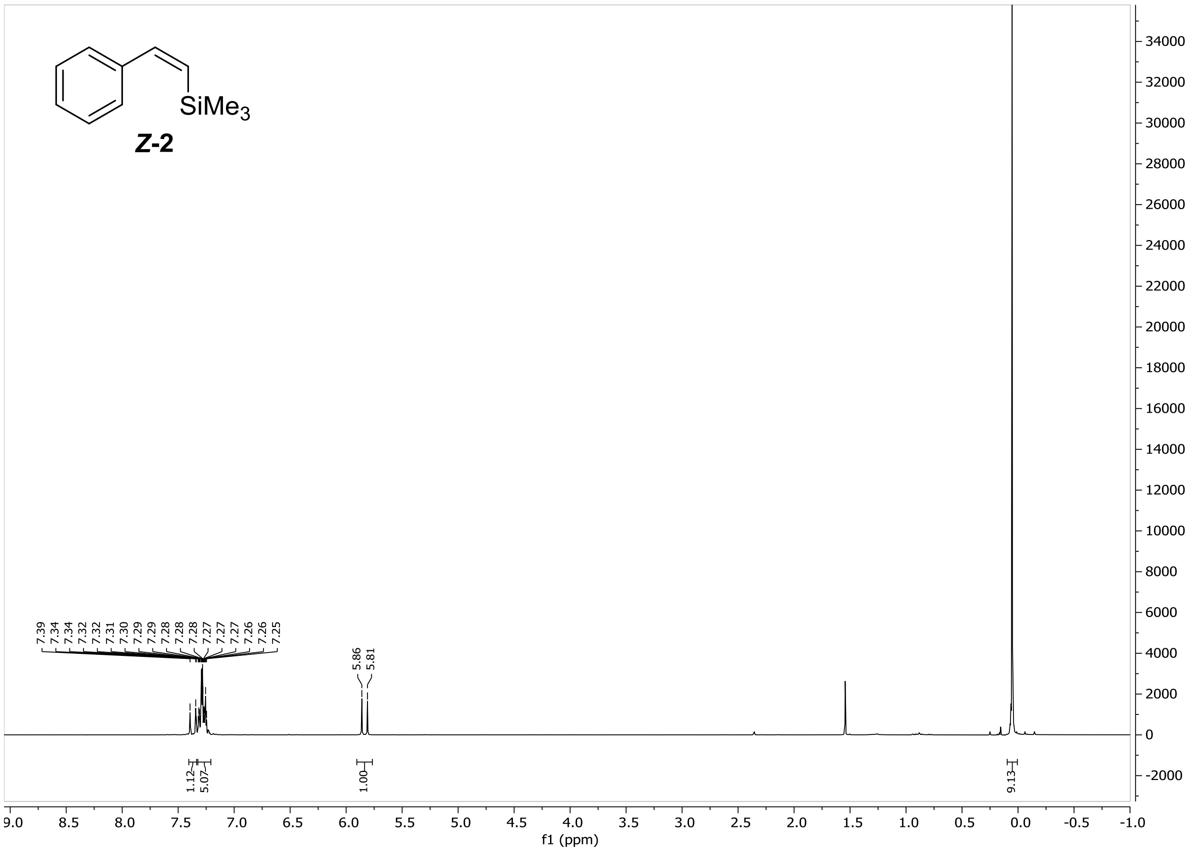

^{1}H NMR (400 MHz, $CDCl_3$)

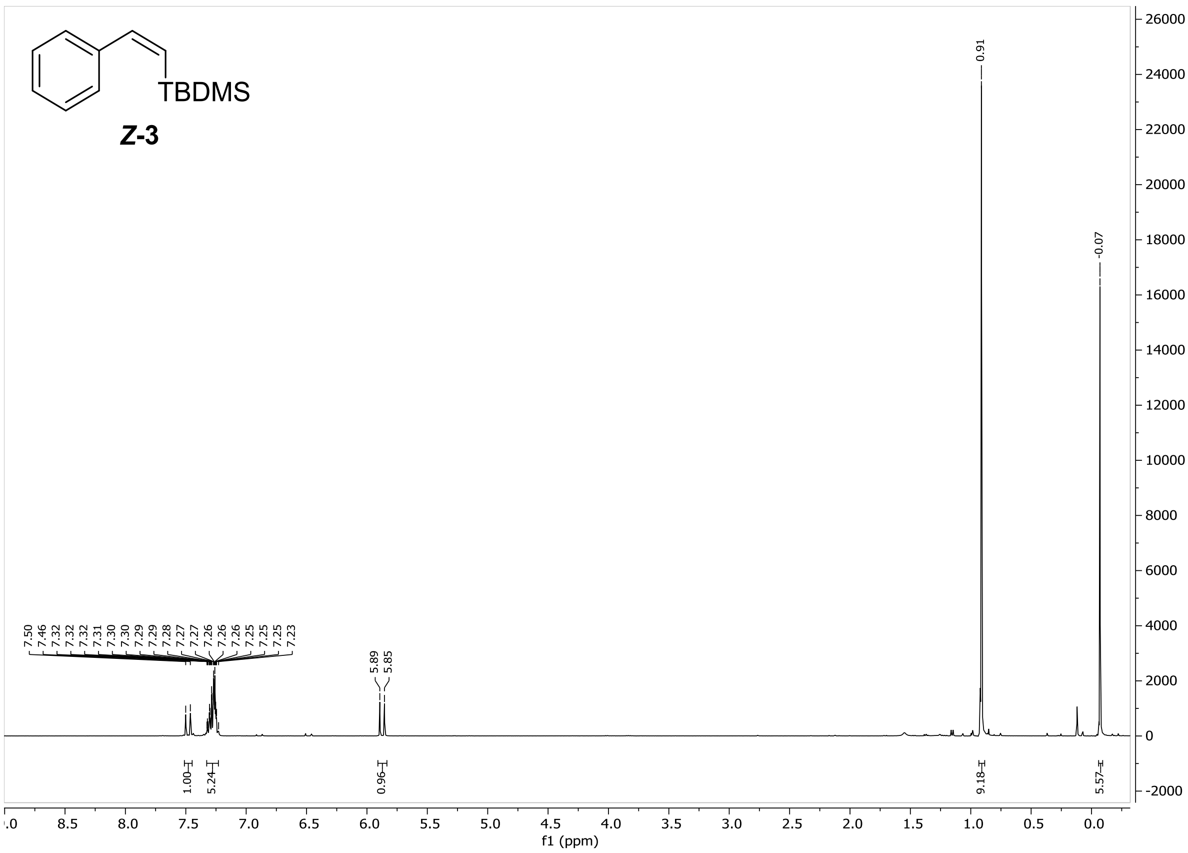

^{1}H NMR (600 MHz, CD_2Cl_2)

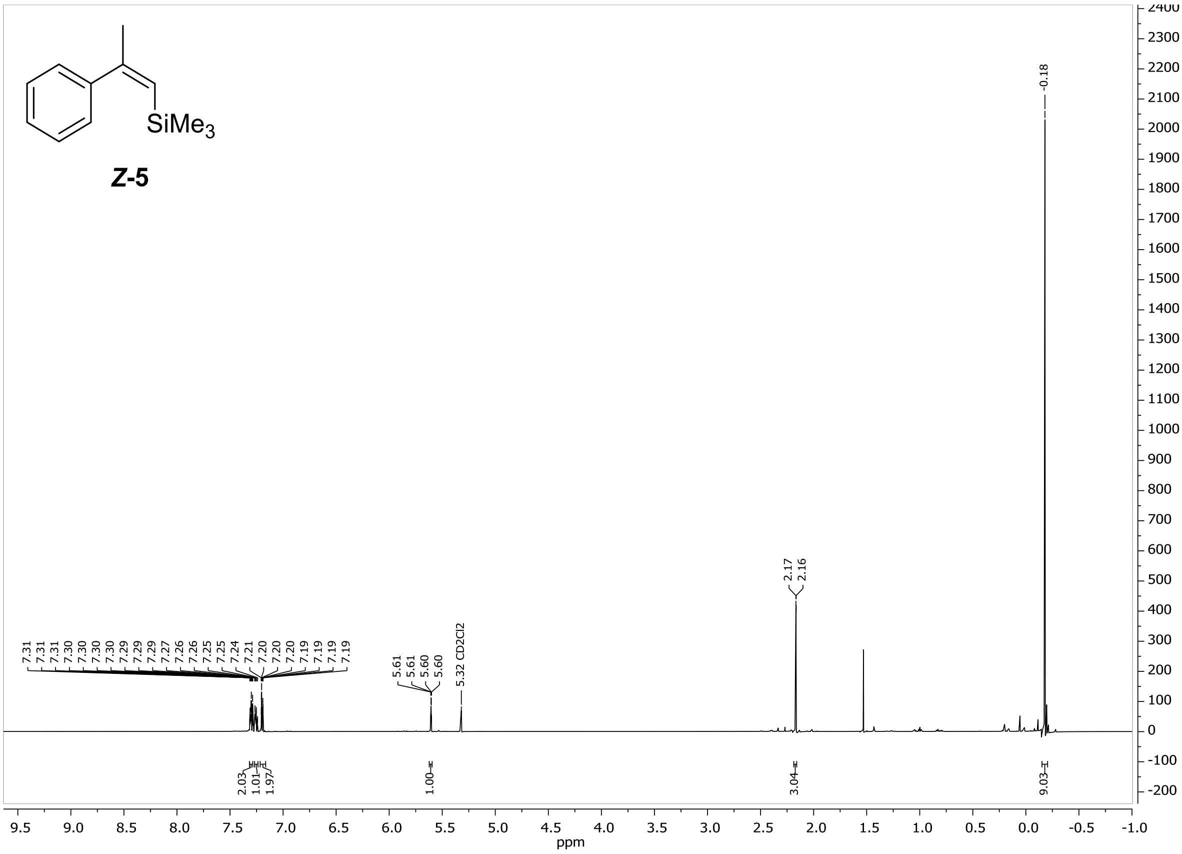

^{13}C NMR (151 MHz, CD_2Cl_2)

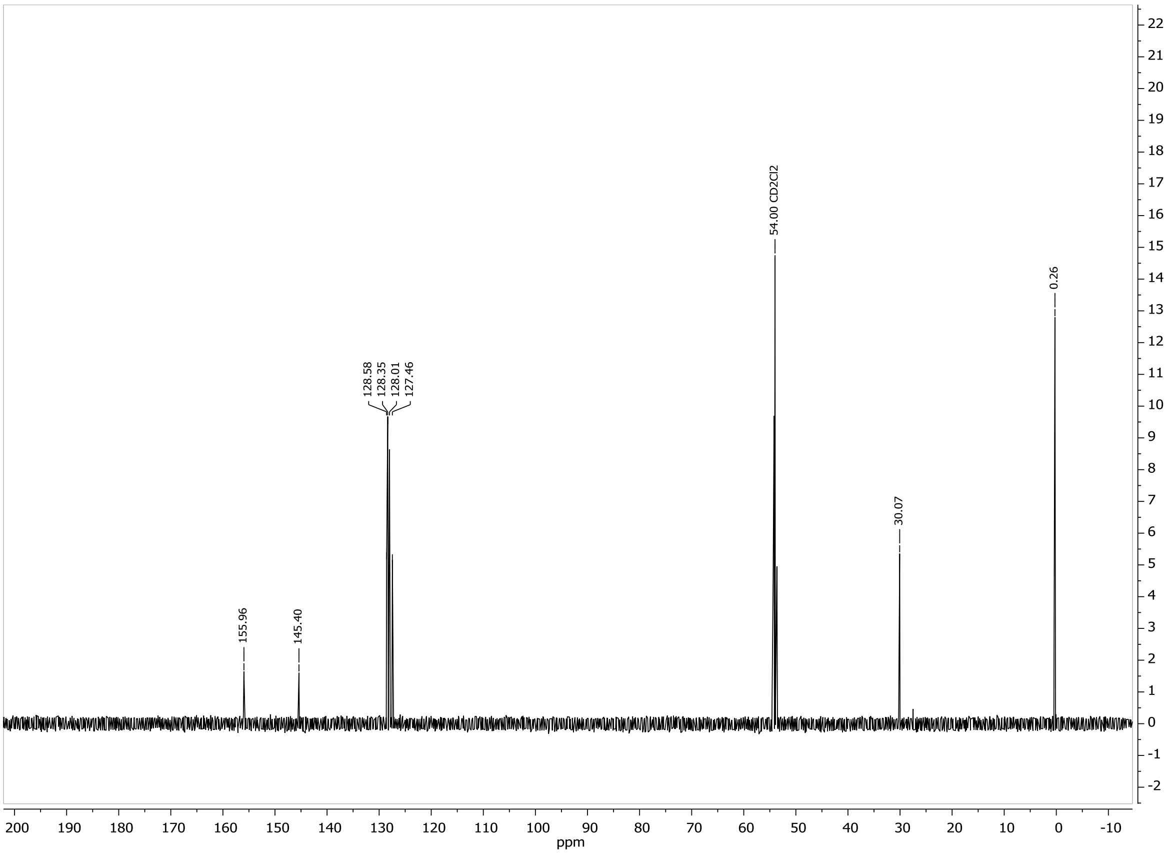

^{1}H NMR (400 MHz, $CDCl_3$)

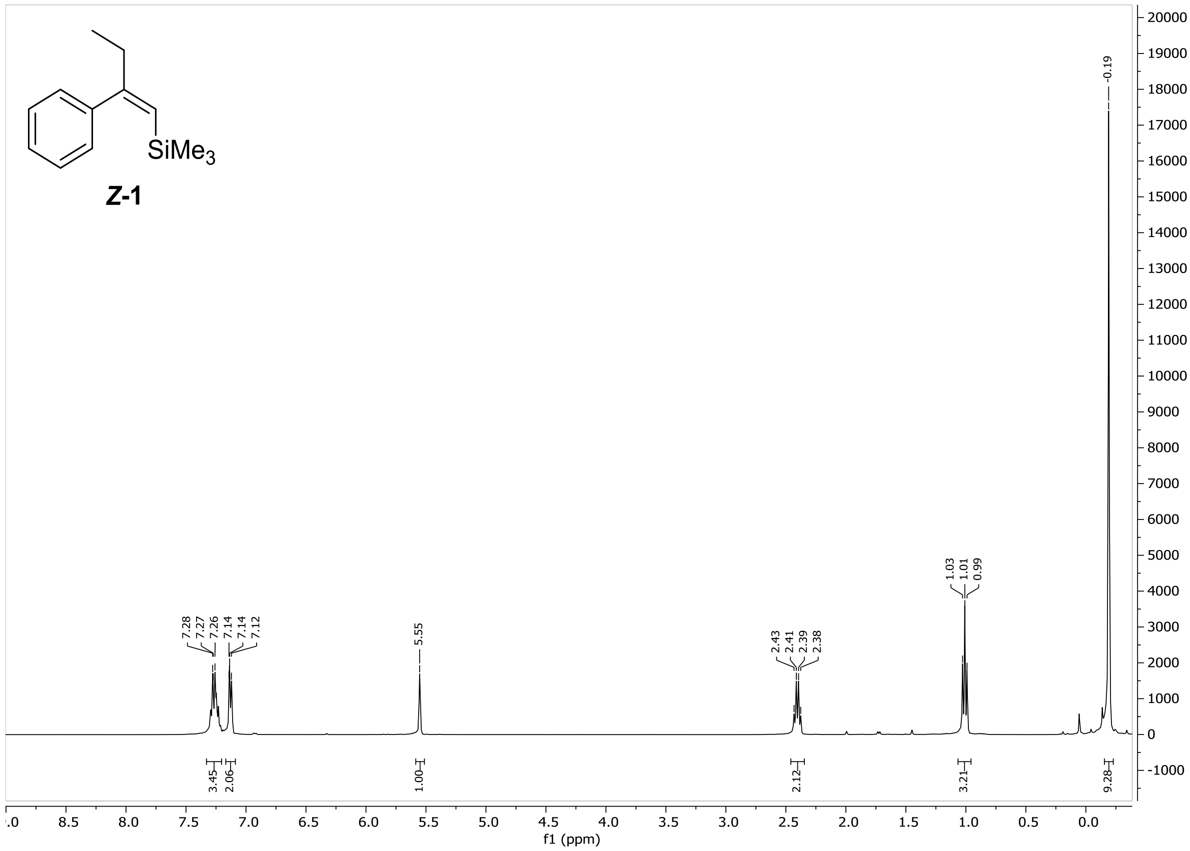

^{1}H NMR (600 MHz, CD_2Cl_2)

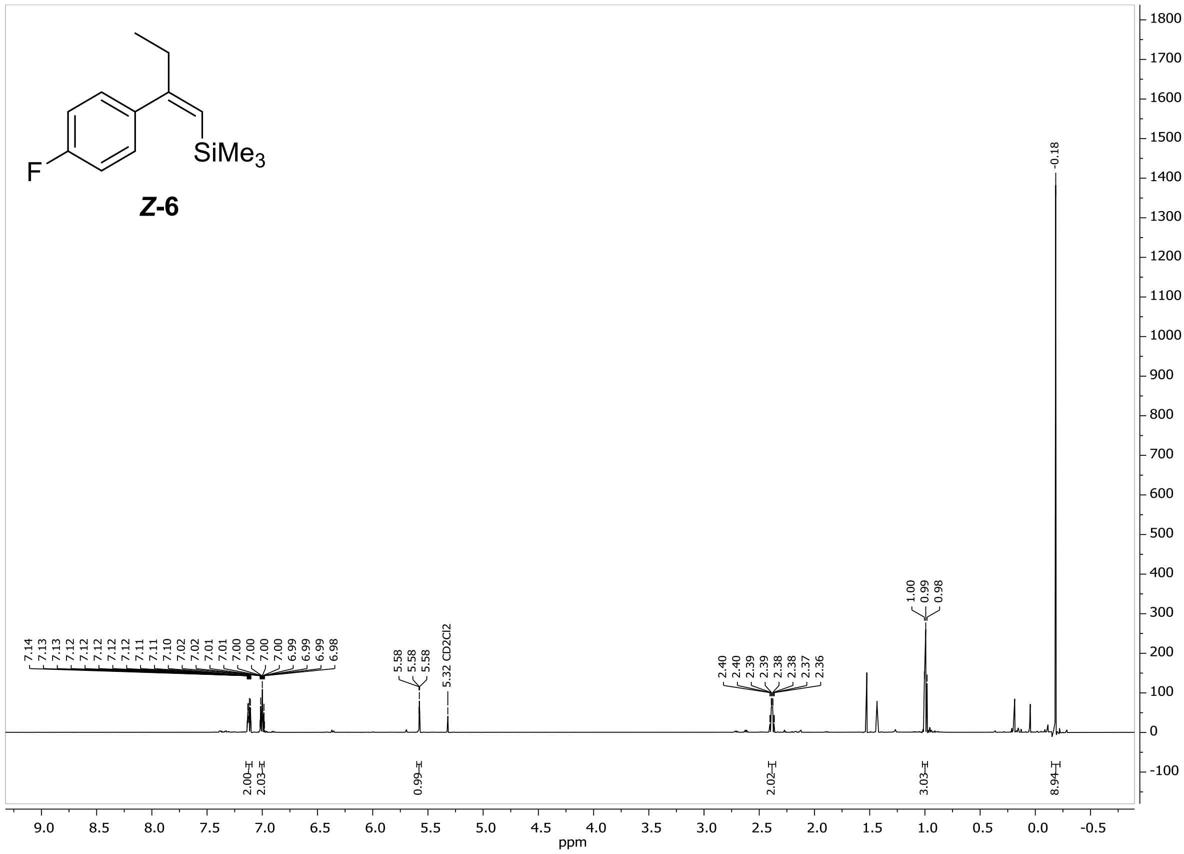

^{13}C NMR (151 MHz, CD_2Cl_2)

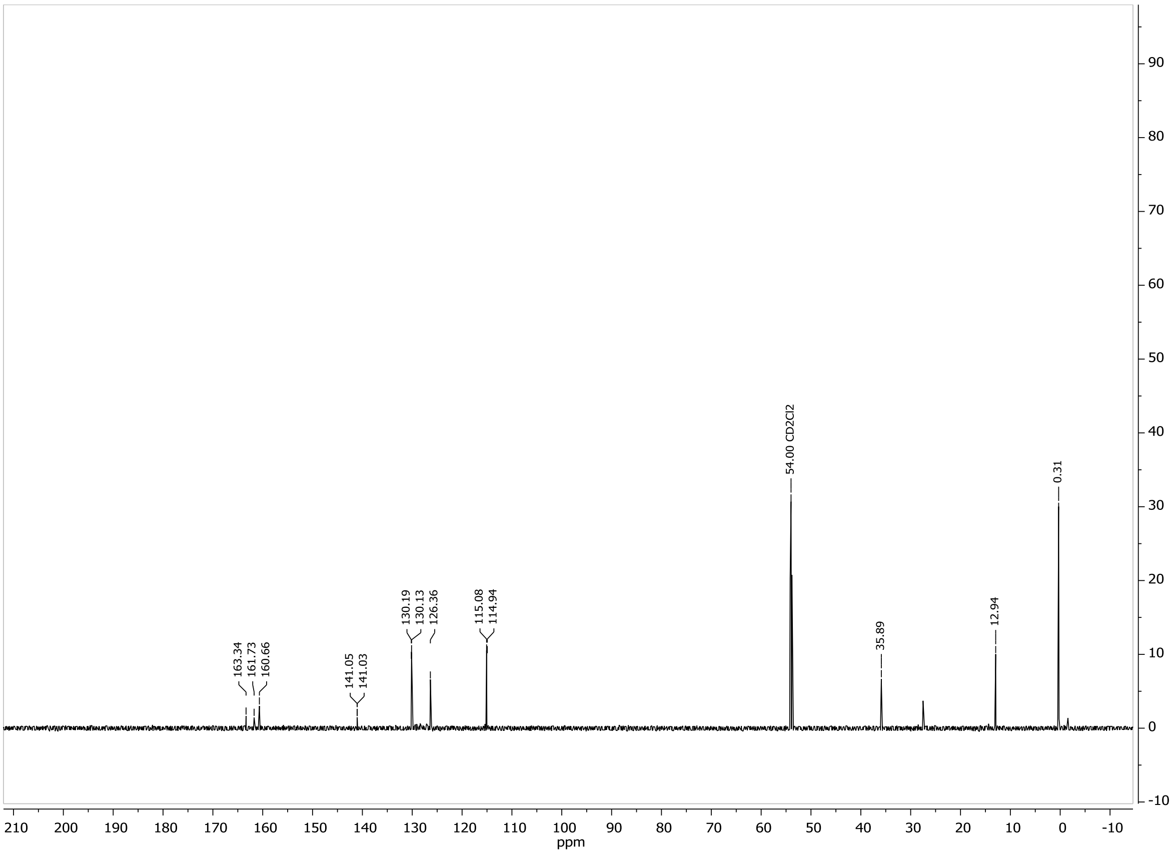

^{19}F NMR (564 MHz, CD_2Cl_2)

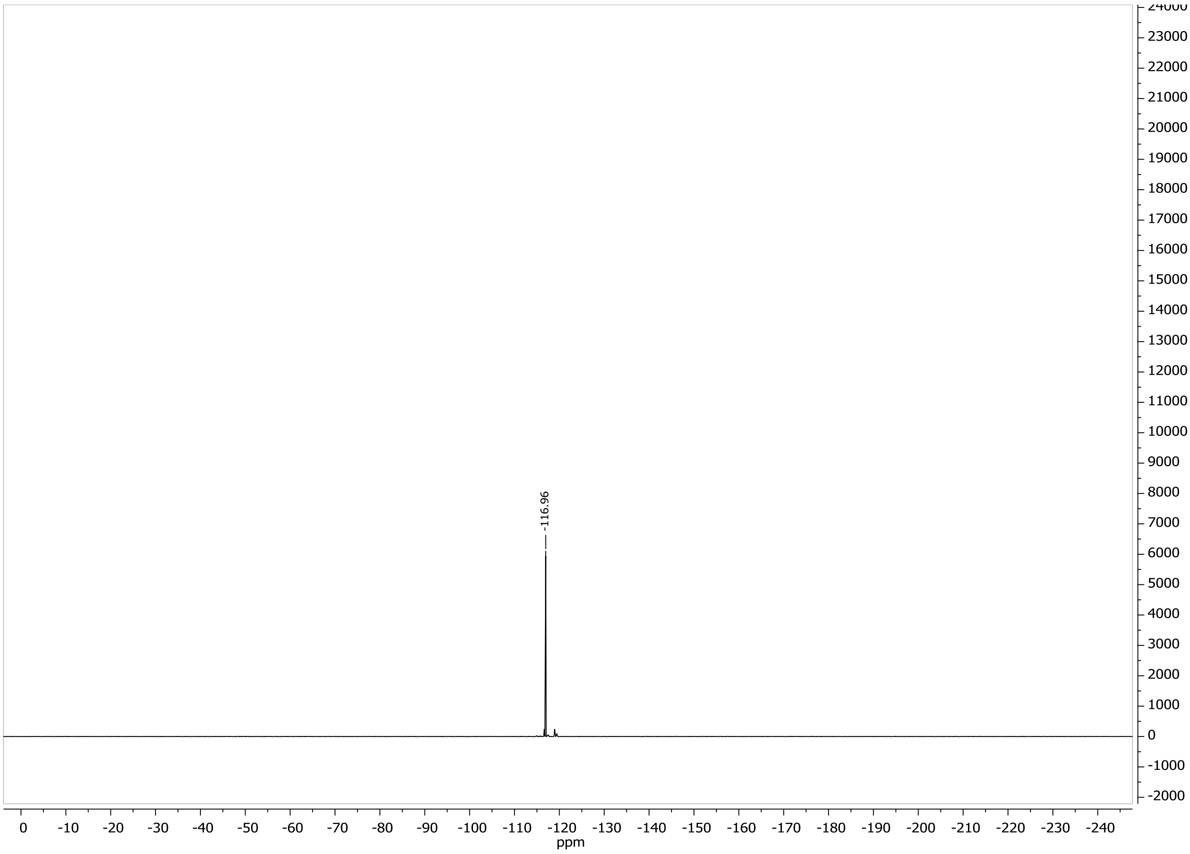

^{1}H NMR (600 MHz, CD_2Cl_2)

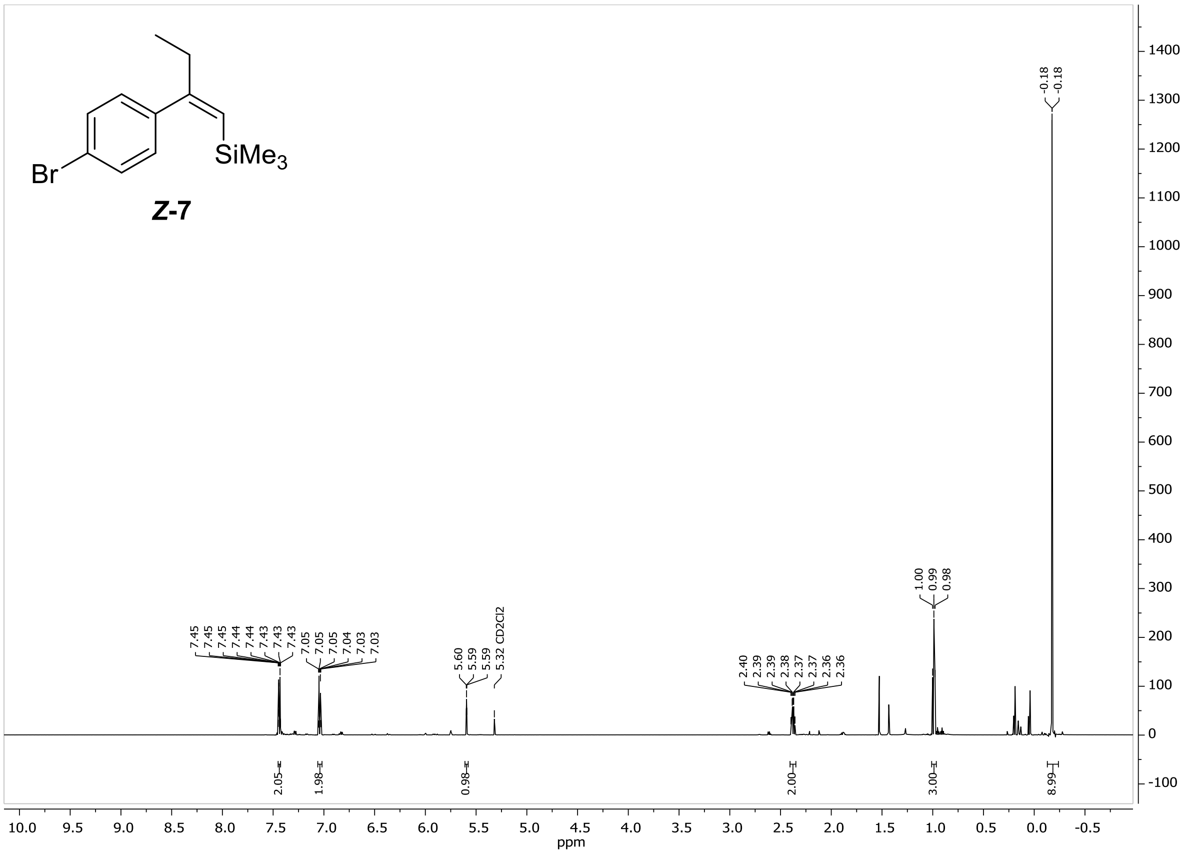

^{13}C NMR (151 MHz, CD_2Cl_2)

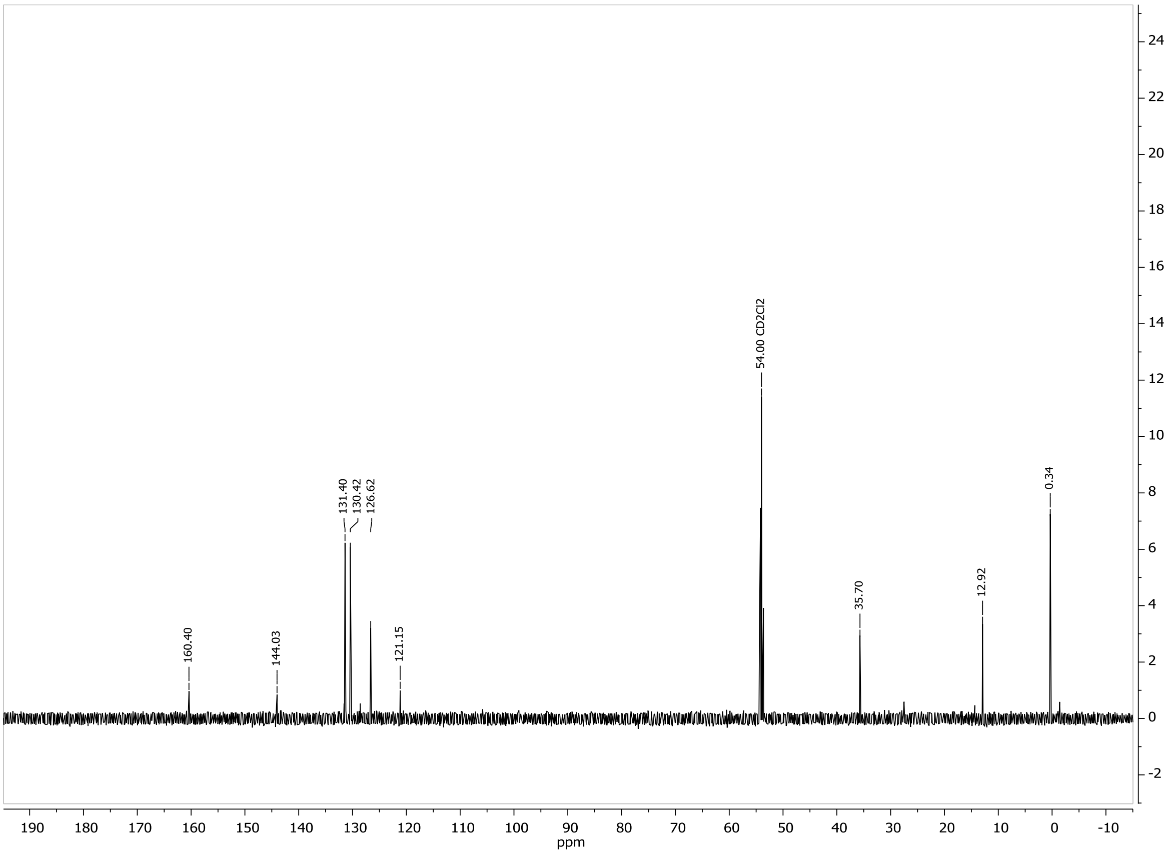

^1H NMR (500 MHz, CD_2Cl_2)

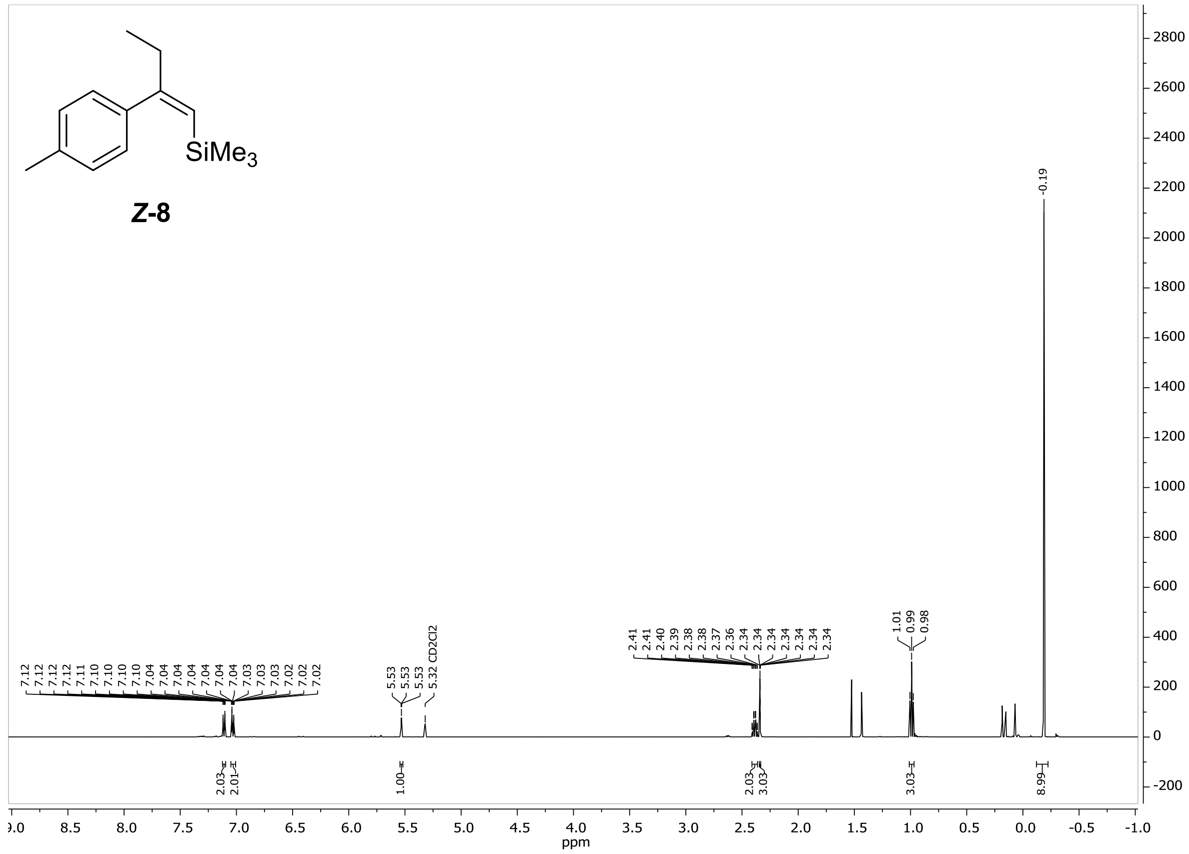

^{13}C NMR (126 MHz, CD_2Cl_2)

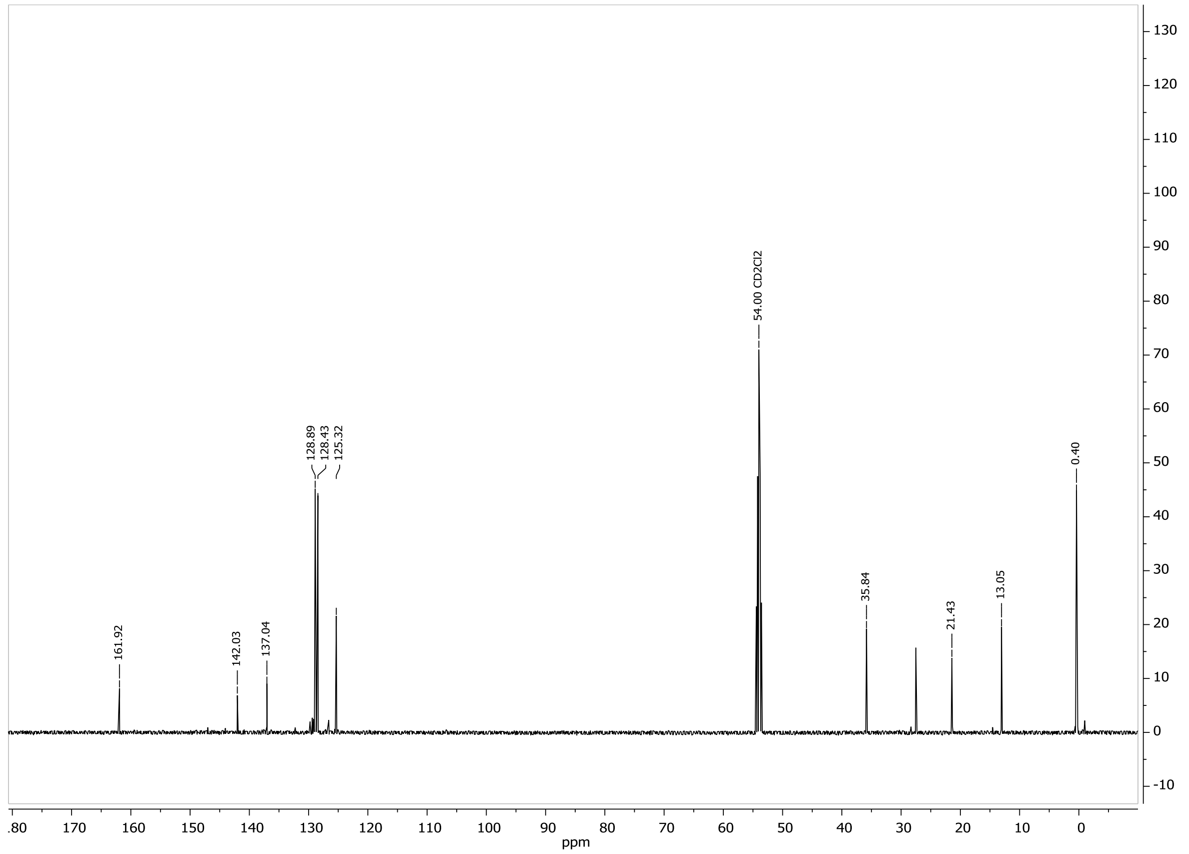

^{1}H NMR (600 MHz, CD_2Cl_2)

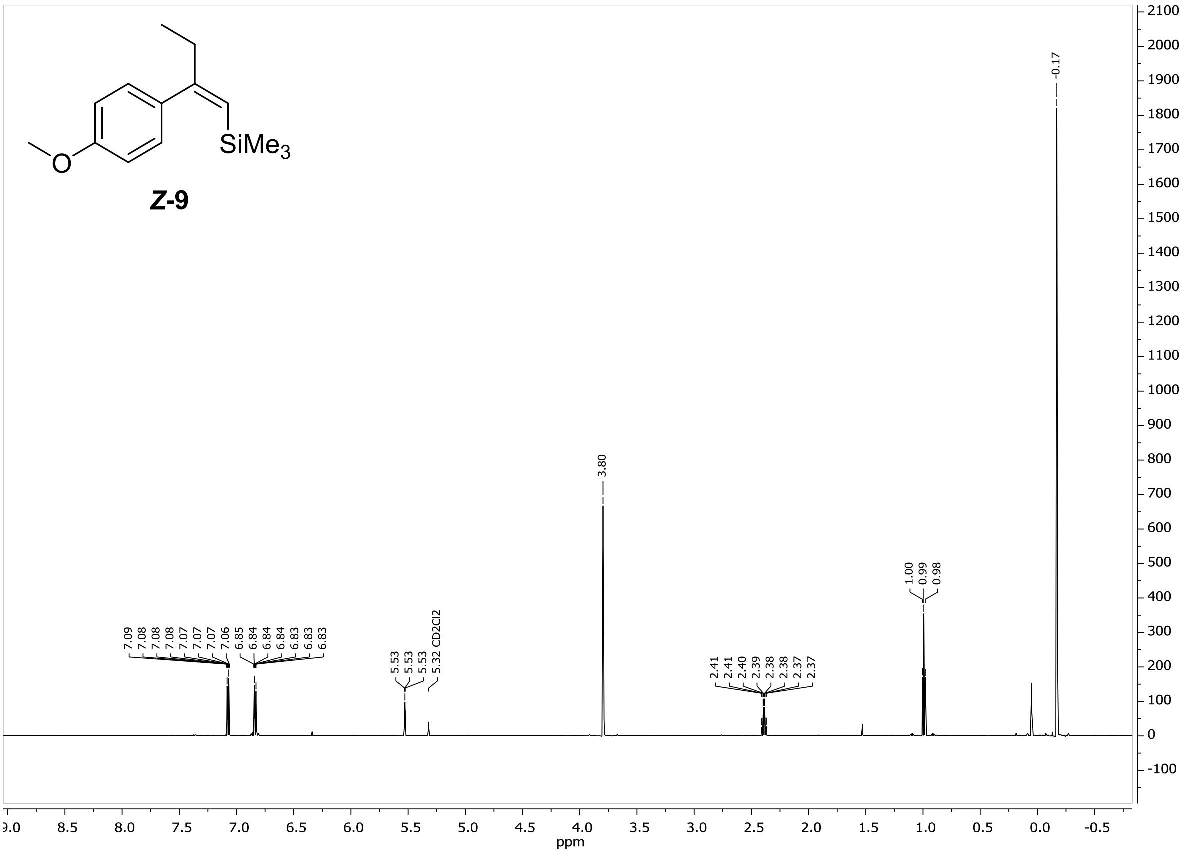

^{13}C NMR (151 MHz, CD_2Cl_2)

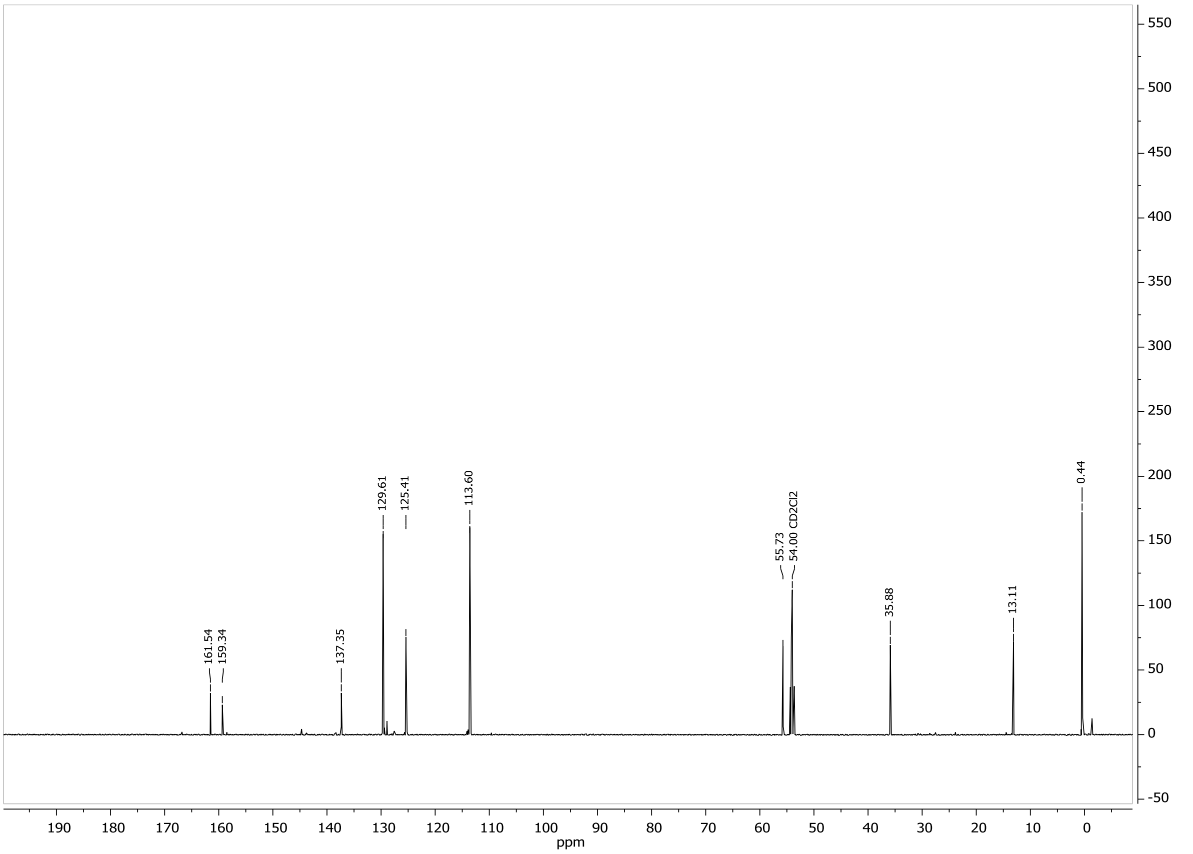

^{1}H NMR (600 MHz, CD_2Cl_2)

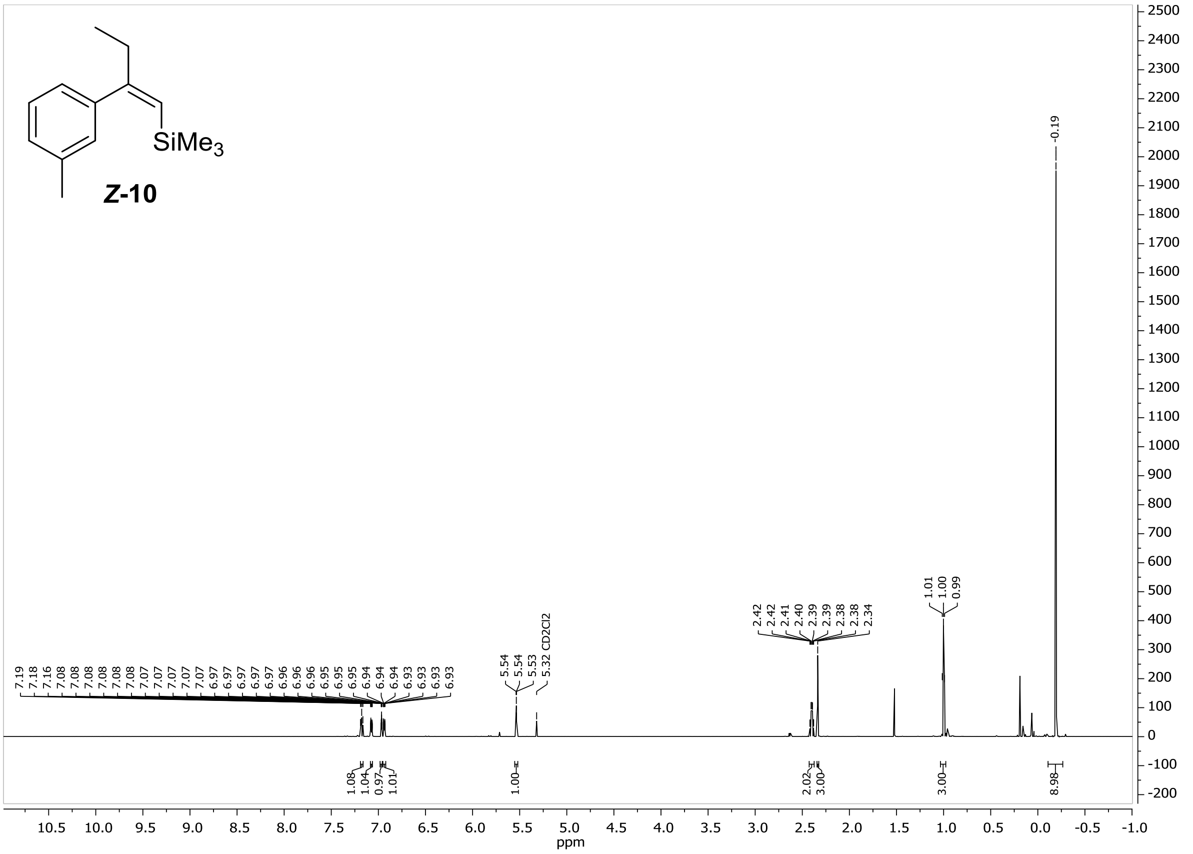

^{13}C NMR (151 MHz, CD_2Cl_2)

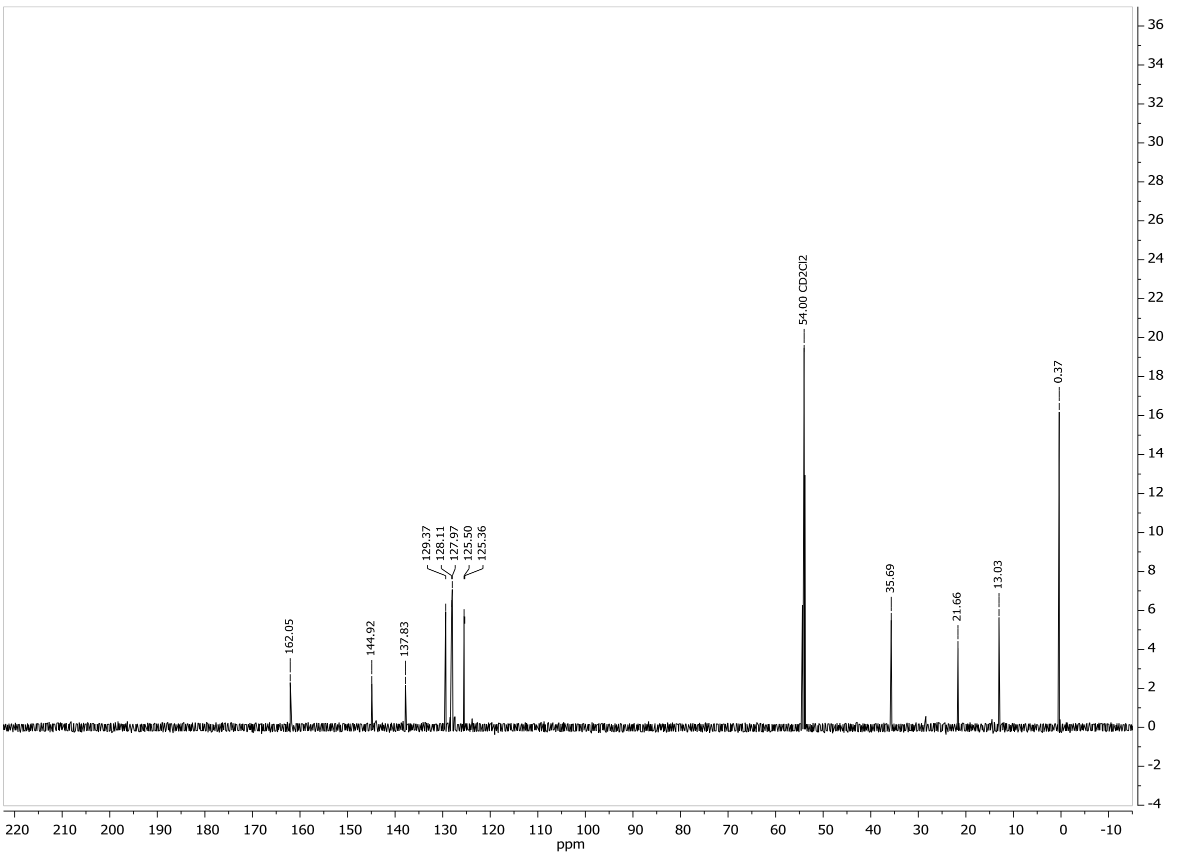

^1H NMR (CD_2Cl_2)

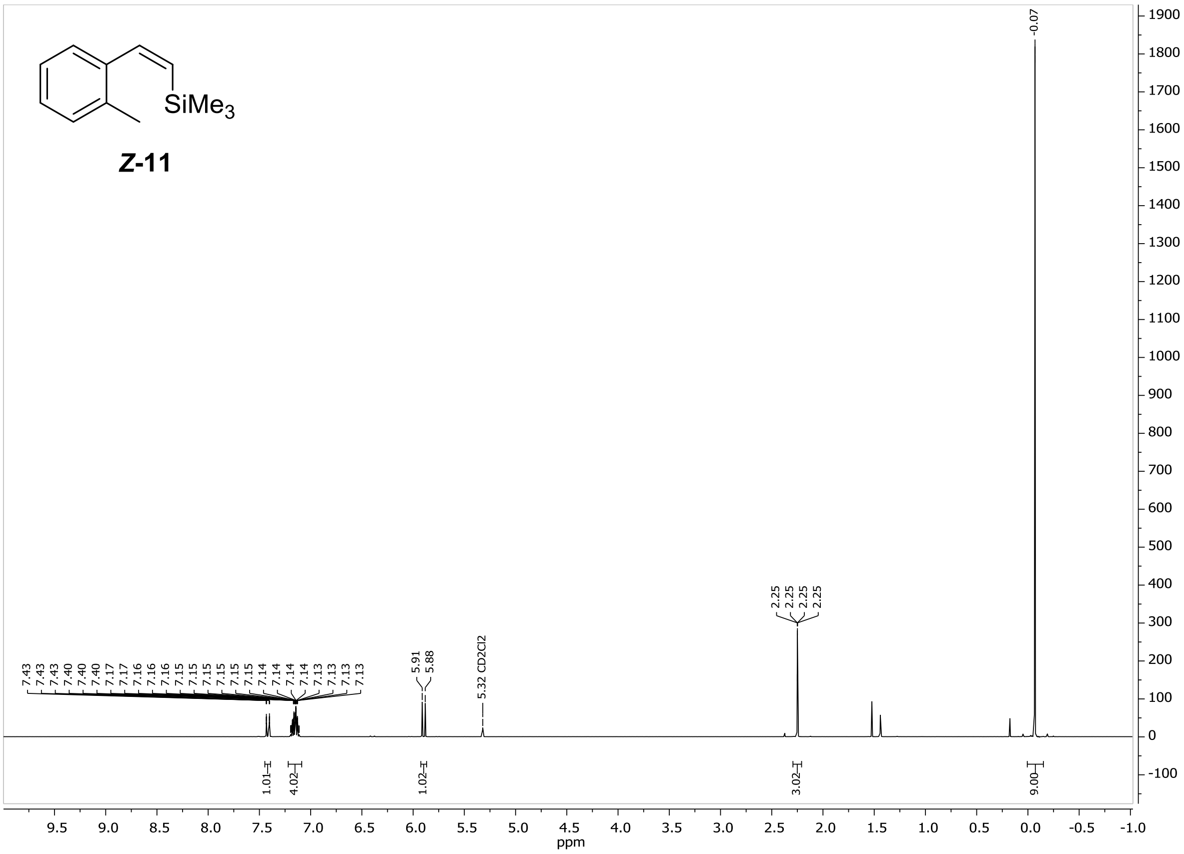

^{13}C NMR (126 MHz, CD_2Cl_2)

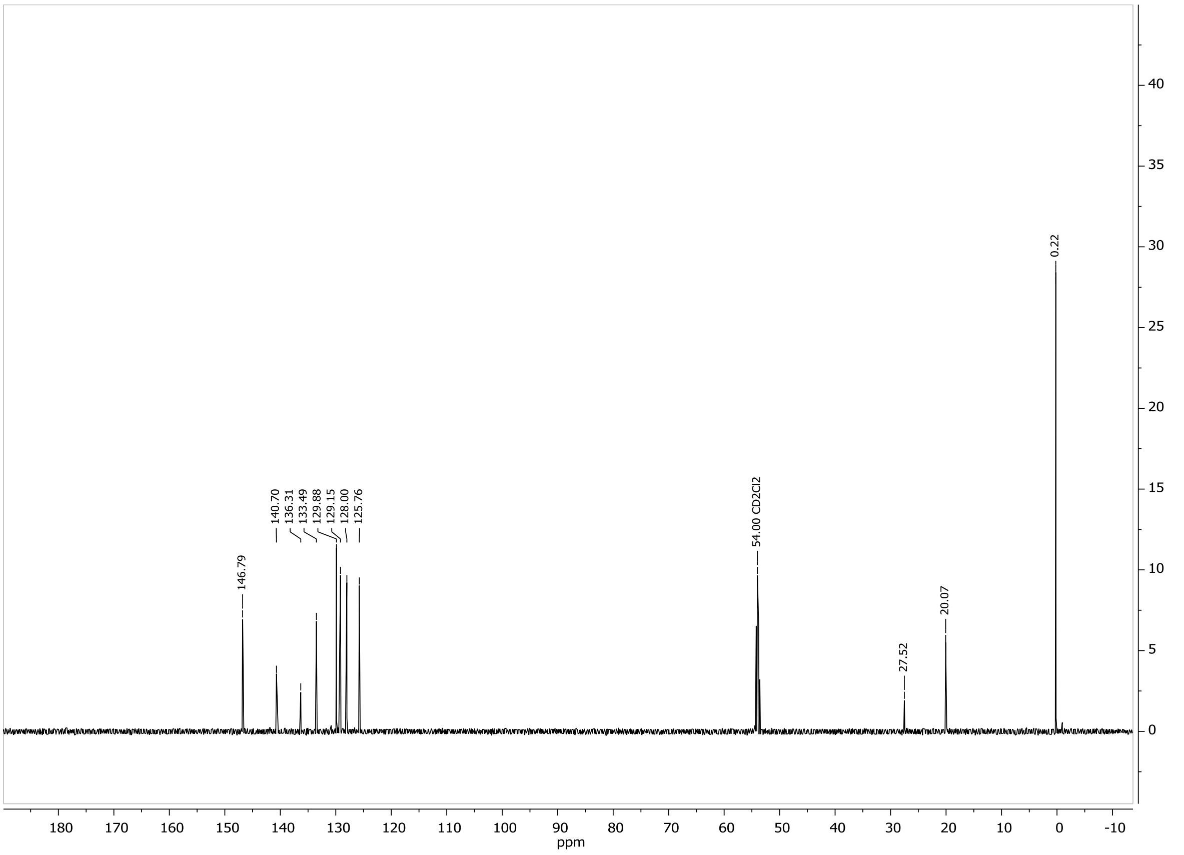

^{1}H NMR (500 MHz, CD_2Cl_2)

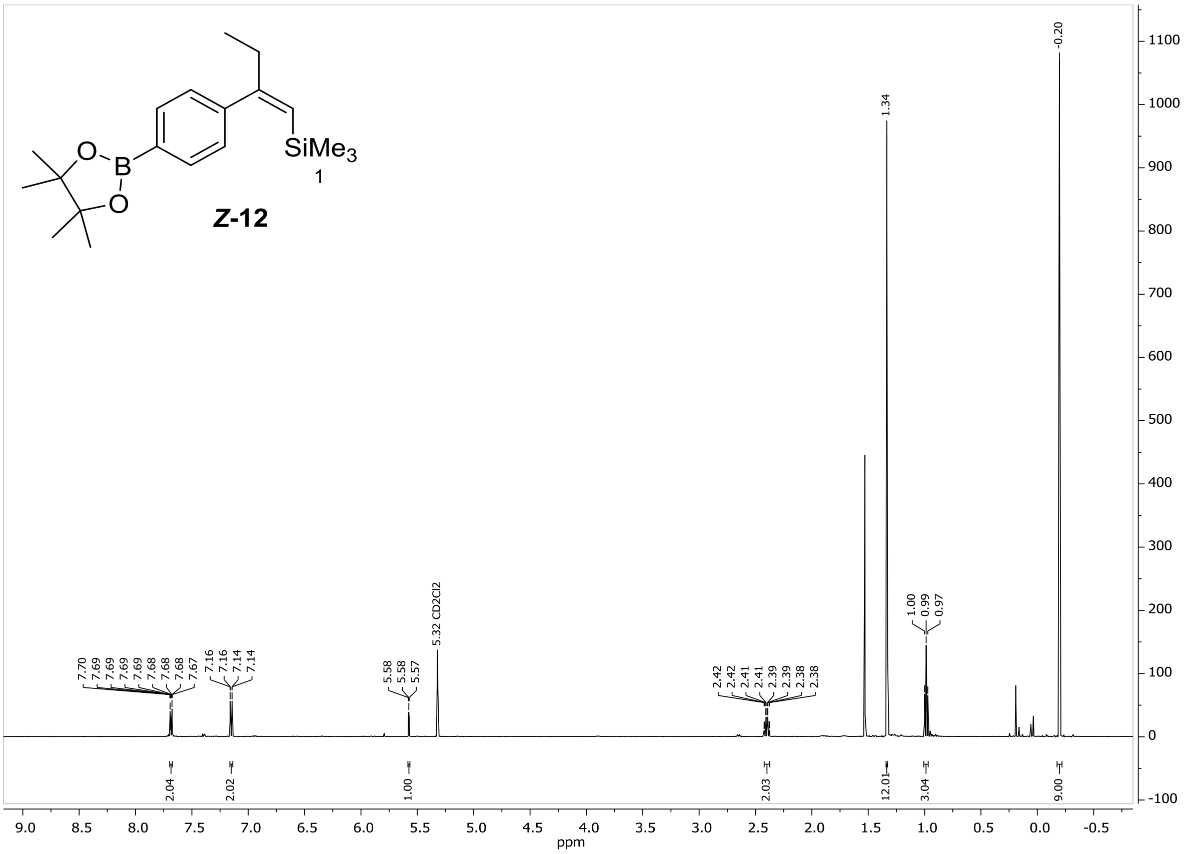

^{1}H NMR (500 MHz, CD_2Cl_2)

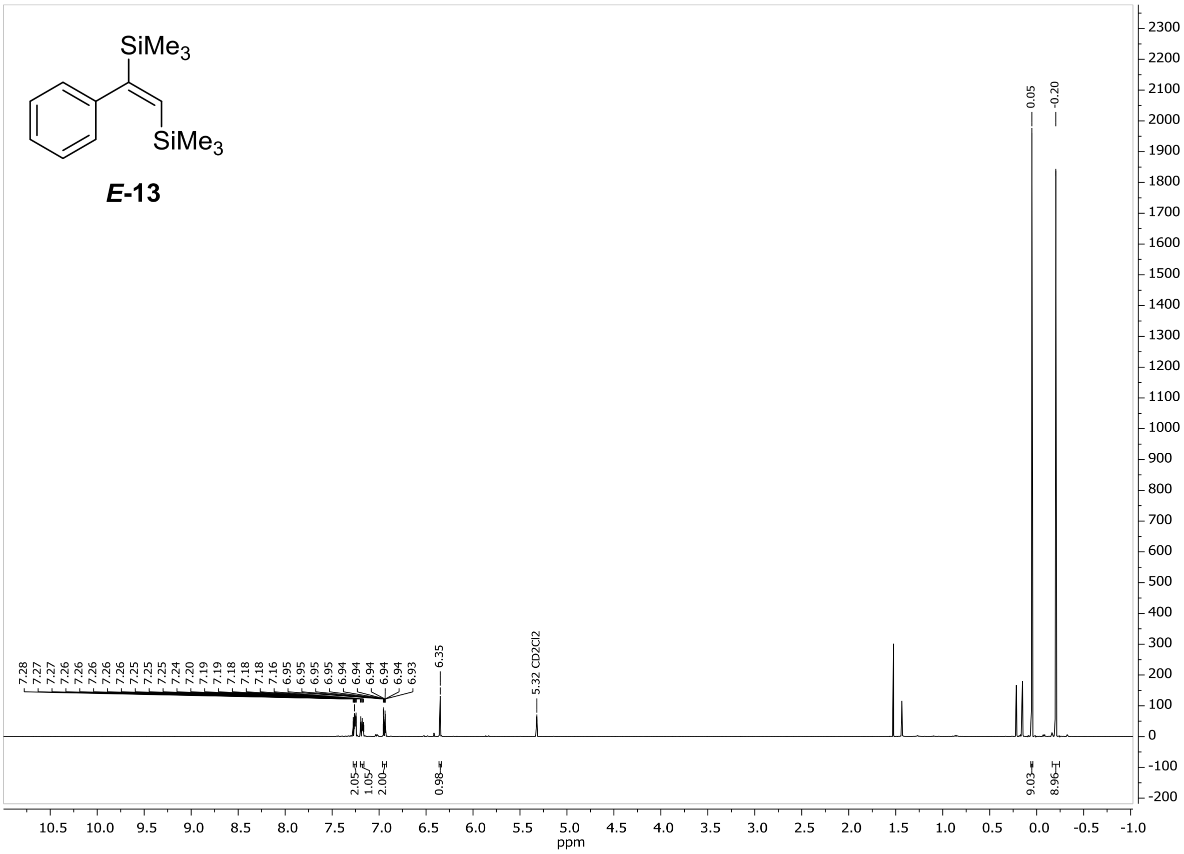

^{13}C NMR (126 MHz, CD_2Cl_2)

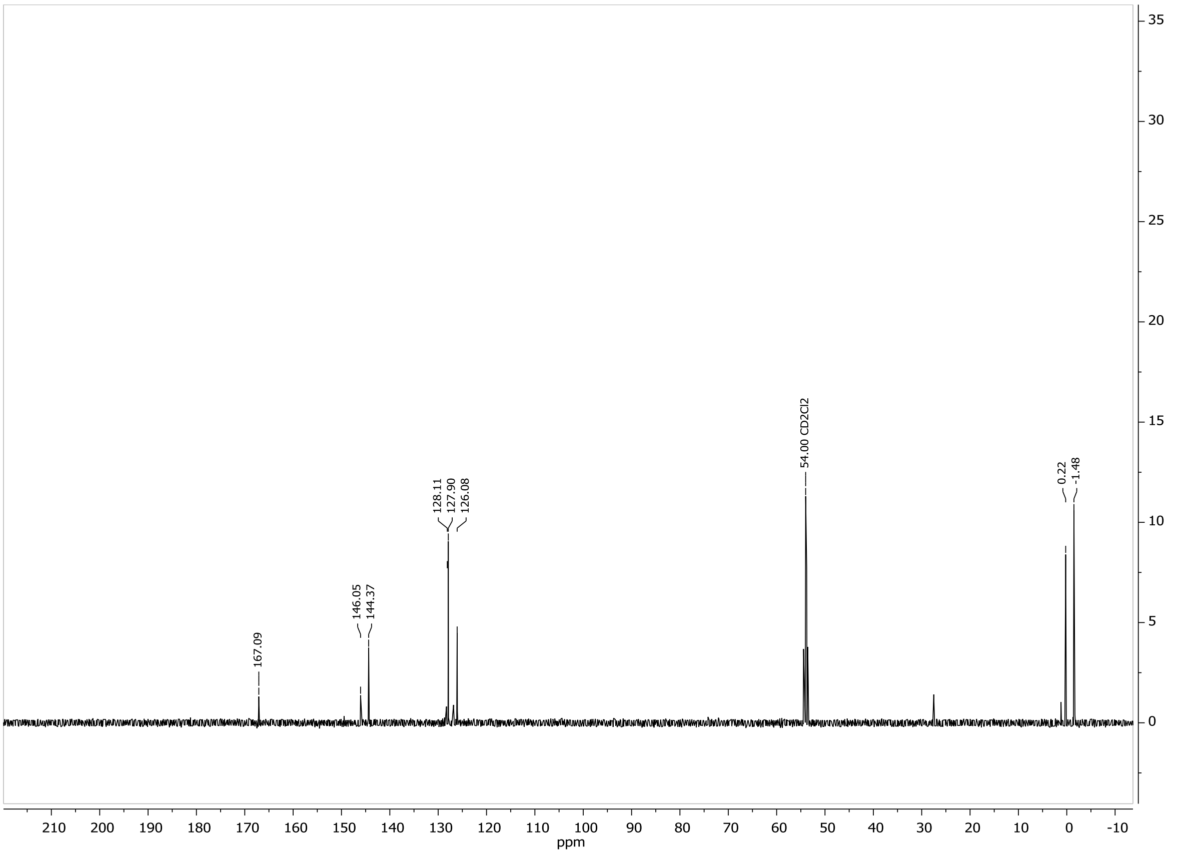

^{13}C NMR (126 MHz, CD_2Cl_2)

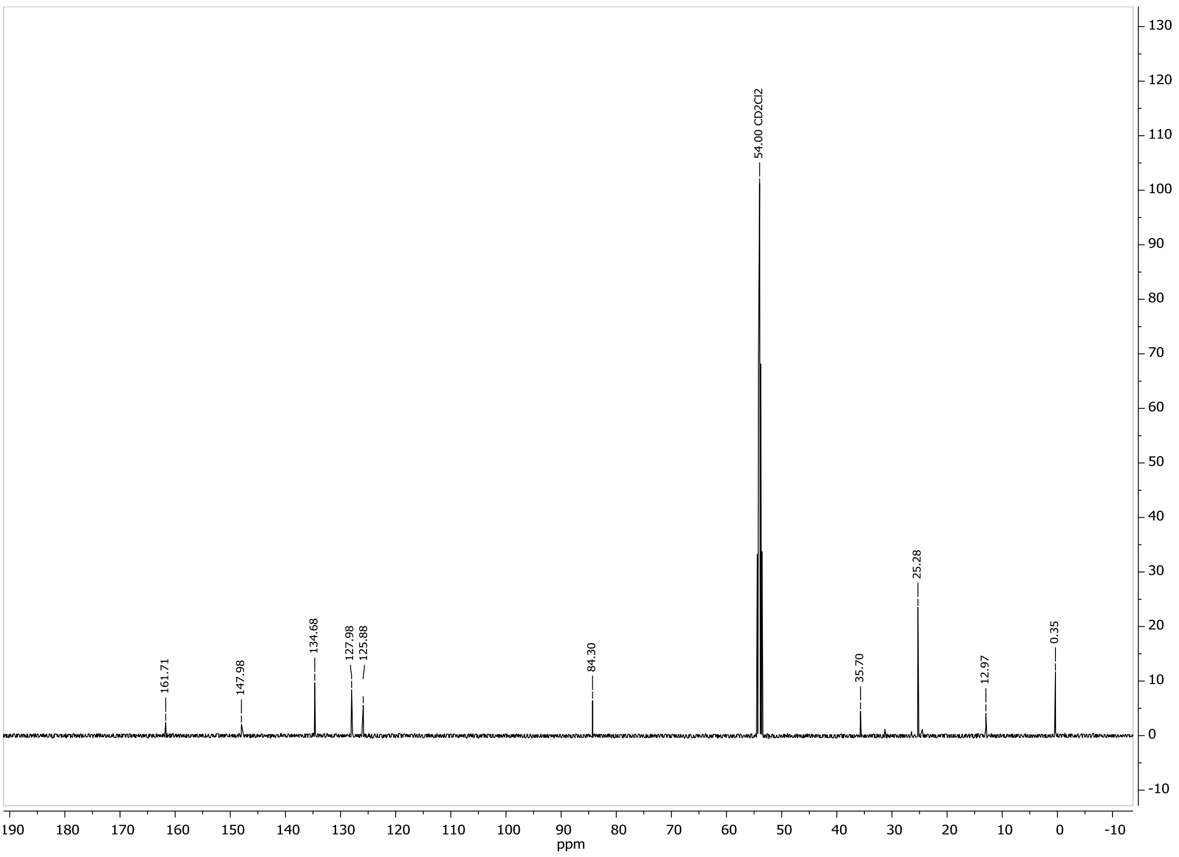

^{1}H NMR (500 MHz, CD_2Cl_2)

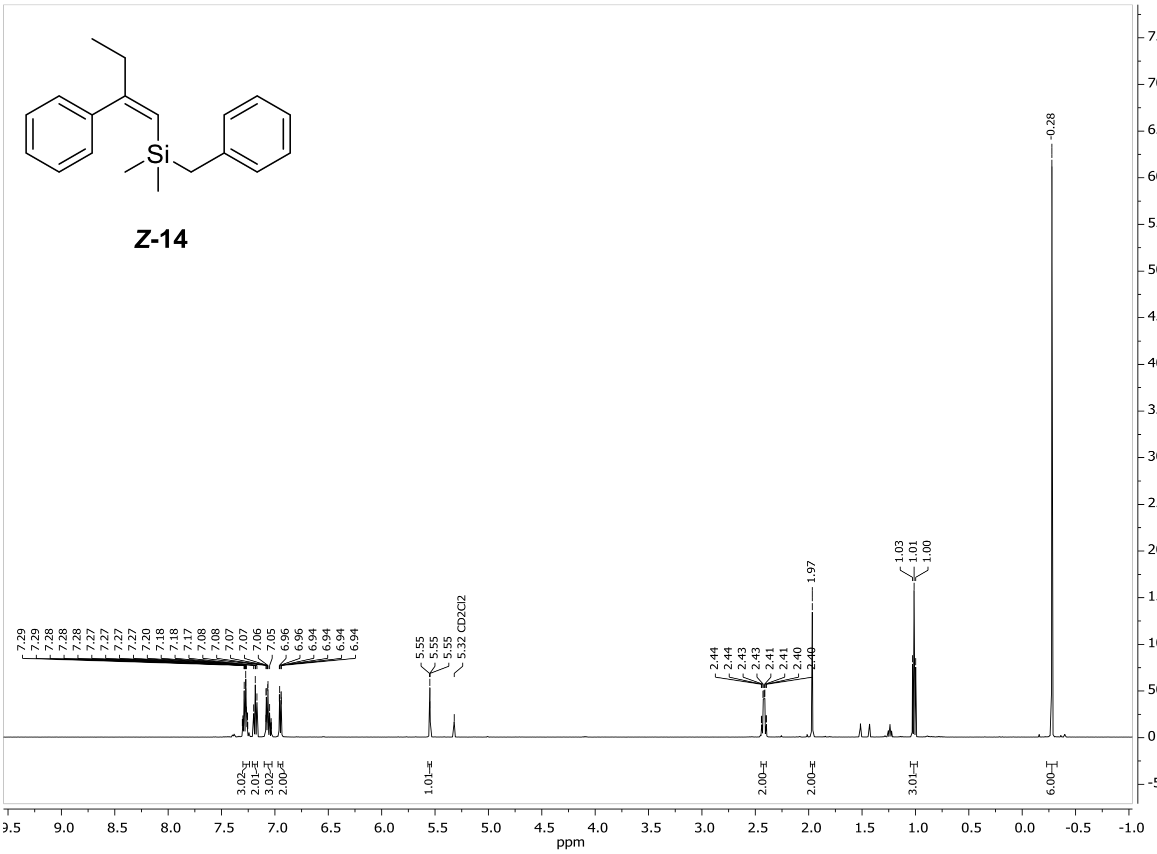

^{13}C NMR (126 MHz, CD_2Cl_2)

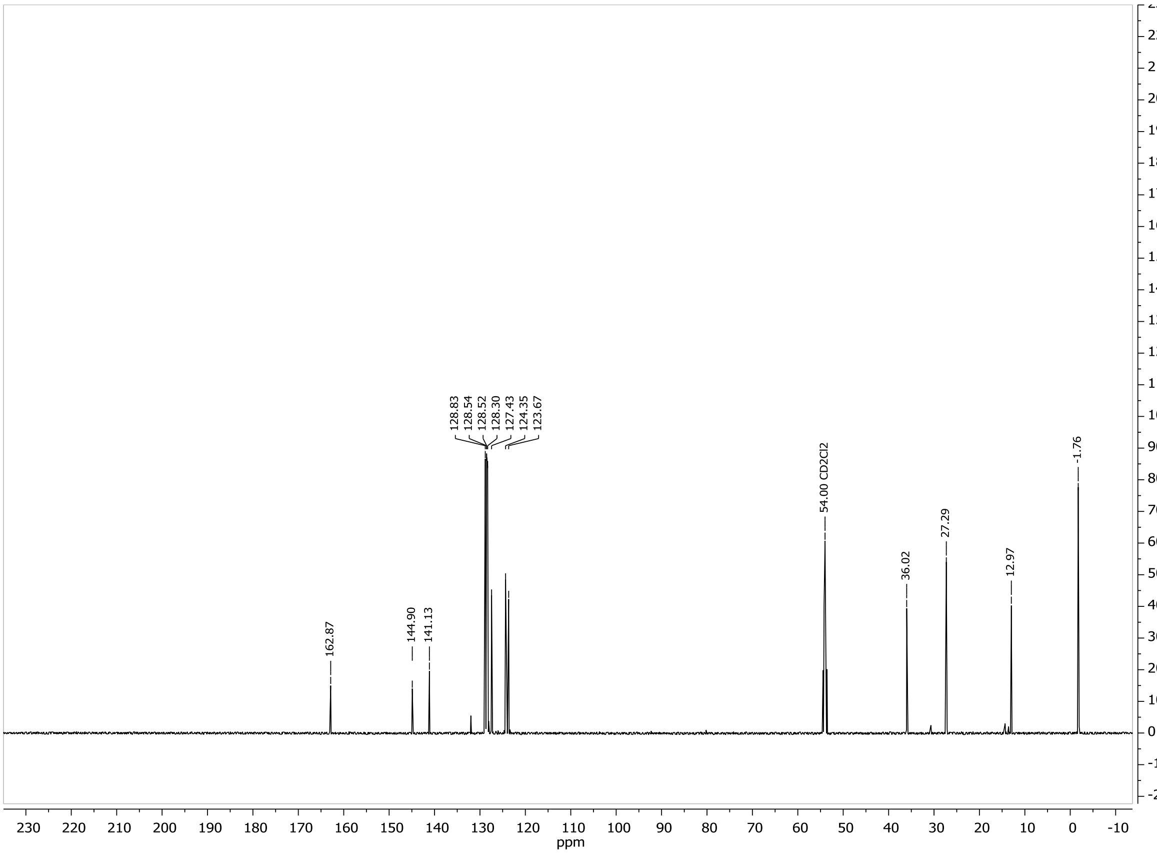

^1H NMR (500 MHz, CD_2Cl_2)

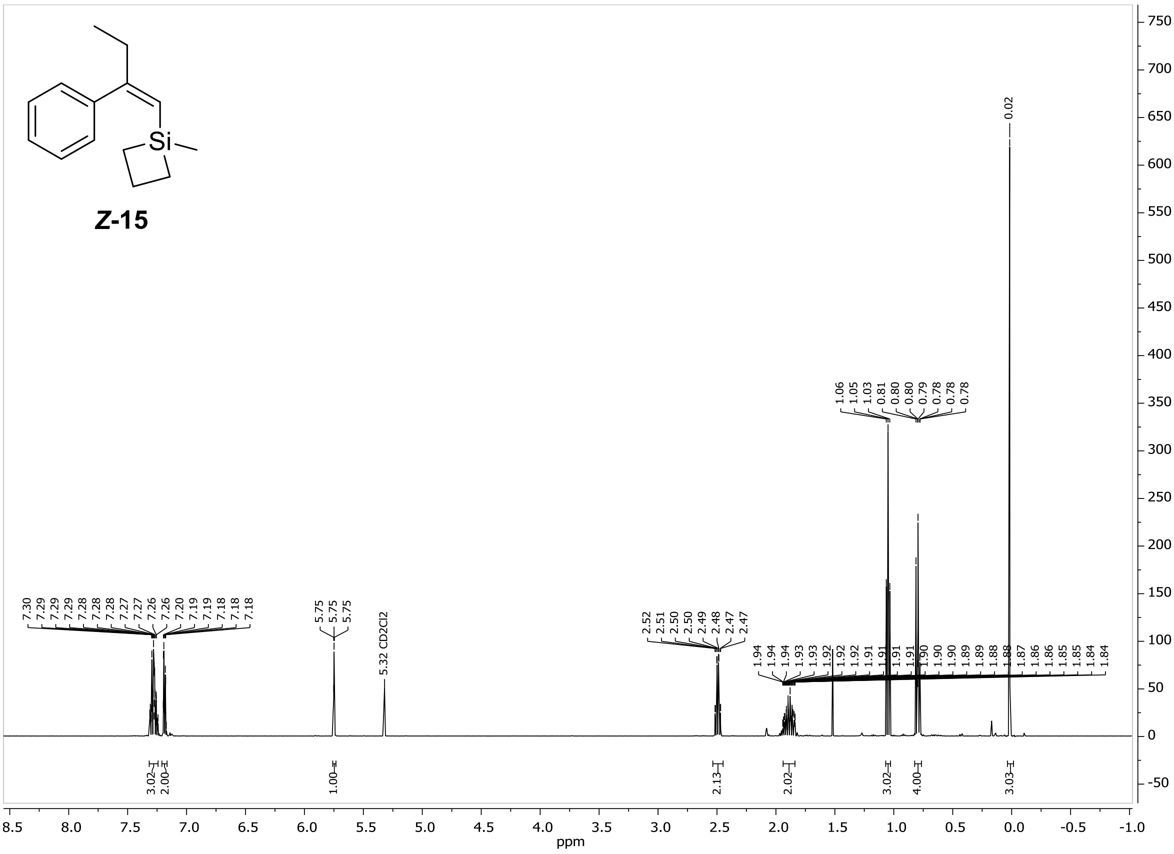

^{13}C NMR (126 MHz, CD_2Cl_2)

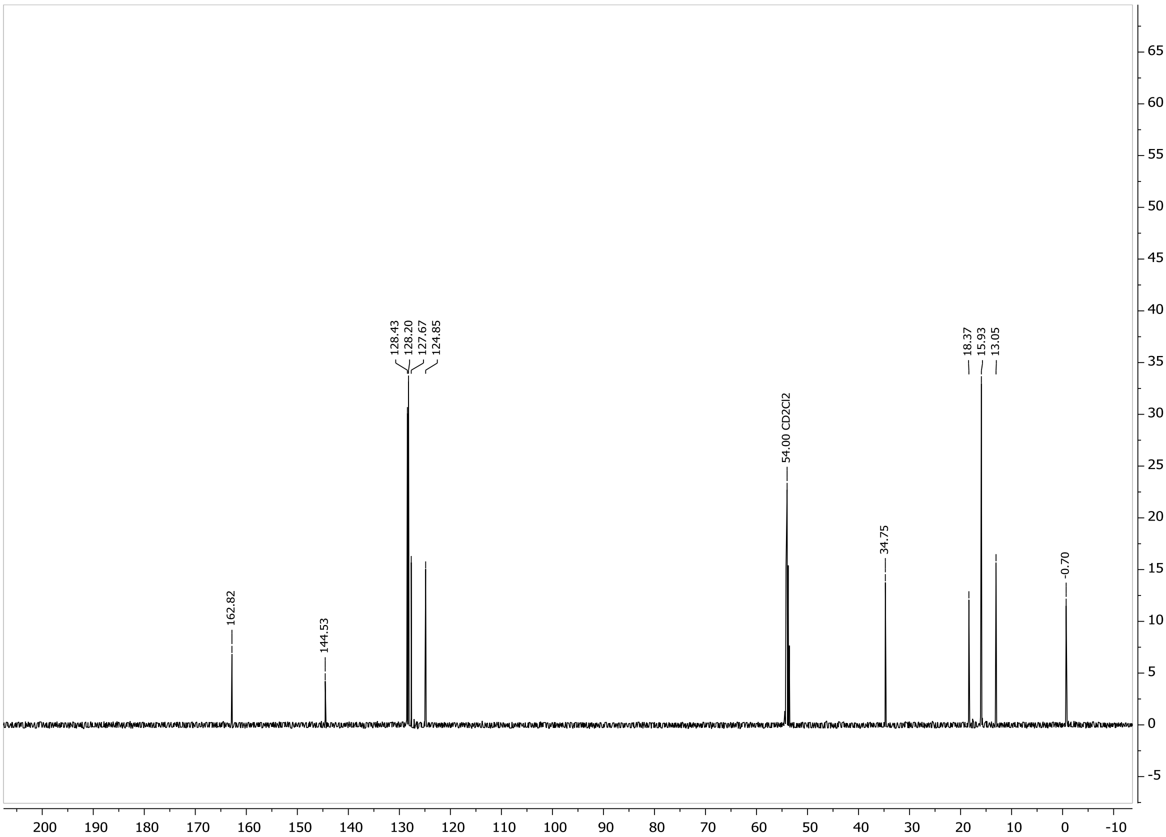

^1H NMR (500 MHz, CD_2Cl_2)

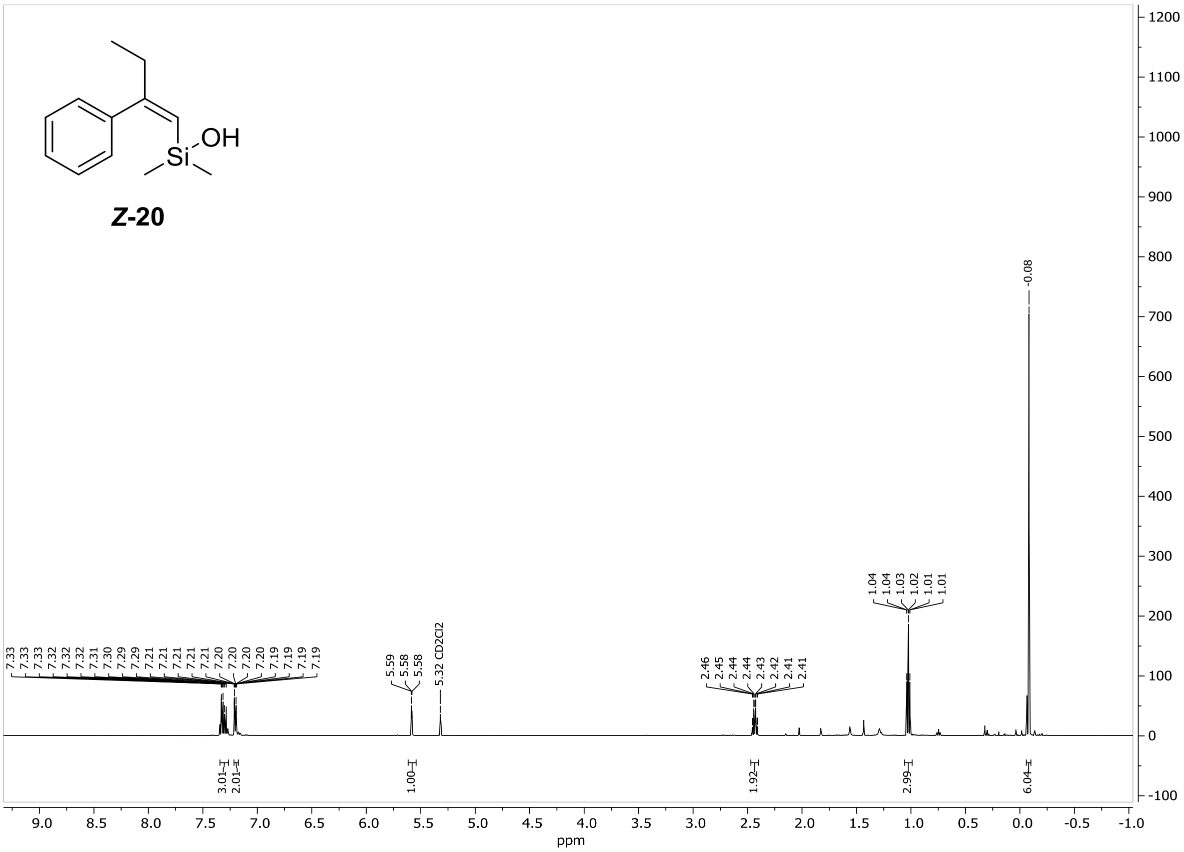

^{13}C NMR (126 MHz, CD_2Cl_2)

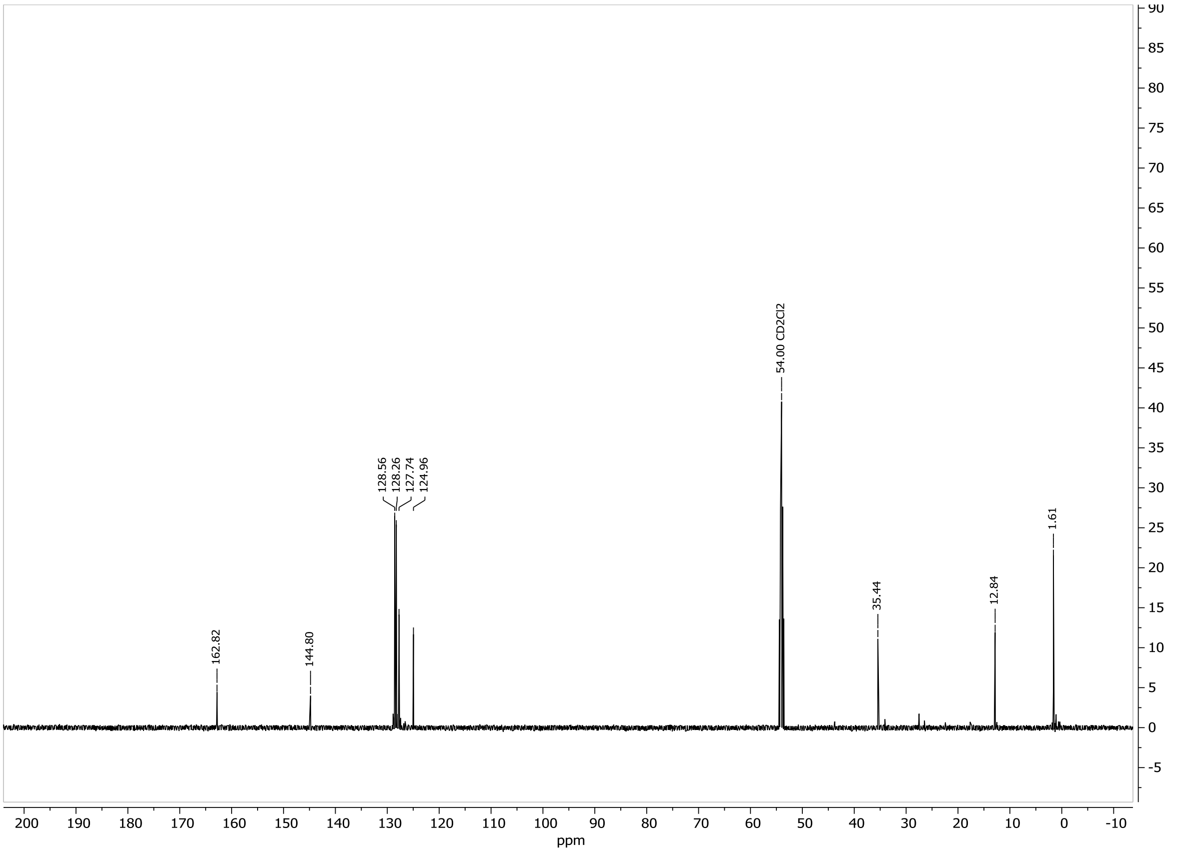

^{1}H NMR (300 MHz, $CDCl_3$)

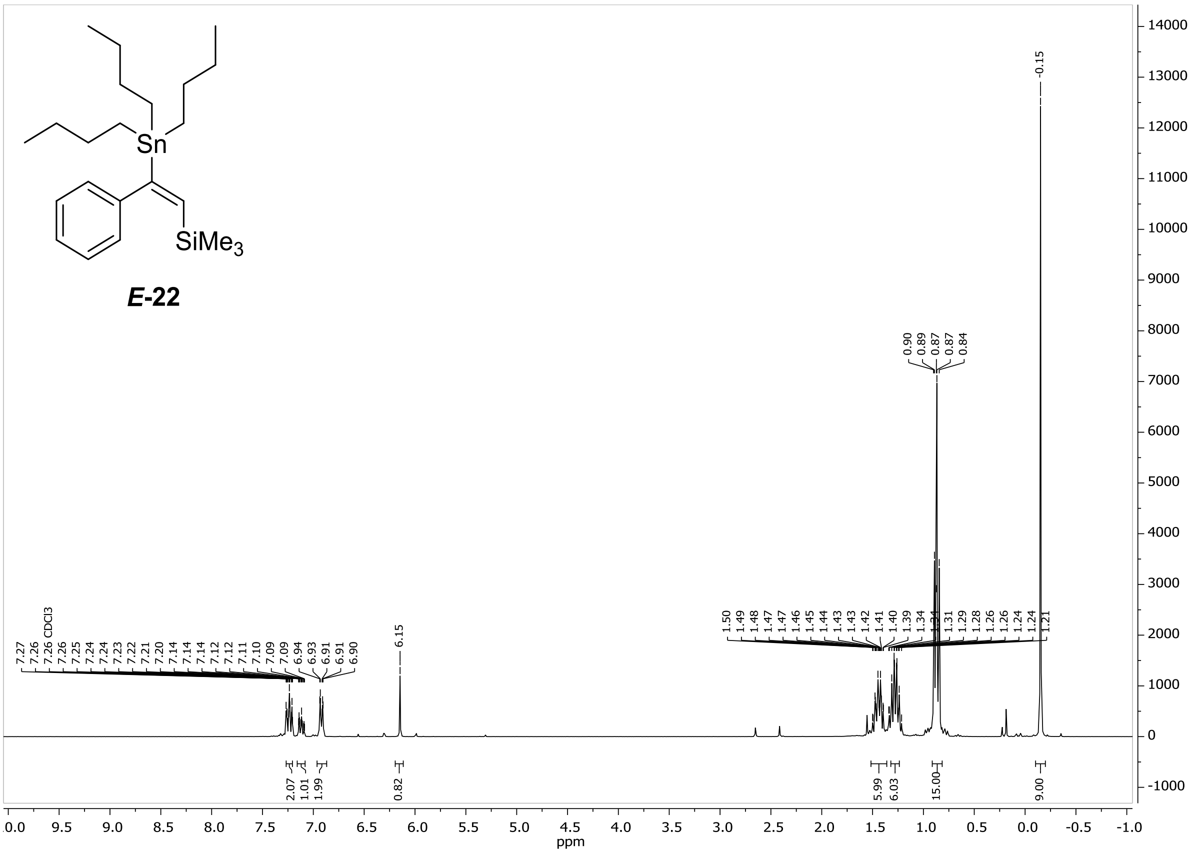

^{1}H NMR (300 MHz, $CDCl_3$)

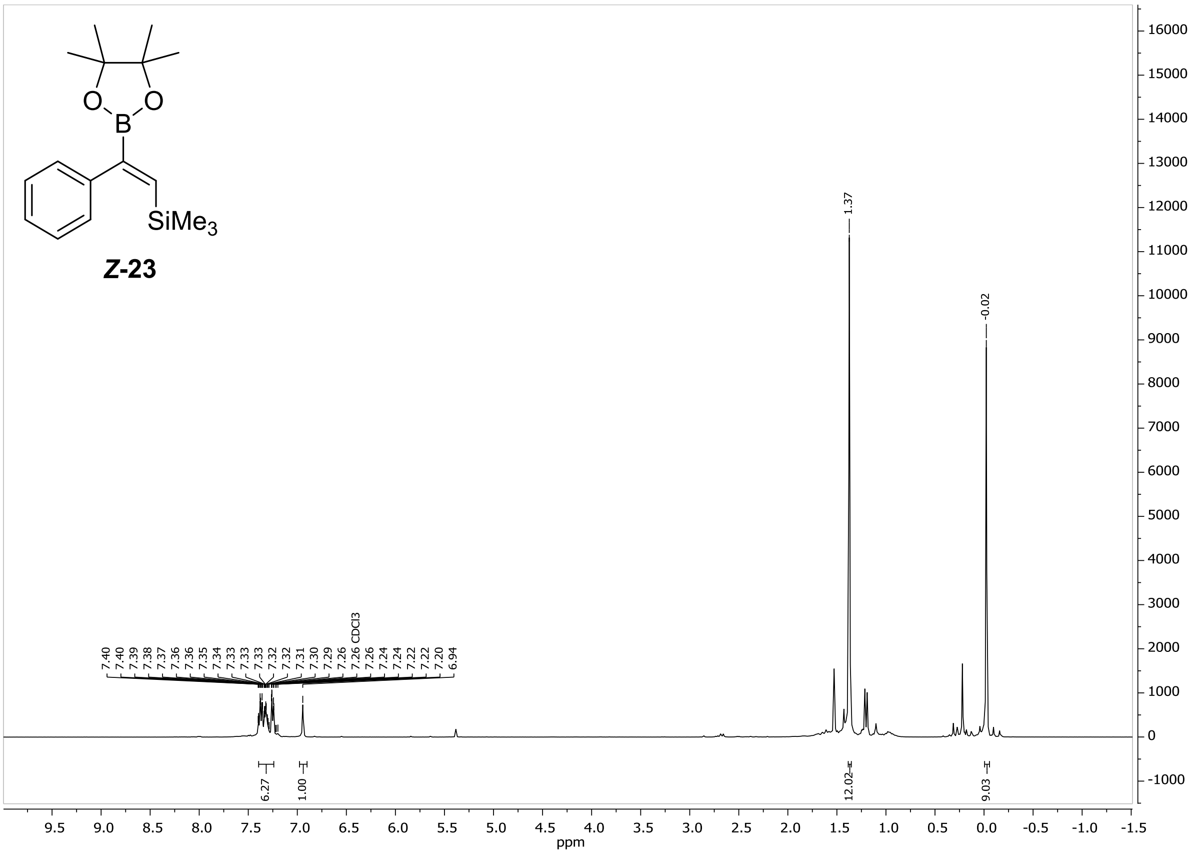

^{1}H NMR (600 MHz, $CDCl_3$)

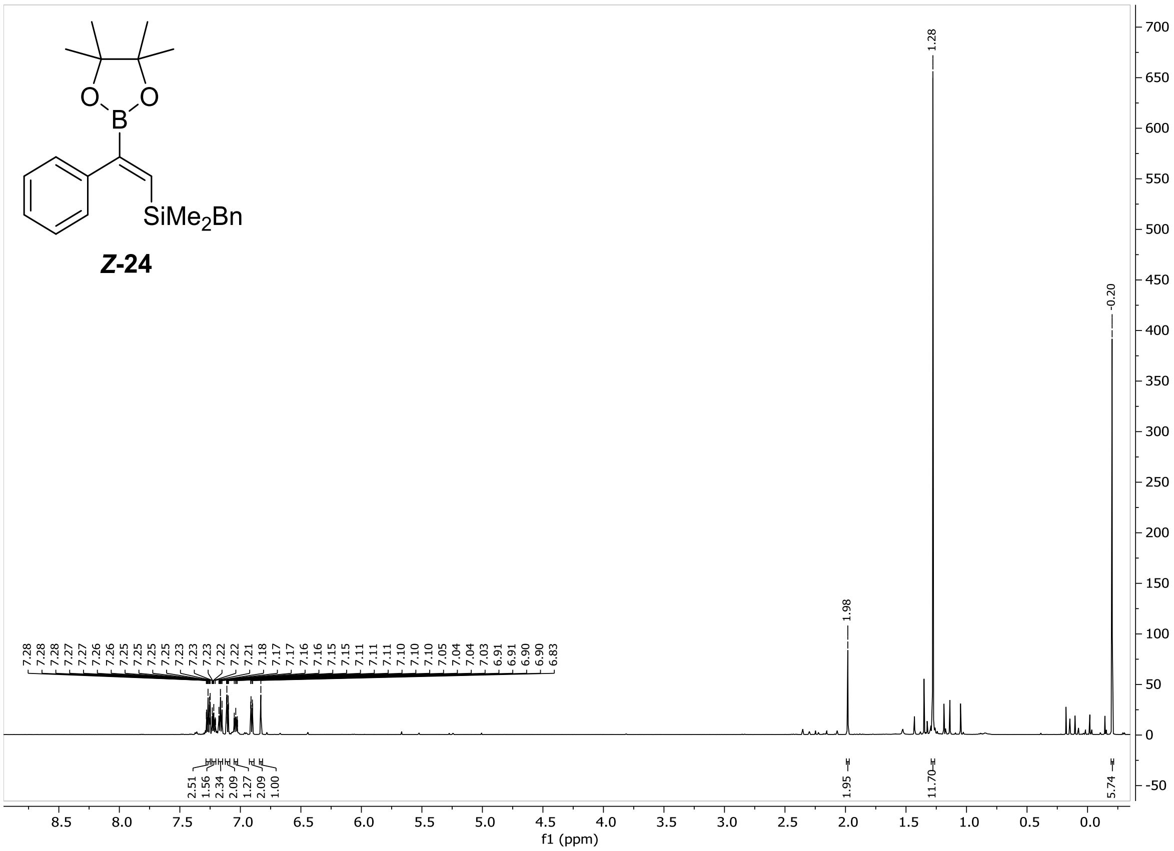

^{13}C NMR (151 MHz, $CDCl_3$)

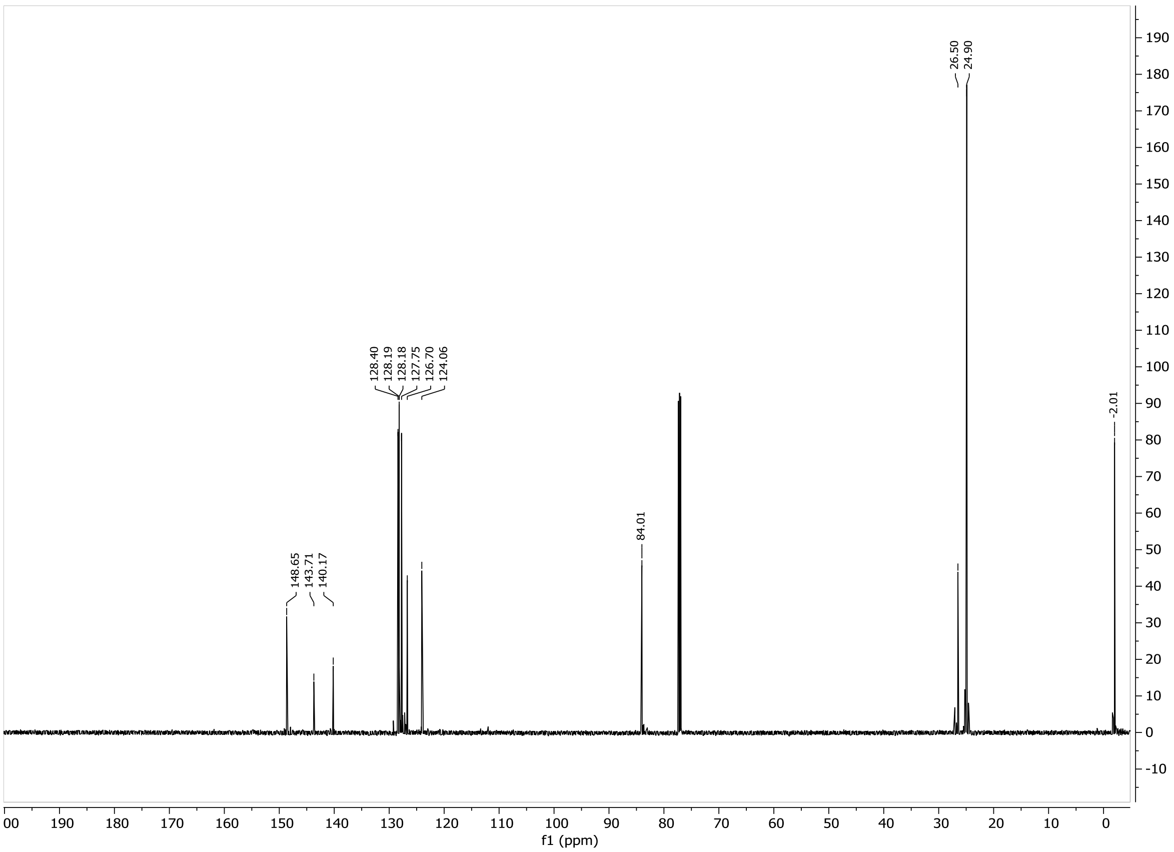

^{1}H NMR (600 MHz, $CDCl_3$)

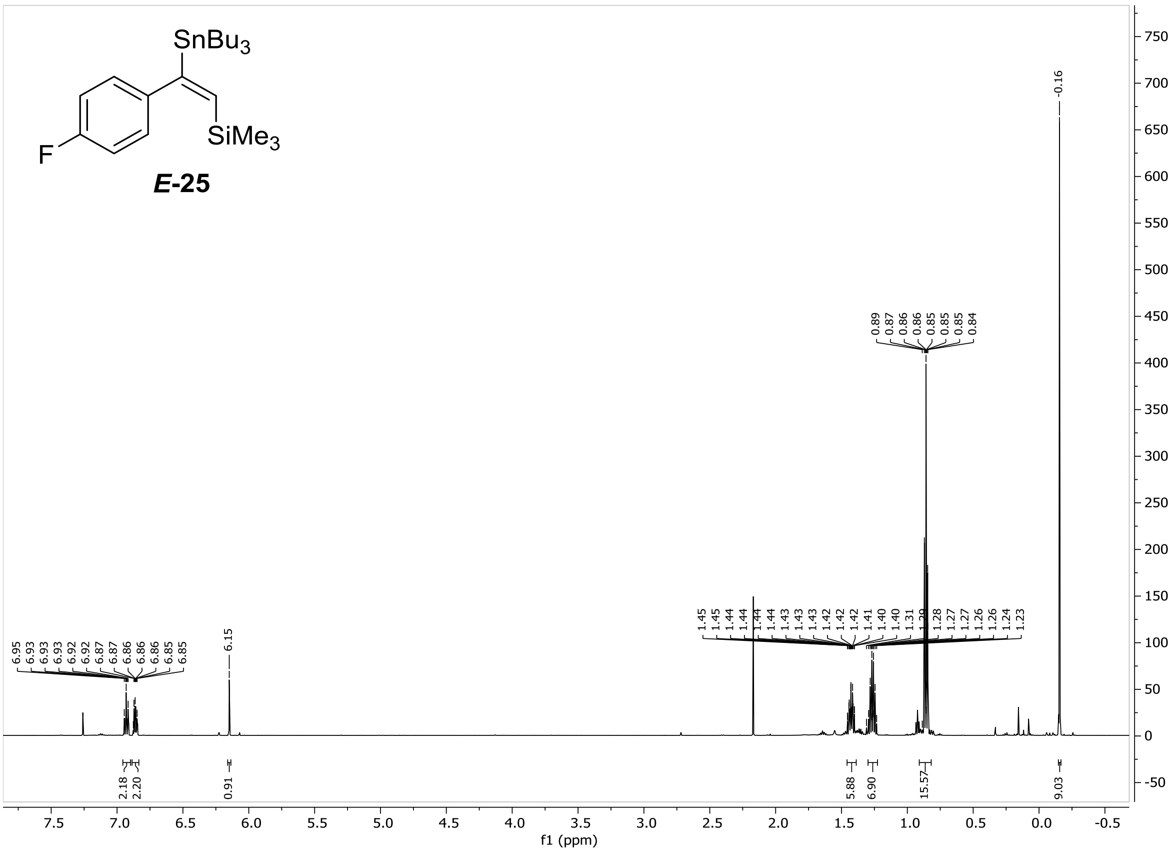

^{13}C NMR (151 MHz, $CDCl_3$)

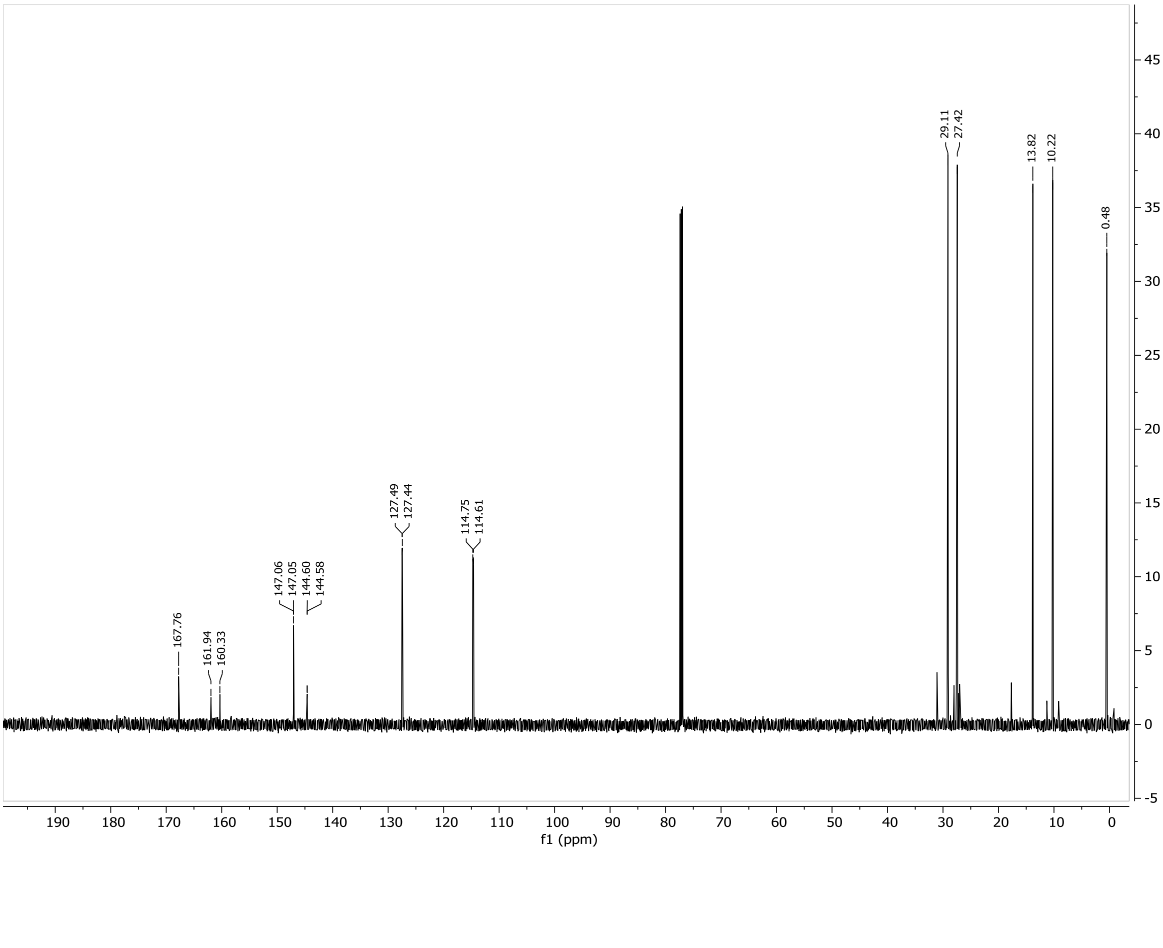

^{19}F NMR (564 MHz, $CDCl_3$)

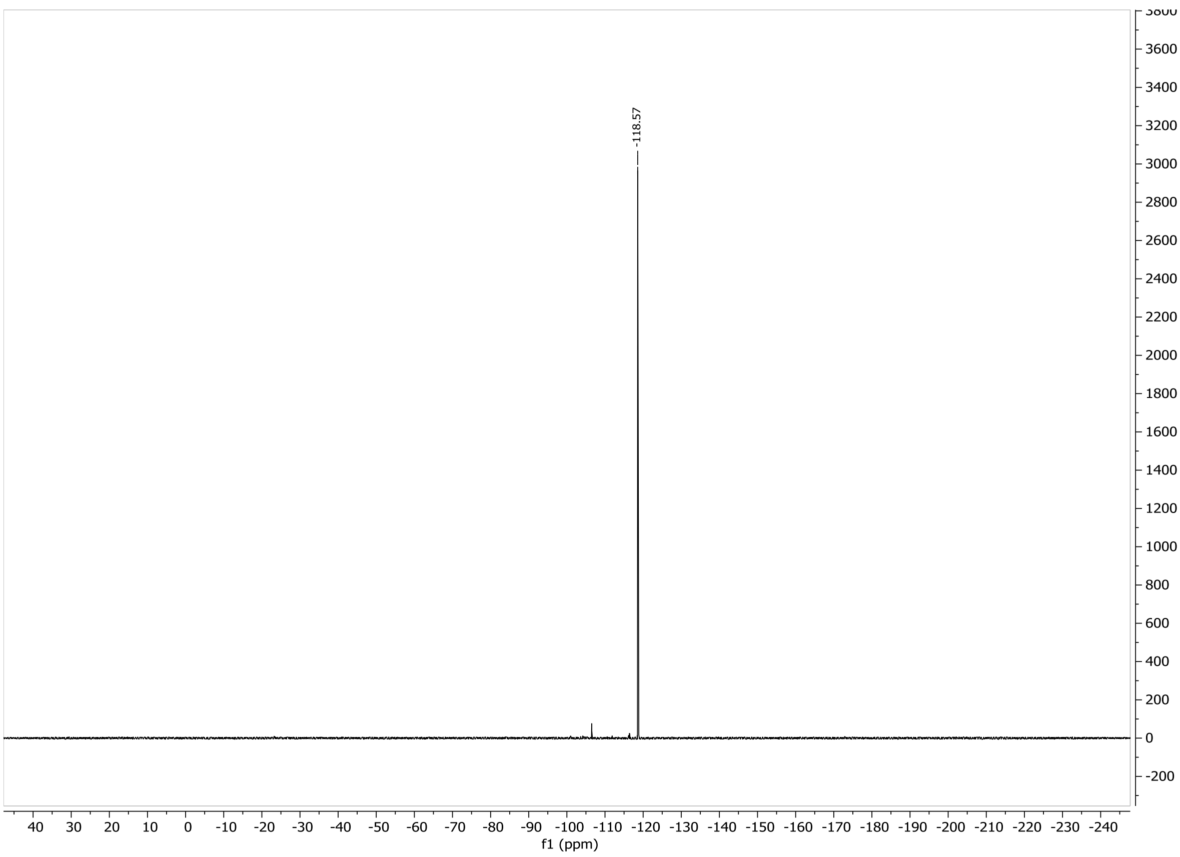

^{1}H NMR (600 MHz, $CDCl_3$)

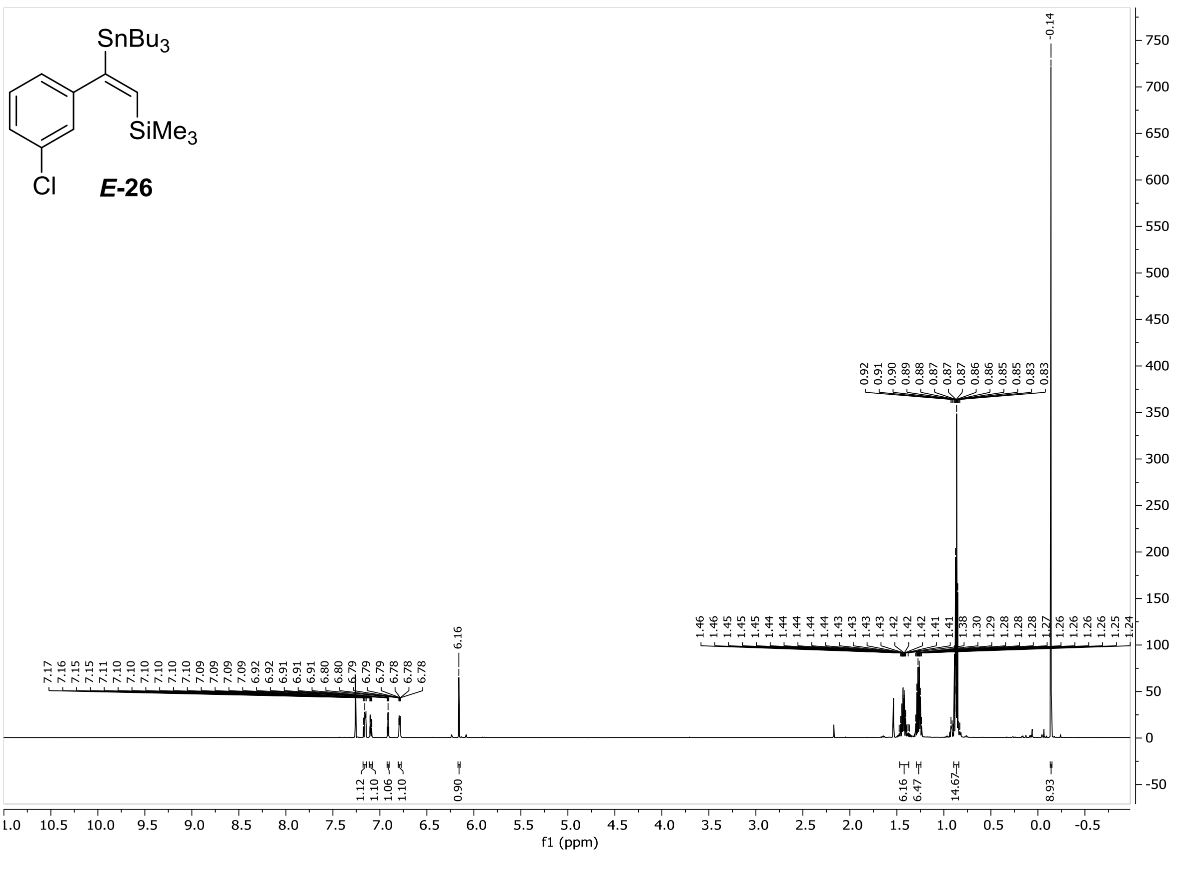

^{13}C NMR (151 MHz, $CDCl_3$)

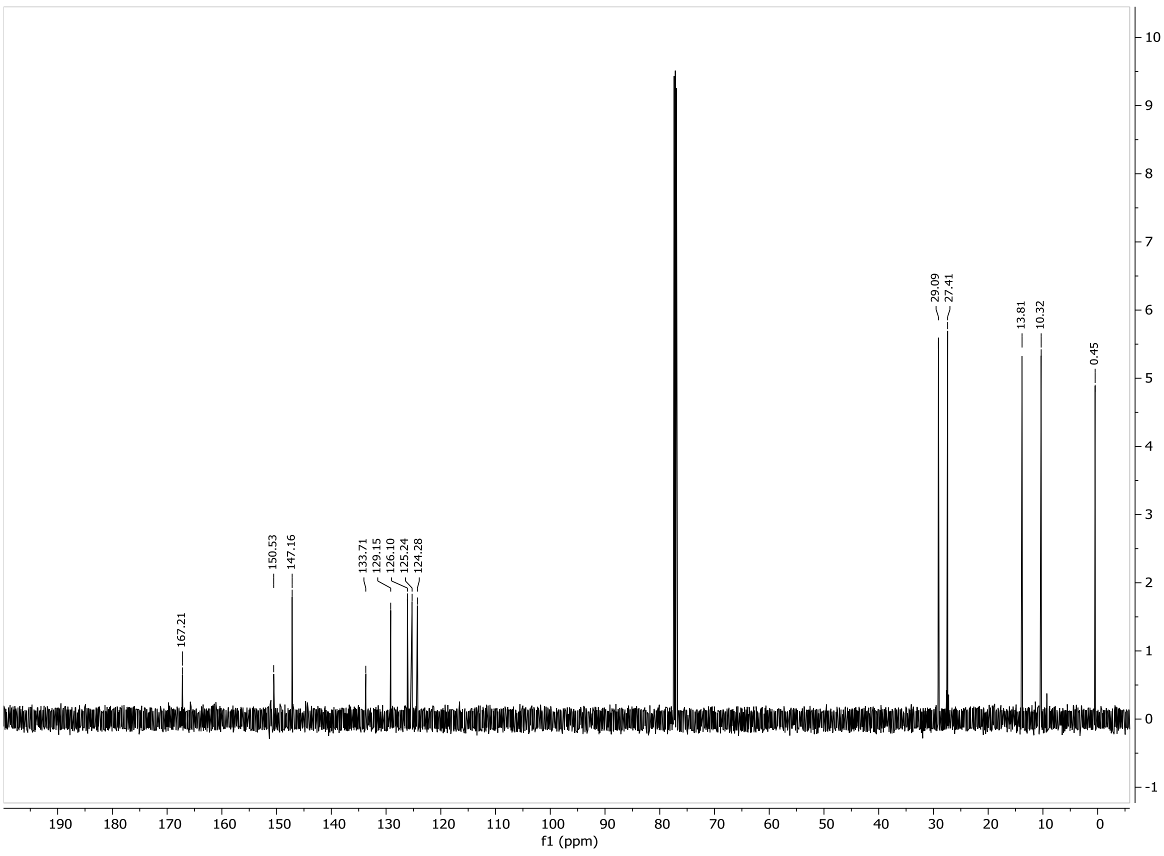

^{1}H NMR (600 MHz, $CDCl_3$)

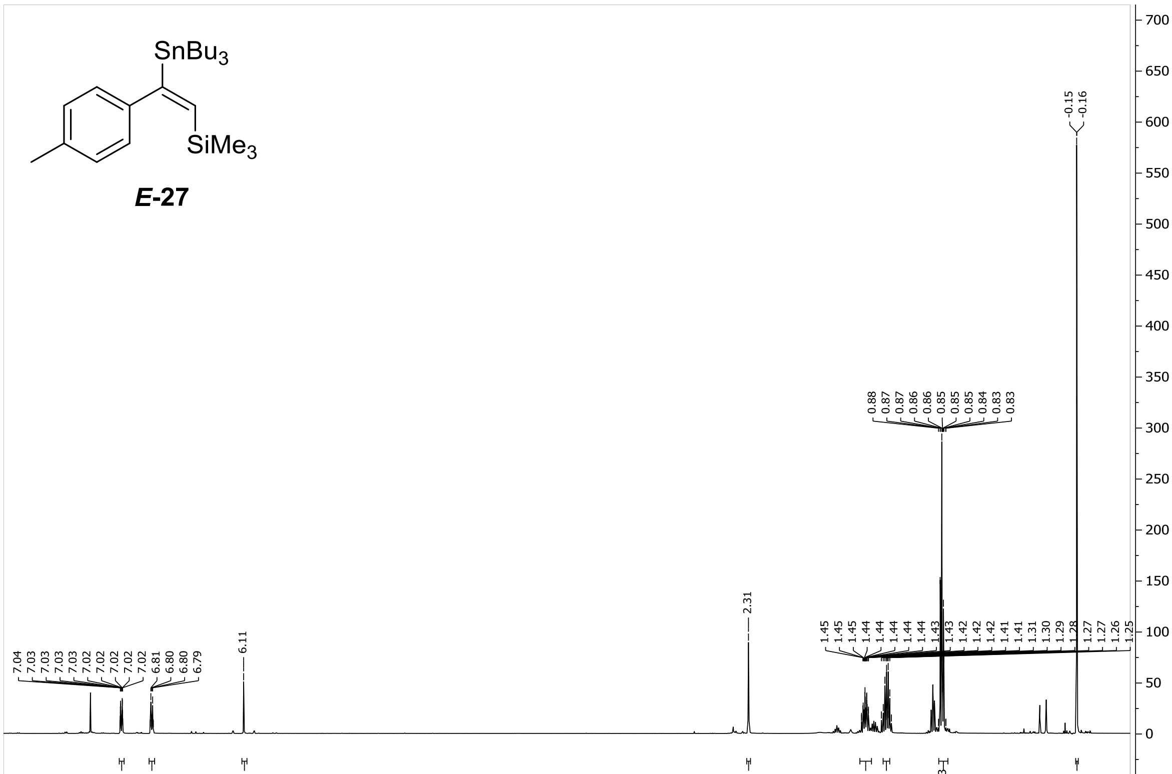

^{13}C NMR (151 MHz, $CDCl_3$)

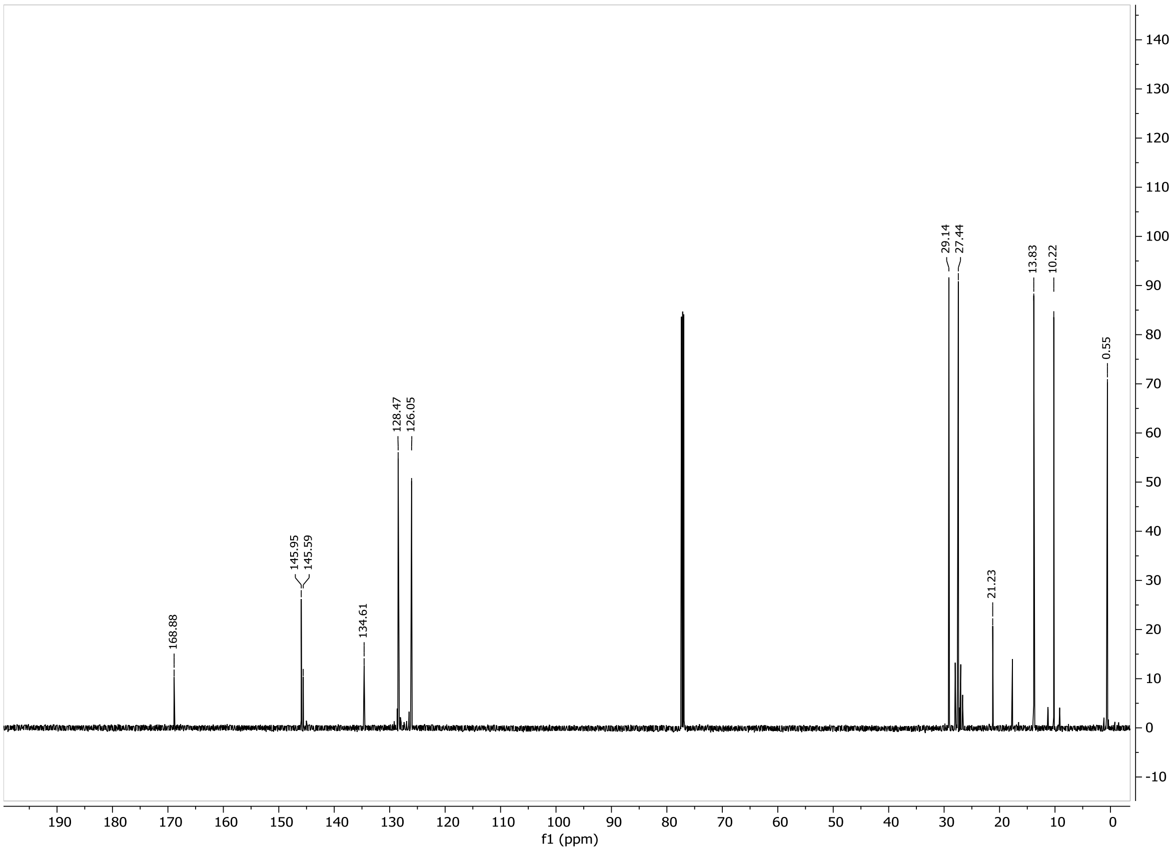

^{1}H NMR (400 MHz, $CDCl_3$)

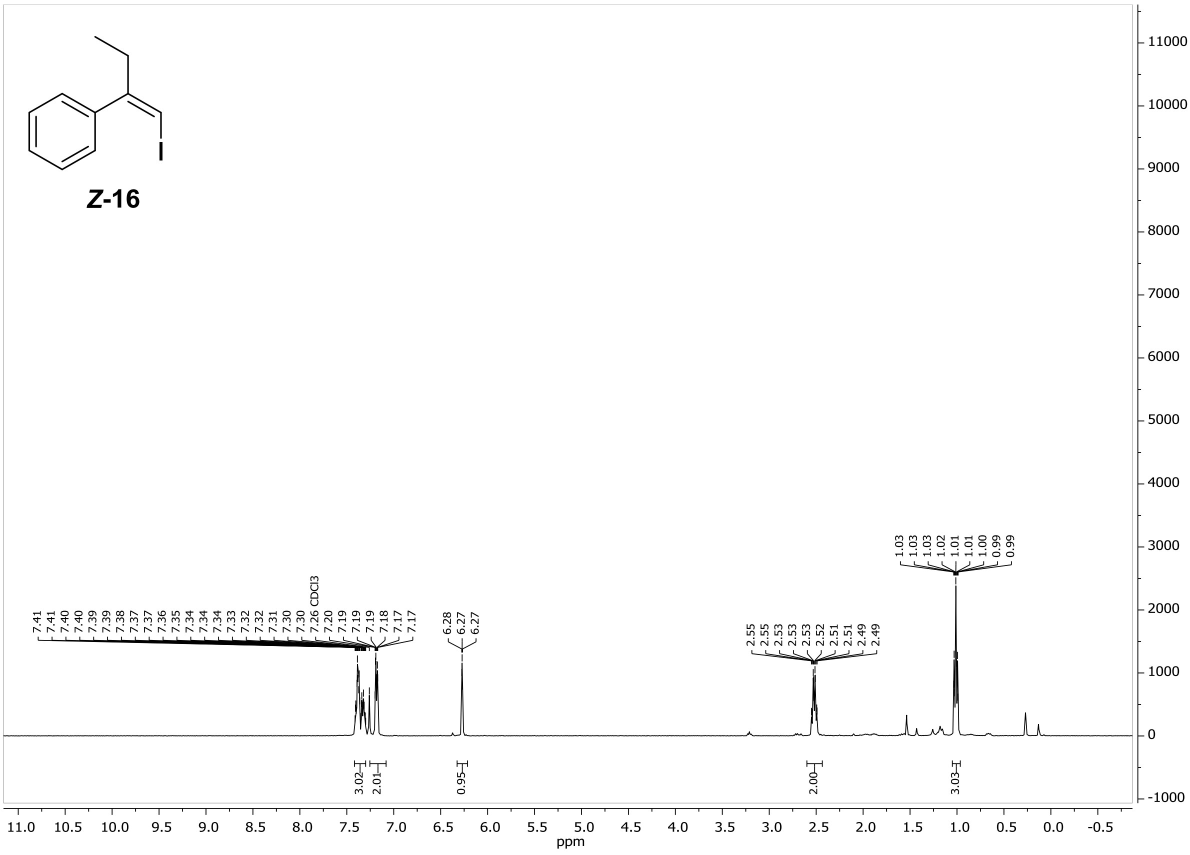

^{1}H (600 MHz, CD_2Cl_2)

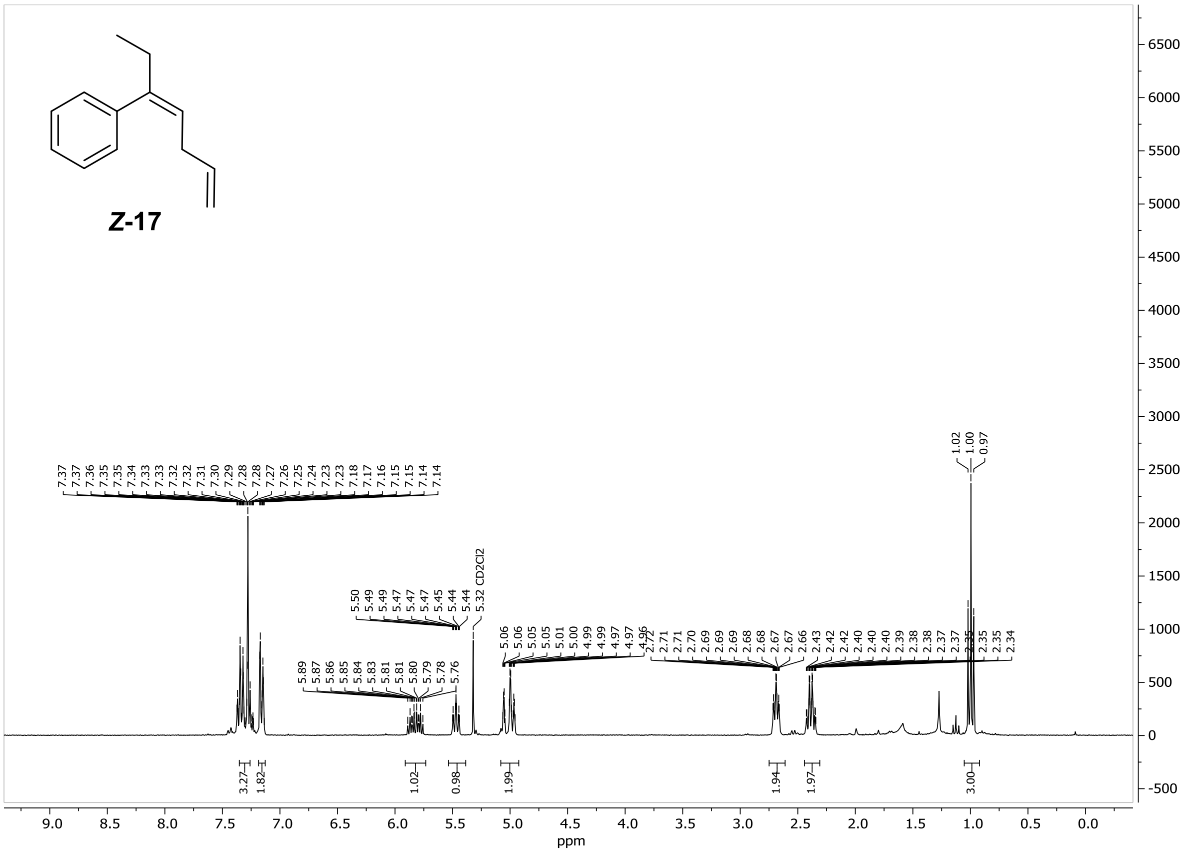

^{13}C (600 MHz, CD_2Cl_2)

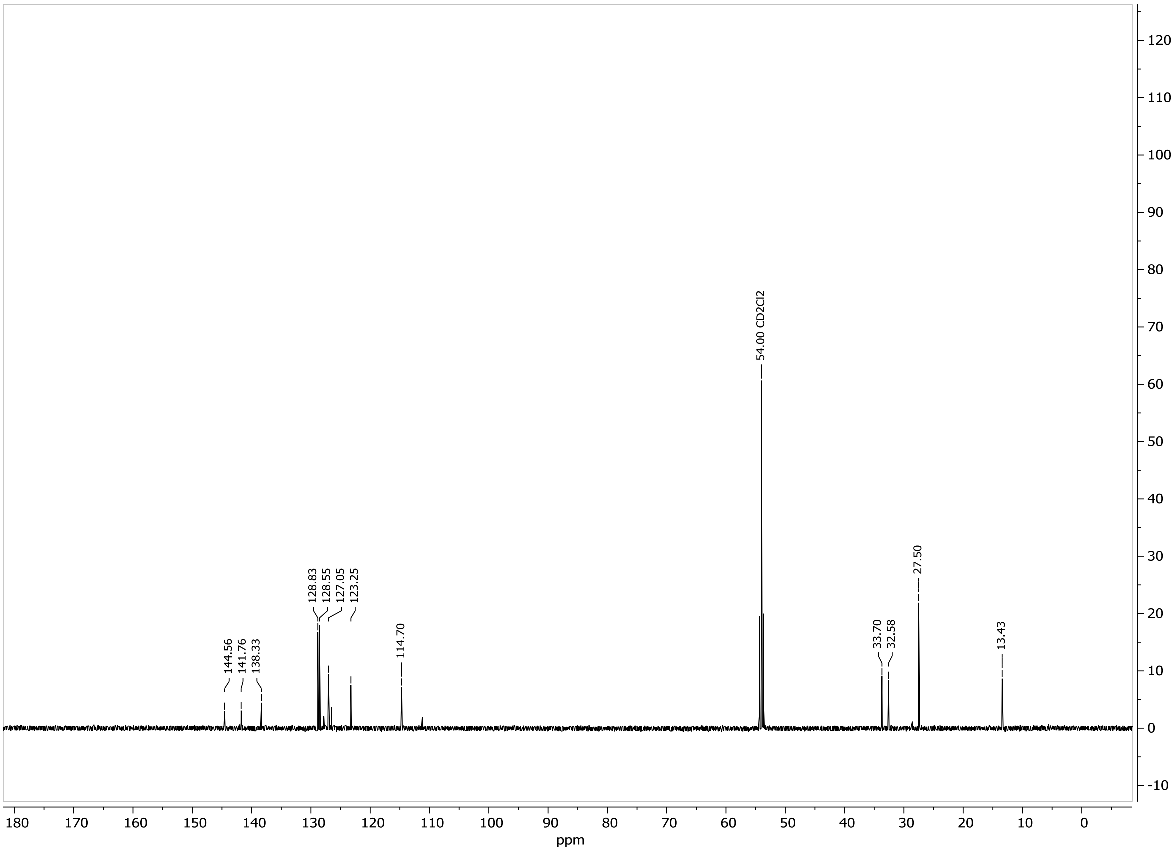

^{1}H NMR (400 MHz, $CDCl_3$)

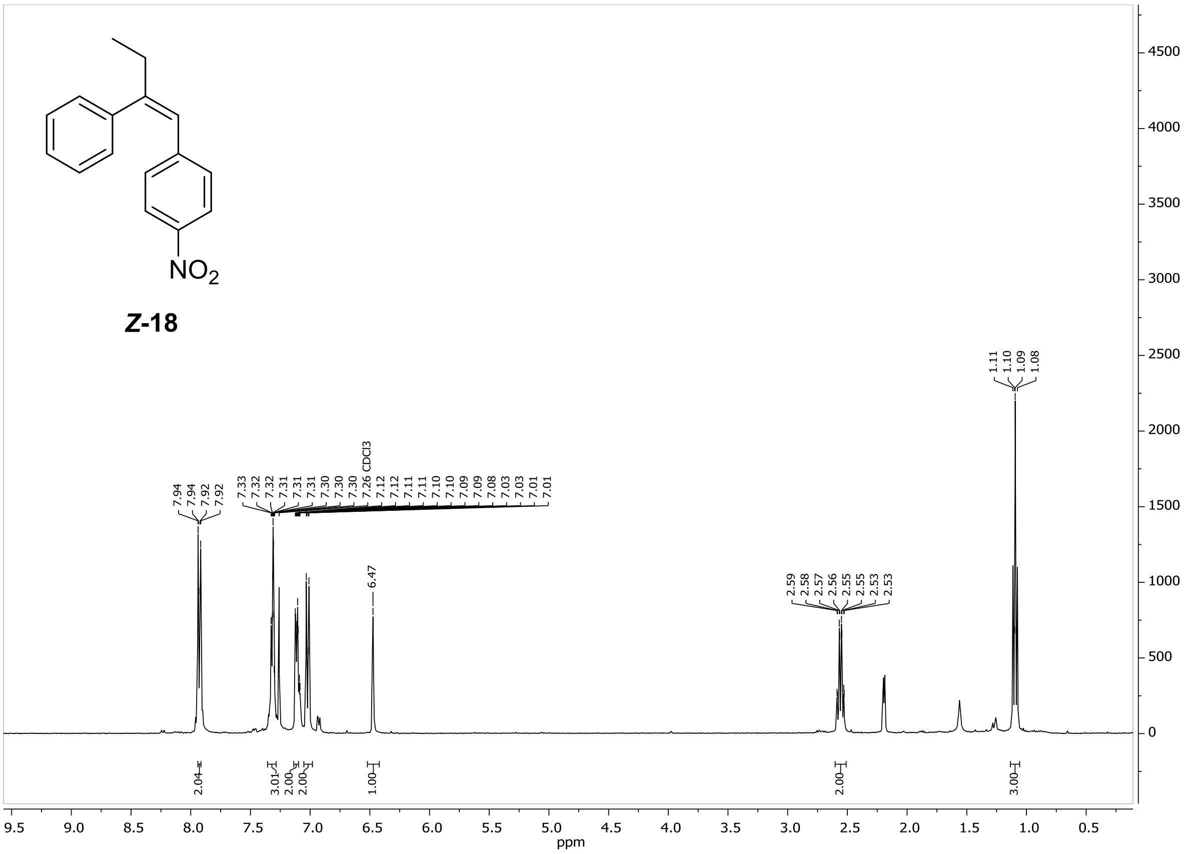

^{1}H NMR (400 MHz, $CDCl_3$)

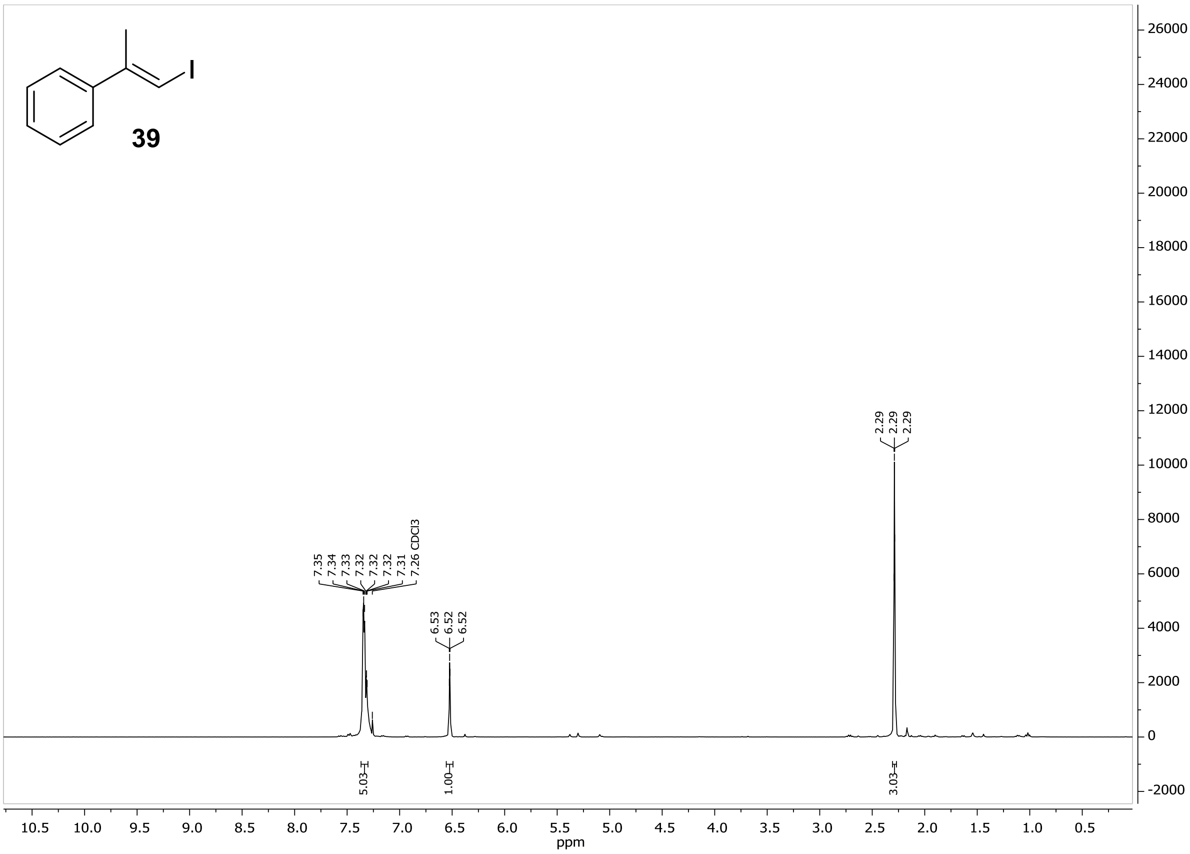

^{1}H NMR (600 MHz, $CDCl_3$)

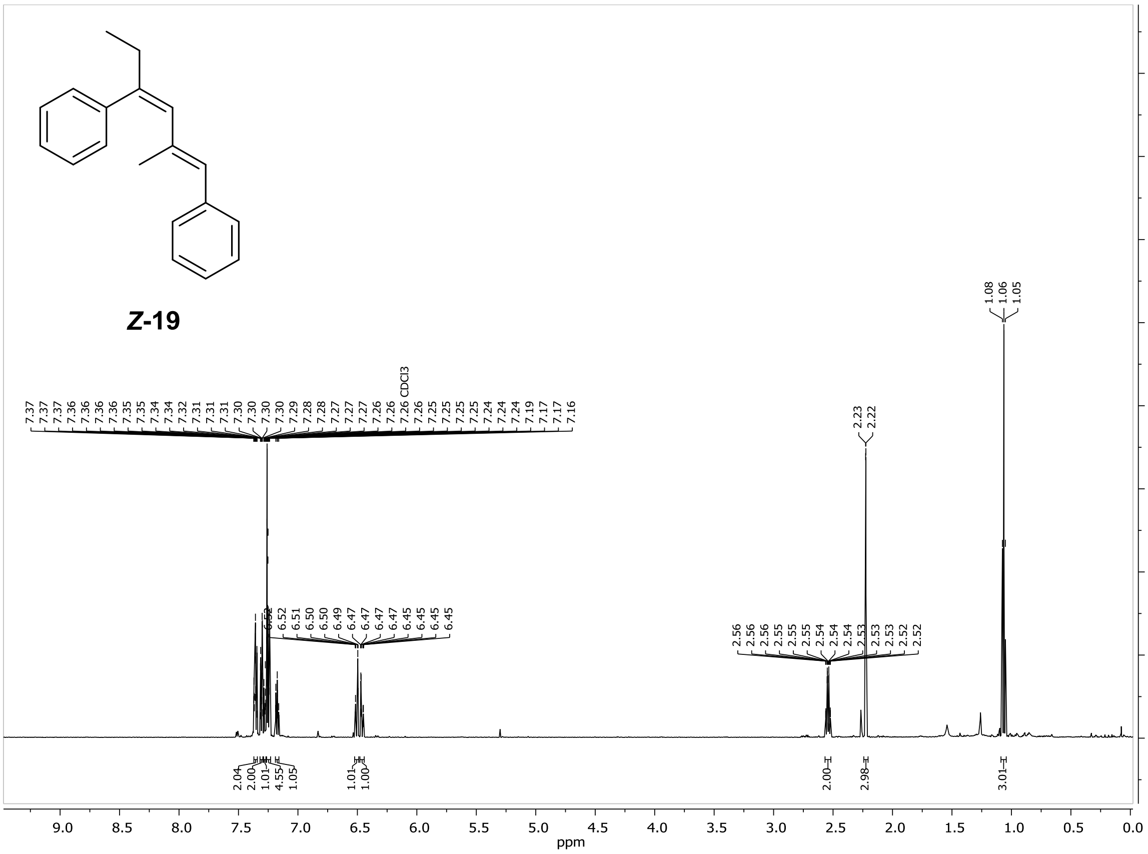

^{13}C NMR (151 MHz, $CDCl_3$)

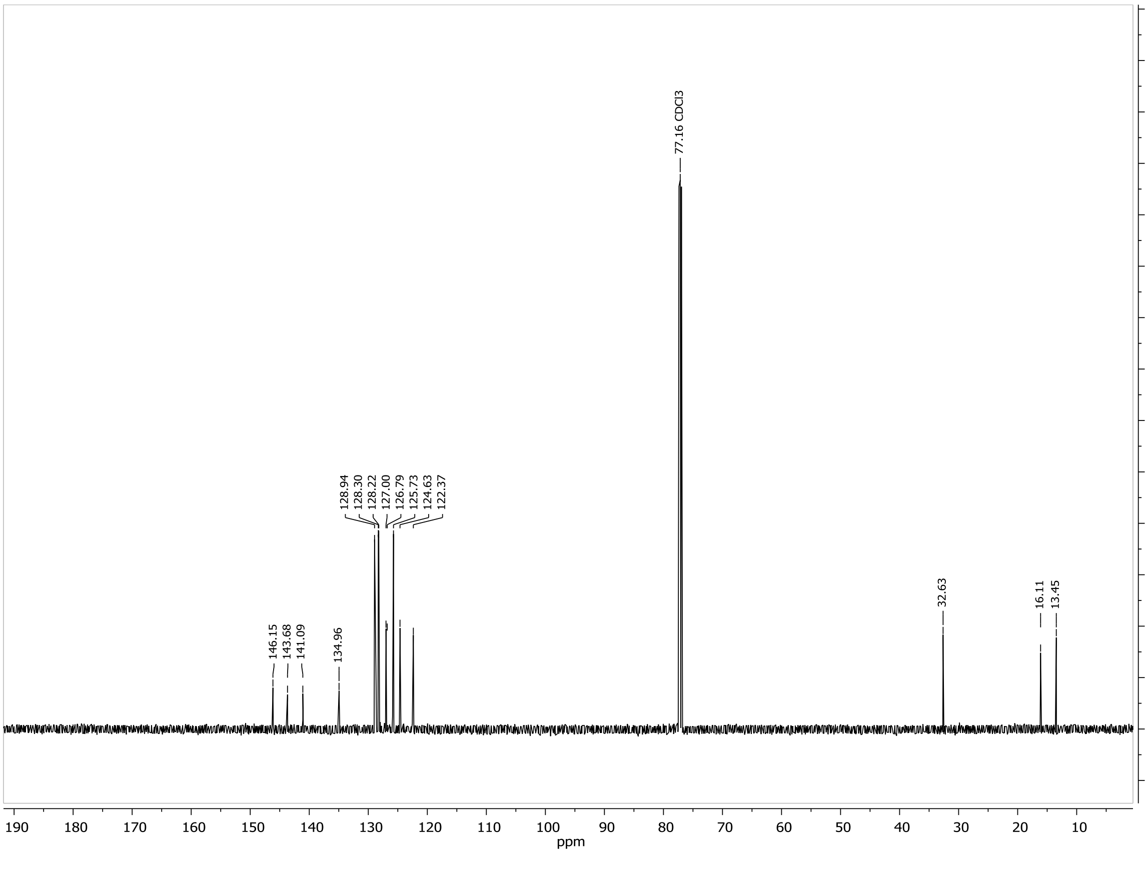

^{1}H NMR (300 MHz, $CDCl_3$)

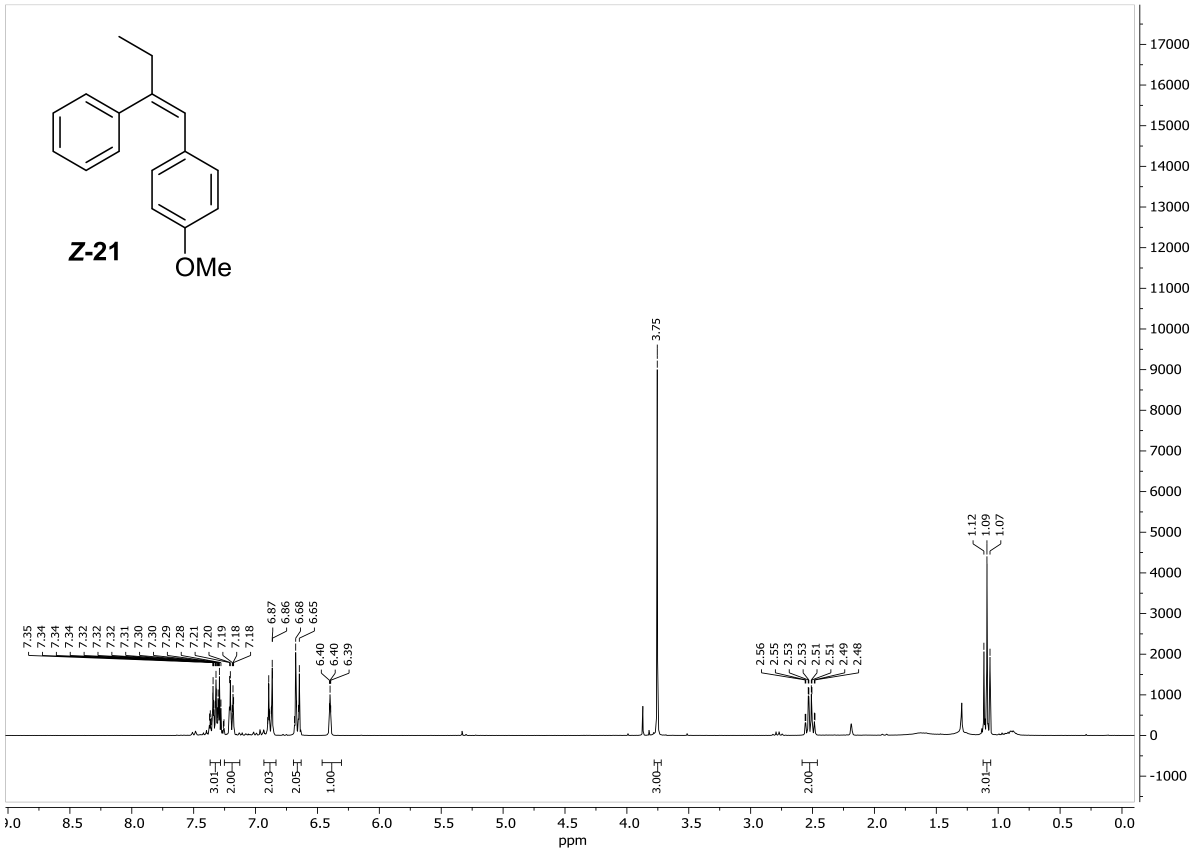

^{1}H NMR (600 MHz, $CDCl_3$)

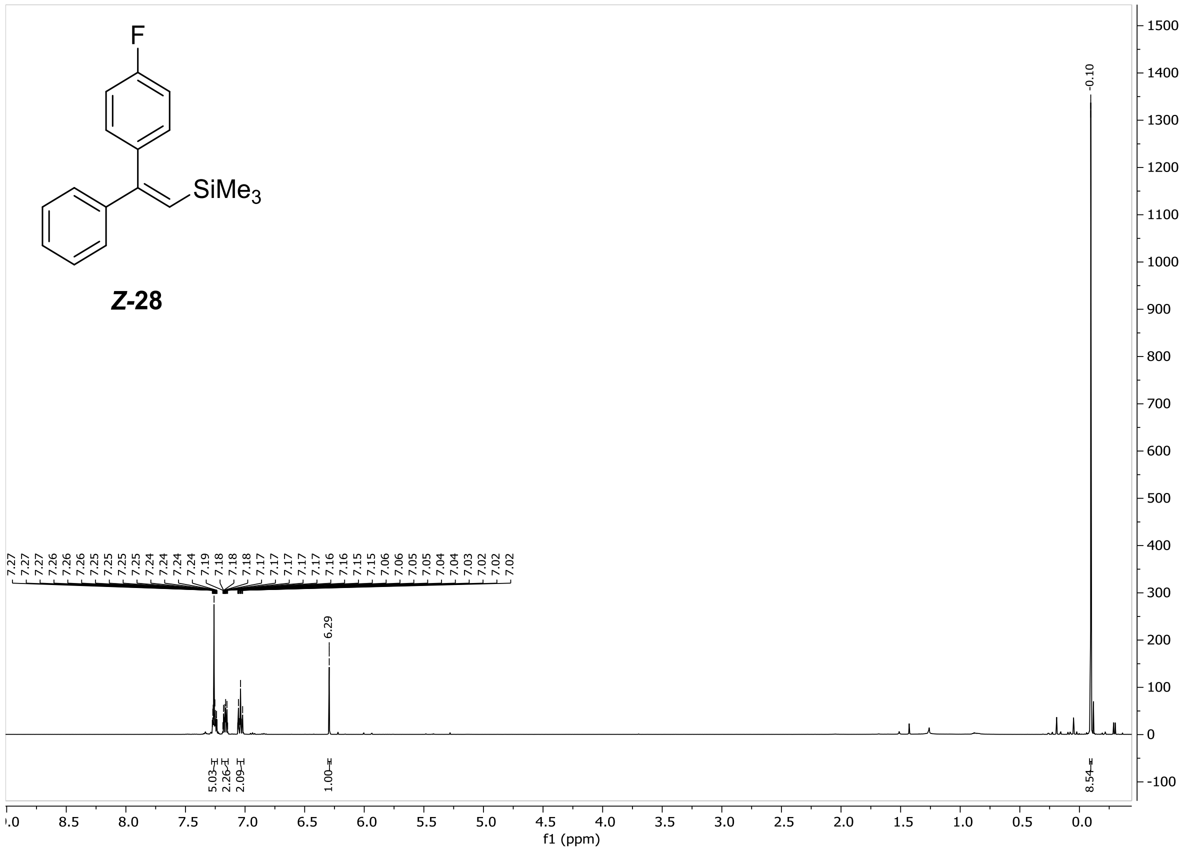

^{13}C NMR (151 MHz, $CDCl_3$)

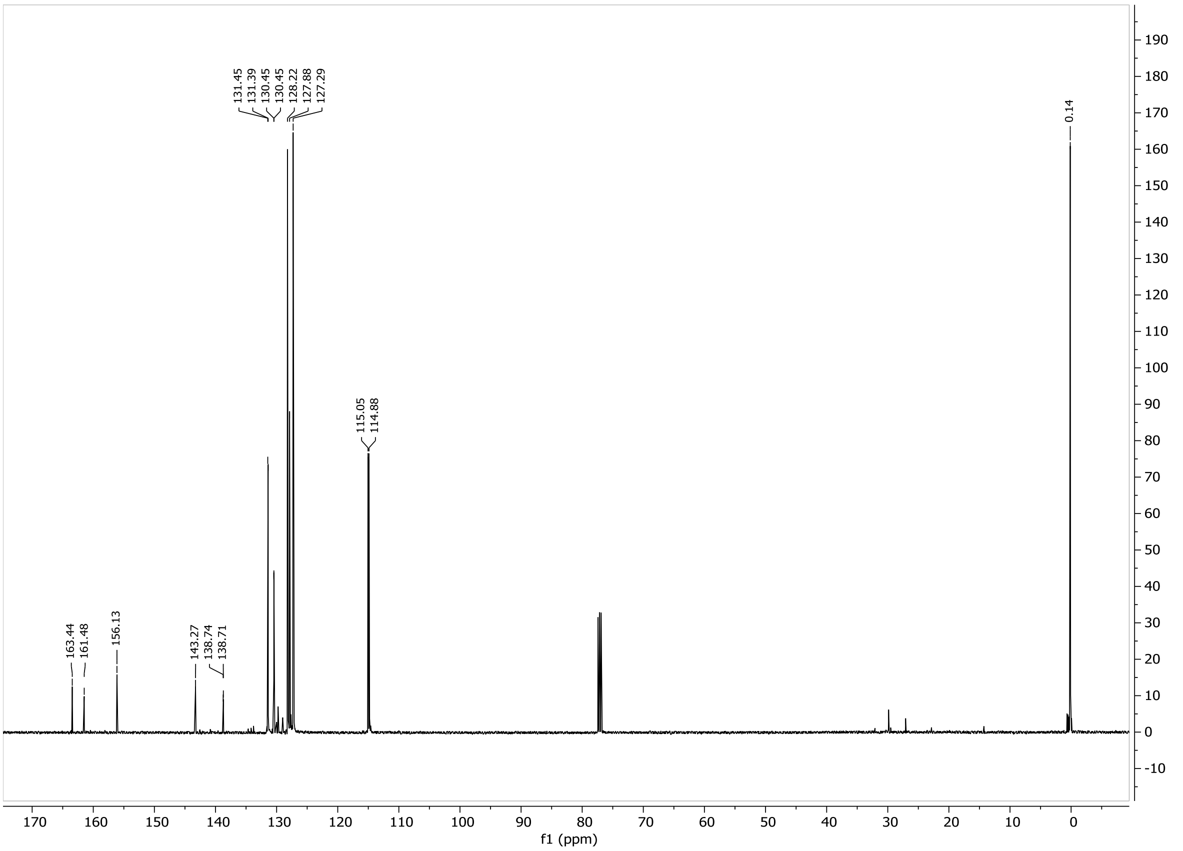

^{19}F NMR (564 MHz, $CDCl_3$)

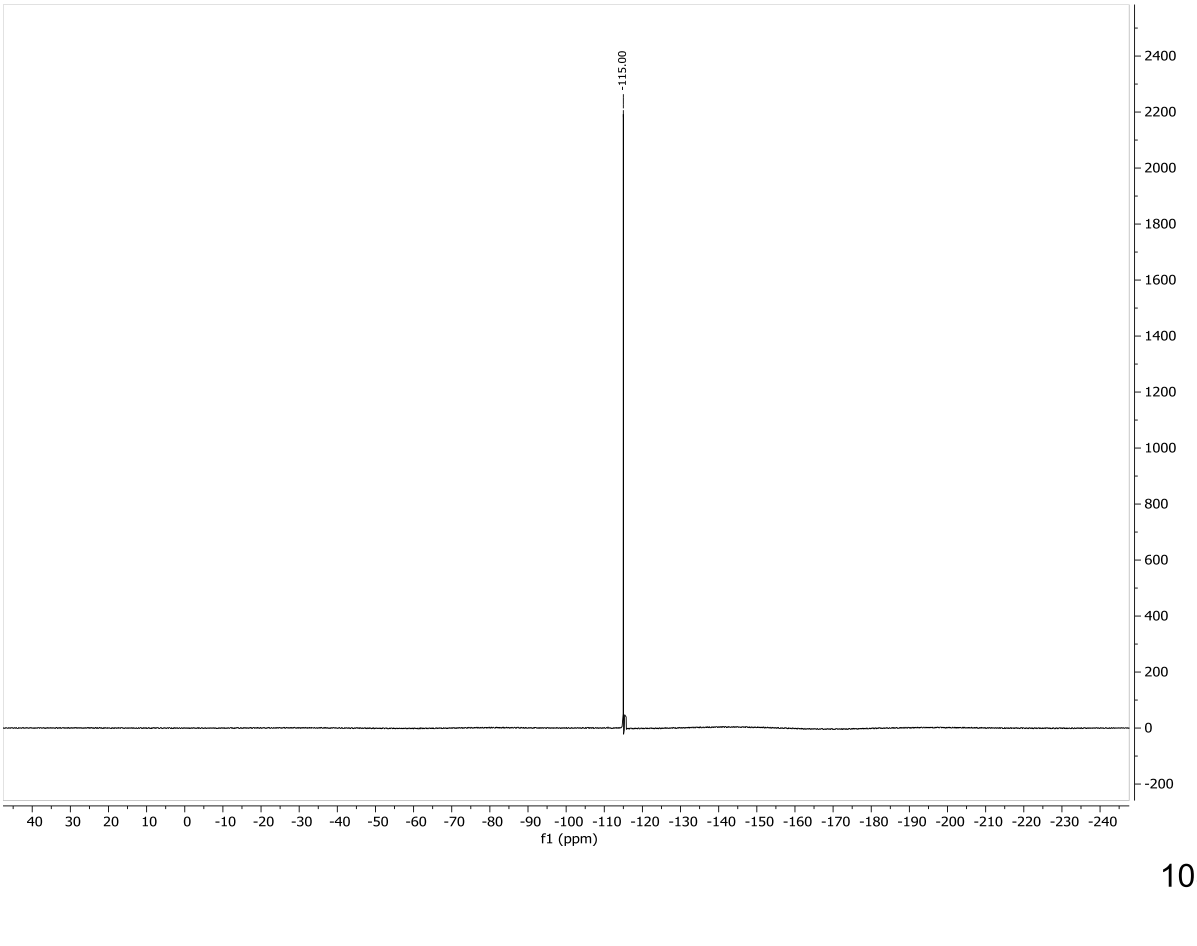

^{1}H NMR (600 MHz, $CDCl_3$)

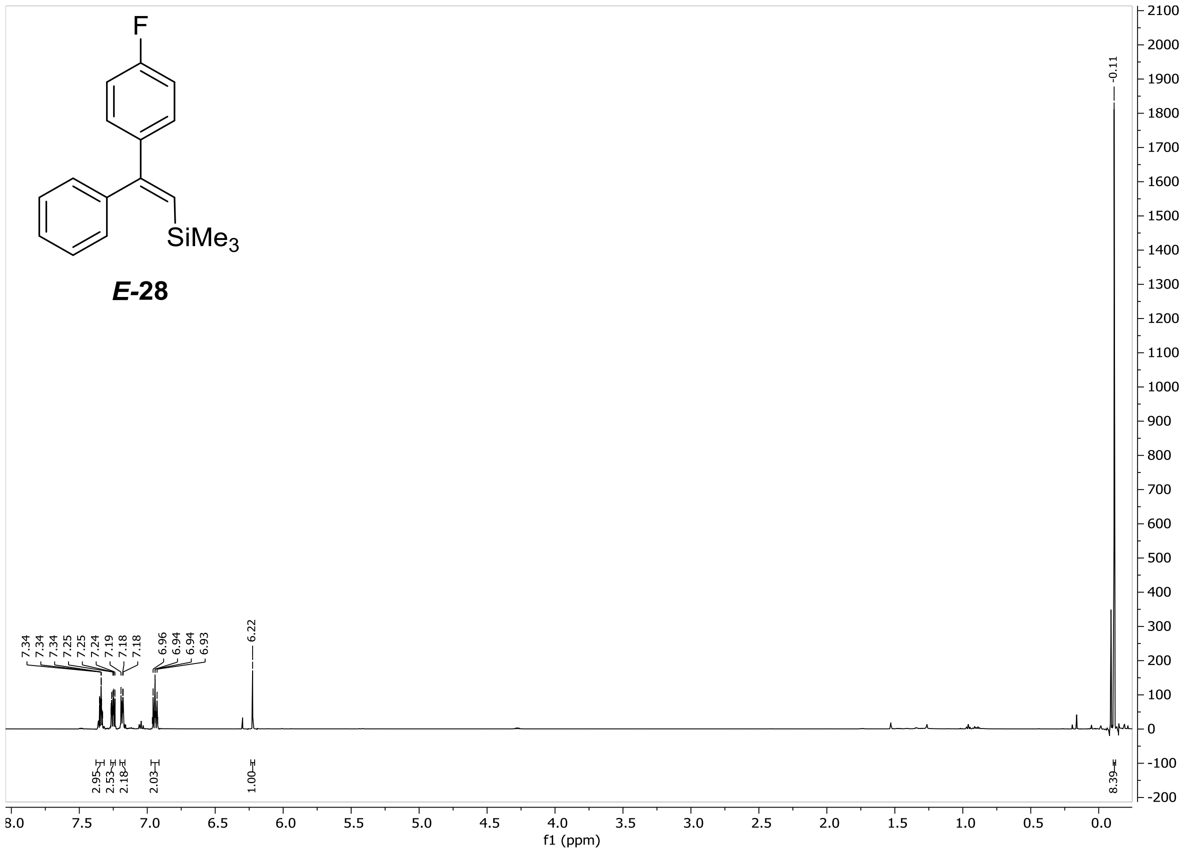

^{13}C NMR (151 MHz, $CDCl_3$)

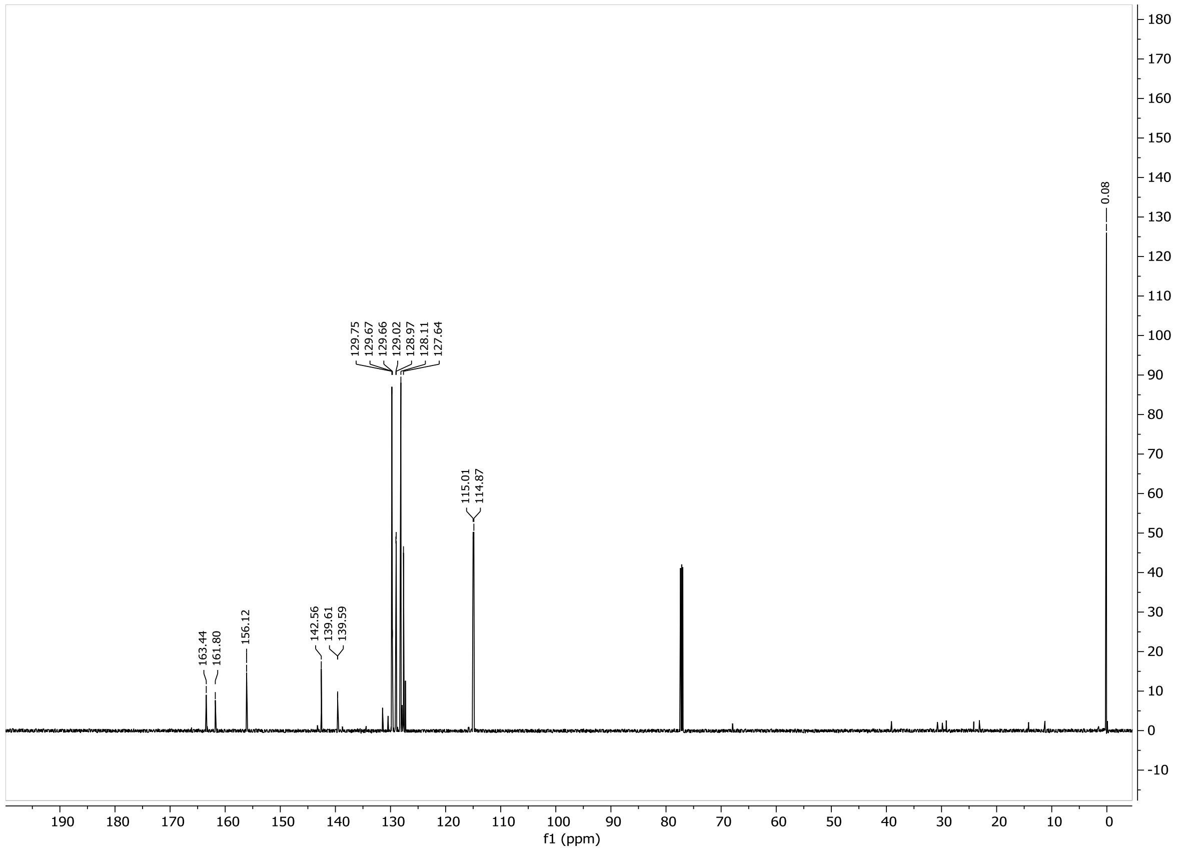

^{19}F NMR (564 MHz, $CDCl_3$)

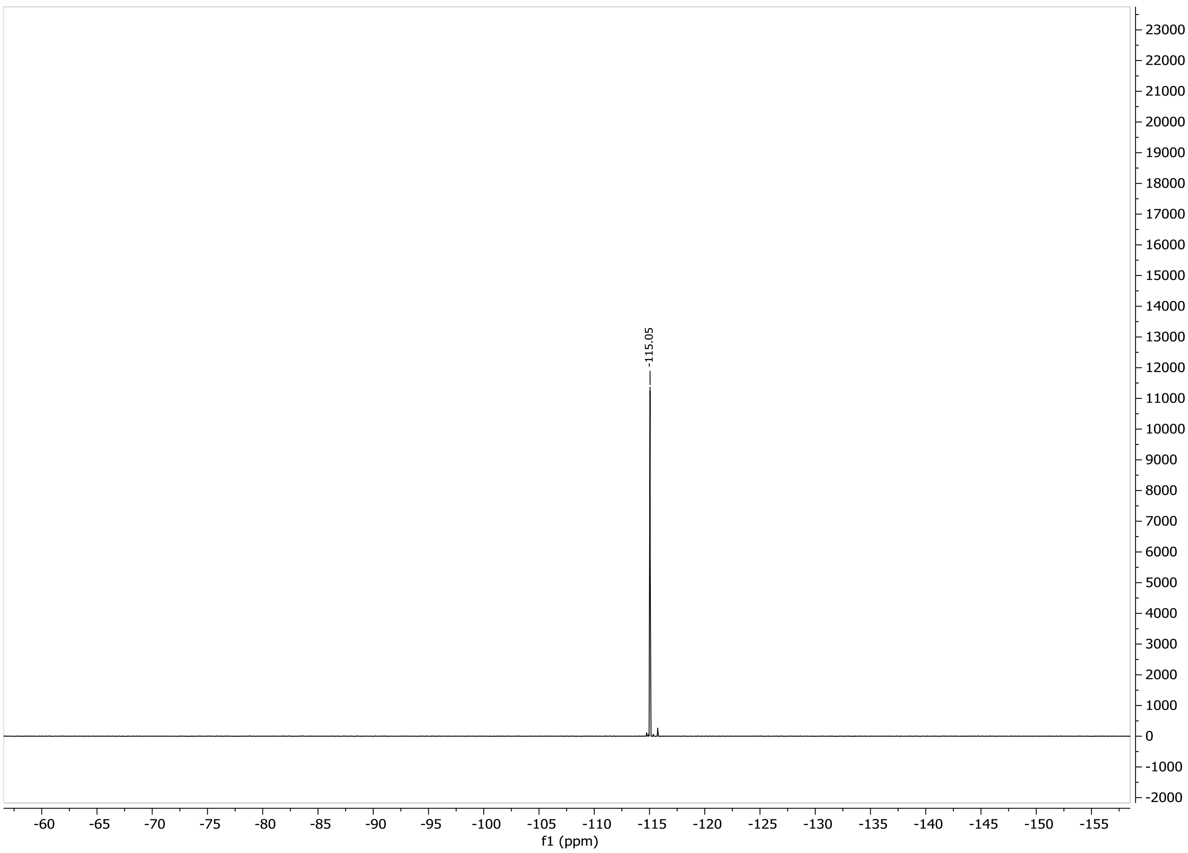

^{1}H NMR (600 MHz, $CDCl_3$)

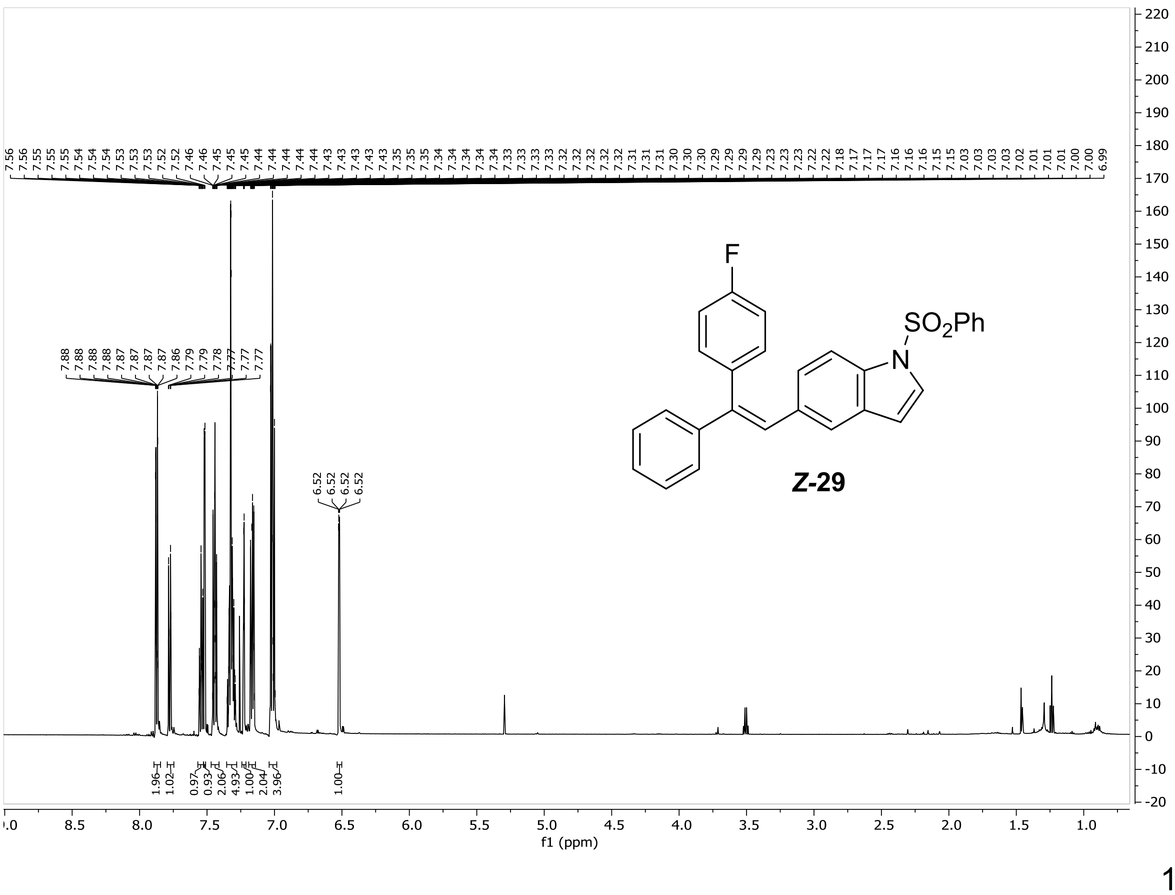

^{13}C NMR (151 MHz, $CDCl_3$)

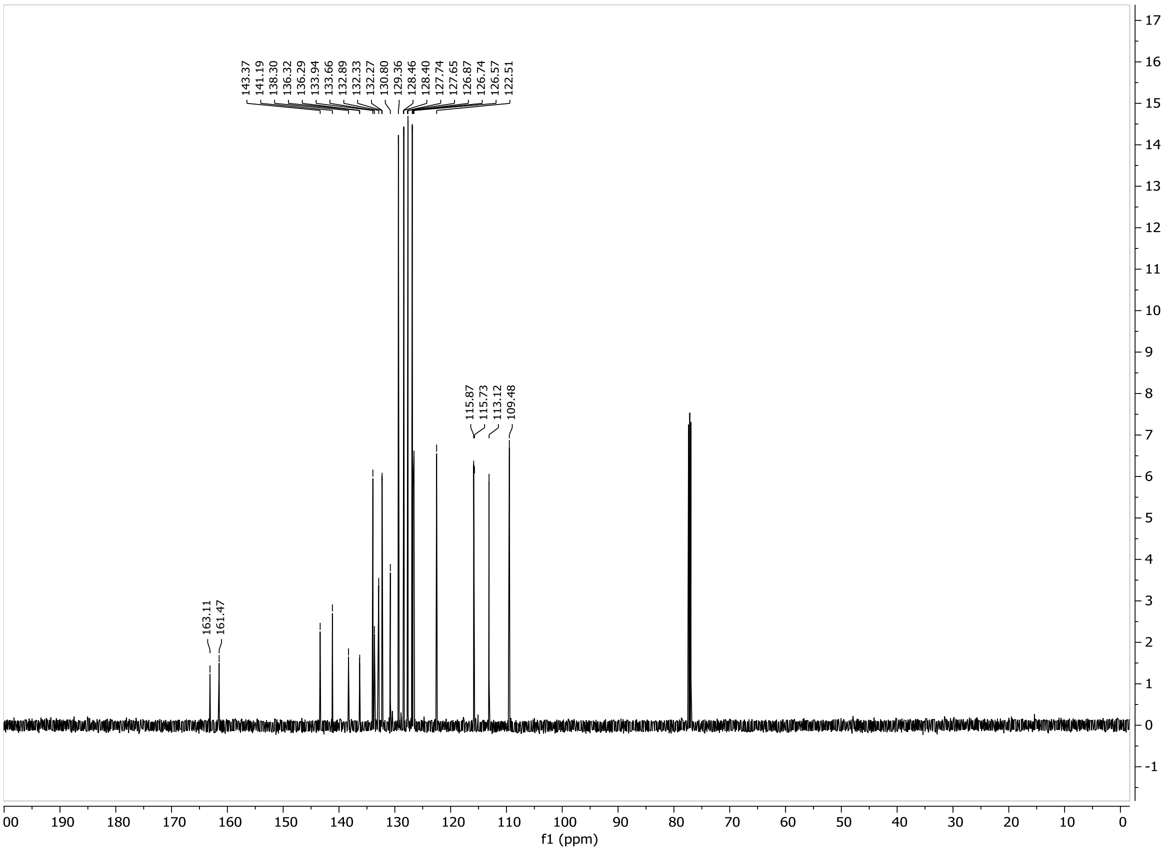

^{19}F NMR (564 MHz, $CDCl_3$)

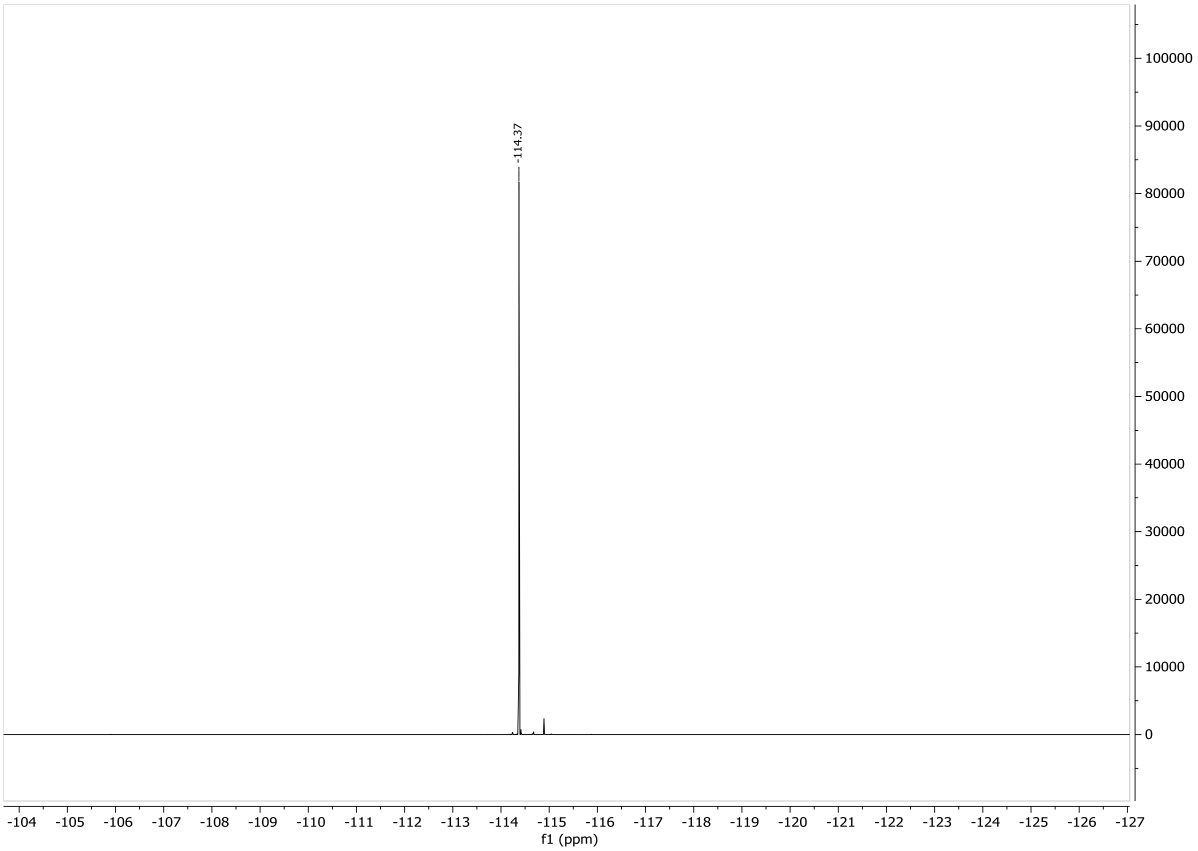

^{1}H NMR (600 MHz, $CDCl_3$)

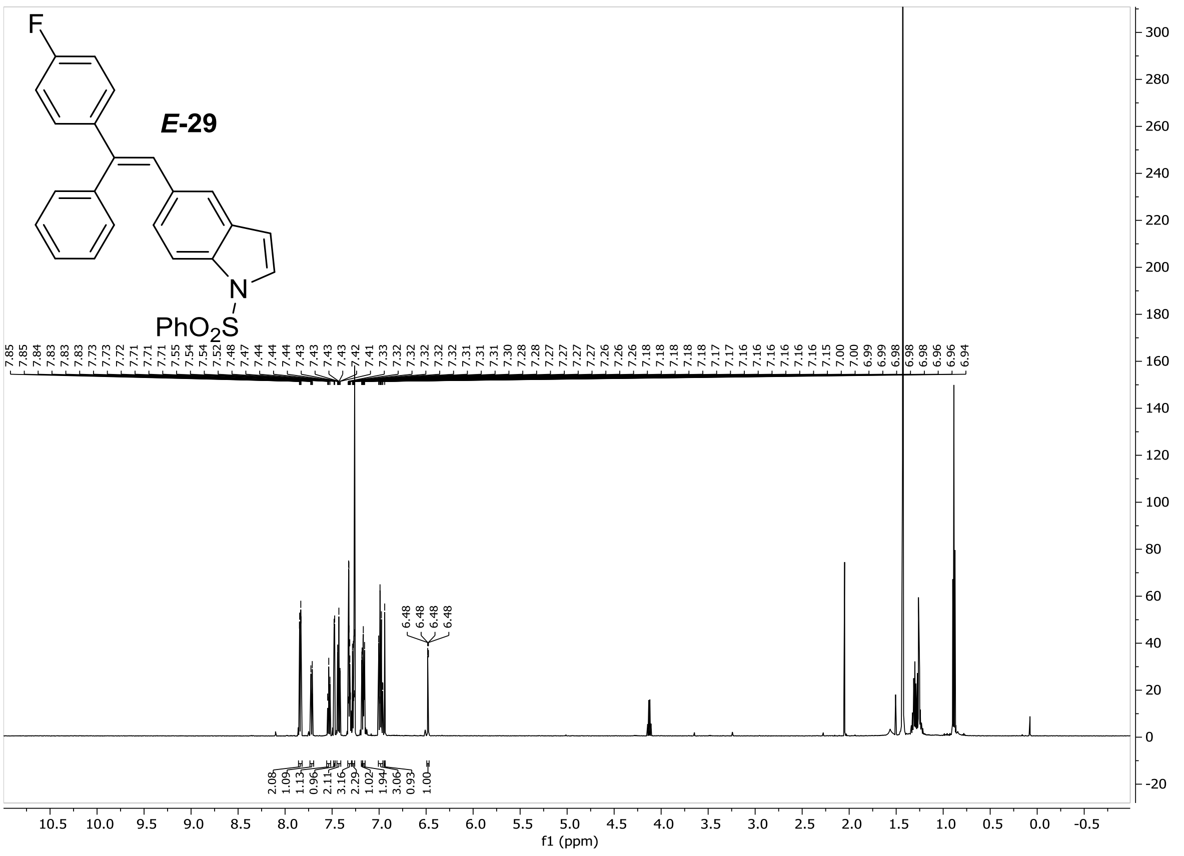

^{13}C NMR (151 MHz, $CDCl_3$)

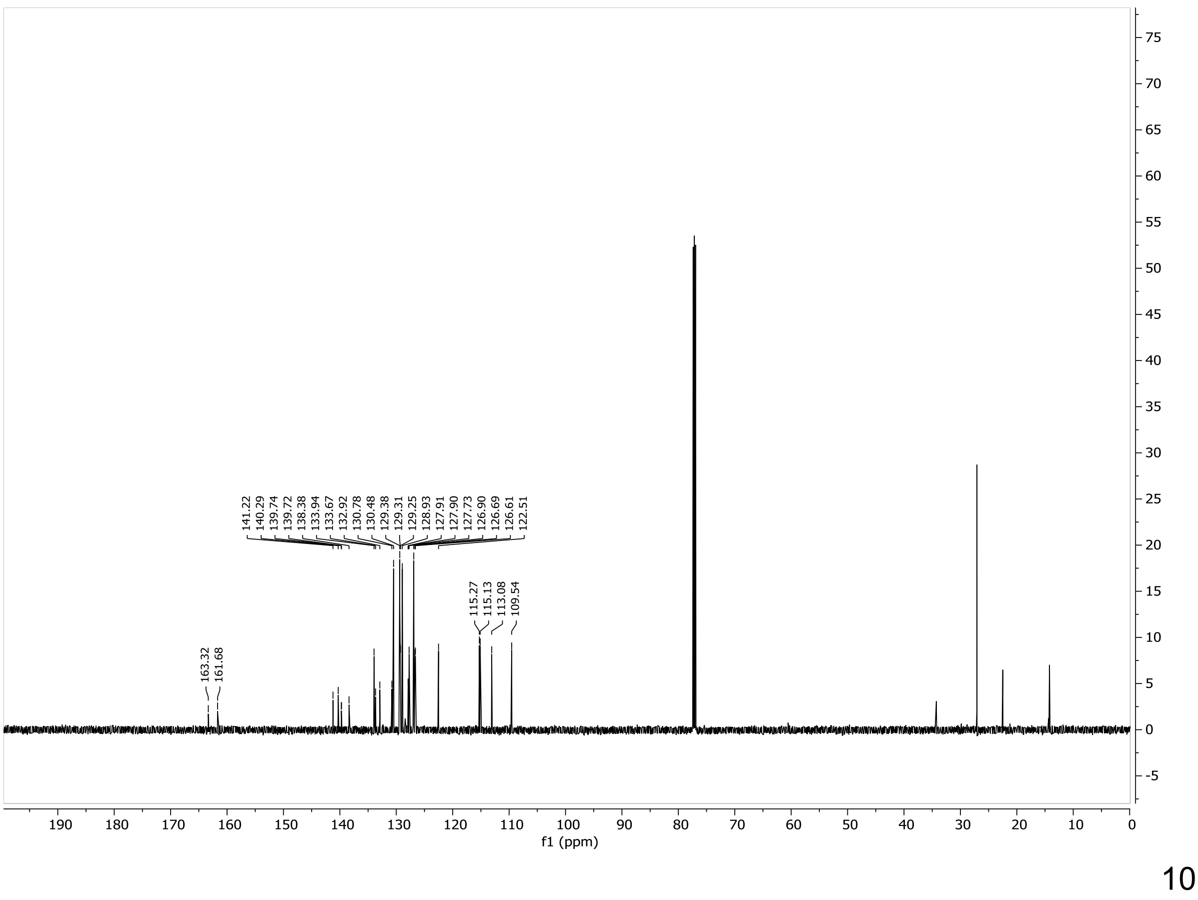

^{19}F NMR (564 MHz, $CDCl_3$)

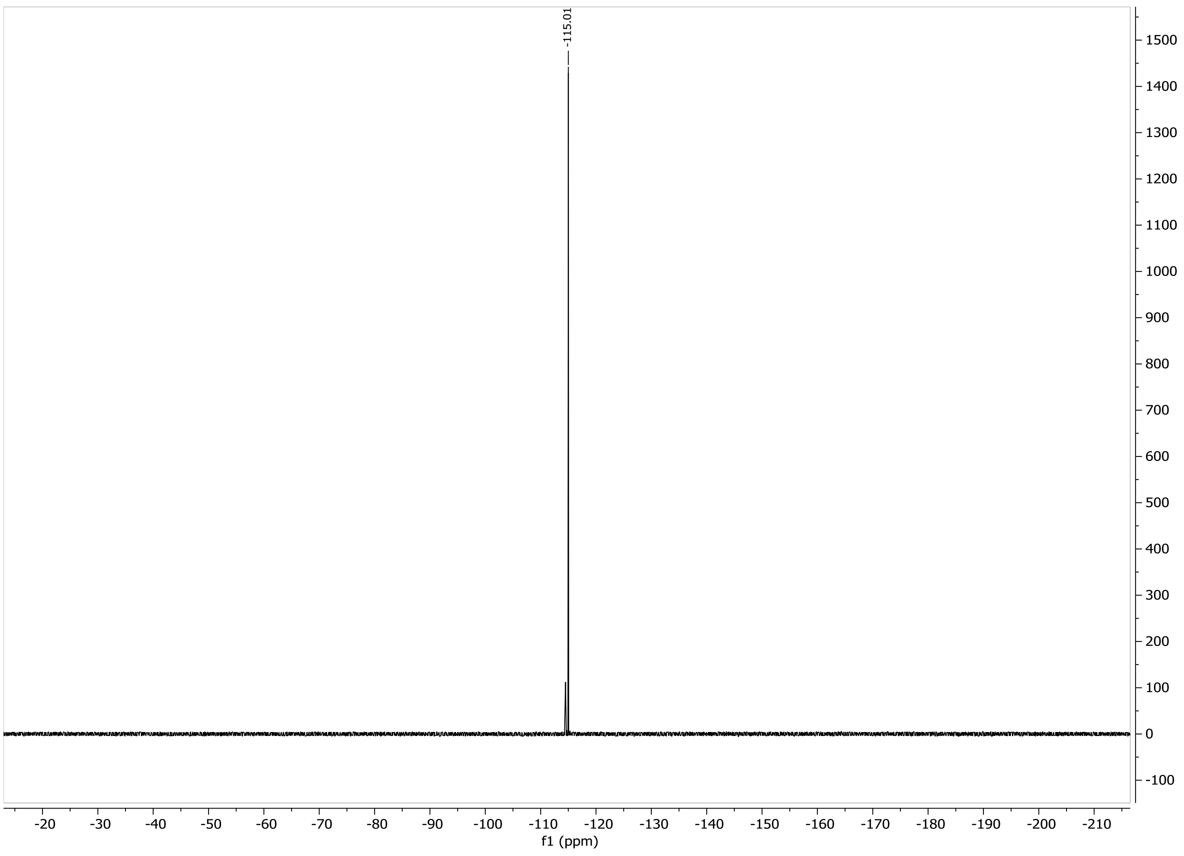

6. References

[1] J. J. Molloy, J. B. Metternich, C. G. Daniliuc, A. J. B. Watson, R. Gilmour, *Angew. Chemie - Int. Ed.* **2018**, *57*, 3168–3172.

[2] N. Chatani, N. Amishiro, T. Morii, T. Yamashita, S. Murai, *J. Org. Chem.* **1995**, *60*, 1834–1840.

[3] S. S. P. Chou, H. L. Kuo, C. J. Wang, C. Y. Tsai, C. M. Sun, *J. Org. Chem.* **1989**, *54*, 868–872.

[4] A. Boelke, L. D. Caspers, B. J. Nachtsheim, *Org. Lett.* **2017**, *19*, 5344–5347.

[5] X. Creary, M. A. Butchko, *J. Org. Chem.* **2002**, *67*, 112–118.

[6] S. Bratovanov, W. Koźmiński, J. Fässler, Z. Molnar, D. Nanz, S. Bienz, *Organometallics* **1997**, *16*, 3128–3134.

[7] T. Y. Luh, D. K. P. Ng, Z. J. Ni, Y. L. Tzeng, P. F. Yang, *J. Am. Chem. Soc.* **1990**, *112*, 9356–9364.

[8] D. Les, S. Sila-, G. Bertrand, C. Manuel, P. Mazerolles, *Tetrahedron* **1981**, *37*, 2875–2880.

[9] M. Belema, V. N. Nguyen, F. C. Zusi, *Tetrahedron Lett.* **2004**, *45*, 1693–1697.

[10] I. Hemeon, R. D. Singer, *J. Mol. Catal. A Chem.* **2004**, *214*, 33–44.

[11] I. Vitorica-Yrezabal, J. R. Lawson, V. Fasano, J. Cid, M. J. Ingleson, *Dalt. Trans.* **2016**, *45*, 6060–6070.

[12] R. Tanaka, H. Sanjiki, H. Urabe, *J. Am. Chem. Soc.* **2008**, *130*, 2904–2905.

[13] Y. Nishihara, D. Saito, K. Tanemura, S. Noyori, K. Takagi, *Org. Lett.* **2009**, *11*, 3546–3549.

[14] B. Huang, Z. Zhou, M. Z. Cai, *Chinese J. Chem.* **2006**, *24*, 1469–1471.

[15] Y. M. Chae, J. S. Bae, J. H. Moon, J. Y. Lee, J. Yun, *Adv. Synth. Catal.* **2014**, *356*, 843–849.

[16] S. E. Denmark, R. F. Sweis, D. Wehrli, *J. Am. Chem. Soc.* **2004**, *126*, 4865–4875.

[17] B. M. Trost, C. E. Stivala, D. R. Fandrick, K. L. Hull, A. Huang, C. Poock, R. Kalkofen, *J. Am. Chem. Soc.* **2016**, *138*, 11690–11701.

[18] Y. Nishihara, M. Miyasaka, M. Okamoto, H. Takahashi, E. Inoue, K. Tanemura, K. Takagi, *J. Am. Chem. Soc.* **2007**, *129*, 12634–12635.

[19] J. J. Mousseau, J. A. Bull, A. B. Charette, *Angew. Chemie - Int. Ed.* **2010**, *49*, 1115–1118.

[20] E. Negishi, D. E. Van Horn, T. Yoshida, *J. Am. Chem. Soc.* **1985**, *107*, 6639–6647.

[21] J. Tao, J. P. Perdew, V. N. Staroverov, G. E. Scuseria, *Phys. Rev. Lett.*, **2003**, *91*, 146401.

[22] a) S. Grimme. J. Antony. S. Ehrlich. H. Krieg. *J. Chem. Phys.* **2010**, *132*, 154104. b) S. Grimme. S. Ehrlich. L. Goerigk. *J. Comput. Chem.* **2011**, *32*, 1456–1465.

[23] F. Weigend. R. Ahlrichs. *Phys. Chem. Chem. Phys.* **2005**, *7*, 3297–3305.

[24] S. Grimme. *Chem. Eur. J.* **2012**, *18*, 9955-9964.

[25] L. Goerigk. S. Grimme. *J. Chem. Theory Comput.* **2011**, *7*, 291-309.

[26] a) A. D. Becke, *Phys. Rev. A.*, **1988**, *38*, 3098-3100. b) C. Lee, W. Yang, R.G. Parr, *Phys. Rev. B* **1988**, *37*, 785-789

[27] TURBOMOLE V7.3 (2018), a development of University of Karlsruhe and Forschungszentrum Karlsruhe GmbH, 1989-2007, TURBOMOLE GmbH, since 2007; available from *http://www.turbomole.com*.

[28] F. Neese. The ORCA Program System. *Wiley Interdisciplinary Reviews: Computational Molecular Science* **2012**, *2*, 73-78.
